# Health trends, inequalities and opportunities in South Africa’s provinces, 1990–2019: findings from the Global Burden of Disease 2019 Study

**DOI:** 10.1136/jech-2021-217480

**Published:** 2022-01-19

**Authors:** Tom Achoki, Benn Sartorius, David Watkins, Scott D Glenn, Andre Pascal Kengne, Tolu Oni, Charles Shey Wiysonge, Alexandra Walker, Olatunji O Adetokunboh, Tesleem Kayode Babalola, Obasanjo Afolabi Bolarinwa, Mareli M Claassens, Richard G Cowden, Candy T Day, Oluchi Ezekannagha, Themba G Ginindza, Chidozie C D Iwu, Chinwe Juliana Iwu, Innocent Karangwa, Patrick DMC Katoto, Nuworza Kugbey, Desmond Kuupiel, Phetole Walter Mahasha, Tivani Phosa Mashamba-Thompson, George A Mensah, Duduzile Edith Ndwandwe, Chukwudi A Nnaji, Mpiko Ntsekhe, Thomas Elliot Nyirenda, Julius Nyerere Odhiambo, Kwaku Oppong Asante, Charles D H Parry, Julian David Pillay, Aletta Elisabeth Schutte, Soraya Seedat, Karen Sliwa, Dan J Stein, Frank C Tanser, Ushotanefe Useh, Heather J Zar, Liesl J Zühlke, Bongani M Mayosi, Simon I Hay, Christopher J L Murray, Mohsen Naghavi

**Affiliations:** 1 Research, Africa Institute for Health Policy, Nairobi, Kenya; 2 Center for Pharmaceutical Policy and Regulation, Utrecht University, Utrecht, Netherlands; 3 Centre for Tropical Medicine and Global Health, University of Oxford, Oxford, UK; 4 Nuffield Department of Medicine, University of Oxford, Oxford, UK; 5 Department of Health Metrics Sciences, School of Medicine, University of Washington, Seattle, Washington, USA; 6 Department of Medicine, University of Washington, Seattle, WA, USA; 7 Department of Medicine, University of Cape Town, Cape Town, South Africa; 8 Institute for Health Metrics and Evaluation, University of Washington, Seattle, WA, USA; 9 Non-communicable Diseases Research Unit, Medical Research Council South Africa, Cape Town, South Africa; 10 MRC Epidemiology Unit, University of Cambridge, Cambridge, UK; 11 School of Public Health and Family Medicine, University of Cape Town, Rondebosch, Western Cape, South Africa; 12 Cochrane South Africa, South African Medical Research Council, Cape Town, South Africa; 13 Centre of Excellence for Epidemiological Modelling and Analysis, Stellenbosch University, Stellenbosch, South Africa; 14 Department of Global Health, Stellenbosch University, Stellenbosch, South Africa; 15 Discipline of Public Health Medicine, University of KwaZulu-Natal, Durban, South Africa; 16 Department of Community Health and Primary Care, University of Lagos, Lagos, Nigeria; 17 Department of Biochemistry and Microbiology, University of Namibia, Windhoek, Namibia; 18 Department of Paediatrics and Child Health, Stellenbosch University, Tygerberg, South Africa; 19 Department of Psychology, University of the Free State, Park WEst, Free State, South Africa; 20 Health Systems Research Unit, Health System Trust, Westville, South Africa; 21 Independent Consultant, Awka, Nigeria; 22 International Institute of Tropical Agriculture, Ibadan, Nigeria; 23 School of Health Systems and Public Health, University of Pretoria, Pretoria, South Africa; 24 South African Medical Research Council, Cape Town, South Africa; 25 Department of Statistics and Population Studies, University of the Western Cape, Cape Town, South Africa; 26 Centre for Tropical Diseases and Global Health, Catholic University of Bukavu, Bukavu, Democratic Republic of Congo; 27 University of Environment and Sustainable Development, Somanya, Ghana; 28 School of Nursing and Public Health, University of KwaZulu-Natal, Durban, South Africa; 29 Department of Nursing, Research for Sustainable Development Consult, Sunyani, Ghana; 30 Grants, Innovation and Product Development Unit, South African Medical Research Council, Cape Town, South Africa; 31 Faculty of Health Sciences, University of Pretoria, Pretoria, South Africa; 32 Center for Translation Research and Implementation Science, National Institutes of Health, Bethesda, Maryland, USA; 33 Division of Cardiology, University of Cape Town, Rondebosch, Western Cape, South Africa; 34 The Cardiac Clinic, Groote Schuur Hospital, Cape Town, South Africa; 35 European and Developing Countries Clinical Trials Partnership (EDCTP), European Commission, Cape Town, South Africa; 36 Department of Management Science and Technology, Technical University of Kenya, Nairobi, Nairobi, Kenya; 37 Department of Psychology, University of Ghana, Accra, Ghana; 38 Alcohol, Tobacco & Other Drug Research Unit, South African Medical Research Council, Cape Town, South Africa; 39 Department of Psychiatry, Stellenbosch University, Stellenbosch, South Africa; 40 Department of Basic Medical Sciences, Durban University of Technology, Durban, South Africa; 41 School of Public Health and Community Medicine, University of New South Wales, Sydney, New South Wales, Australia; 42 The George Institute for Global Health, Sydney, New South Wales, Australia; 43 Hatter Institute Department of Medicine, University of Cape Town, Cape Town, South Africa; 44 Unit on Risk and Resilience in Mental Disorders, South African Medical Research Council, Cape Town, South Africa; 45 University of KwaZulu-Natal, Durban, South Africa; 46 Africa Health Research Institute, Berea, South Africa; 47 Health Sciences Department, North-West University, Mmbatho, South Africa; 48 Department of Paediatrics and Child Health, University of Cape Town, Cape Town, South Africa; 49 Unit on Child & Adolescent Health, Medical Research Council South Africa, Cape Town, South Africa; 50 University of Cape Town, Cape Town, South Africa

**Keywords:** HIV, healthcare disparities, health policy, public health

## Abstract

**Background:**

Over the last 30 years, South Africa has experienced four ‘colliding epidemics’ of HIV and tuberculosis, chronic illness and mental health, injury and violence, and maternal, neonatal, and child mortality, which have had substantial effects on health and well-being. Using data from the 2019 Global Burden of Diseases, Injuries and Risk Factors Study (GBD 2019), we evaluated national and provincial health trends and progress towards important Sustainable Development Goal targets from 1990 to 2019.

**Methods:**

We analysed GBD 2019 estimates of mortality, non-fatal health loss, summary health measures and risk factor burden, comparing trends over 1990–2007 and 2007–2019. Additionally, we decomposed changes in life expectancy by cause of death and assessed healthcare system performance.

**Results:**

Across the nine provinces, inequalities in mortality and life expectancy increased over 1990–2007, largely due to differences in HIV/AIDS, then decreased over 2007–2019. Demographic change and increases in non-communicable diseases nearly doubled the number of years lived with disability between 1990 and 2019. From 1990 to 2019, risk factor burdens generally shifted from communicable and nutritional disease risks to non-communicable disease and injury risks; unsafe sex remained the top risk factor. Despite widespread improvements in healthcare system performance, the greatest gains were generally in economically advantaged provinces.

**Conclusions:**

Reductions in HIV/AIDS and related conditions have led to improved health since 2007, though most provinces still lag in key areas. To achieve health targets, provincial governments should enhance health investments and exchange of knowledge, resources and best practices alongside populations that have been left behind, especially following the COVID-19 pandemic.

## Introduction

Since the end of apartheid in 1994, South Africa has experienced dramatic and rapid changes in population health.^1^ The first years of postapartheid South Africa were characterised by economic growth and notable steps towards reducing inequalities in health and high levels of mortality from infectious and maternal causes.^2^ Unfortunately, the emergence of the HIV/AIDS epidemic during the early 2000s, prior to the rollout of antiretroviral drug therapy (ART), resulted in massive shocks to the health system and reversed many previous gains in health.^3^ Concerted efforts by successive administrations, coupled with international support, have stabilised the epidemic and have begun to shift health policy towards longer-term strategies and goals that now include a wider range of priorities such as non-communicable disease (NCD) prevention and National Health Insurance (NHI)—South Africa’s approach to achieving universal health coverage.^4^

In 2009, *The Lancet* launched a widely read Series on Health in South Africa that influenced government, academia, and civil society with recommendations for priority diseases as well as structural reforms in the areas of financing (ie, through NHI), disease surveillance and research and innovation.^5^ South Africa’s health challenges were framed, then and since, as ‘colliding epidemics’ and the ‘quadruple burden of disease’—the latter encompassing hyperendemic HIV/AIDS with a concurrent high burden of tuberculosis; high rates of violence and injuries; persistently high levels of maternal and child mortality; and the steady rise of NCDs.^4^ Since that time, estimates of mortality by cause and province have been released by the Burden of Disease Research Unit (BoDRU)-SAMRC, showing relatively modest declines in mortality from ‘quadruple burden’ conditions, except for HIV/AIDS mortality, which declined rapidly after 2007.^1^

Underlying these national-level trends are marked subnational (provincial) health inequalities. While South Africa spends more on health than any other African country, studies suggest that improvements in health outcomes are not proportional to the amount spent.^5^ Like many other countries, South Africa is a federal system, and health is a provincial competency wherein provincial governments determine their own health priorities. Arguably, this has contributed to some of the observed interprovincial variations in health outcomes, especially in more recent years.^6^ However, the legacy of the racist apartheid regime continues to influence health outcomes, particularly relating to drivers of population, cross-provincial socioeconomic inequalities and thus differences in health funding and health system development.^7 8^ Large national surveys have demonstrated important variations across provinces in health-related behaviours and in the distribution of various risk factors for disease.^9 10^ Studies have also recorded inequities in health service delivery and utilisation between urban and rural settings and across various racial groups—and by inference, across the nine provinces, which differ in terms of sociodemographics and geography.^11^ These findings substantiate efforts to estimate disease burden, including trends and disparities at subnational scale.

The problem of subnational inequalities in health is not unique to South Africa. Many other large, heterogeneous countries (eg, India, China and Brazil) have considerable variations in health status across provinces or states. For example, most of the world’s ‘poorest billion’ actually live in pockets of poverty in middle-income countries, not in low-income countries. Measuring health levels, trends and drivers at the subnational level is of great interest to international researchers and policy-makers seeking to reduce health inequalities within federal systems. To this end, the Global Burden of Disease (GBD) studies have begun to produce subnational health estimates for countries with large populations. The GBD 2019 Study included an expanded set of countries, including South Africa, for which subnational estimates of diseases, injuries and risk factors were produced. GBD 2019 offers substantial improvements in methods and data sources for producing global health estimates as compared with prior GBD studies, including improvements in estimates for South Africa and its nine provinces. This paper, which is based on GBD 2019 estimates, provides an overview of policy relevant trends of national and provincial-level health loss due to fatal and non-fatal causes and related risk factors. It explores the temporal trends and transitions from the last years of the apartheid regime (1990) to the present day (2019), highlighting progress towards key Sustainable Development Goal (SDG) targets. Additionally, the paper identifies areas of progress as well as emerging and persistent threats to health and subnational health equality in South Africa and thus providing a basis for appropriate and targeted policy interventions.

## Methods

The analytic framework, methods and data sources used for GBD 2019 are described extensively in prior publications.^12–14^ This study complies with GATHER recommendations (online supplemental table S1). Briefly, GBD is a comprehensive effort to identify and use all available data sources, evaluate quality and correct for known biases in each source, implement standardised statistical methods, and produce estimates with 95% uncertainty intervals (UIs) that are propagated throughout all stages of modelling. This report includes estimates of fatal and non-fatal health outcomes and risk factors as well as summary measures of health—years of life lost (YLLs), years lived with disability (YLDs), disability-adjusted life-years (DALYs) and healthy life expectancy (HALE).

10.1136/jech-2021-217480.supp1 Supplementary data

We analysed subnational data in our models by treating provinces as unique geographies nested hierarchically within South Africa.^15^ We drew on census data (1951, 1960, 1970, 1980, 1985, 1991, 1996, 2001 and 2011) and a wide range of data sources including surveys, vital registration (VR) data, disease surveillance data, registries and published scientific literature. A detailed list of data sources used for the GBD South Africa estimates can be found at the Global Health Data Exchange (GHDx)^16^ and in online supplemental appendix 1. Statistical methods relevant to this paper are summarised briefly in the following paragraphs and described in detail in online supplemental information section 2 with references in online supplemental information section 5.^12–14^

We used demographic methods to estimate all-cause, under-5 and adult mortality based on surveys and census data. We then used various South African data sources including provincial-level VR data (1997–2016) in GBD 2019 to produce cause-specific mortality fractions for 286 unique causes of death after correcting for incomplete registration and ‘garbage-coded’ causes. Data were analysed using the Cause of Death Ensemble model, which employs a wide range of predictive models that draw on combinations of covariates correlated with specific causes of death and whose composition is determined by tests of in-sample and out-of-sample performance. Further adjustments were made for discontinuities in long-term trends, including HIV/AIDS as well as improvements in implied pattern of VR completeness by age and sex (online supplemental information section 2). Mortality rates were then scaled to all-cause mortality estimates. Finally, we calculated YLLs and age-standardised rates using a global reference life table.

To estimate non-fatal outcomes (incidence, prevalence, and YLDs), data sources of relevance to South Africa were mapped to 369 unique causes of disease or injury and 3473 unique sequelae. Data were analysed in DisMod-MR 2.1, a Bayesian meta-regression tool that produces internally consistent estimates of disease parameters (incidence, prevalence, remission, excess mortality and cause-specific mortality). The prevalence or incidence of each sequela was then multiplied by its corresponding disability weight to calculate YLDs, and sequelae were aggregated by disease or injury to obtain total YLDs for each cause.

DALYs were calculated by summing YLLs and YLDs for each cause. Separately, the disease burden attributable to specific risk factors was calculated for each cause using the comparative risk assessment method. HALE was calculated using multiple decrement life tables that incorporated estimates of per-capita YLDs. All estimates were produced separately by 5-year age interval, sex, province and year. For the purposes of this report, we generally present estimates for 1990, 2007 and 2019; 2007 is highlighted here as an approximate turning point (peak) in HIV/AIDS-related mortality in South Africa.

We assessed provincial-level health system performance using the Healthcare Access and Quality Index (HAQ Index), a measure developed for the GBD studies that incorporates age-standardised and risk-standardised mortality rates for 32 causes that are amenable to healthcare.^17^ The HAQ Index is scaled from 0 to 100 across all countries (including subnational units) and years included in the GBD study, allowing direct comparisons across locations and time. We compared HAQ Index values and changes over 1990–2019 among the nine provinces and compared with other Southern African Development Community (SADC) countries. This analytical multilevel approach helps contextualise and benchmark the performance of the South African health system subnationally, nationally and regionally, in the efforts to improve population access to healthcare.

Finally, we also assessed progress towards achievement of key health-related SDG targets (namely: maternal, neonatal mortality and under-5 mortality; HIV and tuberculosis incidence and lastly NCD mortality) in South Africa and at provincial level. We assessed observed annualised rate of change (ARC) in the indicator over 2015–2019, the start of the SDG period and contextualised this ARC in terms of that would be required to achieve SDG target by 2030, both nationally and by province.

This paper summarises key findings from our analysis of GBD 2019 estimates. Data files containing all GBD 2019 subnational estimates are available on the GHDx (http://ghdx.healthdata.org/gbd-2019).^16^ International Classification of Disease codes mapped to the GBD cause list are found in online supplemental table S6. Select additional results are presented in online supplemental information section 6; all additional results can be explored through online interactive data visualisations (http://www.healthdata.org/gbd/data-visualizations).

## Results

### Mortality levels and trends

**Figure 1:**
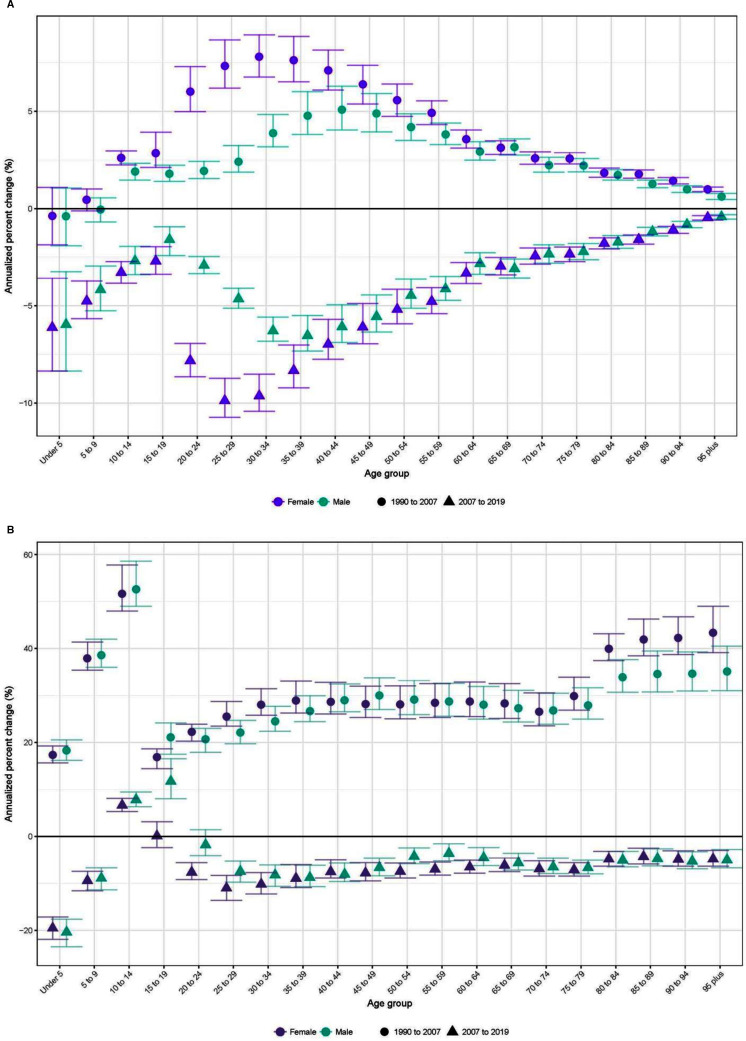
Annualised per cent change in age-specific and sex-specific mortality rates, 1990–2007 and 2007–2019. (A) all-cause mortality. (B) HIV/AIDS-specific mortality.

The number of deaths in South Africa increased from 293 904 deaths (95% 280 307–308 060) in 1990 to 724 828 deaths (667 154–797 221) in 2007, then declined to 521 802 deaths (494 683–554 967) in 2019. Trends in all-cause mortality by age and sex (figure 1A) tracked reasonably closely with trends in HIV/AIDS-specific mortality (figure 1B), with increases in both between 1990 and 2007 and decreases in both between 2007 and 2019. Changes in all-cause mortality were most pronounced in working-age adults. The exception to these overall positive trends was a modest, although noteworthy, increase in mortality among adolescent males and females over 2007–2019.

A major theme at the cross-provincial level was that inequalities in age-specific death rates widened in the 1990–2007 period and narrowed in the 2007–2019 period, owing in large part to the larger impact of HIV/AIDS in some provinces. We summarise trends in age-specific mortality using age-standardised mortality rates (ASMR). The largest ASMR increases in the earlier period were estimated in Free State (from 1243.6 (1143.6–1341.2) to 2593.2 (2438.3–2794) deaths per 100,000) and Mpumalanga (from 923.2 (846.4–1009.4) to 2108.7 (1943.4–2343.4)) deaths per 100 000). The largest decreases in the latter period were estimated in Free State (a decrease of 1158.8 deaths per 100 000), followed by KwaZulu-Natal, Mpumalanga and Northern Cape (decreases of 981.2, 945.4 and 856.0 deaths per 100,000, respectively). HIV/AIDS-specific mortality followed a similar trend, with increases in HIV/AIDS mortality across all provinces from 1990 to 2007, especially in KwaZulu-Natal and Free State. In the 2007–2019 period, all provinces saw a decline in HIV/AIDS mortality rates (online supplemental figure S1 and table S3).

Steady improvements in under-5 mortality rates (U5MR) and the maternal mortality ratio (MMR) were observed across all provinces, with acceleration of declines after 2007 (online supplemental figure S2 and S3, respectively). While all provinces saw improvements, inequalities persisted into 2019: U5MR ranged from 20.1 deaths (16.9–24.1) per 1000 in Western Cape to 51.6 per 1000 (44.1–61.1) in Free State. Similarly, MMR ranged from 39.8 (20.2–67.7) per 100 000 live births in Western Cape and 116 (44.1–208.6) in Mpumalanga (the next lowest) to 251.5 (114.9–421.2) in North-West.

**Figure 2:**
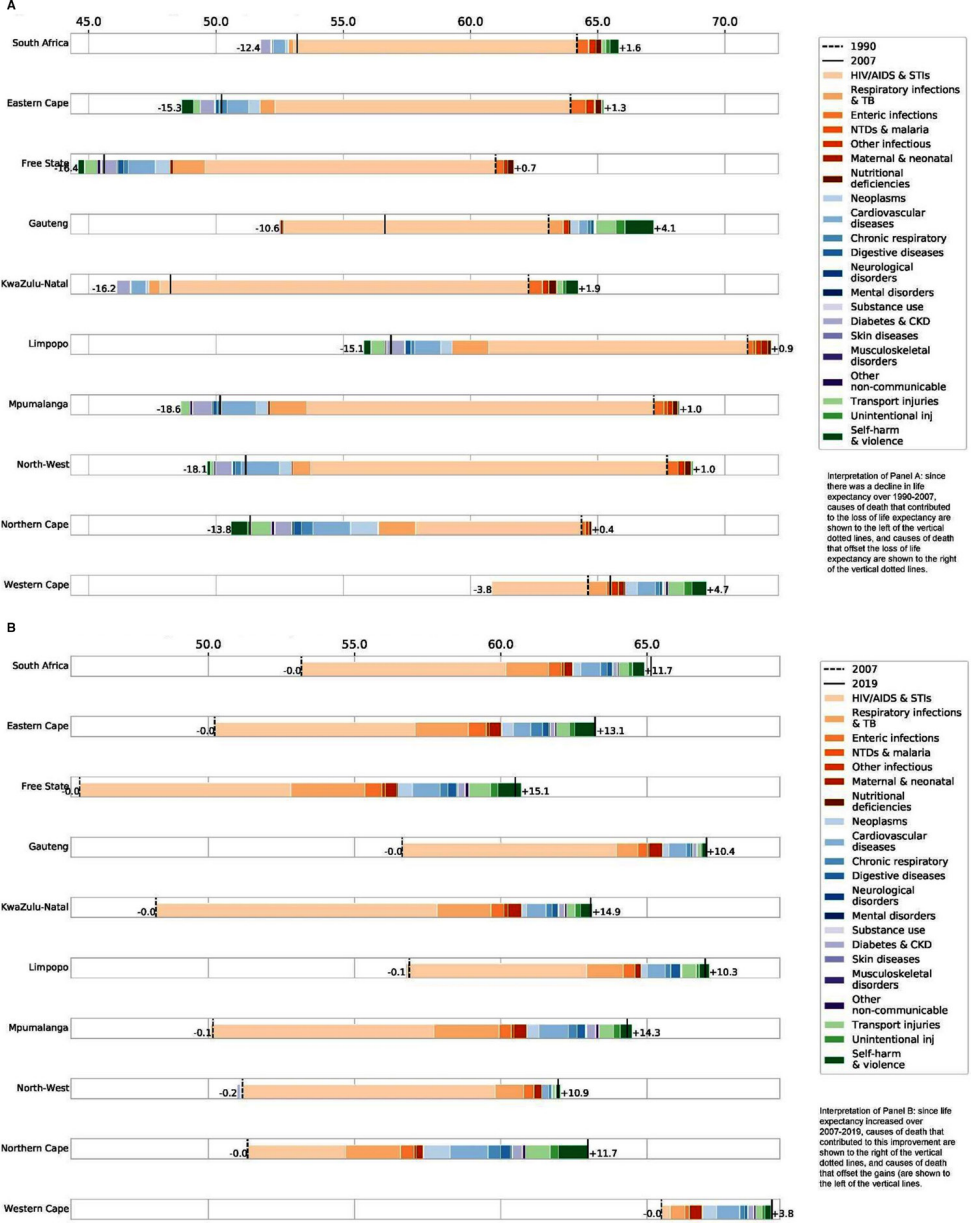
Contribution of various causes of death to changes in life expectancy (both sexes combined) by province. (A) Change in life expectancy between 1990 and 2007. (B) change in life expectancy between 2007 and 2019. In either panel, the vertical dotted lines denote life expectancy at the beginning of the period, and the solid vertical lines denote life expectancy at the end of the period. The figure is interpreted as follows. (A) since there was a decline in life expectancy over 1990–2007, causes of death that contributed to the loss of life expectancy are shown to the left of the vertical dotted lines, and causes of death that offset the loss of life expectancy are shown to the right of the vertical dotted lines. (B) since life expectancy increased over 2007–2019, causes of death that contributed to this improvement are shown to the right of the vertical dotted lines, and causes of death that offset the gains are shown to the left of the vertical lines. STIs-sexually transmitted infections; TB-tuberculosis; NTDs-neglected tropical diseases; CKD-chronic kidney disease.

Changes in life expectancy—declines during 1990–2007, then increases during 2007–2019—differed by province due to differences in cause-specific mortality levels and trends (figure 2). In the earlier period, the HIV/AIDS epidemic caused a significant decline in life expectancy in all provinces, though this was partially offset by progress in other communicable, maternal, neonatal and nutritional diseases and injuries; lack of progress on NCDs contributed to the life expectancy losses in all provinces except Western Cape (figure 2A). In the later period, all provinces saw an increase in life expectancy due to reductions in HIV/AIDS mortality, complemented by modest improvements in mortality from other causes, including NCDs and injuries (figure 2B). The contribution of various causes of death to changes in life expectancy by sex can be found in online supplemental figure S6.

### Trends in nonfatal outcomes

**Figure 3:**
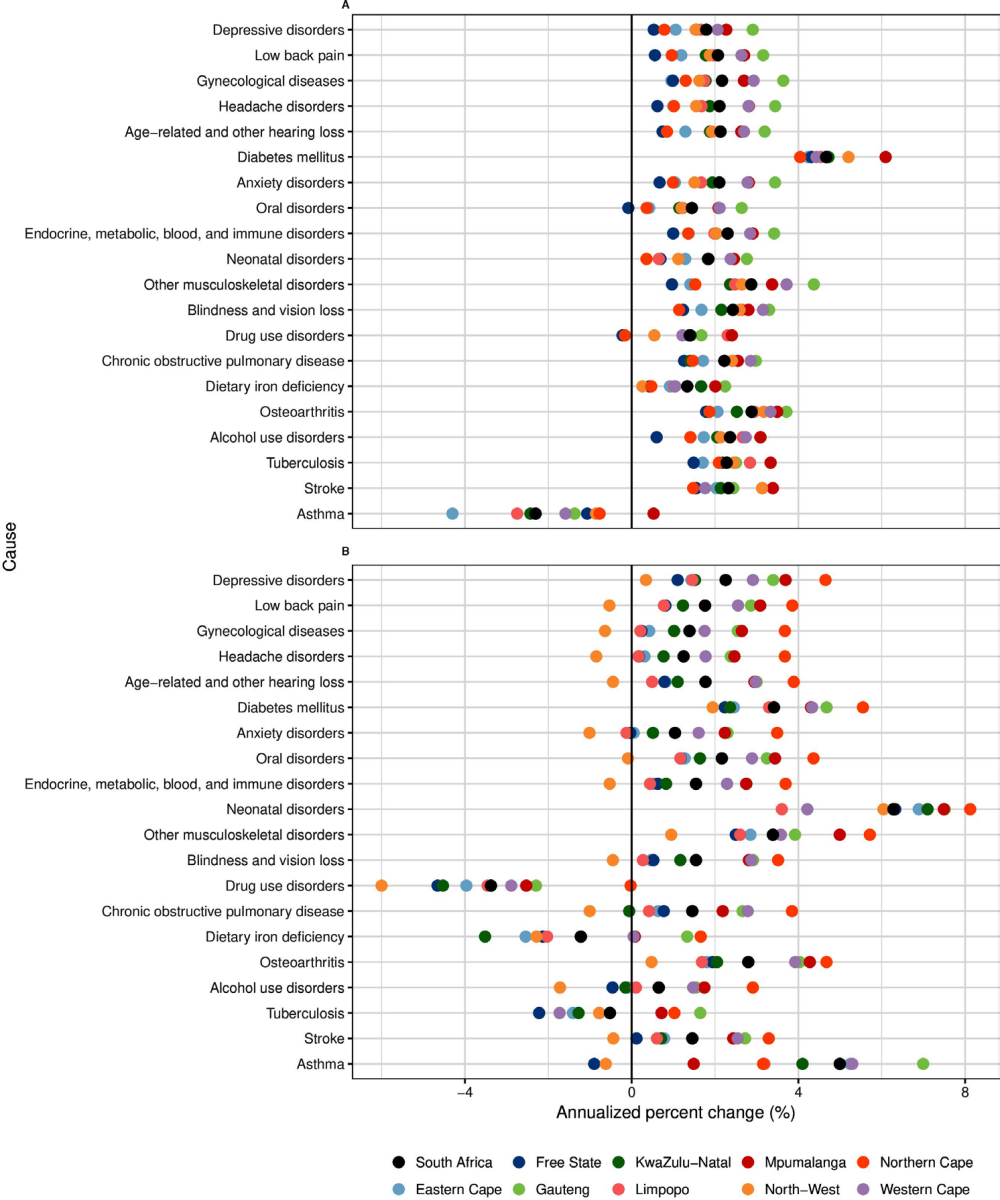
Annualised per cent change in YLDs (all ages, both sexes) by province, 1990–2019. The 21 leading causes of YLDs in 2019 (in descending order) are shown here, except for HIV/AIDS, which had annualised per cent change values that exceeded the scale of this figure by orders of magnitude. (A) Change over 1990–2007. (B) Change over 2007–2019. YLDs, years lived with disability.

GBD 2019 estimated the incidence and prevalence of a range of disease, injuries, and sequelae, summarised here in YLDs. Population growth and ageing were key drivers of nonfatal disease burden: the total number of YLDs nationwide increased from 3.59 (95% UI 2.67–4.61) million in 1990 to 6.50 (4.86–8.37) million in 2019, while age-standardised YLD rates increased from 11 587 (8677–14 796) per 100 000 in 1990 to 12 053 YLDs (9046–15 474) per 100 000 in 2019. Trends in YLDs by province for specific causes of disability are illustrated in figure 3. Aside from YLDs due to HIV/AIDS and its sequelae (including tuberculosis), which increased several orders of magnitude, the largest increases among leading causes of YLDs were from diabetes, chronic kidney disease, neonatal disorders and other musculoskeletal disorders.

All provinces except for North-West experienced a considerable increase in YLDs from NCDs between 2007 and 2019 from the combined effects of demographic change and rising age-specific prevalence of NCDs such as diabetes and chronic kidney disease. The largest increase in YLDs from NCDs was in Northern Cape at 3.8% (+3.5 to +4.0%) per year, while YLDs in North-West decreased at −0.6% (−0.4% to −0.8%). Due to their high prevalence, mental disorders as a group contributed to the largest total number of YLDs in all provinces, though their rate of increase between 1990 and 2019 was less pronounced than for other NCDs. Injuries accounted for about 4.6% of total YLDs in Limpopo and Northern Cape (the highest two provinces), as compared with 3.2% and 3.7% of total YLDs in KwaZulu-Natal and Mpumalanga (the lowest two provinces).

### Summary measures of health

There were an estimated 18.4 million (95% UI 17.0–19.9) DALYs in South Africa in 1990, 38.5 million (35.1–42.6) DALYs in 2007 and 26.6 million (24.4–29.1) DALYs in 2019. The age-standardised DALY rate increased from 53 698 per 100 000 (48 888–57 810) in 1990 to 83 870 per 100 000 (77 045–92191) in 2007, then declined to 49 954 per 100 000 (45 909–54 309) in 2019. Age-standardised DALY rates in 2019 ranged from 40 387 (36 054–46 143) per 100 000 in the Western Cape to 61 124 (54 801–67 672) per 100 000 in Free State (online supplemental table S4). The composition of DALYs also varied by province (online supplemental figure S4). Aside from HIV/AIDS and sexually transmitted diseases, the provinces with the highest proportion of DALYs from communicable, maternal, neonatal, and nutritional diseases were Limpopo, North-West, Northern Cape and Eastern Cape. The provinces with the highest proportion of DALYs from NCDs were Western Cape and Northern Cape.

Violence and unintentional injuries—a less-emphasised component of the ‘quadruple burden of disease’^18^—comprised 3.9% (2.6%–4.8%) (Mpumalanga) to 8.5% (7.2%–9.7%) (Northern Cape) of DALYs in 2019, driven by YLLs. However, this proportion has been declining since 1990, from 11.1% (10.0%–12.0%) of total DALYs to 6.3% (5.4%–7.2%) nationwide by 2019. The proportion of DALYs due to injuries was highest in Northern Cape, Eastern Cape and Free State for much of the 1990–2019 period.

HALE declined across all provinces between 1990 and 2007 and recovered between 2007 and 2019; however, only Gauteng and Western Cape had a longer HALE for both sexes in 2019 compared with 1990 (table 1). The increase in HALE from 2007 to 2019 was particularly dramatic in Free State (+12.3), KwaZulu-Natal (+12.3) and Eastern Cape (+11.6). The exception to this trend was the Western Cape, where HALE slowly increased for both males and females from 1990 to 2019 and increased slightly over the period 1990 to 2007, in contrast to all other provinces.

**Table 1:** Trends in healthy life expectancy (in years) by sex, province and year

	1990			2007			2019			Delta HALE (1990 vs 2019)	
	Male	Female	Both sexes	Male	Female	Both sexes	Male	Female	Both sexes	Male	Female
South Africa	52.8 (50.8 to 54.8)	58.1 (55.2 to 60.7)	55.5 (53.0 to 60.7)	45.5 (43.5 to 47.4)	47.1 (44.2 to 49.7)	46.4 (44.1 to 48.5)	54.4 (52 to 56.8)	57.9 (54.8 to 60.7)	56.2 (53.6 to 58.4)	1.6	−0.3
Eastern Cape	52.4 (49.7 to 55)	57.1 (53.9 to 60.1)	54.9 (52.2 to 57.5)	41.9 (39.9 to 44)	45.4 (42.5 to 48.1)	43.7 (41.4 to 46.0)	51.4 (48.7 to 54.1)	57 (53.7 to 60.3)	54.3 (51.5 to 57.0)	-1	−0.1
Free State	50.7 (48.1 to 53)	56.1 (53 to 59.3)	53.2 (50.5 to 55.5)	39.6 (37.9 to 41.2)	40.8 (38.3 to 43)	40.2 (38.2 to 42.0)	50.5 (47.9 to 53)	54.6 (51.4 to 57.7)	52.5 (49.8 to 55.1)	−0.2	−1.4
Gauteng	52.7 (50.2 to 55)	57.6 (54.7 to 60.5)	54.9 (52.3 to 57.3)	49.9 (47.6 to 52.3)	48.8 (45.6 to 51.8)	49.4 (46.9 to 51.8)	57.3 (54.4 to 60.1)	58.7 (55.1 to 61.8)	58.0 (55.2 to 60.7)	4.6	1.1
KwaZulu-Natal	50.5 (48 to 53.1)	56.9 (53.4 to 60)	53.7 (51.1 to 56.2)	41.1 (38.9 to 43)	42.7 (39.7 to 45.4)	41.9 (39.4 to 44.1)	52.1 (49.3 to 55)	56.1 (52.6 to 59.3)	54.2 (51.4 to 57.0)	1.6	−0.8
Limpopo	60 (57 to 63.1)	61.4 (57.7 to 64.8)	60.8 (57.8 to 63.8)	47.4 (45.2 to 49.4)	51.2 (48.1 to 54.1)	49.4 (47.0 to 51.9)	54.9 (52.3 to 57.5)	59.9 (56.4 to 63.2)	57.6 (54.5 to 60.3)	−5.1	−1.5
Mpumalanga	56.3 (53.6 to 59.1)	59.5 (56 to 62.6)	57.9 (55.0 to 60.7)	43.7 (41.3 to 45.7)	44 (40.7 to 46.7)	43.8 (41.1 to 46.1)	54.1 (50.8 to 57.2)	56.7 (53 to 60.1)	55.4 (52.5 to 58.4)	−2.3	−2.8
North-West	56.9 (53.9 to 59.7)	60 (56.8 to 63.3)	58.4 (55.4 to 61.1)	44.8 (42.3 to 47.4)	44.8 (41.5 to 47.9)	44.8 (42.2 to 47.3)	52.5 (49.5 to 55.6)	55.2 (51.7 to 58.9)	53.7 (51.0 to 56.7)	−4.4	−4.8
Northern Cape	54.8 (52.1 to 57.4)	56.9 (53.8 to 59.9)	55.8 (53.1 to 58.4)	44.2 (42.5 to 45.9)	46.3 (43.9 to 48.5)	45.2 (43.3 to 47.1)	52.9 (50.5 to 55.4)	56.5 (53.2 to 59.5)	54.7 (52.0 to 57.2)	−1.8	−0.4
Western Cape	53.6 (51.2 to 56)	58.6 (55.7 to 61.4)	56.0 (53.5 to 58.4)	55.3 (53.2 to 57.3)	58.4 (55.4 to 61.2)	56.9 (54.4 to 59.2)	58.5 (55.5 to 61)	61.1 (57.5 to 64.4)	59.8 (56.7 to 62.5)	4.9	2.5

HALE, healthy life expectancy.

### Risk-attributable disease burden

**Figure 4:**
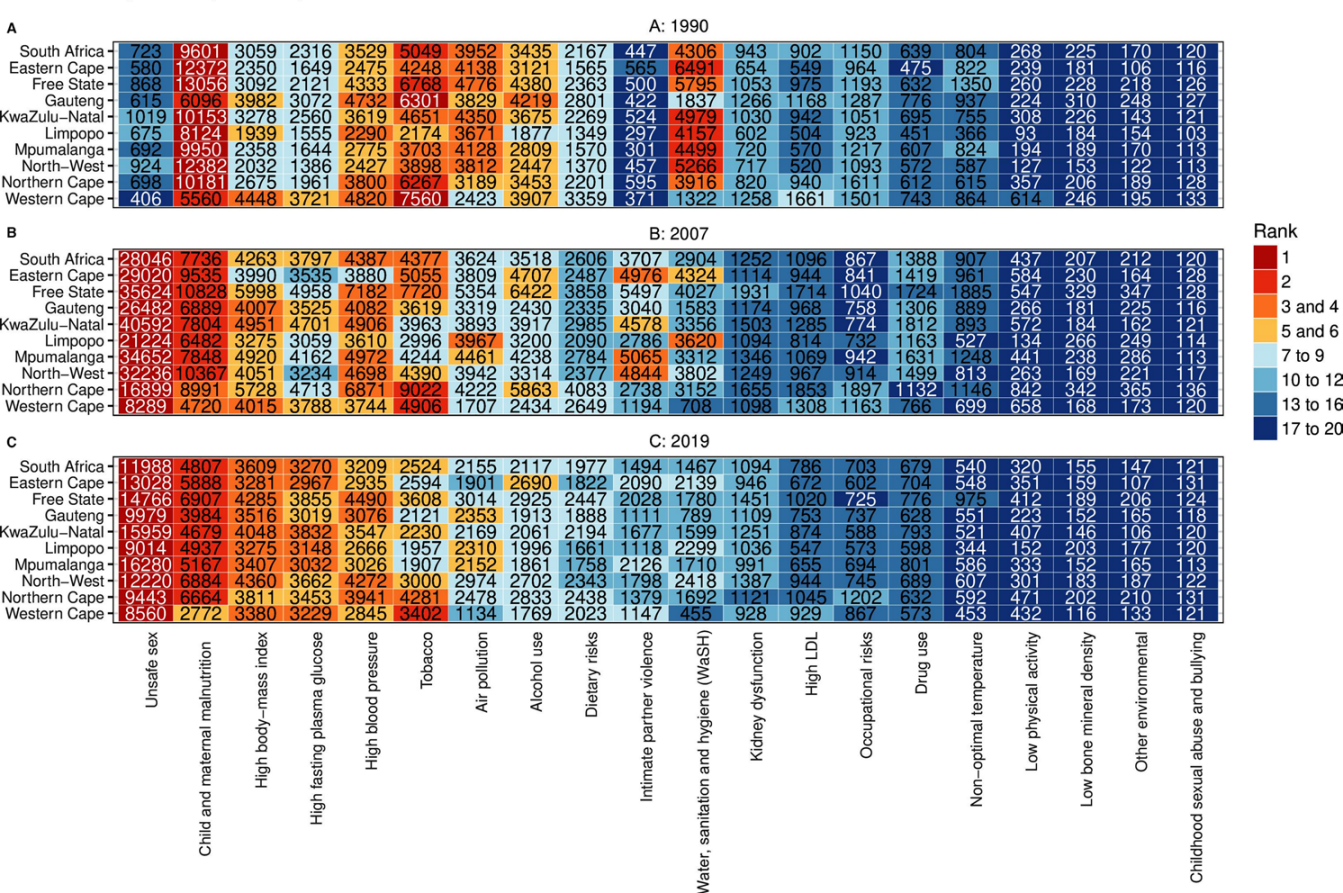
Heatmap showing top causes of age-standardised, risk-attributable DALYs per 100 000 population (both sexes) by province. (A) 1990. (B) 2007. (C) 2019. DALYs, disability-adjusted life-years. LDL-low-density lipoproteins.

Between 1990 and 2019, important shifts occurred in the top risk factors for health loss, both at the national and provincial level (figure 4). In 1990, the top five risk factors generally included child and maternal malnutrition, alcohol use, smoking, and unsafe sanitation, though the Western Cape and Gauteng to a lesser extent were notable for a predominance of NCD risk factors (figure 4A).

By 2019, the risk factors that had ranked in the top five in 1990 had dramatically fallen in importance in all provinces, and NCD risk factors predominated (figure 4C). The exception was unsafe sex, which was the top risk factor in all provinces and to which HIV/AIDS morbidity and mortality were attributed. Most risk-attributable DALYs were due to HIV/AIDS, diabetes and chronic kidney diseases, cardiovascular diseases, respiratory infection and tuberculosis, maternal and neonatal disorders, and self-harm and interpersonal violence.

### Healthcare system performance

**Figure 5:**
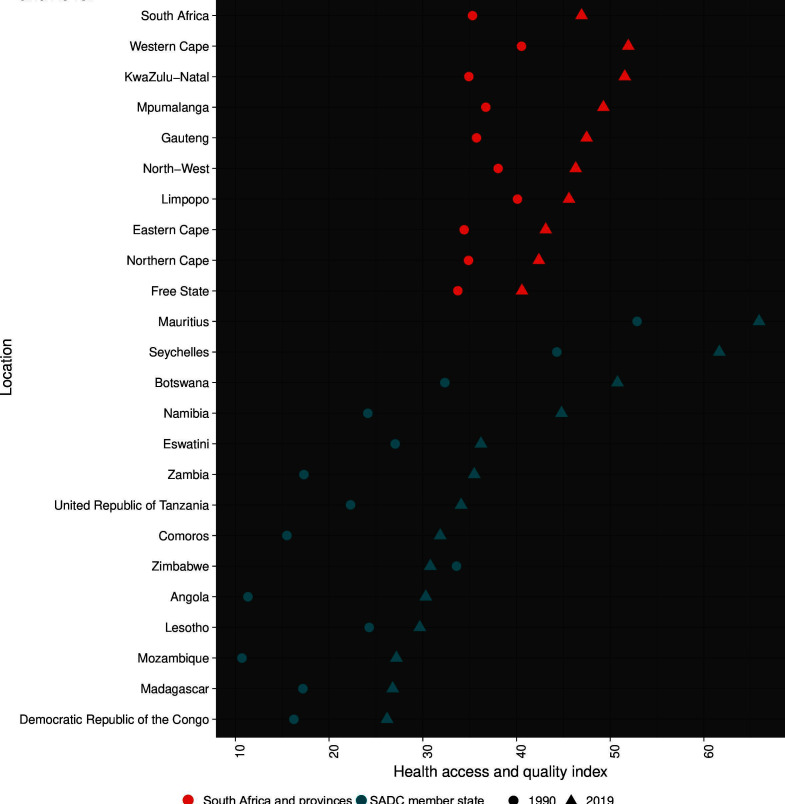
GBD healthcare access and quality index values for South Africa and provinces compared with Southern African Development Community member states, 1990 and 2019. The GBD healthcare access and quality index is a summary measure of health system performance that incorporates estimates of age-standardised and risk-standardised mortality rates for 32 causes that are amenable to healthcare. The index scale ranges from 0 to 100, with higher values indicating better performance. GBD, Global Burden of Disease; SADC, Southern African Development Community.

We compared HAQ Index values for the year 1990 and 2019 across all nine provinces and with the other 15 SADC member countries (figure 5; online supplemental figure S5). In terms of overall performance, South Africa ranked third in 1990 and fourth in 2019, surpassed only by Mauritius and Seychelles in both years. However, HAQ Index values varied considerably between provinces, with the highest values in 2019 for Western Cape and KwaZulu-Natal (similar to Mauritius in 1990), and the lowest values for Free State, Northern Cape, and Eastern Cape (between Eswatini and Namibia in 2019). These three provinces also had the smallest improvements over time. By contrast, the largest improvements in HAQ Index were in KwaZulu-Natal, Mpumalanga and Gauteng; the only SADC neighbours that had a larger improvement than KwaZulu-Natal were Seychelles, Zambia, Botswana, Angola and Namibia.

**Figure 6:**
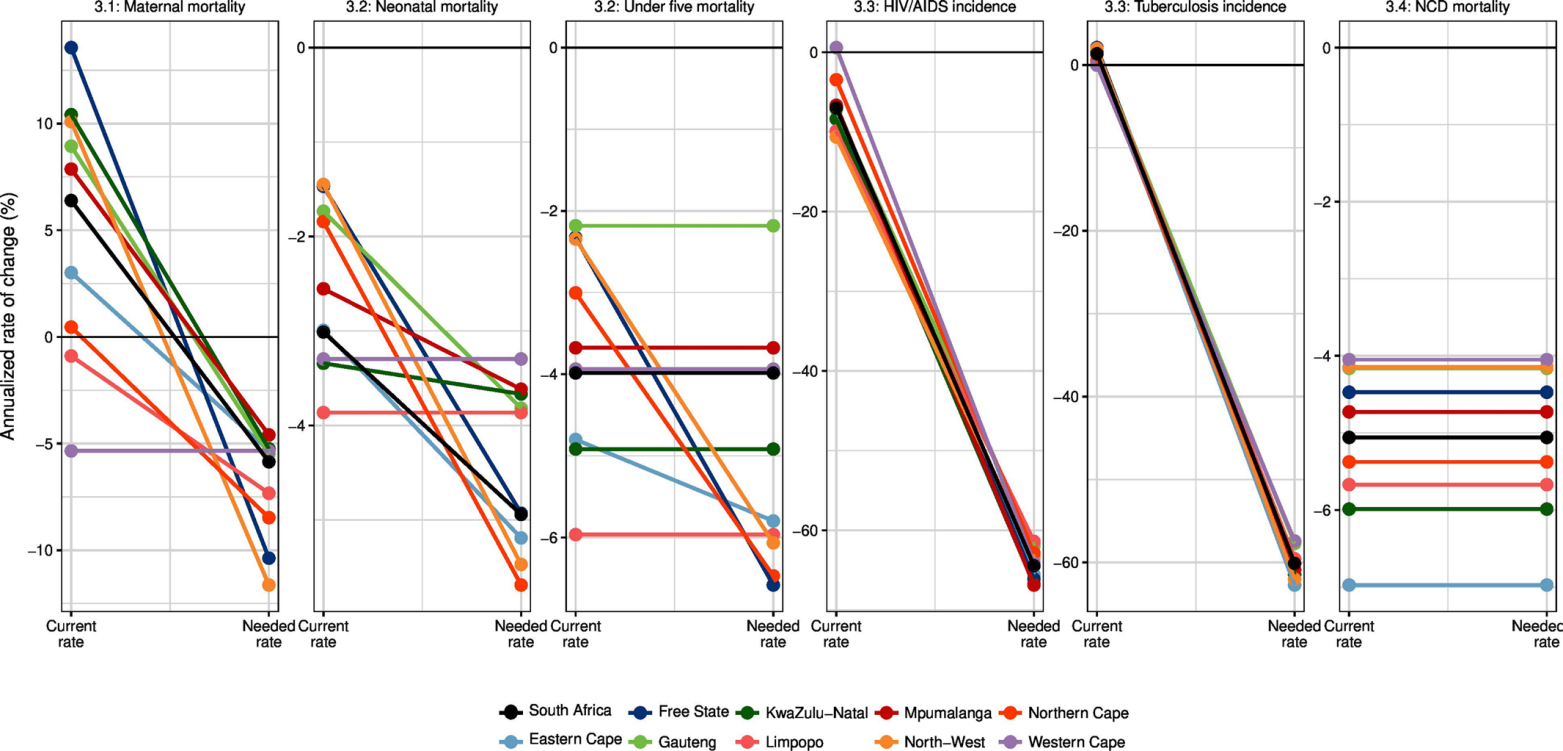
Progress towards achievement of key health-related sustainable development goal targets in South Africa and provinces. In each plot, the left hand y axis shows the observed annualised rate of change in the indicator over 2015–2019, the start of the SDG period. The right hand y axis shows the annualised rate of change in the indicator that would be required to achieve SDG target by 2030. Locations whose rates of change are on track or exceeding needed rates of change are shown as horizontal lines; locations whose rates of change are off track are shown as diagonal lines, with the slope of the line indicating the extent to which the location is off track (steeper slopes indicate locations that are further behind). NCD, noncommunicable disease

Figure 6 plots the observed average ARC (2015–2019) in key quantitative SDG-3 targets by province and compares these to the rate of change that is needed to achieve the target. We estimate that all nine provinces are currently on track to achieve the NCD target, and five are on track to achieve the U5MR target. Two provinces at most are currently on track to achieve neonatal mortality and MMR targets, respectively. HIV incidence declined quite slowly over 2015–2019, and tuberculosis incidence increased, suggesting these targets will be very challenging to achieve.

## Discussion

This analysis adds to the literature on mortality in South Africa and represents the largest systematic effort to date to quantify levels and long-term trends in nonfatal outcomes, risk-attributable burden, and comparative healthcare system performance in South Africa and its provinces.^1 18^ As in other countries, a subnational lens on health progress is important in South Africa because of considerable health inequalities and decentralisation of health planning and policy. South Africa is committed to achieving the health-related SDGs. Achieving SDG targets necessitates monitoring of health outcomes and associated risks, as well as inequality in health infrastructure and inequitable access to services. One practical use of these subnational estimates is to identify which provinces are on track to achieve specific SDG targets to provide opportunities to share best practices and shore up disease-specific programmes in provinces that are off-track. We found that most health indicators varied greatly by province, with poorer and more rural provinces generally showing lower levels of health and less progress since 1990.

While all provinces have made progress on HAQ since 1990, economically advantaged provinces have generally progressed at a faster rate, and performance in disadvantaged provinces has lagged behind neighbouring middle-income SADC countries. Many of the inequalities within and across provinces in South Africa are also due to the fragmented nature of the health system, with an under-resourced public health sector serving most of the people and a well-resourced private sector serving a minority of the population. The NHI provides a vital opportunity to close these gaps by integrating the public and private sector financing. In addition, the COVID-19 pandemic has stressed a fragile healthcare system and has highlighted the ‘syndemic’ nature of South Africa’s quadruple burden of disease by exposing the dominant role of shared risk factors (like harmful alcohol use) and social-structural vulnerabilities in driving disease and injury trends. Further, significant contraction of the local and regional economies is expected to exacerbate inequities in access to healthcare for the most vulnerable.^19 20^ Together, our findings suggest that a doubling-down on investments in key health programmes is needed in nearly all provinces, especially the least advantaged—and these investments are particularly urgent in the wake of COVID-19-related health service disruptions.

Unsurprisingly, the impact of HIV/AIDS and concurrent tuberculosis still dominate much of the quadruple burden of disease in South Africa and remain major drivers of changes in population health. Our analysis confirms that the national response to HIV/AIDS after 2007, including the rollout of ART, significantly improved life expectancy thereafter.^21^ At the same time, we found an increase in the burden of HIV/AIDS and tuberculosis among adolescents, suggesting that more intensive, integrated efforts are needed for prevention and treatment in this age group.^22^ Given the rapid rises in NCDs and associated risk factors, integrated care for adolescents should also have a strong emphasis on NCD prevention.^23^ Furthermore, while significant progress has been made in controlling tuberculosis among people living with HIV, in part because of expanded ART, limited progress has been made in controlling tuberculosis in HIV-negative populations.^24^ This further supports the need for strategies targeting other non-HIV risk factors associated with tuberculosis that are increasingly prevalent, such as diabetes and alcohol abuse.^25 26^ The COVID-19 response in South Africa will need to ensure greater support for people living with HIV and that the HIV care continuum is not significantly disrupted and intensify efforts to prevent a resurgence in tuberculosis in 2021 and beyond.

Maternal and child survival has been an area of success since 1990, though most provinces do not appear to be on track to meet the SDG targets. To some extent, recent progress reflects the rollout of ART, including prevention of mother-to-child transmission, coupled with increased coverage of core Expanded Programme on Immunisation vaccines and, more recently, pneumococcal conjugate and rotavirus vaccines.^27 28^ Crucially, stay-at-home orders that have been implemented to limit the spread of COVID-19 could significantly disrupt immunisation activities, thus increasing the risk of children contracting other infectious diseases, as documented in Pakistan, for example.^19^ A recent modelling study found that the risk of COVID-19 death in the context of receiving immunisation services was far outweighed by the risk of death from a vaccine-preventable disease that resulted from not receiving these services.^29^ To prevent increases in child mortality from vaccine-preventable diseases, the Department of Health will need to assess where provincial and sub-provincial immunisation services have been most disrupted and redirect resources to targeted, mop-up campaigns.

Injuries, especially from interpersonal violence, have been a consistent feature of the South African landscape over the past 25 years. Evidence suggests that violence can increase during and in the aftermath of disease pandemics, as has been observed in the context of COVID-19-related lockdown/stay-at-home orders, suggesting a nuanced approach to COVID-19 mitigation will be required to balance direct and indirect health effects.^30^ The single most important risk factor for injuries in South Africa is a high prevalence of harmful use of alcohol. While WHO and others acknowledge alcohol taxes and regulations as ‘best buys’ in terms of their population health impact, practical challenges remain in implementing these policies in the South African context.^31 32^ Alcohol consumption remains a primary form of recreation for many and is strongly linked to perceptions of masculinity; a large local alcohol industry also ensures easy access. Past efforts to reduce alcohol and drug abuse locally have generally failed to combine interventions targeting high-risk persons and groups with population-wide approaches to reduce per-capita consumption.^33^ Further, alcohol policies may have limited impact without concurrent efforts to address firearm possession and use, or to de-link heavy drinking from gender norms. Integrated alcohol and firearm control policies, combined with sustained public campaigns addressing drinking culture, will be necessary to reduce interpersonal violence, and they could generate extensive social and economic benefits.^34^ However, there are no quick fixes to these inter-related risks; long-term investment and commitment are essential.

We document a rapid increase in nonfatal disease burden and NCD risk factors in all provinces, despite notable progress on reducing age-specific NCD death rates. These increases, driven by prevalent conditions like diabetes, have implications for health planning and resource allocation at the provincial level. Health promotion and prevention efforts are urgently needed in order to prevent a resurgence in NCD mortality and reduce fiscal pressures on NHI.^35^ Tobacco control was an early success story for South Africa, but more recently tobacco use as a risk factor has been replaced by obesity and physical inactivity, which have attributable DALY rates that are significantly higher than in other upper-middle-income countries like Brazil and China.^13 36^ Dietary policies (including taxes, subsidies and regulations) and efforts to promote exercise through the built environment will probably have an outsized role in the intersectoral agenda in the coming years. In addition, a critical gap in the South African health system is the lack of integrated care for mental disorders at the primary level, a highly-specific, evidence-based, best-buy intervention.^37^

Prior reports have summarised the major limitations of the GBD 2019 study in general.^12–14^ This study had specific limitations related to the lack of available local data for a number of important health areas (online supplemental table S2). While GBD attempts to address data gaps using a suite of statistical methods and processes, the accuracy of GBD estimates for South Africa will continue to improve as more local data become available. Previous GBD estimates for South Africa have been critiqued on the basis of their discordance with the estimates of other publications such as those of the BoDRU-SAMRC.^38^ GBD 2019 made substantial improvements in data processing and modelling accuracy as compared with previous GBD rounds. Online supplemental information section 3 and online supplemental table S5 outline these improvements and compare our methods and findings to those in recent BoDRU-SAMRC publications. Generally, GBD 2019 estimates for the year 2017 were concordant with SAMRC estimates for the same year across a range of mortality measures. The most notable exceptions were neonatal and infant mortality, for which GBD 2019 mean estimates were 83% and 43% higher, respectively.

## Conclusions

The GBD 2019 study provides new insights into differences in health risks and outcomes across South Africa’s nine provinces. Since the rollout of ART, progress on reducing health inequalities and improving equitable access to healthcare has been mixed: health indicators have improved in the provinces hardest-hit by the HIV/AIDS epidemic, but provinces that are less economically advantaged are being left behind and appear to be off track to achieve key health SDG targets. The ongoing COVID-19 pandemic threatens to further derail progress since 2007 but could provide new opportunities to reform provincial healthcare systems and tackle emerging risk factors. Our study facilitates benchmarking and promotion of exchange of best practices at the provincial level as the country strives to achieve better health for all.

**What this study adds**

- This is the first systematic subnational assessment of health progress in South Africa within the context of the Global Burden of Diseases, Injuries and Risk Factors (GBD) Study.
- We corroborate and expand on mortality estimates published by the South African Medical Research Council Burden of Disease Research Unit (SAMRC-BoDRU)
- We provide novel provincial-level estimates of trends in nonfatal outcomes, risk factor burden and progress towards key health-related SDG targets, highlighting the need for contextually tailored awareness and prevention policies that tackle risk factors and improve the integration of communicable and non-communicable disease care at the primary healthcare level.

**What is already known on this subject**

- An influential Lancet Series in 2009 on Health in South Africa provided recommendations for priority diseases, structural reforms in the areas of health financing, disease surveillance and research and innovation.
- South Africa’s health challenges were framed at the time as colliding infectious disease, non-communicable disease and injury epidemics.
- National burden of disease studies conducted by SAMRC-BoDRU have documented national and provincial trends in mortality for 140 causes, with the most recent publication providing estimates for 2017, revealing that despite steady progress, there are still persistent and emerging health challenges that need policy attention to sustain gains.

## Data Availability

Data are available in a public, open access repository. This paper summarises key findings from our analysis of GBD 2019 estimates. Data files containing all GBD 2019 subnational estimates are available on the GHDx (http://ghdx.healthdata.org/gbd-2019). Additional results can be explored through online interactive data visualisations (http://www.healthdata.org/gbd/data-visualizations).
