# Supplementary material for: Health trends, inequalities and opportunities in South Africa’s provinces, 1990–2019: findings from the Global Burden of Disease 2019 Study
Source: J Epidemiol Community Health. 2022 Jan 19;76(5):471–81. doi: 10.1136/jech-2021-217480 (PMC8995905; doi:10.1136/jech-2021-217480)
Supplement: Supplementary data [file jech-2021-217480supp001.pdf]

Supplementary appendix to "Health Trends, Inequalities, and Opportunities in South Africa's Provinces, 1990-2019: Findings from the Global Burden of Disease 2019 Study"

This appendix provides supplemental figures and more detailed results for "Health Trends, Inequalities, and Opportunities in South Africa's Provinces, 1990-2019: Findings from the Global Burden of Disease 2019 Study."

Portions of this appendix have been reproduced or adapted from Vos et al.[1], Roth et al.[2], James et al.[3], and Kyu et al[4]. References are provided for reproduced sections.

Table of Contents

List of figures and tables 2
Figures 2
Tables 2
Section 1: Statement of GATHER compliance 3
Section 2: GBD Methods Overview 3
Section 2.1: GBD cause list[1] 3
Section 2.2: GBD 2019 Causes of Death database[1] 4
Section 2.3: Causes of death modelling methods 24
Section 2.4: Central computation[2] 29
Section 2.5: Non-fatal outcome estimation[3] 30
Section 2.6: Estimation Process for DALYs[4] 60
Section 2.7: SDI Analysis[4] 61
Section 3: Burden of Disease Estimation for South Africa 64
Section 4: Author Contributions 66
Section 5: References 67
Section 6: Figures and Tables 70
Figure S1: Absolute change in mortality by province, 1990-2007 versus 2007-2019. Panel A: all-cause mortality. Panel B: HIV/AIDS 70
Figure S2: Under-5 mortality rates per 1000 live births (5q0), both sexes 71
Figure S3: Maternal mortality rates per 100 000 live births 72
Figure S4: Decomposition of DALYs into causes of YLLs and YLDs by province. Panel A: 1990. Panel B: 2007. 73
Figure S5: Health access and quality (HAQ) and life expectancy at birth, 1990-2019, South Africa, provinces, andSADC member states 74
Figure S6: Contribution of various causes of death to changes in life expectancy for males and females by province. Panel A: 1990-2007. Panel B: 1990-2019.Panel C: 2007-2019. 75
Table S1: GATHER checklist of information that should be included in reports of global health estimates, with description of compliance and location information for "Health trends, inequalities, and opportunities in South Africa's provinces, 1990—2019: Findings from the Global Burden of Disease 2019 Study." 82
Table S2: GBD 2019 South Africa subnational data sources 84
Table S3: Mortality per 100,000 for all-cause and HIV/AIDS in South Africa, 1990 and 2019 147
Table S4: All-cause age-standardised DALY rates, 1990—2019 for South Africa and provinces 149
Table S5: Comparison of burden of disease estimates for South Africa 151
Table S6: List of International Classification of Diseases (ICD) codes mapped to the Global Burden of Diseasecause list for causes of death 152

List of figures and tables

Figures and tables are available in section 6.

Figures

Figure S1. Absolute change in mortality by province, 1990-2007 versus 2007-2019.

Figure S2. Under-5 mortality rates per 1000 live births (5q0), both sexes.

Figure S3. Maternal mortality rates per 100 000 live births

Figure S4. Decomposition of DALYs into causes of YLLs and YLDs by province

Figure S5. Health access and quality (HAQ) and life expectancy at birth, 1990-2019, South Africa, provinces, and SADC member states

Figure S6: Contribution of various causes of death to changes in life expectancy for males and females by province. Panel A: 1990-2007. Panel B: 1990-2019. Panel C: 2007-2019.

Tables

Table S1. GATHER checklist of information that should be included in reports of global health estimates, with description of compliance and location information for “Health trends, inequalities, and opportunities in South Africa’s provinces, 1990—2019: Findings from the Global Burden of Disease 2019 Study”

Table S2. GBD 2019 South Africa subnational data sources

Table S3. Mortality per 100,000 for all-cause and HIV/AIDS in South Africa, 1990 and 2019

Table S4. All-cause, age-standardised DALY rates, 199-2019 for South Africa and provinces

Table S5. Comparison of burden of disease estimates for South Africa, SAMRC estimates and GBD 2019 estimates for the year 2017

Table S6. List of International Classification of Diseases (ICD) codes mapped to the Global Burden of Disease cause list for causes of death

Section 1: Statement of GATHER compliance

This study complies with the Guidelines for Accurate and Transparent Health Estimates Reporting (GATHER) recommendations.[5] See table S1 for the GATHER checklist. The GATHER recommendations can be found at the GATHER website under GATHER Statement.

Section 2: GBD Methods Overview

Section 2.1: GBD cause list[1]

The GBD cause and sequelae list is organized hierarchically (see Appendix Table 2) to accommodate different purposes and needs of various users.

The first two levels aggregate causes into general groupings. At Level 1 there are three cause groups: communicable, maternal, neonatal, and nutritional diseases (Group 1 diseases); non-communicable diseases (Group 2); and injuries (Group 3). These Level 1 aggregates are subdivided at Level 2 of the hierarchy into 22 cause groupings (e.g., neonatal disorders, neurological disorders, and transport injuries). The disaggregation into Levels 3 and 4 contains the finest level of detail for causes captured in GBD 2019. The greatest detail available for some causes, such as anxiety disorders or rheumatoid arthritis, is at Level 3 of the hierarchy, while other specific causes are at Level 4 of the hierarchy with an aggregate category at Level 3 (for example, depressive disorders at Level 3, which encompasses major depressive disorders and dysthymia at Level 4). Sequelae of diseases and injuries are organized at Levels 5 and 6 of the hierarchy. In GBD, sequelae are defined as distinct, mutually exclusive categories of health consequences that can be directly attributed to a cause. For example, both neuropathy and blindness due to diabetic retinopathy are sequelae of diabetes; stroke and ischaemic heart disease are not, as these consequences cannot be categorically ascribed to diabetes in an individual despite good evidence for increased risk of these outcomes. The finest detail for all sequelae estimated in GBD is at Level 6 and is aggregated into summary sequelae categories (Level 5) for causes with large numbers of sequelae. Examples include the grouping of the infectious disease episodes and long-term sequelae of meningitis. For GBD 2019 there are 3473 mutually exclusive and collectively exhaustive sequela, 2063 cause sequelae and 1410 injuries sequelae, and thus our YLD estimates at each level of the hierarchy sum to the total of the level above. Prevalence and incidence aggregation is estimated at the level of individuals who may have more than one sequela or disease and therefore are not additive.

The GBD cause list continues to evolve to reflect the policy relevance, and public health and medical care importance of the causes of major losses of health. The cause and sequelae list expanded based on input from the Scientific Council and GBD collaborator network. For GBD 2019 the causes of death cause list has increased to 286 causes, from the 282 causes in GBD 2017. The non-fatal cause list has expanded from 354 causes in GBD 2017 to 364 causes in GBD 2019. The total number of fatal and non-fatal causes combined for GBD 2019 is 369. As in GBD 2017, we made no estimates for YLDs for

Achoki T, *et al. J Epidemiol Community Health* 2022; 76:471–481. doi: 10.1136/jech-2021-217480

just five causes, either because no disability is possible (as is the case with sudden infant death syndrome); because disability may occur rarely but at levels too low for accurate estimation given the data (as for aortic aneurysm); or because the disability is captured by the complicating causes that led to that cause of death (as for indirect maternal deaths, late maternal deaths, and maternal deaths aggravated by HIV/AIDS).

Section 2.2: GBD 2019 Causes of Death database[1]

Background

All available data on causes of death (CoD) data are standardized and pooled into a single database used to generate cause-specific mortality estimates by age, sex, year, and geography. Appendix Figures 1 and 2 show the high-level view of data inputs, analytical steps, and outputs of the causes of death (CoD) analysis frame. Section 2 of this appendix provides details on each step in the development of the CoD database as illustrated in Appendix Figure 1.

CoD data identification[2]

Overview of data types

The CoD database contains seven types of data sources (Appendix Table 3): vital registration (VR), verbal autopsy (VA), cancer registry, police records, sibling history, surveillance, survey/census, and minimally invasive tissue sample (MITS) diagnoses. In countries with complete VR systems, there is no need to use any other data source. Less than half the world's population has deaths captured in a VR system, therefore, for countries with incomplete VR systems, vital statistics for causes of death may be supplemented with other data types (Appendix Figure 3).

ICD-detail

A majority of the CoD data is VR data obtained from the World Health Organization (WHO) Mortality Database, a compilation of data submitted to the WHO by individual countries. VR is also obtained from country-specific mortality databases operated by official offices. Each cause is coded directly to the most detailed CoD when possible, whereas cause codes in data tabulated by International Classification of Disease (ICD-) are coded to aggregated cause groups. The CoD database contains 2,525 country-years of detailed data from 1980 to 2018, which includes underlying CoD coded with 3-5 digit codes, by country, year, sex, and age groups. Detailed causes are coded to one of the following ICD-detail coding systems: ICD-8, ICD-9, or ICD-10 (Appendix Table 4). Each coding system has a similar cause hierarchy and cause list that has continually developed over time. ICD-10 is the current standard and the most exhaustive cause list. Within the cause lists, 5-digit codes are truncated to 4-digit codes to condense the lists. Updates to ICD-detail occur biannually as WHO releases new versions or as country collaborators provide additional data. Updates to data from WHO increasingly include ICD-10 CoD data as it is the most current classification of CoD, while updates to ICD-8 and ICD-9 detailed lists are less common. In the case of overlapping data, preference is given to data from pre-determined country collaborations, which are updated annually.

ICD-tabulations list

The ICD-tabulation lists include the ICD-8 List A (ICD-8A), ICD-9 Basic Tabulation List (BTL), ICD-10 Mortality Tabulation, Russia Tabulation, and India Medical Certification of Cause of Death (MCCD). These data sources make up 1,096 country-years from 1980 to 2016 in the CoD database. All are condensed versions of the ICD-8, ICD-9 and ICD-10 detail lists with some differences in the format of cause lists depending on the data source. ICD-8A, ICD-9 BTL, and ICD-10 Mortality Tabulation CoD are assigned to subtotal groups (referred to as chapters) and cause groups respective to ICD-detail groups. Additionally, ICD-9 BTL includes ICD-9 detail codes for some cancers and a custom tabulation scheme for the former Union of Soviet Socialist Republics (USSR) countries. The Russia Tabulation lists and India MCCD cause lists each have custom nomenclatures based on ICD-detail cause codes.
Two of the drawbacks in using tabulation lists are discrepancies in the accuracy of death counts and lack of detail due to aggregated cause groups. There are instances where the sum of deaths in chapter subtotals are not equal to the sum of cause groups within the chapter. To account for any missing or duplicate deaths reported within the cause groupings, death counts are systematically adjusted by calculating the differences between subtotals and sub-causes within the cause groups. Any differences are assigned to a remainder cause group. To account for the lack of cause code detail, select cause groups are disaggregated (Step 1.1) to create a complete cause list. Updates to ICD-Tabulation lists obtained from WHO occur less frequently compared to ICD-detailed lists as more countries are reporting deaths in ICD-detail. In instances of overlapping

data, preference is given first to detailed collaborator data, followed by detailed WHO data, then tabulated collaborator data, and finally tabulated WHO data.

Standardise input data (step 1)

The input data to the CoD database are received in various formats and must be standardised to run through central CoD machinery to then upload to the database. Raw data inputs come from data sources such as mortality databases, literature reviews, or reports. Usable data sources must have a clear sample size of the number of deaths in the population and exhaustive cause lists. The complexity of the data cleaning process varies drastically across data sources. For VR microdata with the location, age, sex, year, and ICD--coded cause of every death, very little effort is necessary to standardise it into a consistent structure. Other sources may require weeks of careful review to accurately extract scans of hardcover CoD reports into spreadsheets that can be transformed and standardised.

At this point, data are assigned source identifiers so that they can be linked to the GHDx and cited appropriately. Any aggregate age and sex categories are flagged for age-sex splitting. The methods of cause-of-death assignment and data collection are reviewed to determine which source type to assign; for example, we distinguish sibling history data from surveys with a VA module. Only data at the most detailed level of the GBD location hierarchy are used. Documentation from the source is reviewed to determine if the population is representative of the location or only a subset of the population in that location. Data sources representing a subset of the population are flagged as non-representative; this flag is used by Cause of Death Ensemble modelling (CODEm) to increase the variance associated with such data points.
Finally, diagnostics are reviewed at this stage to avoid sending cleaning errors downstream. We review cause-specific deaths for each demographic group to ensure the data are reasonable. For example, it is unlikely that male breast cancer deaths are higher than female breast cancer deaths or deaths from neonatal causes occur in age groups over one year. All death totals are compared with the sum of cause-specific deaths to ensure the observed deaths are accounted for and sample size is complete.

Disaggregation (step 1.1)

CoD in tabulated VR data are condensed into aggregated groups, some of which can be mapped directly to GBD causes, while other aggregated cause groups are not informative and cannot be mapped to them. To correct for this, aggregated causes were mapped and split onto multiple ICD-8, ICD-9, and ICD-10 detail causes, or targets, based on the ICD-groupings within the aggregated causes. ICD-8, ICD-9, and ICD-10 detail codes serve as targets because they are the highest-quality VR data and enable the calculation of proportions used to split the aggregated cause data into detailed causes. The proportions of deaths from nearby countries within the super-region were used to fill in data gaps as they were likely to have similar CoD trends.

We determined the targets based on detail causes missing from the tabulated cause list. For example, in ICD-9 BTL, the tabulated cause list includes a viral diseases group. In the hierarchy of causes, this group is comprised of “measles”, “yellow fever”, “encephalitis”, “hepatitis”, “rabies”, “other infectious diseases”, “garbage code”, and “remainder of viral diseases”. We did not consider this list to be an exhaustive list of viral diseases based on the range of ICD-detail codes given in the ICD-9 BTL documentation. To make the cause list exhaustive and inclusive of other viral diseases, we split the remainder of the viral diseases group into “other meningitis”, “other infectious diseases”, “herpes”, “dengue”, “other neglected tropical diseases”, and “garbage code”. After a list of targets was determined, the aggregated deaths were disaggregated to the target causes by using ICD-8, ICD-9, and ICD-10 detail proportions generated at the super-region level for the corresponding sex and age groups across all years in the time series. For example, in ICD-9 detail data, 54.8% of deaths in males in Latin America and the Caribbean within the target group for the BTL “remainder of viral diseases” group were designated to “other meningitis.” Thus, 54.8% of deaths in the tabulated group “remainder of viral diseases” were assigned to “other meningitis” for any country within that particular super-region. For any cause and demographic group for which we lacked ICD-detail, global proportions were used.

Calculate non-maternal deaths (step 1.3)

In cases when maternal mortality metrics do not include both deaths due to maternal causes and deaths due to non-maternal causes for women of reproductive age, live births and all-cause mortality estimates can be used to calculate deaths. Many studies report maternal deaths as the MMR. MMR is the number of maternal deaths per 100,000 live births and can be used to calculate deaths when it has been derived from primary data and not estimated. Maternal deaths were calculated by using MMR and live births; if live births were missing we substituted live birth estimates and used the following equation:

$$Maternal\ deaths = \frac{MMR}{100{,}000} \times Live\ births$$

If a study was non-representative, we extracted sample size and live births from that study. After maternal deaths were calculated, we used the difference from all-cause mortality estimates to determine non-maternal deaths.
A more accurate and data-inclusive method of calculating maternal and non-maternal deaths incorporates coverage and splits deaths for a range of years into individual years. If there were live births in the study, we adjusted the coverage.

$$Coverage = \frac{Live\ births}{GBD\ estimated\ live\ births}$$

After coverage was calculated, totals deaths were scaled to be more representative. This gives a more accurate death count since the envelope assumes representative coverage. We then calculated non-maternal deaths by using all-cause mortality as an all-cause total.

$$Maternal\ envelope\ with\ coverage = Maternal\ envelope \times Coverage$$

An additional adjustment can be applied to maternal data spanning over a range of consecutive years, which allows for more data inclusion. The years within specified year ranges are separated into individual years, and total deaths within the year range were split between each individual year by using the fixed proportions of maternal deaths from VR in that particular country. We used only VR data to inform the proportions because it was both high-quality and representative.

Map to GBD cause list (step 2)

In GBD 2019, we used 439 maps to translate causes found in the input data to the GBD 2019 cause list. This included 31 maps for VR data, 314 for VA data sources, and 98 for other data types. The largest, and most universal, maps used were those for ICD-9 and ICD-10 VR data. The input data causes varied from 3-4 digit ICD-codes to custom cause lists with cause names such as “cholera” or “hepatitis”. Our mapping process enabled us to compare these various data sources across demographic groups. (Appendix Table 4)

In GBD 2019, we developed additional maps to translate ICD-codes found in the input data that are non-underlying causes to appropriate target codes based on the levels of the GBD cause list. These garbage codes were mapped to Levels 1-4 of the GBD cause list according to the following criteria:

1. **Level 1** includes all garbage codes for which a Level 1 GBD cause cannot be directly assigned. For example, the underlying causes of “sepsis” or “peritonitis”, if not specified in the data, could be an injury, a non-communicable disease, or a type of communicable disease. In these cases, deaths will be redistributed across all three of these Level 1 causes. In addition, deaths coded to impossible or ill-defined causes of death (including “senility” and “unspecified causes”) fall into this category, as they will be redistributed onto all causes.

2. **Level 2** includes all garbage codes that can be assigned to Level 1 causes in the GBD cause list. This would include deaths coded to “unspecified injuries” (X59), which are redistributed onto all injuries.

3. **Level 3** includes all garbage codes for which we know the Level 2 CoD and can redistribute onto Level 3 causes. This includes deaths coded to causes such as “unspecified cardiovascular disease”, which falls within the Level 2 cause “cardiovascular diseases”, as well as those coded to “unspecified cancer site”, which falls within the Level 2 cause “neoplasms”.

4. **Level 4** includes all garbage codes for underlying causes of death that can be redistributed within a Level 3 cause. This includes garbage codes such as “unspecified stroke” or “unspecified road injuries.”

Figure S1: Age Standardized Fraction level 1,2 and level 3,4 of Garbage Codes for South Africa by provinces for 2010 and 2015 both sexes all ages

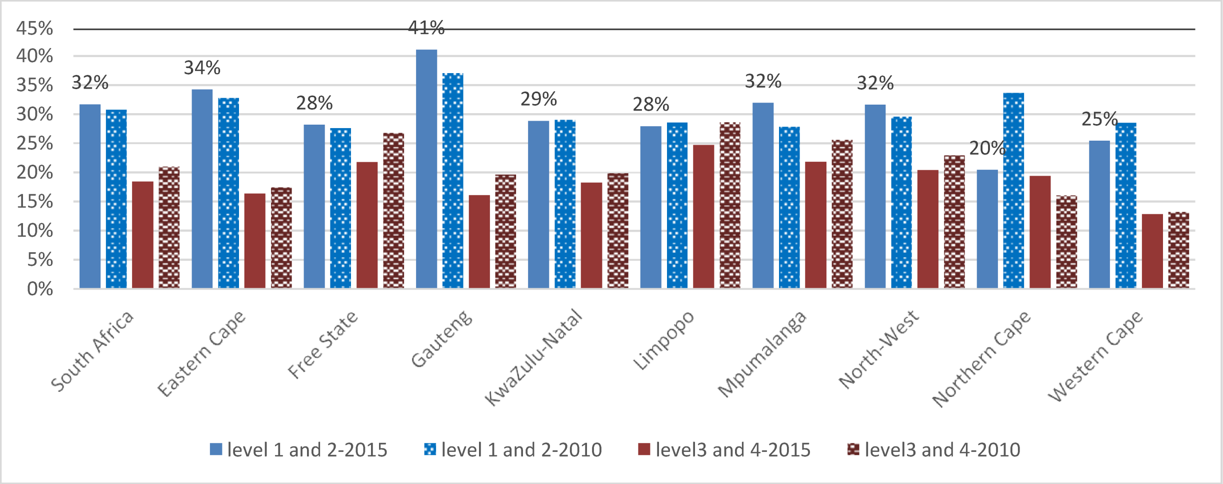

Age-sex splitting (step 3)

Different sources, particularly VA studies, report deaths for a wide range of age groups with varying intervals. For the analysis of CoD, we mapped these different age intervals to the GBD standard set of age groups. The approach to undertake this mapping was the same as in the prior GBD studies (GBD 2017, GBD 2016, GBD 2015, GBD 2013, and GBD 2010).

In the process of assembling a consolidated demographic database, we found that the aggregation of age groups is perhaps the strongest source of inconsistency. By convention, such data are reported in broad age groupings such as “0-4”, “5-14”, and “15-49,” or with both sexes together. The issue of comparability between age-sex groups arose when assembling the GBD CoD database. We developed a tool called age-sex splitting that takes aggregated age groupings and the “both sexes combined” grouping and divides them into what their constituent age groups would likely have been if respective cause-specific and country-specific age distributions had been used. The analytical framework for GBD includes three infant age categories: early neonatal (0-6 days), late neonatal (7-27 days), and post-neonatal (28-364 days), and 20 non-infant age categories: 1-4 years, 5-9 years, and so forth proceeding in five-year age groups until the terminal age group of 95+. We treat unknown ages and sexes in the same manner we treated the “all ages combined” age category and “both sexes combined” sex group. Through this process, we were able to directly compare all data sources on even terms.
The approach to age splitting is based on the following formula. The key assumption underlying this formula is that the relative risk of death by age group compared to a reference age group is invariant across populations. Although this assumption is likely violated in specific cases, a strong biologically based pattern of the relative risk of death for a cause by age is observed for most causes. The basic formula is as follows:

$$D_a = R_a N_a \left(\frac{D_a^{a+x}}{\sum_a^{a+x}(R_a N_a)}\right)$$

Where:

D_a= the number of deaths from a cause in age group a

R_a= global cause-specific mortality rate of age group a

N_a= the country-year-sex-specific population in age group a

D_a^{a+x}= the number of deaths in the age group a to $a+x$

With the assumption of invariant relative risks of death by age with respect to a reference age group, this equation can be used, along with population distribution by age, to split an aggregate number of deaths for the age groups a to $a+x$ into specific deaths for each age group within the aggregate interval.

$$D_{as} = R_{as} N_{as} \left(\frac{{}^{a+x,s}_{as}}{\sum_{a}^{a+x}(R_{as} N_{as})}\right)$$

Where:

D_{as}= the number of deaths from a cause in age group *a,* sex *s*

R_{as}= global cause-specific mortality rate of age group *a,* sex *s*

N_{as}= the country-year-sex-specific population in age group *a* for sex *s*

$D_{a,s}^{a+x,s}$= the number of deaths in the age group *a* to *a+x* for sex *s*

In some cases, deaths are reported for an aggregate age group for both sexes combined. The task in this case is more complicated, but the same principle can be applied. In this case we assumed that the relative risks of death by age and sex are constant.

This equation can be used to split data aggregated by age and sex. The assumption, however, of invariant relative risks across age and sex is a stronger assumption. Fortunately, data pooled across sexes are less common in the published or unpublished CoD data.

The relative risk of death in a particular age group for a given sex is derived from the global distribution of cause-specific mortality rates found in available VR data. Location-years from the following code systems are used, provided they report the requisite age- and sex-detail: ICD-7, ICD-8, ICD-9 BTL, ICD-10 tabulated, ICD-9, and ICD-10. Upon compiling these data, we mapped them to GBD causes and aggregated up to cause Level 3. This is the level at which a particular cause is split – that is, any child cause of a Level 3 parent is split by using the age distribution of that parent (so, chronic kidney disease due to diabetes would be split by using the age pattern of chronic kidney disease).

We next adjusted separately for estimated adult and child VR completeness. Location-year-age-sex-cause specific deaths and population were then aggregated across all location-years, to produce cause-specific mortality rates by age and sex. These were used to determine the risk of death at any age relative to any reference age group, as shown in the above equations.

Correct age-sex violations

Occasionally, data sources include deaths by a cause for which medical consensus exists that death is impossible for the sex and age. For example, some number of deaths may be attributed to cervical cancer in males, or to maternal causes in children younger than 10 years. We have constructed a conservative list of age-sex restrictions. When deaths violate these restrictions, we redistribute them proportionally onto all causes. All restrictions are included in Appendix Table 5, Restrictions on age and sex by cause for GBD 2019.

<u>Correction for miscoding of Alzheimer's and other dementias, Parkinson's disease, and atrial fibrillation and flutter (step 4)</u>

Objective

For certain CoD, mortality rates reported in VR systems are impossible to reconcile with observed trends in disease prevalence and excess mortality. For dementia, Parkinson's disease, and atrial fibrillation and flutter, these disparities can largely be attributed to death certification practices. We sought to address the known bias in CoD data by first identifying the proportion of all deaths that should be assigned to these causes and next determining the GBD causes and garbage groups to which these deaths are being incorrectly assigned.

In past GBD iterations, we estimated Alzheimer's disease and other dementias, Parkinson's disease, and atrial fibrillation and flutter on the basis of longitudinal prevalence and excess-mortality data to help account for changing patterns in death certification and corresponding implausible time trends in many VR sources. This method was first implemented for Alzheimer's disease and other dementias in GBD 2013. We added atrial fibrillation and flutter to the causes modelled in GBD 2015 and Parkinson's disease to the causes modelled in GBD 2016 by using this strategy. All of these causes were processed in CoDCorrect in a manner that was agnostic to the likely targets of misclassification, which inappropriately led to changes in mortality estimates for causes unrelated to these three in GBD 2015. For GBD 2016, we improved this

process by completing a literature review to identify the causes of death most closely associated with Parkinson's and Alzheimer's diseases[8–11] and limiting the CoDCorrect adjustments to include only those causes. For GBD 2017, we refined this approach further by using multiple CoD data to determine the GBD causes and garbage codes from which we move deaths as well as the pattern of misclassification.

Correction process

For Parkinson's disease and atrial fibrillation and flutter, we first estimated excess mortality from prevalence and CoD data in countries with the highest ratio of cause-specific mortality to prevalence, which represents the greatest willingness to code to an under-coded cause. Then, using DisMod-MR 2.1 (named from disease model Bayesian meta-regression), we derived estimates of cause-specific mortality rates from available prevalence surveys as well as the estimates of excess mortality rate, applied across all countries and over time. We divide this value by the all-cause mortality rate to determine the fraction of overall mortality to attribute to each under-coded cause. For dementia, the modeling process was redesigned in 2019 to no longer depend on vital registration data from the highest dementia mortality locations. Instead, we used relative risk data from cohort studies to calculate total number of excess deaths due to dementia, and end-stage disease proportions from linked hospital to death records to subset these deaths to the proportion of excess deaths with end-stage conditions, which we attributed to dementia. Finally, we used log-linear interpolation to interpolate final estimates of death due to dementia for the entire time series, and saved as a custom CoD model.

To ascertain the causes from which we would move deaths to under-coded causes, we leveraged multiple CoD data from the United States – by looking to the combinations of intermediate and immediate causes (i.e., chain causes) present on death certificates with an under-coded cause listed as underlying, and identifying other causes with similar or identical chain causes, we can determine the expected pattern of miscoded deaths.

The first stage in this process is to parse out years we believe coding practices in the United States to be relatively stable. For dementia, this "gold standard" dataset features 2010-2015, for Parkinson's 2005-2015, and for atrial fibrillation and flutter 2014-2015. We then collect all deaths in those years with the under-coded cause listed as underlying and remove any mention of the under-coded cause from the death certificate. Next, for each unique chain, we search the entire time series of data (1980-2015) to identify the distribution of underlying causes that share that chain. The premise here is that if the diagnosis of dementia, Parkinson's, or atrial fibrillation and flutter were missed, the other causes listed on the death certificate would have been the basis for certification. We then reallocate the under-coded deaths by chain based on that alternative underlying cause distribution.

Upon iterating through all unique chains, we are left with a dataset excluding under-coded causes of death, each remaining cause able to be subdivided into correctly coded deaths and deaths that have been recoded from an under-coded cause by the process described (although not all causes are necessarily targeted by the recoding algorithm). The quantity of interest is the ratio of miscoded deaths to total deaths by cause, age, and sex in our counterfactual dataset.

We apply the ratios derived from the multiple cause data to all VR data to determine the local pattern of miscoding. In this way, the method is sensitive to the observed epidemiology of a given place and time. Then, we calculate the deficit in under-coded cause mortality for each location, year, age, and sex by taking the difference in the expected cause fraction based on prevalence and excess mortality compared to the proportion of deaths actually certified by the VR system. Finally, we scale the cause-specific miscoded deaths to match the deficit and then move them accordingly. We assumed that misclassification of actual dementia and Parkinson's deaths in past years occurred only for reported causes of death that might have plausibly been the direct result of dementia or resulted from misdiagnosis of other organic brain diseases based on clinical expert judgement. A similar assumption is used for atrial fibrillation and flutter, for which only cardiovascular causes and ill-defined garbage codes are considered.

Because the deaths being reallocated vary by location-year, we need a mechanism to ensure plausible limits to how many deaths are extracted from each GBD cause and garbage code. To achieve this, we first run the above-mentioned algorithm on all 5-star VR data (see Section 2.16 of this appendix for an explanation of the star data quality rating system). Then, we determine the 95th percentile of the proportion of deaths moved for each GBD cause and garbage code group by age and sex across location-years among these data. Those values are subsequently stored and applied as the limits for deaths moved by this process.

Redistribute (Step 5)

Achoki T, *et al. J Epidemiol Community Health* 2022; 76:471–481. doi: 10.1136/jech-2021-217480

A crucial aspect of enhancing the comparability of data for CoD is to deal with uninformative, so-called garbage codes. Garbage codes to which deaths were assigned should not be considered as the underlying CoD--for example: “heart failure”, “ill-defined cancer site”, “senility”, “ill-defined external causes of injuries”, and “septicaemia”. The methods for redistributing these garbage-coded deaths were outlined in detail in Naghavi et al[12], and the underlying algorithm for redistributing deaths assigned to these codes has not changed since GBD 2013.

Redistribute HIV-related garbage codes (step 5.1)

Because of the disparate nature of HIV/AIDS mortality across space and time, dynamic redistribution of HIV/AIDS-related garbage codes was needed (Appendix Table 6). To inform this redistribution, we generated target proportions for each garbage group by age band (under 1 month, 1-59 months, 5-19 years, 20-49 years, 50-59 years, 60-69 years, 70-79 years, and 80+ years), five-year time interval, and sex. The garbage groups either target HIV or a remainder target. The allotment of deaths to either of these is based on the regional increase in the mortality rate of all codes in the group relative to the rates seen in 1980–1984 – an increase greater than 5% is assumed to be HIV/AIDS-related, and the proportion of those deaths exceeding 5% are redistributed to HIV/AIDS. Any increase less than or equal to 5% is then assigned to the remainder target.

Regress garbage codes versus non-garbage codes (step 5.2)

For each redistribution package, we defined the “universe” of data as all deaths coded to either the package’s garbage codes or the package’s redistribution targets for each country, year, age, and sex. We then ran a regression based on the following equation separately for each target group and sex:

$$TG_{crt} = \alpha + \beta_1 Gar_{crt} + \beta_2 Age_{crt} Gar_{crt} + \theta_r Gar_{crt} + \gamma_r + \varepsilon_{ct}$$

Where:

TG_{crt} = percentage of deaths within the given garbage code’s universe that were coded to a given target group, by country

Gar_{crt} = percentage of deaths within the given garbage code’s universe that were coded to a given set of garbage codes

α = constant

β_1 = slope coefficient describing the association between Gar_{crt} and TG_{crt}

β_2 = slope coefficient describing the association between the interaction $Age_{crt}Gar_{crt}$ and G_{crt}

γ_r = region-specific random intercept (or super-region if the random effect on region is not significant)

θ_r = region-specific random slope (or super-region if the random effect on region is not significant)

ε_{ct} = standard error, normally distributed and calculated by bootstrapping

This regression was adjusted from GBD 2013 to include fixed effects on the interaction of garbage and age to ensure smooth age patterns. We made this decision after investigating diagnostic visualisations that showed unlikely gaps between proportions assigned to different age groups.

Once proportions were produced for each country, sex, age, and target group, certain adjustments were made to conform our packages to the best medical evidence available. In some cases, we implemented restrictions on the proportions that the regressions could yield. For example, we did not allow any redistribution onto “Chagas disease” outside of Latin America and the Caribbean or “suicide” under the age of 15 years. In other cases, we capped the proportion for some targets to the level that would be produced from proportional redistribution; for example, “haemoglobinopathy” and “haemolytic anaemia” were restricted to the level of proportional redistribution in the redistribution of “left heart failure”. Occasionally, further adjustments were made on a case-by-case basis per country, age, sex, and target group to suppress the impact of outliers based on existing epidemiological evidence and expert judgment.

In GBD 2019, we updated the regressions for stroke and diabetes. We dropped the proportion of garbage from the

regression formula and ran regression on high-quality, low proportion garbage data (4/5 stars, < 50% GC). We also included all covariates included in the CODEm models for both stroke and diabetes.

Development of an algorithm for redistribution of garbage codes based on multiple CoD data

Multiple CoD data are a form of individual record causes of death data that include an underlying CoD along with other causes in the death chain, including intermediate and immediate causes. By analysing this type of data, we can sometimes find the true underlying CoD in other CoD data where the underlying cause is a garbage code or a misassigned CoD. For GBD 2019, this method was expanded and used in redistribution of the following intermediate causes: sepsis, embolism (pulmonary and arterial), heart failure (left, right, and unspecified), acute kidney injury, hepatic failure, acute respiratory failure, pneumonitis, and unspecified central nervous system disorders. Using multiple CoD records for the United States, Mexico, Brazil, Taiwan, Italy, and Colombia we identified the fraction of deaths where the underlying cause of death and the intermediate cause was in the causal chain. Using a mixed effect linear regression, we estimated the fraction of intermediate-cause related deaths by underlying GBD cause. These fractions were multiplied by the GBD 2017 CoDCorrect result to calculate the number of deaths intermediate cause-related deaths for each GBD cause. Lastly, we calculated the "intermediate cause fraction", with total intermediate-cause related deaths as the denominator, by age, sex, location, year GBD cause. These fractions were used to redistribute the intermediate-cause-related deaths to a GBD cause.

To redistribute X59 and Y34 (unspecified injuries) deaths, we used a multi-step approach that utilized the pattern of N-codes in the causal chain in the multiple CoD data. First, we looked at deaths where X59, Y34, and GBD injuries causes were the underlying cause of death and got the pattern of N-codes in the chain. We then derived a cause-specific redistribution proportion based on the probability of a given pattern being coded to X59/Y34 or a GBD injuries cause and summing up these proportions for all patterns. An example below is given for X59:

$$P_{(pattern_j|UCoD\ X59)} = \frac{\#\ of\ pattern_j\ deaths\ |\ UCoD\ X59}{\sum_{j=0}^{m}(\#\ of\ pattern_j\ deaths\ |UCoD\ X59)}$$

$$P_{(GBD\ injuries\ cause_i|pattern_j)} = \frac{\#\ of\ UCoD\ GBD\ injuries\ cause_i\ deaths\ |\ pattern_j}{\sum_{i=0}^{n}(\#\ of\ UCoD\ GBD\ injuries\ cause_i\ deaths\ |pattern_j)}$$

$$redistribution\ proportion_{GBD\ injuries\ cause_i} = \sum_{j=0}^{m}(P(pattern_j|UCoDX59) * P(GBD\ injuries\ cause_i|pattern_j))$$

Where:

$pattern_j$ = a given N-code pattern in the chain of the multiple CoD data

UCoD X59 = a death with X59 coded as the underlying cause of death (UCoD)

UCoD GBD injuries $cause_i$ = a death with a GBD injuries causes coded as the UCoD

We applied these cause-specific redistribution proportions on the data where X59/Y34 were the underlying cause of death to get the number of X59/Y34 deaths "attributable" to each GBD injuries cause. Then, for each GBD injuries cause in the multiple CoD data, we calculated the fraction of redistributed X59/Y34 deaths over the fraction of total injuries death for that cause and modeled this intermediate cause fraction using a mixed effects linear regression similar to the one mentioned above. Like mentioned above, these fractions were then multiplied by GBD 2017 CoDCorrect results, and the cause fractions for X59 and Y34 were calculated by age, sex, location, year, and GBD injuries cause, and then used to redistribute X59 and Y34 deaths to GBD injuries causes.

Additionally, multiple cause of death data was used in the correction of the mis-assignment of deaths due to drug overdoses to unintentional other poisoning. More than 90% of these types of poisonings are due to exposure to narcotics, psychodysleptics, and other drugs, specified or unspecified. More than 97% of these poisonings by substance or drug occurred in ages 15–65 years. These are clearly not cases of accidental ingestion of substances but rather deliberate ingestion and unintentional poisoning. Using multiple CoD records for the United States, Mexico, Brazil, Taiwan, Italy, Colombia, Australia, and various European countries from 1980 to 2017, we selected all deaths with underlying causes coded to X40–X44 (Table A). Table B shows the combination of other potential causes that can be found in the multiple CoD data for these underlying causes, and table B shows the ICD 10 codes corresponding to these causes. On the basis of this table, we

proportionally redistributed mis-assigned unintentional poisoning deaths to one of these causes. The main assumption behind this algorithm is the predominance of the fatality of some substances when a combination of drugs is considered. Given the combination of different drugs and substances in these codes, opium is the main cause of fatality.[13,14] Other substances, like cocaine, methamphetamine, and alcohol in combination with cannabis are less likely to be dominant in fatality.[15]

For example, if the multiple CoD data show that 40% of deaths include opioid use disorders as an intermediate cause where the underlying cause is X40–X44, the redistribution proportion for opioid use disorders will be exactly 40% due to the dominance of the fatality of opioid use disorders compared to other drugs in the above table. Additionally, in our final results, cannabis and psychoactive and psychedelic drug use disorder deaths were mapped to other drug use disorders.

Table A. ICD 10 codes for substances or drugs used to assign deaths coded to an underlying cause of unintentional poisoning by using multiple CoD data

Accidental poisoning codes	All X40, X41, X42, X43, X44 codes
Opioid Codes	T40.0, T40.1, T40.2, T40.3, T40.4, T40.6, F11.0, F11.1, F11.2, F11.3, F11.4, F11.5, F11.6, F11.7, F11.8, F11.9
Amphetamine Codes	T43.6, F15.0, F15.1, F15.2, F15.3, F15.4, F15.5, F15.6, F15.7, F15.8, F15.9
Cocaine Codes	T40.5, F14.0, F14.1, F14.2, F14.3, F14.4, F14.5, F14.6, F14.7, F14.8, F14.9
Psychoactive and psychedelic drug	T40.8, T40.9, T43.6, F16.0, F16.1, F16.2, F16.3, F16.4, F16.5, F16.6, F16.7, F16.8, F16.9
Alcohol Codes	T51.0, F10.0, F10.1, F10.2, F10.3, F10.4, F10.5, F10.6, F10.7, F10.8, F10.9
Cannabis Codes	T40.7, F12.0, F12.1, F12.2, F12.3, F12.4, F12.5, F12.6, F12.7, F12.8, F12.9

Table B. Algorithm for the selection and assignment of a substance or drug use CoD for deaths coded to an underlying cause of unintentional poisoning by using multiple CoD data

Selection Algorithm						
	Opioids	Cannabis	Cocaine	Amphetamines	Alcohol	Psychoactive and psychedelic drugs
Opioids	Opioids	Opioids	Opioids	Opioids	Opioids	Opioids
Cannabis	Opioids	Cannabis	Cocaine	Amphetamines	Alcohol	Psychoactive and psychedelic drugs
Cocaine	Opioids	Cocaine	Cocaine	Amphetamines + cocaine	Cocaine + alcohol	Cocaine
Amphetamines	Opioids	Amphetamines	Amphetamines + cocaine	Amphetamines	Amphetamines + alcohol	Amphetamines
Alcohol	Opioids	Alcohol	Cocaine + alcohol	Amphetamines+ alcohol	Alcohol	Psychoactive and psychedelic drugs
Psychoactive and psychedelic drugs	Opioids	Psychoactive and psychedelic drugs	Cocaine	Amphetamines	Psychoactive and psychedelic drugs	Psychoactive and psychedelic drugs

Multiple CoD data were only available to us for the United States, Mexico, Brazil, Taiwan, Italy, Colombia, Australia, and various European countries. Because of this limited sample, we applied the result from the multiple CoD analysis from each country to its respective super region and used global proportions for Sub-Saharan Africa. We hope for increased availability of multiple CoD data in future analyses to achieve a more precise distribution for more locations.

Verbal autopsy anaemia adjustment (step 5.3)

To compensate for the over-representative cause fractions from anaemia found in VA studies, we redistributed these deaths based on the causal attribution of severe anaemia from the GBD 2015 study. The proportions were country-year-age-sex specific.

Calculate redistribution uncertainty (step 5.4)

We categorized garbage codes into four levels in order of increasing specificity (see Section 2.4 of this appendix). Some garbage codes are redistributed on all causes (e.g., unspecified causes of death) and others are only redistributed onto specific causes (e.g., unspecified cancer). Major garbage refers to garbage codes in Levels 1 or 2. Because of the variation in redistribution, estimating uncertainty from garbage redistribution for CODEm modeling was an important goal for GBD 2019.

We assigned redistribution variance to each data point in the CoD database by calculating residual variance from a regression predicting the percentage of garbage coded deaths redistributed to a cause, given the proportion of garbage codes we observed for that location, year, age, sex, cause, and the age standardized relative rate of major garbage codes across all causes. If there is a cause that has greater residual variance, we assume greater redistribution uncertainty.

The two model inputs are the observed percentage of Levels 1, 2, and 3 garbage codes (by cause, age, sex, location, and year) in redistributed CoD data and the percentage of garbage codes in the raw data (calculated as the age standardized mortality rate ratio of major garbage coded deaths to all deaths in the raw data by location, year, and sex). Level 4 garbage codes were excluded from the model to avoid over estimating uncertainty in countries with high percentages of major garbage codes. Additionally, the classification of Level 4 garbage codes is not stable between successive GBD rounds – for example, “unspecified diabetes” was not a garbage code in GBD 2016, and in GBD 2017 was re-classified as a level 4 garbage code to permit estimation of diabetes by type. These deaths are still taken into account later in the uncertainty estimation process. The model predicts the percentage of garbage coded deaths redistributed to a cause, given the proportion of garbage codes we observed for that location, year, age, sex, cause, and the age standardized relative rate of major garbage codes across all causes. From this model we calculate residual variance. It is important to note that the variance here is a measurement of uncertainty of redistribution, not of the level of miscoding in the raw CoD data for a given demographic.

To calculate variance, a dataset was generated containing percent garbage by location, year, age, sex, and cause, where percent garbage is determined by the equation

$$pct_{garbage} = \frac{deaths_{redistributed} - deaths_{raw}}{deaths_{redistributed}}$$

A mixed-effect linear regression model was then fit to predict the logit percent of deaths from redistribution by age-standardized relative rate of major garbage codes.

$$\begin{aligned} logit\,(pct_{garbage_{ij}}) &= \beta_0 + \beta_1 * \log(ASR_{majorgarbage_{ij}}) + \beta_2 * 15yearage_{ij} + \gamma_{1j} * \log(ASR_{majorgarbage_{ij}}) + u_j \\ &+ e_{ij},\ \ \theta_{\{i\}} \sim N(0, \sigma^2) \end{aligned}$$

Where:

i indexes dataset-location-year-age-sex-cause data points nested within *j* groups by GBD region

ASR major garbage: age standardized relative rate of major garbage

Residual variance, as estimated by the MAD, was calculated for each cause, sex, and age.

The next step was to use the residual variance to calculate uncertainty around each data point in the CoD database. First, we calculated the percent garbage of each data point by treating all deaths that could not be directly mapped to a GBD cause as garbage, including Level 4 garbage codes. Percent garbage was calculated as:

$$pct_{garbage} = \frac{deaths_{redistributed} - deaths_{corrected}}{deaths_{corrected}}$$

Where:

death corrected: deaths post misdiagnosis correction (Appendix Section 2.6)

deaths redistributed: deaths post redistribution (Appendix Section 2.7)

Residual variance was matched to each data point and 100 draws were sampled from a normal distribution by using the cause, age, sex, specific residual variance, and mean of 0. The logit transformed percent garbage was added to each value in the distribution. Each draw was then transformed out of logit space, and the post-redistribution deaths were calculated as

$$deaths = \frac{deaths_{corrected}}{1 - pct\_garbage}$$

Draws of deaths were processed through noise reduction before calculating the final redistribution variance passed to CODEm, which was added to the total data variance. The mean of the draws was not used as the final estimate because it was found that the logit transformation biases the distribution of cause fractions higher than if only point estimates are used.

<u>HIV/AIDS misclassification correction (step 6)</u>[2]

In many location-years, certain causes of death known to be comorbid with HIV/AIDS (e.g., tuberculosis, other infectious diseases) are seen to have age patterns that diverge from those observed in location-years without widespread HIV epidemics and are in fact more reflective of HIV mortality trends. To identify these instances, a global relative age pattern is generated by using all VR deaths in countries with observed HIV prevalence less than 1% by using the following equation

$$RR_{asc} = \frac{R_{asc}}{\bar{x}(R_{65sc}, R_{70sc}, R_{75sc})}$$

Where:

RR_{asc} is the relative death rate for age group *a*, sex *s*, cause *c*;

R_{asc} is the rate for that age group

$\bar{x}(R_{65sc}, R_{70sc}, R_{75sc})$ is the mean of the rates in ages 65–69, 60–74, and 75–79 for that sex and cause.

This is preferable to comparing mortality rates because we are able to isolate divergence in age pattern while accounting for varying levels of overall mortality by fixing death rates to age groups that are unlikely to be confounded by the presence of HIV. Expected deaths for an identified cause were then determined by the equation

$$ED_{lyasc} = \bar{x}(R_{ly65sc}, R_{ly70sc}, R_{ly75sc}) \times p_{lasc} \times RR_{asc}$$

Where:

ED_{lasc} are deaths for location *l*, year *y*, age group *a*, sex *s*, and cause *c*;

$\bar{x}(R_{l65sc}, R_{l70sc}, R_{l75sc})$ is the mean of the rates for ages 65–69, 60–74, and 75–79 for that location-year-sex-cause;

p_{lasc} is the population for that location-year-age-sex-cause

RR_{asc} is the global standard relative rate determined in the previous step for that age-sex-cause.

The expected deaths remain attributed to that particular cause, while the difference between observed and expected are

reallocated to HIV/AIDS.

Scale strata to province (step 7)

Over time, a higher proportion of deaths have been registered in China through the expansion of the DSP system and provincial/county efforts to increase CoD registration. With the expansion of coverage, it is possible that province aggregates do not accurately represent the population distribution between urban and rural areas in each year. For this reason, we stratified the data preparation by urban and rural status for each county within each province. Stratification was based on the median level of urbanisation across counties within each province as recorded in the 2010 China census. In the provinces of Tibet and Hainan, all counties were placed into one strata based on largely homogeneous urbanisation levels within each province. This yielded a total of 62 analytical province-strata. Macao and Hong Kong were not included in this stratification system as the VR systems there are independent from that on the mainland; no weighting scheme needs to be carried out in these complete VR systems with quality data on CoDs.

Within each province-strata, a larger proportion of deaths in-hospital might be reported than that of deaths outside of hospital because of the internet hospital reporting system. To avoid bias, we reweighted in-hospital and out-of-hospital deaths based on the age-sex-province-specific fraction of deaths in and out of hospital in the DSP system. DSP data have been used to establish these percentages because, in these communities, there is a concerted effort to identify all out-of-hospital deaths. Province-strata death rates are combined to produce overall province death rates by weighting each strata by population in each age-sex-year group. Province death rates are rescaled so that all-cause mortality equals the estimated death rate in each age-sex-year estimated in the life-table analysis. The Bayesian noise reduction algorithm was used to deal with zero counts and small number issues for rare causes.[13]

Restrictions post-redistribution (step 8)

Some causes of death can only be reliably assigned through an autopsy by a trained physician. For example, a VA would be unlikely to reliably distinguish between ischaemic and haemorrhagic stroke.

This step ensures that the detail of the cause list at this point in the data prep process is reasonable given the detail of the original data source and the methods by which the CoD was assigned. Two primary corrections are applied. First, any cause that is purely an artifact of the redistribution machinery targeting too detailed a cause is aggregated up to the parent cause. Second, a “bridge map” is applied over a certain set of sources to ensure that these sources do not contain causes that could not reliably be determined by the methods used. These two corrections are applied to ICD-9 detail, ICD-9 BTL, ICD-10 tabulated, ICD-8 detail, ICD-8 A, China DSP (tabulated ICD-9), India MCCD, India SRS, USSR tabulated ICD-9, the Philippine Vital Statistics Reports, Iran ICD-10 VR from the Ministry of Health and Medical Education, and all VA.

Drop VR country years or mark as non-representative (step 9)

Lozano and colleagues[17] describe the negative impact that low-completeness VR data could have on CoD modelling for GBD 2010. In particular, in settings where a data source does not capture all deaths in a population, the cause composition of deaths captured might be different from those that are not. However, a completeness sensitivity test found that low-completeness VR data had little impact on the cause-specific mortality trends at the global level.

For GBD 2019, we investigated the impact of these data at the country and subnational levels by using the more thorough diagnostic visualisations available to us. We determined that these data produced unlikely trends in the models affected. Despite the minimal impact on global trends, better models were produced by eliminating or marking as non-representative data with extremely low completeness. VR completeness was estimated as the number of deaths registered divided by the number of deaths estimated in the GBD mortality envelope.

For this round, VR location-years with completeness less than 50% were dropped, while location-years with completeness between 50% and 69% were marked as non-representative.

In addition, any country-year with a number of deaths registered to major garbage codes greater than 50% of the deaths registered was dropped.

Cause aggregation (step 10)

The cause list is organised in a top-down hierarchical format containing four levels. The first group, or Level 1, sums all causes. Following all cause-mortality are Level 2 causes, which include three broad groupings of causes of deaths: "communicable, maternal, neonatal, and nutritional diseases"; "non-communicable diseases"; and "injuries". Within those Level 2 groupings are finer levels used for modelling. Level 3, or parent causes, are aggregated; the mortality estimate for a parent cause in the hierarchy represents the sum of the causes under that rubric. Sub-causes within Level 3 causes – Level 4 – are more detailed. For example, the parent cause "intestinal infectious diseases" contains the three sub-causes: "typhoid fever", "paratyphoid fever", and "other intestinal infectious diseases". Included in the parent cause estimate are deaths mapped directly to the parent and any Level 4 sub-causes. In data where there was not enough information to assign a Level 4 cause, we aggregated to the Level 3 parent cause. Exceptions to aggregating the Level 4 sub-causes to the parent are instances when certain sub-causes are not present. The United Nations Crime Trends police data only identify homicides, and aggregating homicides to injuries would not accurately represent all injuries.

Remove shocks and HIV/AIDS maternal adjustments (step 11)

For GBD 2019, CODEm models use an HIV/AIDS- and shock-free envelope. To be comparable, cause fractions must also be HIV/AIDS- and shock-free. Cause fractions were uploaded to the CoD database as the number of deaths due to the cause over an adjusted sample in which the number of deaths due to "HIV/AIDS", "conflict and terrorism", "executions and police conflict", and "exposure to forces of nature" were removed.

Remove HIV/AIDS and shocks from denominator where cause list includes HIV/AIDS (step 11.1)

The first step to generate HIV- and shock-free cause fractions was to remove any deaths from the sample that were directly coded to "HIV/AIDS", "collective violence and legal intervention", or "exposure to forces of nature". The cause fraction uploaded to the database can be calculated by a simple equation.

$$CF_{l,t,a,x,c} = \frac{D_{l,t,a,x,c}}{D_{l,t,a,x} - D_{l,t,a,x,hiv} - D_{l,t,a,x,war} - D_{l,t,a,x,disaster}}$$

Where:

$CF_{l,t,a,x,c}$ is the cause fraction for a location *l*, year *t*, age *a*, sex *x*, and cause *c*

$D_{l,t,a,x,c}$ is the number of deaths observed for cause *c* in location *l*, year *t*, age *a*, and sex *x*

$D_{l,t,a,x}$ is the total number of deaths due to all causes observed in location *l*, year *t*, age *a*, and sex *x*

$D_{l,t,a,x,hiv}$, $D_{l,t,a,x,war}$, and $D_{l,t,a,x,disaster}$ are the numbers of deaths observed in location *l*, year *t*, age *a*, and sex *x* for causes "HIV/AIDS," "collective violence and legal intervention," and "exposure to forces of nature," respectively

Cause fractions for HIV/AIDS and shock causes were also uploaded to the database for use in separate estimation processes described by Wang et al.[12] In this case, cause fractions followed the standard equation, with variables following the same explanation.

$$CF_{l,t,a,x,c} = \frac{D_{l,t,a,x,c}}{D_{l,t,a,x}}$$

Remove HIV/AIDS deaths from maternal mortality sources (step 11.2)

HIV-free cause fractions were also uploaded for sources on mortality due to maternal causes. In these cases, the sample of all deaths observed in the study is likely to contain some amount of deaths due to HIV/AIDS and shocks, but the sample only includes cause information on maternal deaths. To account for the presence of HIV/AIDS and shocks in the entire sample, we assumed the same proportion of total deaths due to HIV/AIDS by location, age, sex, and year as provided from the estimation of HIV/AIDS and all-cause mortality described by Wang et al.[18]

Maternal mortality studies were only corrected for HIV/AIDS if the sample of total deaths was provided in the data source. Where sources provided only the MMR, we applied the rate to the HIV- and shock-free envelope produced by the analysis described in Wang et al.[18] and thus did not need to adjust cause fractions at this point in the process.

Where a correction was applied, we used the following equation:

$$CF_{l,t,a,x,mat} = \frac{D_{l,t,a,x,maternal}}{D_{l,t,a,x,maternal} + \frac{E[D_{l,t,a,x,hiv\ shock\ free}]}{E[D_{l,t,a,x}]} D_{l,t,a,x,non-maternal}}$$

Where:

$CF_{l,t,a,x,mat}$ is the resulting cause fraction due to maternal causes for the location (l), year (t), age (a), sex (x);

$D_{l,t,a,x,mat}$ is the number of observed deaths in the sample due to maternal causes

$D_{l,t,a,x,non-maternal}$ is the number of observed deaths in the sample due to non-maternal causes

$E[D_{l,t,a,x}]$ is the GBD estimate of all-cause mortality in the location, year, age, and sex

$E[D_{l,t,a,x,hiv\_shock\_free}]$ is the GBD estimate of HIV- and shock-free mortality in the location, year, age, and sex

HIV/AIDS correction of sibling history, census, and survey data (step 11.3)

As described in our analysis from GBD 2013, many studies have failed to find increased mortality in HIV+ pregnant mothers, but those who have advanced HIV are known to have increased baseline mortality. Prior to GBD 2013, we did not distinguish between deaths in HIV+ women that were caused by pregnancy and those for whom the pregnancy was incidental to their death. To more explicitly quantify the contribution of pregnancy to death in HIV+ women, and therefore more accurately estimate the maternal death count, we completed two additional analyses for GBD 2013 and all subsequent GBD analyses. First, we determined the population attributable fraction (PAF) of HIV/AIDS to pregnancy-related death. Second, we determined the proportion of pregnancy-related deaths in HIV+ pregnant mothers that are aggravated by pregnancy and are therefore by definition maternal deaths.

$$PAF = \frac{P(RR-1)}{1+P(RR-1)}$$

Where:

PAF is the population attributable fraction

P denotes the prevalence of HIV in pregnancy

RR is relative risk of mortality in HIV+ vs HIV- pregnant mothers.

To recap our analysis for GBD 2013, we used the paper published by Calvert and Ronsmans to identify sources[19] that could inform Step 1 of our HIV-correction analysis. We independently reviewed each of the component studies in Calvert and Ronsmans' review and extracted data directly, not from the systematic review paper. We identified only one additional study that was not used in Calvert and Ronsmans' analysis. We have, however, not used all the studies included in that review.

Specific details are as follows:

1) Figueroa-Damian, et al. was excluded for not including any postpartum deaths at all.[20]

2) In the case of Ryder, et al. and Zvandasara, et al. we excluded those deaths that occurred more than 12 months after delivery.[21,22]

3) We excluded the results from Chilongozi, et al. from the site that did not include any HIV- patients.[23]

4) Leroy, et al. was not in the bibliography. We could not locate it for review so it was excluded.[24]

5) Kourtis, et al. was extracted with adjustment of the denominator based on the average number of hospitalisations per delivery in each group.[25]

6) Ticconi, et al. was excluded for being both non- representative and including subgroup data from mothers with malaria infection.[26]

A total of 21 sources were included in our analysis of the increased mortality risk of HIV+ versus HIV- women in pregnancy.[27] We performed DerSimonian-Laird random effects meta-analysis to derive a pooled estimate of RR of death during pregnancy given HIV positivity.[28] The pooled effect size was 6·40 (95% uncertainty interval [UI] 3·98−10·29), which was then used to calculate an HIV PAF for each country, age group, and year. To determine the proportion of those HIV-related deaths that were attributable to maternal causes, we performed a second systematic literature review. This time we sought evidence for the excess mortality risk of pregnancy in those women who are already HIV+. Most studies have failed to find such an effect, but most also did not stratify their study population by stage of HIV or ART (antiretroviral therapy) status. Only two studies did this stratification, with a pooled effect size of 1·13 (95% UI 0·73−1·77).

An updated literature review to inform the relative risk of mortality in pregnancy in HIV+ versus HIV- women had 14 non-usable sources. We completed this search on May 10, 2019, using the following search strings:

((HIV[Title/Abstract] OR "Acquired Immunodeficiency Syndrome"[Title/Abstract] OR AIDS[Title/Abstract]) AND (“pregnant”[Title/Abstract] OR “pregnancy”[Title/Abstract] OR “postpartum”[Title/Abstract] OR "post partum"[Title/Abstract]) AND (“mortality”[Title/Abstract] OR “death”[Title/Abstract]) NOT "case report" NOT (animals[MeSH] NOT humans[MeSH])

AND (2016/08/15[PDat] : 3000/12/31[PDat]))

Prevalence of HIV in pregnant women was calculated by using the Joint United Nations Programme on HIV and AIDS (UNAIDS) Spectrum model, a compartmental HIV progression model used to generate age-specific incidence, prevalence, and death rates from pre-calculated incidence curves and assumptions about intervention scale-up and local variation in epidemiology. For each location, we used UNAIDS’ age-specific ratios of fertility in women living with HIV to fertility in women not living with HIV. In most locations, this ratio is assumed to be greater than one in women aged 15–24 years and less than one and decreasing as age increases beyond 24 years. Since Spectrum assumes fertile ages of 15–49 years, we used the ratio of HIV prevalence in pregnant women to HIV prevalence in the general population at either end of that range to extend estimates to age bands 10–14 years and 50–54 years.

Unlike GBD 2013, when we applied the population attributable fraction (PAF) correction to the envelope of maternal deaths predicted by CODEm, we instead applied country-year-age-group-specific PAF to maternal mortality input data prior to modelling in CODEm. This ensured that both the numerator and denominator of all CF data were internally consistent in their exclusion of background HIV/AIDS mortality. The cause fractions for maternal deaths in sibling history, survey, and census data were therefore adjusted as follows:

$$CF_{l,t,a,x,mat_{adj}} = CF_{l,t,a,x,mat} \times (1 - ProP_{hiv_{l,t,a,x}})$$

$$ProP_{hiv_{l,t,a,x}} = PAF_{l,t,a,x,hivpos} \times (1 - rr_{mat})$$

$$CF_{l,t,a,x,mat_{hiv}} = CF_{l,t,a,x,mat} \times ProP_{maternalhiv_{l,t,a,x}}$$

$$ProP_{maternalhiv_{l,t,a,x}} = PAF_{l,t,a,x,hivpos} \times rr_{mat}$$

Where:

$CF_{l,t,a,x,mat}$ = The proportion of deaths due to all maternal causes before HIV/AIDS correction for the location, year, age, and sex.

$CF_{l,t,a,x,mat_{adj}}$= The proportion of deaths due to maternal causes after the adjustment for the location, year, age, and sex.

$CF_{l,t,a,x,mat_{hiv}}$ = The proportion of deaths due to maternal deaths aggravated by HIV/AIDS after the adjustment for the location, year, age, and sex.

$PAF_{l,t,a,x,hivpos}$ = The PAF that describes the percentage of all maternal deaths that were HIV-related for the location, year, age, and sex

$ProP_{hiv_{l,t,a,x}}$ = The proportion of deaths in pregnancy for the location, year, age, and sex that are estimated to be incidental deaths due to HIV/AIDS and therefore not a maternal CoD.

$ProP_{maternalhiv_{l,t,a,x}}$ =The proportion of deaths in pregnancy for the location, year, age, and sex that are estimated to be HIV+ and maternal deaths that are aggravated by HIV/AIDS.

rr_{mat} = .13/1.13 = The proportion of HIV/AIDS deaths during pregnancy that were exacerbated by the pregnancy.

HIV/AIDS correction of other maternal mortality data (step 11.4)

Although a specific subset of codes in ICD-10 corresponds to HIV/AIDS deaths aggravated by pregnancy, these codes are sparsely used and unreliable. We therefore adapted the method described to also correct VR and VA sources for the systematic exclusion of HIV-related maternal deaths. This correction was calculated in the same manner, by using the same input data as above, with the only difference being that HIV correction of VR and VA sources resulted in a net increase in the maternal correction factor Maternal deaths aggravated by HIV/AIDS are calculated in the following way:

$$CF_{l,t,a,x,mat_{hiv}} = CF_{l,t,a,x,mat} \times ProP_{maternalhiv_{l,t,a,x}}$$
$$ProP_{maternalhiv_{l,t,a,x}} = \frac{PAF_{l,t,a,x,hivpos} \times rr_{mat}}{1 - PAF_{l,t,a,x,hivpos} \times rr_{mat}}$$

<u>Noise Reduction (step 12)</u>

To deal with problems of zero counts in VR, VA, cancer registries, or sibling histories for a given age group in a given year, we use a Bayesian noise-reduction algorithm. For this algorithm, we assume a normal prior and a normal data likelihood. We estimate the normal prior for a given country-series of data by running a Poisson regression to estimate the number of deaths due to each respective cause and sex with dummy variables for age and year. With two notable exceptions (detailed below), these regressions are sex-, cause-, and country-specific, so borrowing strength over age and year is only within a given data type, country, cause, and sex.. The variance of the prior, τ^2, is estimated from the Poisson regression, taking into account the variance-covariance matrix of the regression coefficients. For the data variance, we use the Wilson approximation which provides an estimate of σ^2 even in cases with a zero count of cause-specific deaths. The posterior estimate for each data point is

$$Mean = (\frac{\tau^2}{\tau^2 + \sigma^2} X + \frac{\sigma^2}{\tau^2 + \sigma^2} \mu)$$

$$Variance = (\frac{\tau^2 \sigma^2}{\tau^2 + \sigma^2})$$

Where:

X is the mean of the data

μ is the mean of the prior.

This approach to noise reduction avoids the problem that zero counts in an *ln* rates model or a logit cause fraction model will be dropped from the regression and lead to upward bias in the estimates. This is particularly important in two settings: high-income countries with small numbers of cause-specific deaths, and the analysis of sibling history data where for any given age group in any given year the number of deaths reported in the survey that are pregnancy-related or the number of deaths from all causes in that age group may be small.

Regarding the exceptions to the regression, the first is that country-years with populations under 1 million are pooled with the region data to prevent overdispersion and provide a stronger signal. Additionally, VA data diverge from the above description in two ways. First, all data for a given super-region are pooled together and a study dummy variable is added, allowing for different studies and surveillance sites to borrow strength from one another within a super-region. Second, unless the data are part of a time series (e.g., the Matlab Health and Demographic Surveillance System), the regression has no year component.

Table C: Leading causes of death in South Africa before and after garbage code redistribution, 2015

Achoki T, *et al. J Epidemiol Community Health* 2022; 76:471–481. doi: 10.1136/jech-2021-217480

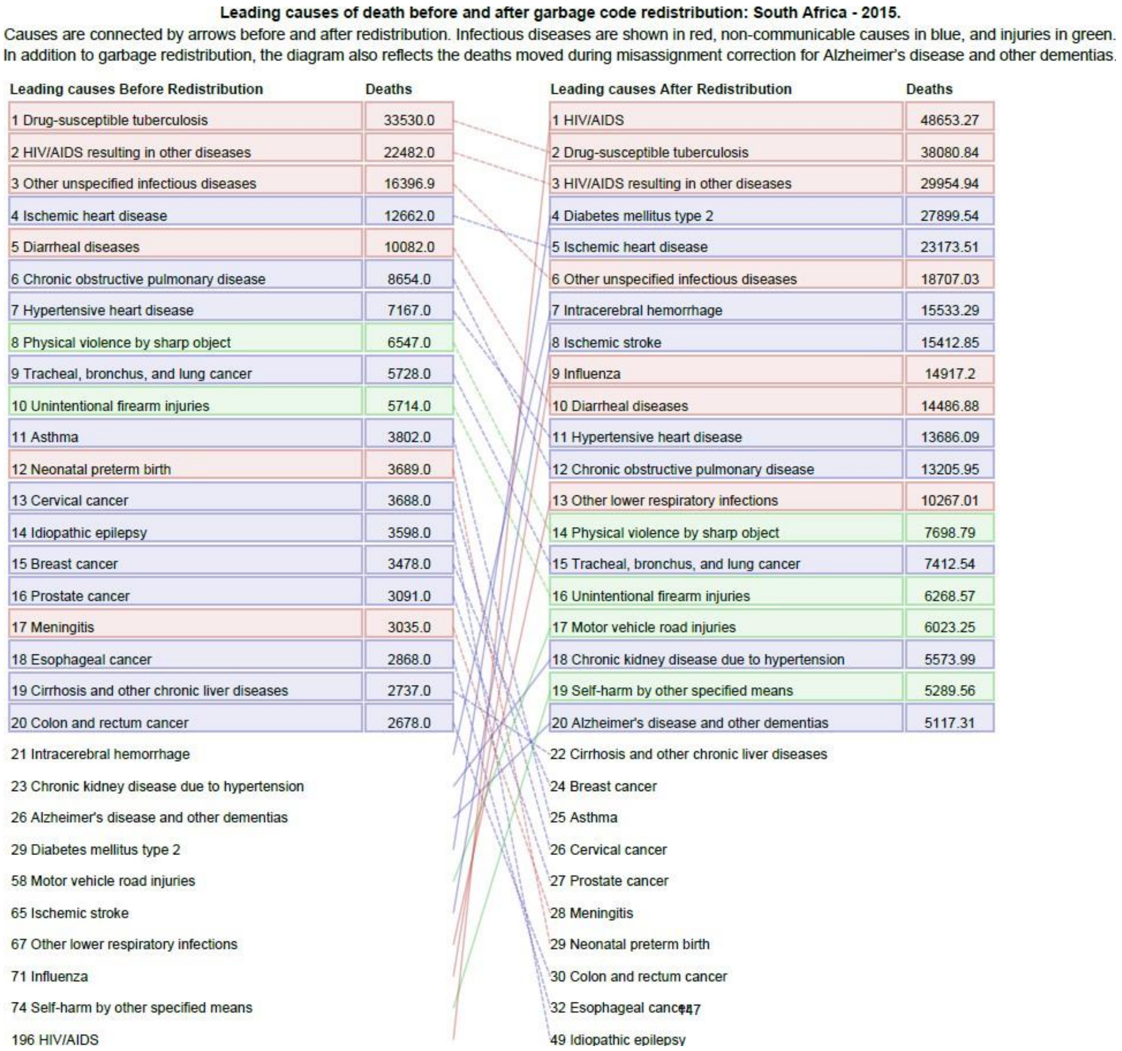

Cause of death database and outlier identification (step 13)

Death rates for different CoD generally have a stable age pattern. In large populations, these patterns will not change very rapidly over time. We can assume a relatively stable pattern in death rates for all causes except for some epidemic diseases and specific types of injuries. Rare causes in large populations and prevalent causes in small populations usually have stochastic patterns. To correct for these stochastic patterns, we implemented a noise-reduction process, explained in Step 12. In VR data, we infrequently find one or more data points for specific geography/age/sex/year combinations that lie very far from the stable pattern of death rates. In these situations, the model usually ignores the data point(s). If the model fails to ignore these data, dramatic jumps or drops can occur in the death rates. When no logical explanation exists for variation in the death rates to this degree, we regard the data point(s) as outlier(s). The selection of data points to regard as outliers occurs after data have been prepped for modelling, as well as during preliminary reviews of the models.

In non-VR sources, data-collection methods and data quality can vary widely from source to source. Where data points in each age-sex-geography-year are very sparse, extreme data points can have a bad effect on regional estimation. In these situations, we investigate the study's methods and consider lower-quality data points as outliers.

Identifying outliers in the CoD data occurs prior to finalisation of models for each cause. We do not automate the selection of outliers but investigate the source of the offending data as well as reviewing other data sources for the same cause,

geography, and year. Ultimately, outliers are identified based on the judgement of the modeller and senior faculty. Outlier decisions are reversible and may be revisited.

<u>Causes of death data star rating calculation</u>

GBD estimates are most accurate when computed with a full time series of complete VR with a low percentage of garbage codes. For GBD 2016, we developed a simple star-rating system from 0 to 5 to give a picture of the quality of data available in a given country over the full time series used in GBD estimates. Countries improve in the star rating as they increase availability, completeness, and detail of their mortality data and reduce the percentage of deaths coded to ill-defined garbage codes or highly aggregated causes (Table 7, Figure 5a and 5b). Underlying indicators for the percent well-certified calculation are listed in Appendix Table 8.

We assign "star" ratings to rate the quality of data for any given location year. The inputs that determine this star rating are the percentage of total deaths determined to be major garbage (such as All, Ill-defined), and the level of comable pleteness in the dataset. Causes such as "injuries" or "cancer" will also be included in major garbage percentage because this percentage includes use of highly aggregated causes. These three values were used to create a "percent well-certified" value between 0 and 1, determined as:

$$pct_{wellcertified} = Completeness \times (1 - pct_{majgarbage})$$

The mapping of percent well certified to star rating is as followed:

0 star: $0\% = pct_{wellcertified}$

1 star: $0\% < pct_{wellcertified} < 10\%$

2 star: $10\% <= pct_{wellcertified} < 35\%$

3 star: $35\% <= pct_{wellcertified} < 65\%$

4 star: $65\% <= pct_{wellcertified} < 85\%$

5 star: $pct_{wellcertified} >= 85\%$

While stars are calculated for each five-year time interval as well as the full time series from 1980 to 2019, stars in the main text are presented for the full time series only.

In the case of VA, all garbage codes are considered ill-defined because redistribution for VA is highly imprecise.
For each VA data source, percent well-certified is

$$pct_{wellcertified} = VerbalAutopsyAdjustment \times (1 - pct_{majgarbage})$$

Where:

$$VerbalAutopsyAdjustment = SubAdj \times RegAdj \times AgeSexCoverage$$

SubAdj is 10% for subnationally representative studies, 100% for nationally representative studies. This adjustment, while arbitrary in its specific value, reflects the bias that can be associated with studies that only cover a potentially non-representative sample of a country's population.

RegAdj is 64% for all VA data sources. This accounts for the inaccuracy of VA in assigning CoD compared to medically verified VR. The specific multiplier 0·64 is based on the chance-corrected concordance of Physician Certified Verbal Autopsy (PCVA) versus medical certification by the Population Health Metrics Research Consortium.[29]

Age-Sex Coverage is the number of deaths estimated in the GBD mortality envelope for the ages and sexes in the study for the country and year divided by the number of deaths estimated in the GBD mortality envelope for the country and year.

Achoki T, *et al. J Epidemiol Community Health* 2022; 76:471–481. doi: 10.1136/jech-2021-217480

Studies that only cover children younger than 5 or maternal mortality, for example, will be highly discounted by this multiplier.

Once percent well-certified is calculated for each location-year of VR and each VA study-year, we then combine these into one measurement for each five-year time interval and the full time series 1980–2019. For each five-year time interval, we take the maximum percent well-certified. Then for 1980–2019, we take the average of the maximum percentages well-certified for the seven five-year time intervals, including any five-year time interval in which no data were available as a zero.

Prior to GBD2019, the causes of death team used an all ages, both sex cause fraction to estimate the percentage of garbage coded deaths in a given location year. Thus, the percentage of garbage for a given location year was determined as:

$$CF_G = \frac{D_G}{D}$$

Where:
CF_G represents the cause fraction of percent garbage.

D_G represents total garbage coded deaths

D represents the total deaths in a given location/year.

In GBD 2019 we moved to calculating the percentage of garbage coded deaths using an age standardized cause fraction. The steps for creating these age standardized cause fractions, in the case of garbage, are as followed:

1. Create both sex, **age specific** cause fractions of garbage for each age group
2. Scale these cause fractions by a set of both sex age weights, determined by global mortality estimates from 2010 to present. That is, weights for each GBD age group were determined as:

$$W_a = \frac{D_a}{D}$$

Where:
W_a is the weight for given age group "a",

D_a is the total both sex, global deaths from 2010 to present in age group "a", and

D is the total both sex, global deaths from 2010 to present across all ages.

3. Sum these weighted cause fractions across all age groups to produce the age standardized cause fraction

In the case of percent garbage for a given location year, the formula to calculate percent garbage would be given as the sum of the weighted age specific cause fractions across all age groups "a":

$$CF_G = \sum_a \left(\frac{G_a}{D_a} \times W_a\right)$$

Where:

G_a represents the total both sex garbage deaths in age group "a,"

D_a represents the total both sex deaths in age group "a," and

W_a represents the weight generated from mortality estimates for age group "a."

ICD-10 and ICD-9 codes assigned to Level 1 or 2 garbage can be found in Appendix Table 4.

Section 2.3: Causes of death modelling methods

CODEm[2]

Overview of methods
Cause of death ensemble modelling (CODEm) is the framework used to model most cause-specific death rates in the GBD.[30] It relies on four key components:

First, all available data are identified and gathered to be used in the modelling process. Although the data may vary in quality, they all contain some signal of the true epidemiological process.

Second, a diverse set of plausible models are developed to capture well-documented associations in the estimates. Using a wide variety of individual models to create an ensemble predictive model has been shown to outperform techniques using only a single model both in CoD estimation[30] and in more general prediction applications.[31,32]

Third, the out-of-sample predictive validity is assessed for all individual models, which are then ranked for use in the ensemble modelling stage.

Finally, differently weighted combinations of individual models are evaluated to select the ensemble model with the highest out-of-sample predictive validity.

For some causes (eg, lower respiratory infections), evidence exists that the relationship between covariates and death rates might differ between children and adults. Separate models are therefore run for different age ranges, when applicable. Additionally, separate models are developed for countries with extensive, complete, and representative VR for every cause to ensure that uncertainty can better reflect the more complete data in these locations.

In order to ensure the addition of subnational locations are not driving changes in estimates, in GBD 2019, we run a global model that excludes data from non-standard locations; the resulting covariate betas are then used as priors for the true global model.

In addition to CoD modelling, we also estimate fatal discontinuities. Fatal discontinuities are events that are stochastic in nature, that cannot be modelled because they do not have a predictable time trend. The fatal discontinuities by cause are aggregated by age and sex and added to the estimated number of deaths in CoD modelling for those causes during CoDCorrect. Details on their methods can be found in Section 3.4.

Model pool development

Because many factors may co-vary with any given CoD, a range of plausible statistical models are developed for each cause. In the CODEm framework, four families of statistical models are used: linear mixed effects regression (LMER) models of the natural log of the cause-specific death rate, LMER models of the logit of the cause fraction, spatiotemporal Gaussian process regression (ST-GPR) models of the natural logarithm of the cause-specific death rate, and ST-GPR models of the logit of the cause fraction (see the 2x2 table in Foreman et al).[30] For each family of models, all plausible relationships between covariates and the response variable are identified. Because all possible combinations of selected covariates are considered for each family of models, multi-collinearity between covariates may produce implausible signs on coefficients or unstable coefficients. Each combination is therefore tested for statistical significance (covariate coefficients must have a coefficient with p-value $< 0{\cdot}05$) and plausibility (the coefficients must have the directions expected on the basis of the literature). Only covariate combinations meeting these criteria are retained. This selection process is run for both cause fractions and death rates, then ST-GPR and LMER-only models are created for each set of covariates. For a detailed explanation of the covariate selection algorithm, see Foreman et al.[30]

Data variance estimation

Achoki T, *et al. J Epidemiol Community Health* 2022; 76:471–481. doi: 10.1136/jech-2021-217480

The families of models that go through ST-GPR described in Section 3.1.2 incorporate information about data variance. The main inputs for a Gaussian process regression (GPR) are a mean function, a covariance function, and data variance for each data point. These inputs are described in detail in Foreman et al.[30] For GBD 2019, we have updated this calculation to incorporate garbage code redistribution uncertainty.

Three components of data variance are now used in CODEm: sampling variance, non-sampling variance, and garbage code redistribution variance. The computation of sampling variance and non-sampling variance has not changed since previous iterations of the GBD and is also described in Foreman et al.[30] Garbage code redistribution variance is computed in the CoD database process described in Section 2.7 of this appendix. Since variance is additive, we calculate total data variance as the sum of sampling variance, non-sampling variance, and redistribution variance. Increased data variance in GPR results in the GPR draws not following the data point as closely.

Testing model pool on 15% sample

The performance of all models (individual and ensemble) is evaluated by means of out-of-sample predictive validity tests. Thirty percent of the data are randomly excluded from the initial model fits. These individual model fits are evaluated and ranked by using half of the excluded data (15% of the total), then used to construct the ensembles on the basis of their performance. Data are held out from the analysis on the basis of the cause-specific missingness patterns for ages and years across locations. Out-of-sample predictive validity testing is repeated 20 times for each model, which has been shown to produce stable results.[30] These performance tests include the root mean square error (RMSE) for the log of the cause-specific death rate, the direction of the predicted versus actual trend in the data, and the coverage of the predicted 95% UI.

Ensemble development and testing

The component models are weighted on the basis of their predictive validity rank to determine their contribution to the ensemble estimate. The relative weights are determined both by the model ranks and by a parameter ψ, whose value determines how quickly the weights taper off as rank decreases. The distribution of ψ is described in more detail in Foreman et al.[30] A set of ensemble models is then created by using the weights constructed from the combinations of ranks and ψ values. These ensembles are tested by using the predictive validity metrics described in Section 3.1.4 on the remaining 15% of the data, and the ensemble with the best performance in out-of-sample trend and RMSE is chosen as the final model.

Final estimation

Once a weighting scheme has been chosen, 1000 draws are created for the final ensemble, and the number of draws contributed by each model is proportional to its weight. The mean of the draws is used as the final estimate for the CODEm process, and a 95% UI is created from the 0·025 and 0·975 quantiles of the draws. The validity of the UI can be checked via its coverage of the out-of-sample data; ideally, the 95% UI would capture 95% of these data. Higher coverage suggests that the UIs are too large, and lower coverage suggests overfitting.

Selection of causes for which CODEm is used

CODEm is used to model 193 causes, described in detail in Section 3.3. However, it is unsuitable for use in modelling certain causes, including those with very low death counts, those where cause-specific death record availability is inadequate, or those for which there are marked biases or variability for CoD certification over time that cannot be fully accounted for with the current garbage code redistribution algorithms. Criteria for causes where CODEm is not used are discussed in further detail in Section 3.2.

Model-specific covariates

Modellers select covariates to be used in CODEm, but those covariates may not be significant or in the direction specified

during the covariate selection step of CODEm and will therefore not be used in the model. These covariates are listed with a '—' for number of draws. Additionally, covariates may be selected by CODEm but only exist in submodels that perform poorly and may end up with zero draws included in the final ensemble. Finally, all other covariates are listed with the number of draws in the final ensemble from submodels that had the covariate.

Causes modelled outside of CODEm[2]

Overview

A number of causes required alternative modelling strategies to those used for CODEm because they were not compatible with CODEm estimation infrastructure and processes. Such unsuitability included having very low death counts; inadequate availability of cause-specific death records; and marked biases or variability for CoD certification over time that could not be fully accounted for with current garbage code redistribution algorithms. The inclusion of these causes in CODEm often renders its out-of-sample predictive validity testing unstable, but the validity of this type of testing is a key advantage of using CODEm for CoD estimation. Alternately, CODEm simply fails to generate plausible mortality rates in the absence of enough VR or VA data when these causes are included. Because of increased data availability and redistribution algorithm refinements, we were able to incorporate several new causes, which were modelled separately for GBD 2013, into CODEm for this iteration of the GBD study; with each annual update of GBD, we aim to add more causes within the CODEm estimation space. For GBD 2019, we used alternative modelling approaches for these causes, including negative binomial models, natural history models, sub-cause proportion models, and prevalence-based models (appendix table 10).

Negative binomial models

For eight rare causes of death, too few observed deaths were included in the CoD database to produce stable estimates. For these causes, we ran negative binomial regression models, with either a constant or a constant multiplied by the mean assumption for the dispersion parameter, by using reverse step-wise model building. We selected one of the two model dispersion assumptions based on best fit to the data by using the same method as GBD 2013. For GBD 2015, we also tested zero-inflated Poisson models for these rare causes of death but rejected them after finding that they did not substantially affect the mean predictions but instead produced unrealistically large UIs. Descriptions of the modelling process for each of these causes follows in the next sections.

DisMod-MR 2.1

Until GBD 2010, non-fatal estimates were based on a single data source on prevalence, incidence, remission, or a mortality risk selected by the researcher as most relevant to a particular location and time. For GBD 2010, we set a more ambitious goal: to evaluate all available information on a disease that passes a minimum quality standard. That required a different analytical tool that would be able to pool disparate information presented in varying age groupings and from data sources by using different methods. The DisMod-MR 1.0 tool used in GBD 2010 evaluated and pooled all available data, adjusted data for systematic bias associated with methods that varied from the reference, and produced estimates with UIs by world regions. For GBD 2013, the improved DisMod-MR 2.0 had increased computational speed, allowing computations that were consistent between all disease parameters at the country rather than the region level. The hundred-fold increase in speed of DisMod-MR 2.0 was partly due to a more efficient rewrite of the code in C++ but also to changing to a model specification using log rates rather than a negative binomial model used in DisMod-MR 1.0. In cross-validation tests, the log rates specification worked as well as or better than the negative binomial specification.[33] For GBD 2015, the computational engine (DisMod-MR 2.1) remained substantively unchanged, but we re-wrote the wrapper code that organised the flow of data and settings at each level of the analytical cascade. The sequence of estimation occurred at five levels: global, super-region, region, country, and, where applicable, subnational locations (see flow diagram of DisMod-MR 2.1 cascade that follows). The super-region priors were generated at the global level with mixed-effects, non-linear regression by using all available data; the super-region fit, in turn, informed the region fit and so on down the cascade. The wrapper gave analysts the choice to branch the cascade in terms of time and sex at different levels depending on data density. The default used in most models was to branch by sex after the global fit but to retain all years of data until the

lowest level in the cascade. For GBD 2015, we generated fits for the years 1990, 1995, 2000, 2005, 2010, and 2015.

In updating the wrapper, we consolidated the code base into a single language, Python, to make the code more transparent and efficient and to better deal with subnational estimation. The computational engine is limited to three levels of random effects; we differentiated estimates at the super-region, region, and country levels. In GBD 2013, the subnational units of China, Mexico, and the UK were treated as countries, such that a random effect was estimated for every location with contributing data. However, the lack of a hierarchy between country and subnational units meant that the fit to country data contributed as much to the estimation of a subnational unit as the fits for all other countries in the region. We found inconsistency between the country fit and the aggregation of subnational estimates when the country's epidemiology varied from the average of the region. Adding an additional level of random effects required a prohibitively comprehensive rewrite of the underlying DisMod-MR engine. Instead, we added a fifth layer to the cascade, with subnational estimation informed by the country fit and country covariates, plus an adjustment based on the average of the residuals between the subnational unit's available data and its prior. This procedure mimicked the impact of a random effect on estimates between subnationals.

For GBD 2015, we improved how country covariates differentiate non-fatal estimates for diseases with sparse data. The coefficients for country covariates were re-estimated at each level of the cascade. For a given location, country coefficients were calculated by using both data and prior information available for that location. In the absence of data, the coefficient of its parent location was chosen to utilise the predictive power of our covariates in data sparse situations.

For GBD 2017, the DisMod-MR 2.1 tool was used. Updates included estimation of new age groups through the GBD 2017 terminal age group of 95 years and older in addition to the new locations added for the GBD 2017 cycle.

DisMod-MR 2.1 likelihood estimation

Analysts have the choice of using a Gaussian, log-Gaussian, Laplace, or log-Laplace likelihood function in DisMod-MR 2.1. The default log-Gaussian equation for the data likelihood is as follows:

$$-log[p(y_j|\Phi)] = \log(\sqrt{2\pi}) + \log(\delta_j + s_j) + \frac{1}{2}\left(\frac{\log(a_j + \eta_j) - \log(m_j + \eta_j)}{\delta_j + s_j}\right)^2$$

Where:

y_j is a measurement value (ie, data point)

Φ denotes all model random variables

η_j is the offset value, *eta*, for a particular integrand (prevalence, incidence, remission, excess mortality rate, with-condition mortality rate, cause-specific mortality rate, relative risk, or standardised mortality ratio)

a_j is the adjusted measurement for data point j, defined by

$$a_j = e^{(-u_j - c_j)} y_j$$

Where:

u_j is the total area effect (ie, the sum of the random effects at three levels of the cascade: super-region, region, and country)

c_j is the total covariate effect (ie, the mean combined fixed effects for sex, study-level, and country-level

covariates), defined by

$$c_j = \sum_{k=0}^{K[I(j)]-1} \beta_{I(j),k} \hat{X}_{k,j}$$

with standard deviation (SD)

$$s_j = \sum_{l=0}^{L[I(j)]-1} \zeta_{I(j),l} \hat{Z}_{k,j}$$

Where:

k denotes the mean value of each data point in relation to a covariate (also called x-covariate)

$I(j)$ denotes a data point for a particular integrand, j

$\beta_{I(j),k}$ is the multiplier of the kth x-covariate for the ith integrand

$\hat{X}_{k,j}$ is the covariate value corresponding to the data point j for covariate k

l denotes the SD of each data point in relation to a covariate (also called z-covariate)

$\zeta_{I(j),k}$ is the multiplier of the lth z-covariate for the ith integrand

δ_j is the SD for adjusted measurement j, defined by

$$\delta_j = log[y_j + e^{(-u_j - c_j)}\eta_j + c_j] - log[y_j + e^{(-u_j - c_j)}\eta_j]$$

Where mj denotes the model for the jth measurement, not counting effects or measurement noise and defined by

$$m_j = \frac{1}{B(j) - A(j)} \int_{A(j)}^{B(j)} I_j(a)\, da$$

Where:

$A(j)$ is the lower bound of the age range for a data point j

$B(j)$ is the upper bound of the age range for a data point j

$I(j)$ denotes the function of age corresponding to the integrand for data point j

The source code for DisMod-MR 2.1 as well as the wrapper code is available at https://github.com/ihmeuw/ihme-modelling/tree/master/gbd_2017/shared_code/central_comp/nonfatal/dismod.

Natural history models

For some causes for which CoD data may be systematically biased either owing to misclassification or because the disease exists in focal communities without VR or VA studies, we have developed natural history models. In natural history models, incidence and case-fatality rates are modelled separately and then combined to produce estimates of cause-specific mortality.

Prevalence-based models

The modelling strategies for atrial fibrillation and flutter are distinct from those used for other causes modelled as natural history models. These models use prevalence estimates and excess mortality rates (EMR) generated through DisMod-MR 2.1 rather than incidence and case-fatality rates.

Sub-cause proportion models

For certain sub-causes for which accurate diagnoses are known to be very difficult, we first modelled the parent cause in the GBD hierarchy with CODEm and then allocated deaths to specific causes by using proportions of the parent cause for each age-sex-location-year for each sub-cause. For these causes, we identified no significant predictors in negative binomial regressions. This approach was taken because the available data on these specific causes may come from sources other than VR, such as end-stage renal disease registries, or may come from too few places to model the death rates directly. Details for each cluster of causes analysed in this way follow.

Section 2.4: Central computation[2]

Imported cases

Imported cases are fatalities that occur in a geographic area where a particular CoD is known to be eradicated in a specific time period or where infection cannot occur. We apply space-time restrictions to these causes in the modelling strategy for that location and time period. However, in some rare cases, deaths from these causes occur outside of restricted locations and time periods. These deaths are referred to as imported cases.

Illustrating this concept, Chagas disease is transmitted by insect vectors that only exist in the Americas. For this reason, Chagas disease is restricted in the models for countries such as Russia. However, someone traveling in Latin America could contract Chagas disease and then die after returning home to Russia. Imported cases accounts for these kinds of deaths.

To calculate these imported cases, we find all cases from the VRs of data-rich countries for any CoD that is otherwise geographically or temporally restricted. We then create a beta distribution from that data point by using the sample size of the VR for that data point and upload these draws as a custom CoD model. This model is then used as an input to CoDCorrect.

CoDCorrect

Objective of CoDCorrect

As mentioned in the main text, the CoD models are cause-specific. As such, there is no guarantee that the sum of these models will equal the results of the all-cause mortality estimates or that model results of child causes add up to the parent model results. The CoDCorrect process is used to make the CoD and all-cause mortality estimates internally consistent by using a very simple algorithm.

Algorithm and levels

The core algorithm remains the same as it did in GBD 2013. The equation can be written as follows:

$$CD_{lyasjd} = D_{lyasjd}\left(\frac{PD_{lyasjd}}{\sum_{j=1}^{j=k} D_{lyasjd}}\right)$$

Where:

CD_{lyasjd} is the corrected number of deaths for a location l, year y, age a, sex s, cause j, and draw d

PD_{lyasjd} is the parent CoD for a location l, year y, age a, sex s, cause j, and draw d

D_{lyasjd} is the uncorrected number of deaths estimated from a cause-specific model for a l, year y, age a, sex s, cause j, and draw d

The CoDCorrect process starts by rescaling the Level 1 causes to match the all-cause mortality estimates (used for PD_{lyasjd} in the previous equation). Level 2 causes are then rescaled to their corrected parent causes. This process continues until all levels of the hierarchy have been rescaled. Causes and their levels within the CoDCorrect hierarchy can be found in appendix table 9.

Since GBD 2017, HIV has not been included in the CoDCorrect process. To account for this change, Level 1 CoDCorrect causes are rescaled to HIV-deleted mortality estimates that are produced as part of the mortality and HIV estimation process. Results from the GBD version of Spectrum are added to the post-CoDCorrect death estimates with fatal discontinuities and imported cases to generate the full set of death estimates.

Diagnostic results of CoDCorrect by cause and location

For more detail on diagnostic results of CodCorrect by cause see appendix table 15.

<u>Years of life lost calculation</u>

Years of life lost (YLL) owing to premature mortality were computed for 1082 locations and 39 years. First, we used the lowest observed age-specific mortality rates by location and sex across all estimation years from locations with total populations greater than 5 million in 2016 to establish a theoretical minimum risk reference life table.

The YLL is a metric that is computed by multiplying the number of estimated deaths by the standard life expectancy at age of death. The metric therefore highlights premature deaths by applying a larger weight to deaths that occur in younger age groups. We propagated uncertainty from CoDCorrected deaths for all demographics. The core equation can be written as follows:

$$YLL = \sum_{c=1,a=0,s=1}^{\infty} d_{cas}e_a$$

<u>GBD world population age standard</u>

Age-standardised populations in the GBD were calculated by using the GBD world population age standard. For GBD 2013, GBD 2015, and GBD 2016, the age-specific proportional distributions of all national locations from the UN Population Division World Population Prospects 2012 revision for all years from 2010 to 2035 were used to generate a standard population age structure by using the non-weighted mean across all the aforementioned country-years. For GBD 2017, we used the non-weighted mean of 2017 age-specific proportional distributions from the GBD 2017 population estimates for all national locations with a population greater than 5 million people in 2017 to generate an updated standard population age structure.[34] For GBD 2019, we have continued to use this method using GBD 2019 population estimates.[35]

Section 2.5: Non-fatal outcome estimation[3]

The GBD 2019 non-fatal estimation process describes the steps necessary to estimate incidence, prevalence, and YLDs for

disease and injury sequelae in GBD 2019. Conceptually, the estimation effort is divided into eight major components: (1) compiling data sources through data identification and extraction; (2) data adjustment; (3) estimation of prevalence and incidence by cause and sequelae by using DisMod-MR 2.1 or alternative modelling strategies for selected cause groups; (4) estimation by impairment; (5) severity distributions; (6) incorporation of disability weights (DWs); (7) comorbidity adjustment; and (8) the estimation of YLDs by sequelae and causes. Section 4.12 contains additional detail specific to each non-fatal disease, impairment, and injury, and their sequelae. Non-fatal modelling strategies vary significantly between causes.

Data sources, identification, and extraction[3]

Systematic reviews

For GBD 2019, updated systematic reviews were conducted for 49 causes. Over 123,925 studies were screened for inclusion, and over 1250 articles were newly incorporated into GBD 2019 non-fatal models. For other disease sequelae, only a small fraction of the existing data appears in the published literature, and other sources predominate, such as survey data, disease registers, notification data, or hospital inpatient data. As was done in past rounds of GBD, data were systematically screened from household surveys archived in the GHDx (http://ghdx.healthdata.org/), including Demographic and Health Surveys, Multiple Indicator Cluster Surveys, Living Standards Measurement Surveys, and Reproductive Health Surveys. Other national health surveys were identified on the basis of survey series that had yielded usable data for past rounds of GBD, sources suggested to us by in-country collaborators, and surveys identified in major multinational survey data catalogues such as the International Household Survey Network and the WHO Central Data Catalog, as well as through country Ministry of Health and Central Statistical Office websites. Case notifications reported to the WHO were updated through 2019. Citations for all data sources used for non-fatal estimation in GBD 2019 are provided in searchable form through a web tool (http://ghdx.healthdata.org/). A description of the search terms used for cause-specific systematic reviews are detailed by cause in Section 4.12.

Survey data preparation

For GBD 2019, survey data for which we have access to the unit record data constitute a substantial part of the underlying data used in the estimation process. During extraction, we concentrated on demographic variables (eg, location, sex, age), survey design variables (eg, sampling strategy and sampling weights), and the variables used to define the population estimate (eg, prevalence or a proportion) and a measure of uncertainty (standard error, confidence interval or sample size, and number of cases).

Disease registries

For GBD 2019 non-fatal estimation, disease registries were an important source for a select number of conditions such as cancers, end-stage renal disease, and congenital disorders.
Registry data is particularly key in the estimation of neoplasms when we consider the increasing attention to non-communicable diseases, particularly cancers, in low and middle-income areas of the world. The GHDx source tool (http://ghdx.healthdata.org/data-type/disease-registry) provides a comprehensive list of registry data used in GBD estimation processes.

Estimation of hospital envelope

Figure A. Overview process of estimation of hospital envelope.
This process utilises administrative data, reported tabulations, and survey microdata to estimate the rates of inpatient admissions per capita for every location and demographic group in the GBD hierarchy.

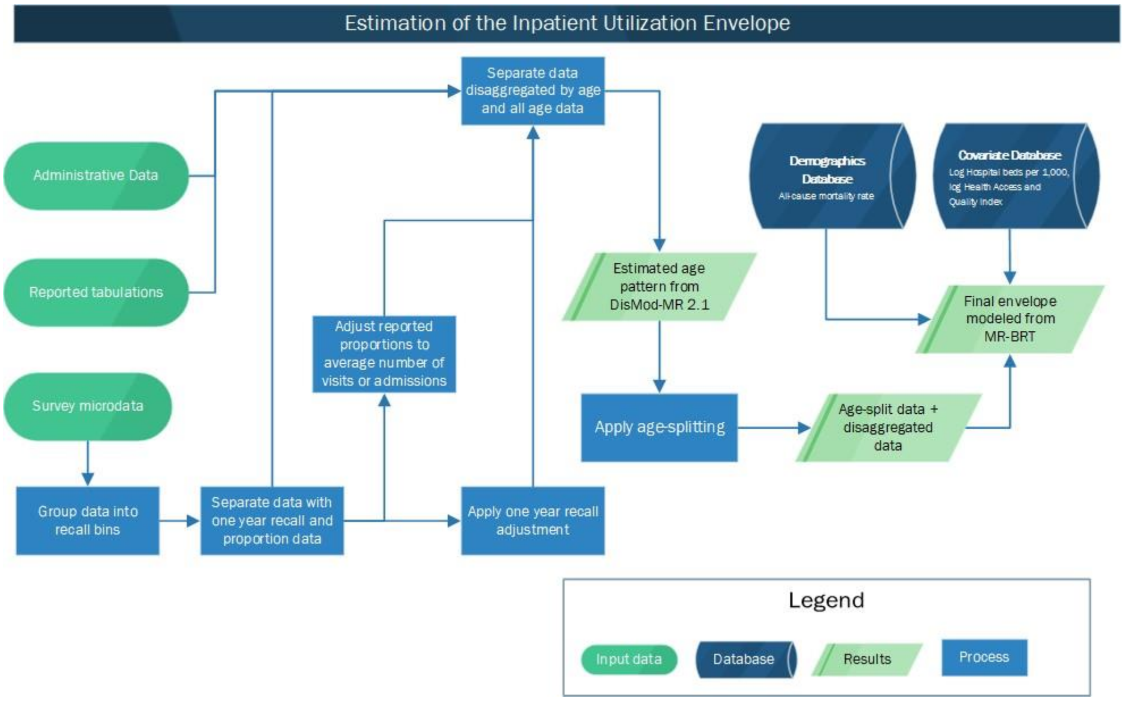

Input data and methods summary[3]

Case definition

We defined a hospital admission as admission into a formal health care facility for an overnight stay. However, we excluded admissions to long-term care facilities (>120 days), nursing care facilities, and facilities staffed by traditional or spiritual healers.

Input data

We searched the GHDx for population surveys, administrative records, and censuses from January 1990 to September 2017. We applied the following keyword filters: "Health care use" OR "Length of stay" AND "Hospitals" OR "Health care services". We applied no language restrictions to our search and required all returned records to contain either microdata or tabulated reports. We searched the returned records' metadata for measures of inpatient care. For inclusion, we required all measures to be nationally or subnationally representative. Additionally, we consulted with experts and GBD collaborators to gather data sources that were not within the GHDx.

To estimate inpatient admission rates for newborns, we input estimates of the in-facility delivery (IFD) rates for every subnational and national location at 5-year intervals starting at 1990 and including the most recent 2019 estimate. IFD was estimated by using an ST-GPR model based on population-representative surveys and administrative data. We accepted data sources from 28,646 location-years (1413 from administrative records and 27,233 from population surveys).

Modelling strategy[3]

Data adjustment

We classified each of the accepted data sources into four data types: (1) proportion of survey respondents who were admitted into the hospital in the last 30 days; (2) proportion of survey respondents who were admitted to the hospital in the last year; (3) average number of admissions (utilisation rate) reported by survey respondents in the last year; and (4)

average number of visits reported by annual administrative records. We assigned measures reported by annual administrative records as our reference group because these data types were free from recall bias and most closely matched our case definition. From data sources for which microdata were available, we extracted and binned the data based on gender and age groups of less than 1 year, 1–4 years, 4–9 years, 10–14 years, and similar increments of years up to 95 years and older.

We crosswalked each of the three non-reference (survey) data types to the reference (administrative record) data type through the use of penalised spline regressions to account for non-systematic differences between the data types. For each non-reference data type and each sex, we looked for overlap between the non-reference data type and the reference data type based on location, year, age group, and sex. With the overlapping data, we calculated the ratio of the point estimate from the reference data type, μ_{ref}, to the non-reference data type, μ_s. We fit these ratios with a penalised spline regression equation

$$\ln\left(\frac{\mu_{ref,i}}{\mu_{s,i}}\right) = h(age_i) + \varepsilon_i \quad (1)$$

Where:

i denotes a given matched observation

$h(age_i)$ represents a basis function that estimated a cross-validated, penalised spline over the population weighted mean age of the age group

ε represents the residual

In the figures that follow, for each non-reference data type, we plot the ratio of μ_{ref} and μ_s across age and by sex and the predictions from the penalised spline regressions.

Figure B. Global age-sex specific crosswalks to equate each non-reference data type to the reference data type.

For each non-reference data type and each sex, we plotted the ratio of reference data points to non-reference data points, which were matched based on location, age group, year, and sex. Using a penalized spline regression, we estimated the crosswalk between each non-reference data type and the reference type. We plotted the crosswalk and the associated prediction error in the following figures:

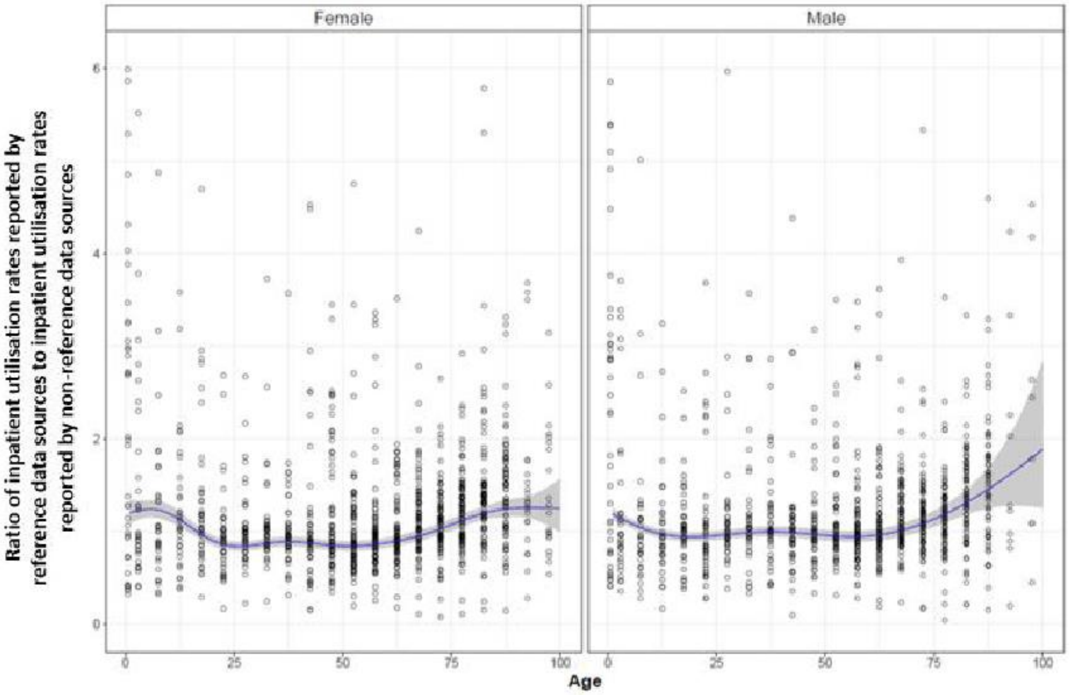

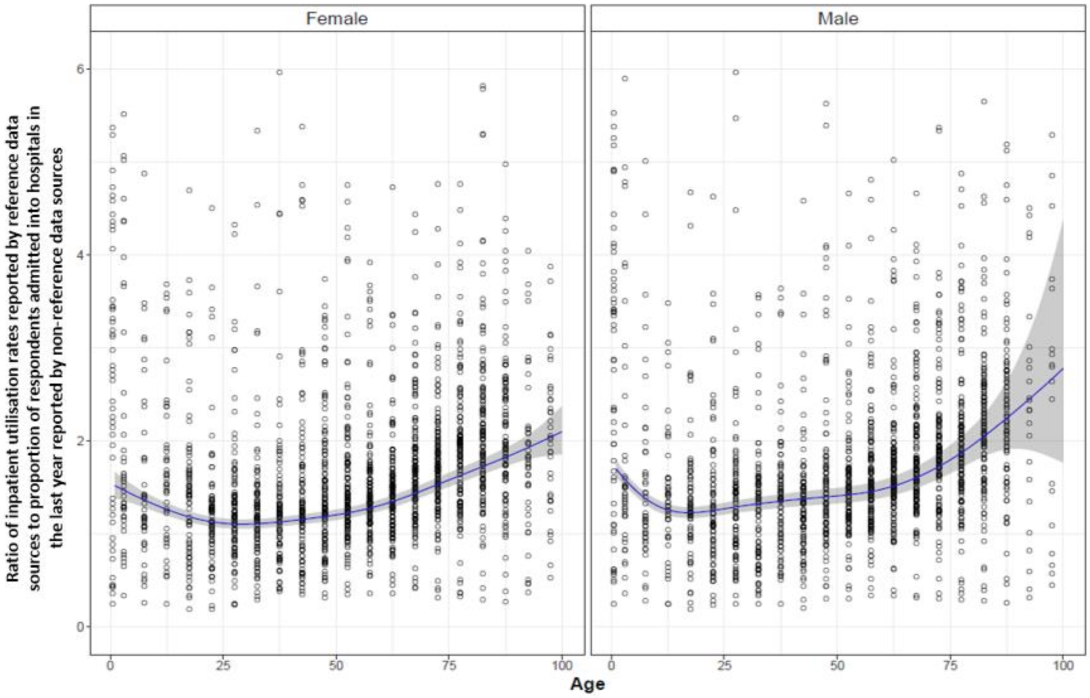

Achoki T, *et al. J Epidemiol Community Health* 2022; 76:471–481. doi: 10.1136/jech-2021-217480

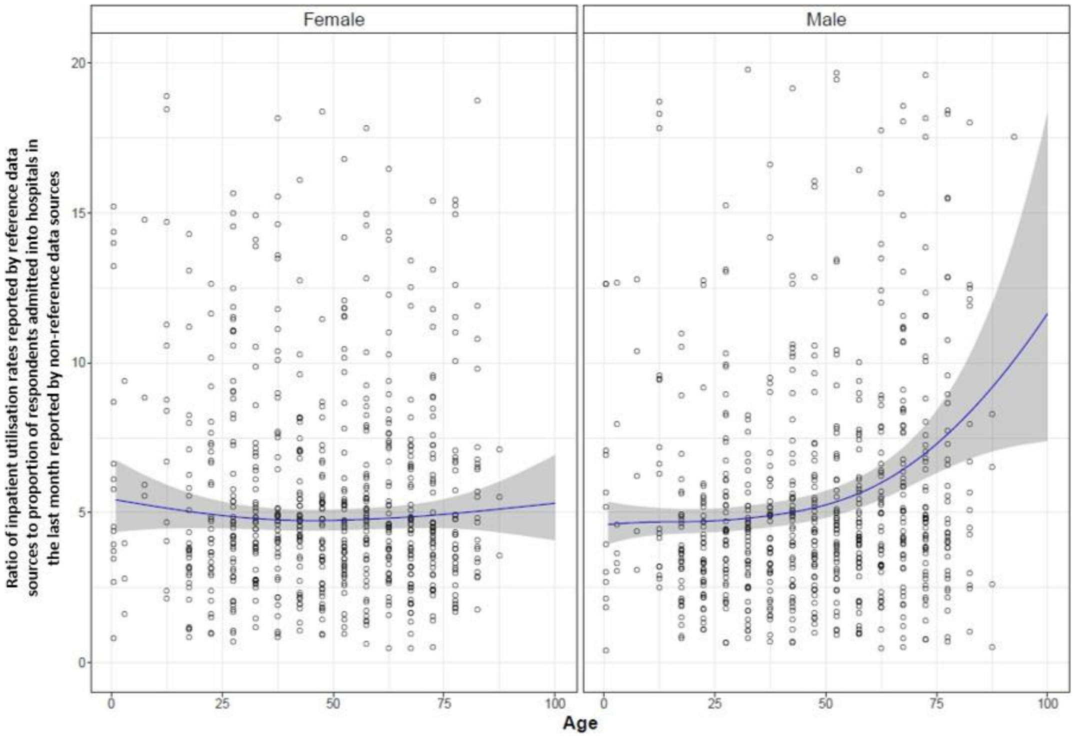

To crosswalk non-reference data types to reference data types, we multiplied non-reference data types by the exponentiated predictions from respective penalised spline regressions. Uncertainty from the adjustments was accounted for by the equation

$$se_a = \sqrt{se_m^2 \cdot se_s^2 + se_m^2 \cdot \mu_s^2 + se_s^2 \cdot \mu_m^2} \quad (2)$$

Where:

se_a is the standard error of the adjusted non-reference data point
se_m is the standard error of the exponentiated crosswalk prediction
se_s is the standard error of the non-reference data point
μ_s is the mean of the non-reference data point
μ_m is the exponentiated crosswalk prediction from the penalised spline regression

Age-sex splitting

Before modelling, we ran a Dismod-MR 2.1 model with data disaggregated by age to estimate countries' age-pattern and then applied the estimated age-pattern to split aggregated all-age data into the age groups that are necessary 5-year age groups encouraged by ST-GPR. This procedure was done by calculating a constant, k, which was the ratio of the aggregated all-age data point, $\mu_{all\ age}$, to the all-age estimated utilisation rate from the DisMod-MR 2.1 model, $\hat{\mu}$

$$k = \frac{\mu_{all\ age}}{\hat{\mu}} \quad (3)$$

The constant, k, was then multiplied by age-specific utilisation rates from the DisMod-MR 2.1 model. The uncertainty from the data and the age-pattern were propagated by following Equation 2. The split data were then incorporated into the final DisMod-MR 2.1 model.

ST-GPR modelling

The input data were modelled by using ST-GPR to allow for smoothing over age, time, and location in locations that were missing complete datasets. To further help explain variation in geographies with little to no data, we used the covariates of the natural log of hospital beds per 1000 and the HAQ Index for every location. Hospital beds per 1000 was estimated by using ST-GPR on data sourced from the World Bank. Coefficients for the covariates are presented in the table that follows.

Table B. Estimated coefficients of the hospital envelope model.

**Covariate**	**Sex**	**Coefficient (95% UI)**	**Exponentiated Coefficient**
Log hospital beds per 1000	Male	0.41<br>(0.36 to 0.45)	1.50<br>(1.44 to 1.57)
	Female	0.41<br>(0.37 to 0.45)	1.50<br>(1.45 to 1.56)
HAQ Index	Male	0.029<br>(0.027 to 0.030)	1.029<br>(1.027 to 1.030)
	Female	0.028<br>(0.026 to 0.029)	1.028<br>(1.027 to 1.029)
All-cause mortality	Male	2.14<br>(2.11 to 2.17)	8.49<br>(8.25 to 8.73)
	Female	2.33<br>(2.30 to 2.36)	10.24<br>(9.93 to 10.55)

Claims, inpatient hospital, and outpatient data

Claims, inpatient hospital, and outpatient data played a key role in the process of estimating many non-fatal causes in GBD 2019. All sources of administrative clinical data were aggregated and processed together for all causes of disease that included this type of data in their estimates. Data sources were heterogeneous in granularity, comprehensiveness, and level of detail, and the methods described below were used to transform data to be comparable and complete across locations, ages, sexes, and years, and causes.

Claims data

For GBD 2019, we accessed aggregate data derived from the Truven database of USA private health insurance and subset of public insurance schemes of Medicaid and Medicare for the years 2000, 2010-2016. The population covered in each year was 3.3 million in 2000, 40.4 million in 2010, 44.4 million in 2011, 40.8 million in 2012, 42.2 million in 2013, 36.4 million in 2014, 22.6 million in 2015, and 22.4 million in 2016. For each of these individuals, information on every health service encounter was collected and all episodes of care were linked to individuals by unique identifiers. Outpatient claims could have up to four diagnoses while inpatient claims had up to 15 diagnoses. Data from Taiwan (province of China), the Philippines, Poland, Russia, and Singapore were also incorporated as claims data. We mapped ICD diagnoses in each source to GBD causes. GBD conditions were extracted as “prevalence” or “incidence” depending on cause duration and based on the specification of the research team responsible for the cause. In a given year, for each individual in the claims data, a prevalent case was defined as any mention in any diagnostic field associated with any claim, including inpatient and outpatient encounters. To reduce noise from spurious coding practices, an additional requirement is placed on prevalence in outpatient claims whereby a minimum of two claims must be filed in a calendar year to count as a prevalent case. An incident case was defined the same way but assumed that claims within a condition-specific duration were the same case. In this way, an individual could have multiple incident cases in a given year, but double-counting of cases with multiple claims from a single illness episode was avoided.

Figure C. GBD 2019 Claims Data Processing

Achoki T*, et al. J Epidemiol Community Health* 2022; 76:471–481. doi: 10.1136/jech-2021-217480

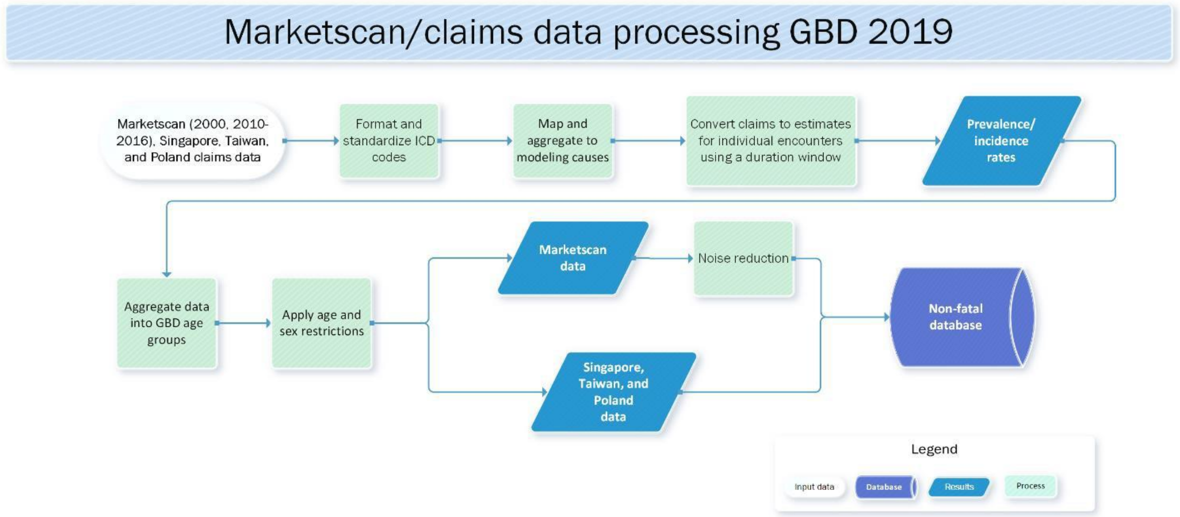

Inpatient hospital admissions

Inpatient hospital data were extracted from 4401 location-years in 45 countries. ICD coding was standardised across sources and versions of ICD. Counts of admissions with a primary diagnosis of each cause were extracted from all sources and modelled through the inpatient hospital process. Secondary diagnostic detail was included in estimation through corrections as described below. A case of any cause of disease was defined as an overnight inpatient admission with a primary diagnosis of that cause.

For GBD 2015, our use of hospital data in non-fatal disease estimation was limited by the challenge of accessing accurate information on coverage populations for any given data source. Section 4.1.4 of the appendix describes the modelling strategy that was developed for the hospital utilisation envelope, an estimate of admission per capita in each location. In GBD 2016, we used the hospital utilisation envelope in place of information on coverage population. We calculated age-specific and sex-specific cause fractions in each inpatient hospital data source and multiplied these fractions by the hospital utilisation envelope to produce incidence or prevalence rates. In GBD 2017, we used the same approach except the hospital envelope was measured in ST-GPR to accommodate admissions data reflecting newborns being delivered in facilities. In GBD 2019, we updated the modelling framework to the hospital utilisation envelope, adding all-cause mortality as a covariate and improving the space-time smoothing to more accurately fit locations with and without data.

We performed three adjustments on inpatient hospital data to synthesise all inpatient sources to the same definition of care and to account for cases that were not captured in some inpatient sources depending on data availability. Data were first adjusted to account for multiple admissions for a single case of disease. It was then adjusted to account for cases of any cause that were non-primary reasons for admission. Finally, admissions were scaled by the ratio of outpatient cases observed for any inpatient case of disease to account for additional cases that did not warrant an inpatient admission. Combined with the uncorrected version (with no scalar applied), this process resulted in four stages of incidence and prevalence estimates from inpatient hospital data: (1) (un-corrected) inpatient admissions by episode, primary diagnosis; (2) inpatient admissions by individual, primary diagnosis only; (3) inpatient hospital admissions, accounting for all diagnoses; and (4) an estimate of inpatient admissions and outpatient visits by individual, accounting for all diagnoses. Estimate 4 was applied to all causes except those where outpatient care or non-primary diagnosis was not expected based on the nature of the disease. Adjustment ratios were calculated using all clinical inpatient sources that had patient-level data and primary and non-primary diagnoses. Sources of this data include Marketscan and Taiwan (province of China) claims data as described above; claims and inpatient data from Singapore, the Philippines, Ecuador, and New Zealand; and the HCUP SID database spanning years 2003–2008. Only Marketscan and Taiwan (province of China) claims data included a link between inpatient and outpatient care to be used in the fourth estimate described. Ratios from these sources were modelled over age

and sex using a mixed-effects model in MR-BRT for each cause. If data for any ratio did not exist for the youngest or oldest age groups, we assumed a uniform tail on the model from the nearest age group with data. All models were conducted in log-space in order to bound the model to be greater than one for any age, sex, and cause. We used the following equations for each of the three scalars:

1) Correction to account for multiple admissions, which gives us inpatient admissions by individual, primary diagnosis only
   a. $inpatient^{1°}_{admin} * (\frac{inpatient^{1°}_{indiv}}{inpatient^{1°}_{admin}}) = inpatient^{1°}_{indiv}$
2) Correction to adjust for non-primary diagnoses, which gives us inpatient admissions by individual, all diagnoses
   a. $inpatient^{1°}_{admin} * (\frac{inpatient^{all}_{indiv}}{inpatient^{1°}_{admin}}) = inpatient^{all}_{indiv}$
3) Correction to account for inpatient and outpatient care, which gives us inpatient admissions and outpatient visits by individual for all diagnoses
   a. $inpatient^{1°}_{admission} * (\frac{inpatient^{all}_{indiv} \cup outpatient^{all}_{indiv}}{inpatient^{1°}_{admissions}}) = inpatient|outpatient^{all}_{indiv}$

Determination of maternal causes used separate cause-fractions and a different scalar calculated from a maternal hospital admissions rate instead of the hospital envelope, and the equation

$$(\frac{events}{\#\ of\ total\ hospital\ visits}) * (\frac{hospital\ visits}{live\ births}) * (\frac{births}{population})$$

Determination of injuries used a separate correction factor from those described above which adjusted data that was only E-coded by data that contained E-codes and N-codes (nature of injury codes) with the following equation

$$\frac{1}{\frac{E\text{-}code\ primary\ dx}{E\text{-}code\ any\ dx + N\text{-}code\ any\ dx}}$$

A final adjustment was applied to each of the above estimates. The HAQ Index was used to account for differences in access and quality of health care across time and space. The HAQ Index adjustment was applied by dividing the above estimates by a scalar ranging from 0 to 100, where 0 represents the first percentile of observed access and quality and 100 the 99th percentile.

Figure D. GBD 2019 Inpatient Hospital Data Processing

Achoki T, *et al. J Epidemiol Community Health* 2022; 76:471–481. doi: 10.1136/jech-2021-217480

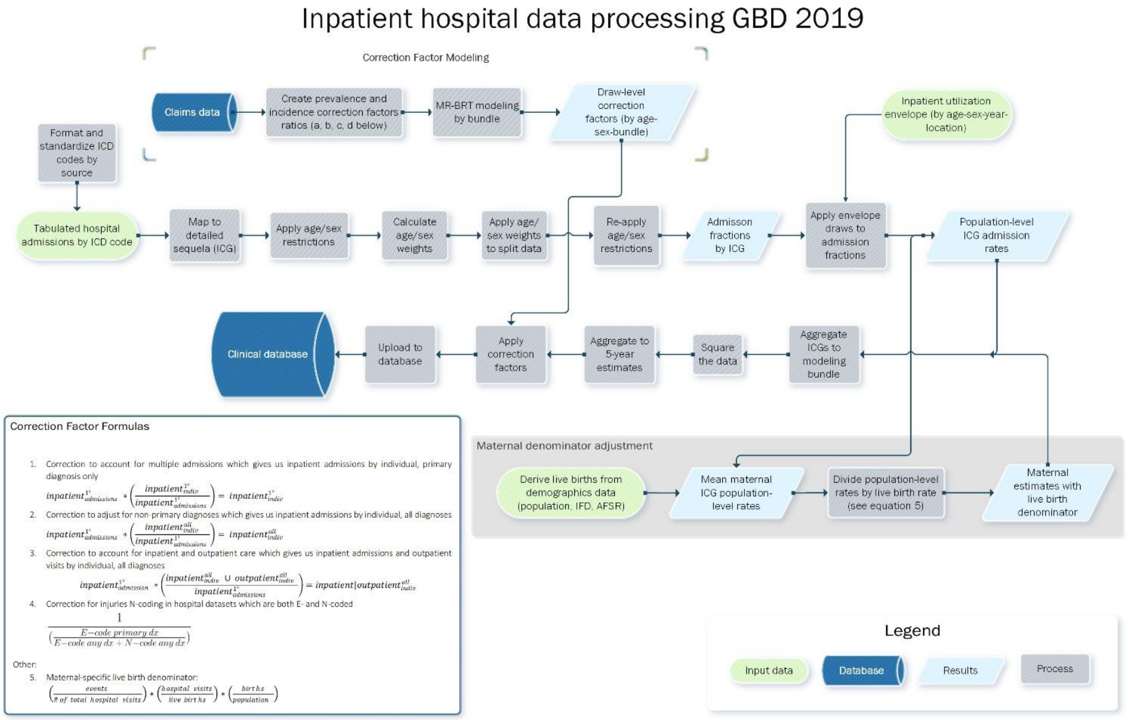

Achoki T, *et al. J Epidemiol Community Health* 2022; 76:471–481. doi: 10.1136/jech-2021-217480

Outpatient encounter data

Outpatient encounter data were available from the USA and Sweden for 109 location-years. No changes were made in the processing of outpatient data from GBD 2017, except for updates to the ICD mappings to GBD cause.

As with the inpatient hospital data, a scalar was calculated by using Marketscan claims data to adjust for multiple visits per individual within one year (for prevalent conditions) and within a cause-specific duration (for incident causes).

Figure E. GBD 2019 Outpatient data extraction process

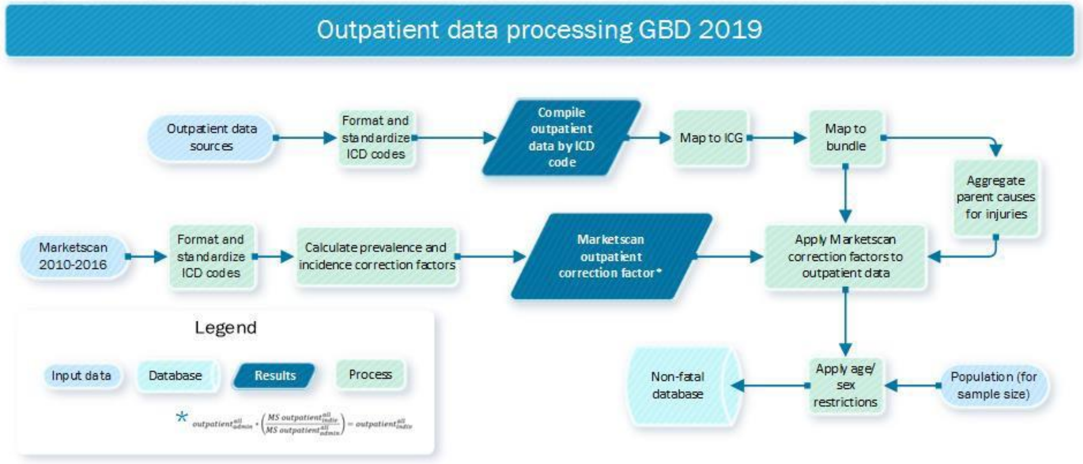

Case notifications

Case notifications, active screening, intervention coverage studies, and surveillance contributed to estimates of infectious diseases. If data were available, we extracted it from survey and administrative microdata; otherwise, data were extracted from published literature and reports. For many infectious diseases and neglected tropical diseases (NTDs), we used of cases for which notification was made by countries to the WHO and other global monitoring entities. The causes for which we used WHO case notification data included tuberculosis, measles, yellow fever, rabies, dengue, cholera, whooping cough, human African trypanosomiasis (HAT), meningitis, all sexually transmitted infections, and other infectious diseases and NTDs, such as Ebola.

Data adjustment

MR-BRT and Fitting Procedures
This section details the statistical models underlying MR-BRT, and fitting procedure used to obtain estimates. Further details on models and algorithms can be found in the technical report.[36]
The MR-BRT program is a set of wrappers customized for global health problems that use the open source mixed effects package LimeTr (https://github.com/zhengp0/limetr). We describe the basic functionality in the sections below.

Mixed-Effects Model
We consider the following nonlinear mixed effects model:

$$\begin{aligned} \boldsymbol{y}_i &= \mathbf{F}_i(\beta) + \mathbf{Z}_i\boldsymbol{u}_i + \boldsymbol{\epsilon}_i \\ \boldsymbol{u}_i &\sim N(\mathbf{0},\boldsymbol{\Gamma}),\ \boldsymbol{\Gamma} = \mathrm{diag}(\gamma), \qquad \boldsymbol{\epsilon}_i \sim N(\mathbf{0},\boldsymbol{\Lambda}), \end{aligned} \tag{1}$$

where $y_i \in \mathbb{R}^{n_i}$ is the vector of observations from the ith study, $\boldsymbol{\epsilon}_i \in \mathbb{R}^{n_i}$ are measurement errors with given covariance $\boldsymbol{\Lambda}$, $\boldsymbol{u}_i \in \mathbb{R}^{k_\gamma}$ are independent random effects, and $\mathbf{Z}_i \in \mathbb{R}^{n_i \times k_\gamma}$is a linear map, and β are regression coefficients. The models F_i may be nonlinear.

To fit (β, γ) we solve the marginal likelihood problem:

$$\min_{\beta,\gamma} f(\beta,\gamma) := \sum_{i=1}^{m} \frac{1}{2}(\boldsymbol{y}_i - \mathbf{F}_i(\beta))^\top (\mathbf{Z}_i \boldsymbol{\Gamma} \mathbf{Z}_i^\top + \boldsymbol{\Lambda}_i)^{-1} (\boldsymbol{y}_i - \mathbf{F}_i(\beta)) + \frac{1}{2} \ln \left| \mathbf{Z}_i \boldsymbol{\Gamma} \mathbf{Z}_i^\top + \boldsymbol{\Lambda}_i \right|. \quad (2)$$

When the model is linear, we can write:

$$\mathbf{F}_i(\beta) = \boldsymbol{X}\beta. \quad (3)$$

Linear models are very common in cross-walks, and for network analysis, which is detailed below.

Network Analysis

Network analysis is a special case of the linear model (3) that is used to compare multiple treatment effects. To explain the coding we use a running example with four treatments A, B, C, D.

For simplicity assume *A* is this reference treatment. We then have the following coding.

$$AB \to B - A: \quad [1 \quad 0 \quad 0]$$
$$AC \to C - A: \quad [0 \quad 1 \quad 0]$$
$$AD \to D - A: \quad [0 \quad 0 \quad 1].$$

We see from this simple example that the design matrix under the basic network assumption is always full rank, since a subset of rows forms the identity matrix.

Comparisons that do not include the reference can be computed. For example,

$$BC \to C - B = (C - A) - (B - A)$$
$$= [0 \quad 1 \quad 0] - [1 \quad 0 \quad 0]$$
$$= [-1 \quad 1 \quad 0]$$

Using this simple algebra, we quickly obtain the remaining codings.

$$BC \to C - B: \quad [-1 \quad 1 \quad 0]$$
$$BD \to D - B: \quad [-1 \quad 0 \quad 1]$$
$$CD \to D - C: \quad [0 \quad -1 \quad 1]$$

Each row of the design matrix $\boldsymbol{X}$ is coded according to the comparison.

When doing network analysis, the design matrix $\boldsymbol{X}$ does not include the intercept term (**1** column).

Constraints and Priors

The ML estimate (2) can be extended to incorporate nonlinear inequality constraints

$$\mathbf{C}(\boldsymbol{\theta}) \leq c,$$

where $\boldsymbol{\theta} = (\beta, \gamma)$. Constraints play a key role for polynomial splines.

It is also essential to allow priors on parameters of interest. We assume that priors are given by a functional form

$$\boldsymbol{\theta} \sim \exp(-\rho(\boldsymbol{\theta}))$$

The likelihood problem is then augmented by adding the term $\rho(\boldsymbol{\theta})$ to the ML objective. The function ρ may be nonlinear and nonconvex, but we assume it is smooth.

Trimming outliers

Least trimmed squares (LTS) is a robust estimator[37,38] for the standard regression problem. Given the problem

$$\min_{\beta} \sum_{i=1}^{n} \frac{1}{2}(y_i - \langle \boldsymbol{X}_i, \beta \rangle)^2, \quad (4)$$

the LTS estimator minimizes the sum of *smallest h* residuals rather than all residuals. These estimators were initially introduced to develop linear regression estimators that have a high breakdown point (in this case 50%) and good statistical efficiency (in this case $n^{-1/2}$). Breakdown refers to the percentage of outlying points which can be added to a dataset before the resulting M-estimator can change in an unbounded way. Here, outliers can affect both the outcomes and training data (features).

LTS estimators are robust against outliers, and arbitrarily large deviations that are trimmed do not affect the final $\hat{\beta}$.

Rather than writing the objective in terms of order statistics, it is far simpler to extend the likelihood using an auxiliary variable $\boldsymbol{W}$:

$$\min_{\beta, \mathbf{W}} \sum_{i=1}^{n} w_i \left(\frac{1}{2}(y_i - \langle \mathbf{X}_i, \beta \rangle)\right)^2 \quad \text{s.t.} \quad \mathbf{1}^\top \boldsymbol{W} = h, \quad \mathbf{0} \leq \boldsymbol{W} \leq \mathbf{1}. \quad (5)$$

The set

Achoki T, *et al. J Epidemiol Community Health* 2022; 76:471–481. doi: 10.1136/jech-2021-217480

$$\Delta_h := \{\mathbf{W} : \mathbf{1}^\top \mathbf{W} = h, \quad \mathbf{0} \le \mathbf{W} \le \mathbf{1}\} \tag{6}$$

is known as the *capped simplex*, since it is the intersection of the h-simplex with the unit box.[37] For a fixed β, the optimal solution of (5) with respect to $\mathbf{W}$ assigns weight 1 to each of the smallest h residuals, and 0 to the rest. Problem (5) is solved *jointly* in $(\beta, \mathbf{W})$, simultaneously finding the regression estimate and classifying the observations into inliers and outliers. This joint strategy makes LTS different from post-hoc analysis, where a model is fit first with all data, and then outliers are detected using that estimate.

To explain how trimming enters the marginal likelihood problem, we focus on a single group term from the ML likelihood (2):

$$\frac{1}{2}(\boldsymbol{y}_i - \mathbf{F}_i(\beta))^\top (\mathbf{Z}_i \boldsymbol{\Gamma}^{-1} \mathbf{Z}_i^\top + \boldsymbol{\Lambda}_i)^{-1} (\boldsymbol{y}_i - \mathbf{F}_i(\beta)) + \frac{1}{2}\ln|\mathbf{Z}_i \boldsymbol{\Gamma}^{-1} \mathbf{Z}_i^\top + \boldsymbol{\Lambda}_i|$$

We introduce auxiliary variables $\mathbf{W}_i \in \mathbb{R}^{n_i}$, and define

$$\boldsymbol{r}_i := y_i - \mathbf{F}_i(\beta), \;\; \mathbf{W}_i := \mathrm{diag}(\mathbf{W}_i), \;\; \sqrt{\mathbf{W}_i} := \mathrm{diag}(\sqrt{\mathbf{W}_i}).$$

We now form the objective

$$\frac{1}{2}\boldsymbol{r}_I^\top \sqrt{\mathbf{W}_i}\left(\sqrt{\mathbf{W}_i}\mathbf{Z}_i \boldsymbol{\Gamma}^{-1}\mathbf{Z}_i^\top \sqrt{\mathbf{W}_i} + \boldsymbol{\Lambda}_i^{\odot \mathbf{W}_i}\right)^{-1} \sqrt{\mathbf{W}_i}\,\boldsymbol{r}_i + \frac{1}{2}\ln|\sqrt{\mathbf{W}_i}\mathbf{Z}_i \boldsymbol{\Gamma}^{-1}\mathbf{Z}_i^\top \sqrt{\mathbf{W}_i} + \boldsymbol{\Lambda}_i^{\odot \mathbf{W}_i}|, \tag{7}$$

where $\odot$ denotes the elementwise power operation:

$$\boldsymbol{\Lambda}_i^{\odot \mathbf{W}_i} := \begin{bmatrix} (\lambda_{1j})^{w_{i1}} & 0 & \cdots & 0 \\ 0 & \ddots & \ddots & \vdots \\ 0 & \cdots & 0 & (\lambda_{in_i})^{w_{in_i}} \end{bmatrix} \tag{8}$$

When $w_{ij} = 1$, we recover the contribution of the ijth observation to the original likelihood. As $w_{ij} \downarrow 0$, the ijth contribution to the residual is correctly eliminated by $\sqrt{w_{ij}} \downarrow 0$. The jth row and column of $\sqrt{\mathbf{W}_i}\mathbf{Z}_i\boldsymbol{\Gamma}^{-1}\mathbf{Z}_i^\top\sqrt{\mathbf{W}_i}$ both go to 0, while the jth entry of $\boldsymbol{\Lambda}_i^{\odot \mathbf{W}_i}$ goes to 1, which effectively removes all impact of the jth point on the covariance matrix.

For full details and analysis, please see the technical report.[36]

Final Estimator

Putting together the trimmed ML with priors and constraints, we arrive at the following estimator.

$$\begin{aligned} \min_{\beta,\gamma,\mathbf{W}} \; f(\beta,\gamma,\mathbf{W}) := \sum_{i=1}^{m} \frac{1}{2} r_i^\top \sqrt{\mathbf{W}_i}\left(\sqrt{\mathbf{W}_i}\mathbf{Z}_i\boldsymbol{\Gamma}^{-1}\mathbf{Z}_i^\top\sqrt{\mathbf{W}_i} + \boldsymbol{\Lambda}_i^{\odot \mathbf{W}_i}\right)^{-1} \\ \sqrt{\mathbf{W}_i}\, r_i + \frac{1}{2}\ln|\sqrt{\mathbf{W}_i}\,\mathbf{Z}_i\boldsymbol{\Gamma}^{-1}\mathbf{Z}_i^\top\sqrt{\mathbf{W}_i} + \boldsymbol{\Lambda}_i^{\odot \mathbf{W}_i}| + \rho(\beta,\gamma,\boldsymbol{\Lambda}) \end{aligned} \tag{9}$$

$$\text{s.t. } \boldsymbol{r}_i = \boldsymbol{y} - \mathbf{F}_i(\beta), \;\; \mathbf{1}^\top\mathbf{W} = h, \;\; 0 \le \mathbf{W} \le 1, \;\; \boldsymbol{C}\begin{pmatrix}\beta \\ \gamma\end{pmatrix} \le c\,.$$

The fit is obtained using iterative optimization techniques. Problem (9) is nonlinear and non-smooth, and the optimization is implemented in the LimeTR package[3] (https://github.com/zhengp0), and relies on the IPopt interior point method.[39]

Nonlinear Dose-Response Curves with Constrained Splines

In this section we discuss spline models for dose-response relationships. General background on splines and spline regression are available elsewhere.[40,41]

B-splines and bases

A spline basis is a set of piecewise polynomial functions with designated degree and domain. If we denote polynomial order by p, and the number of knots by k, we need $p + k$ basis elements s_j^p, which can be generated recursively as illustrated in Figure A.

Figure A. Recursive generation of b-spline basis elements (orders 0, 1, 2)

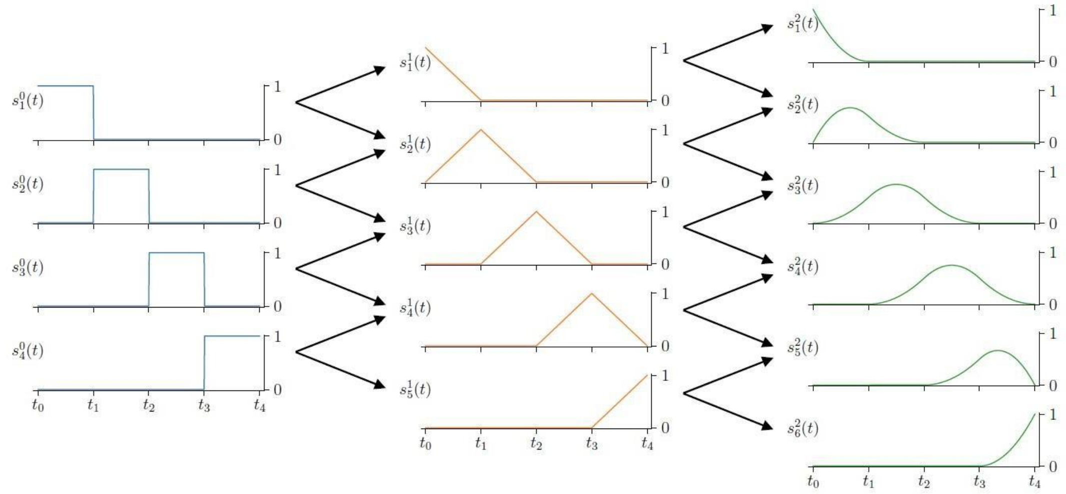

.

Given such a basis, we can represent any dose-response relationship as the linear combination of the spline basis elements, with coefficients $\beta \in \mathbb{R}^{p+k}$:

$$f(t) = \sum_{j=1}^{p+k} \beta_j^p s_j^p(t). \tag{10}$$

These coefficients are then inferred as part of the general estimator (9) as discussed in the previous section. An explicit representation of (11) is obtained by building a design matrix $\mathbf{X}$. Given a set of t values at which we have data, the jth column of $\mathbf{X}$ is given by the expression

$$\mathbf{X}_{\cdot,j} = \begin{bmatrix} s_j^p(t_0) \\ \vdots \\ s_j^p(t_k) \end{bmatrix}. \tag{11}$$

The model for direct observations data coming from (11) can now be written compactly as

$$\boldsymbol{y} = \mathbf{X}\beta + \mathbf{Z}_i\boldsymbol{u}_i + \boldsymbol{\epsilon}_i \,,$$

which is a special case of the main problem class (1).

Shape constraints

We can impose shape constraints such as monotonicity, concavity, and convexity on splines. Constraints on splines have been developed in the past through reformulation techniques.[42] The development in this section uses explicit constraints instead.

Monotonicity. Spline monotonicity across the domain of interest follows from monotonicity of the spline coefficients.[40] Given coefficients

$$\beta = \begin{bmatrix} \beta_1 \\ \vdots \\ \beta_n \end{bmatrix},$$

the curve $f(t)$ in (11) is monotonically non-decreasing when

$$\alpha_1 \leq \alpha_2 \leq \cdots \leq \alpha_n$$

and monotonically non-increasing if

$$\alpha_1 \geq \alpha_2 \geq \cdots \geq \alpha_n.$$

The relationship $\alpha_1 \leq \alpha_2$ can be written as $\alpha_1 - \alpha_2 \leq 0$. Stacking these inequality constraints for each pair (α_i, α_{i+1}) we can write all constraints simultaneously as

$$\underbrace{\begin{bmatrix} 1 & -1 & 0 & \cdots & 0 \\ 0 & 1 & -1 & \cdots & 0 \\ \ddots & \ddots & \ddots & \ddots & \vdots \\ 0 & \cdots & \cdots & 1 & -1 \end{bmatrix}}_{\mathbf{C}} \begin{bmatrix} \alpha_1 \\ \alpha_2 \\ \alpha_3 \\ \vdots \\ \alpha_n \end{bmatrix} \leq \begin{bmatrix} 0 \\ 0 \\ \vdots \\ 0 \end{bmatrix}.$$

These linear constraints are a special case of the general estimator (9) that allows $\mathbf{C}(\beta) \leq c_\beta$.

Convexity and Concavity. For any twice continuously differentiable function: $f : \mathbb{R} \to \mathbb{R}$, convexity and concavity are captured by the signs of the second derivative. Specifically, f is convex if $f''(t) \geq 0$ is everywhere, and concave if $f''(t) \leq 0$ everywhere. We can compute $f''(t)$ for each interval, and impose linear inequality constraints on these expressions.

Enforcing linear tails. For large consumption with little data, we need the capability to ensure that the last segment of the spline is linear, with slopes that match the adjacent segment at the knot. The estimated spline is then a best fit to the data, subject to this specification. Priors on the tails can also be provided.

Figure B. Spline extrapolation. Left: linear extrapolation. Right: nonlinear extrapolation.

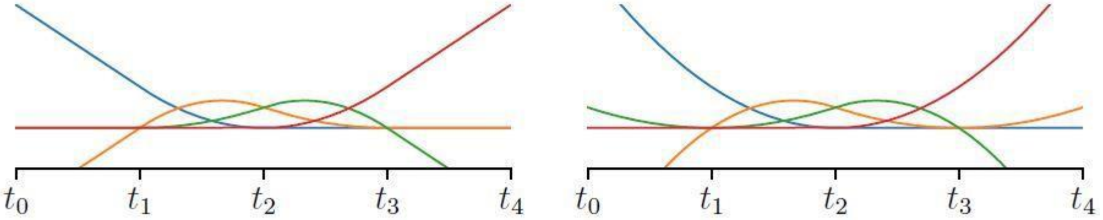

In general, using linear head and/or tail pieces to extrapolate outside the original domain or interpolate in the data sparse region is far more stable that using higher order polynomials, see figure B. The figure shows symmetric linear tail modifications, but for the analyses in the paper we only impose a right linear tail shape constraint.

Posterior Variance Estimation

To obtain posterior uncertainty, we use a parametric bootstrap.[43] Once we solve (9) to obtain estimates $\hat{\beta}$and $\hat{\gamma}$ we have a model distribution of the errors (1):

$$\boldsymbol{y}_i = \mathbf{F}_i(\hat{\beta}) + \mathbf{Z}_i \boldsymbol{u}_i + \boldsymbol{\epsilon}_i$$

Achoki T, *et al. J Epidemiol Community Health* 2022; 76:471–481. doi: 10.1136/jech-2021-217480

We sample datasets from this distribution to generate full data sets $\{\mathbf{Y}\}^j$, for $j = 1, \ldots, N$. For each dataset $\mathbf{Y}^j$, we then re-solve the fitting problem (9) to obtain estimates $\hat{\beta}^j$ and $\hat{\gamma}^j$, and the set $\{\hat{\beta}^j, \hat{\gamma}^j\}$ over all j allows us to estimate any posterior statistic we need.
In particular, the posterior set of dose-response curves is given by

$$\{f(t)^j + u_0^j\}$$

where $f(t)^j$ is the curve obtained by using the re-fit value $\hat{\beta}^j$, and u_0^j is a sample from $N(0, \hat{\gamma}_0^j)$, the associated unexplained heterogeneity parameter.

<u>Bias adjustment for alternative case definitions and study methods</u>

In GBD 2019, we decided to do all our adjustments of non-fatal and risk exposure data to deal with alternative case definitions or study methods prior to entering data into our main analytical tools of DisMod-MR 2.1 and ST-GPR. This decision also included the adjustment of data presented for both sexes to a male and female equivalent. The starting point was to explicitly state the reference case definition and study method and identify alternative definitions and study characteristics that fall within our inclusion criteria.
We compiled data from both within-study comparisons (ie, data that used alternative and reference definitions in the same population) and between-study comparisons (ie, data that used an alternative definition in one population and a reference definition in another population that overlap in location, time, age, and sex) of different case definitions. For between-study comparisons, we allowed a maximum calendar year difference between studies of five years. Where validation studies (ie, those carried out at the introduction of a new set of diagnostic criteria comparing to previous criteria) were available, we extracted data on the comparison of alternative to reference. For quantities of interest with multiple alternative definitions/methods we also look for pairs comparing two alternatives. In a network analysis, if A is the reference and B and C are two alternatives, a comparison of A vs B and B vs C provides an indirect comparison of the alternative C against the reference A.

We pooled either the logit difference between alternative and reference or the natural log of the ratio of alternative to reference. From simulations we found that the two methods provide almost identical results for quantities that after adjustment do not exceed a value of 0.5 (eg, prevalence or proportion). The logit difference method much better dealt with higher values and avoided prevalence or proportions to exceed one. If the values of either the reference or alternative were zero, we aggregated values across age groups until both values had non-zero observations. We used the delta method to compute the standard error of the reference and alternative measures in logit space. The standard error of the logit difference was computed as the square root of the sum of the variances of each data point in a pair.

Age-sex splitting

Age-sex splitting was commonly applied to literature data reported by age or sex but not by age and sex. For GBD 2019, we split all data reported in age groups with a width greater than 20 years, and we did so by using age patterns from available survey microdata or regional patterns derived from an initial run of the main modelling tool, DisMod-MR 2.1.

Data analysis

We used a network random effects meta-regression in MR-BRT. In a network analysis, if A is the reference and B and C are two alternatives, a comparison of A vs B and B vs C provides an indirect comparison of the alternative C against the reference A. To implement the network we included dummy variables with a particular structure. This was implemented as follows, where A is the reference definition/method:

- Create *k* dummy variables where *k* are all definitions/methods other than A (eg, *k* = B, C)
- Code dummy *k* as
  - 1 if the first term of the logit difference is *k*;
  - -1 if *k* is second term of the logit difference;
  - 0 otherwise

For example:

Study	Comparison	DummyB	DummyC
1	logit(B)-logit(A)	1	0
2	logit(B)-logit(A)	1	0
3	logit(C)-logit(A)	0	1
4	logit(C)-logit(A)	0	1
5	logit(C)-logit(B)	-1	1
6	logit(C)-logit(B)	-1	1

The coding structure outlined above in step 1 assumes that all case definitions are mutually exclusive. In some cases, however, individual case definitions are a function of different components or dimensions. For example, case definitions may vary by the type of symptoms that a respondent experiences as well as the recall period over which those symptoms are experienced. In the presence of sparse data, it may be difficult to find both direct and indirect comparisons of all individual case definitions. In these case, an alternative approach is to assume different dimensions of case definitions have a multiplicative effect. In other words, the effect of recall period has the same relative effect across different categories of symptoms reported by respondents. To implement this coding scheme:

- Create *k* dummy variable columns for each case definition dimension
- For each dummy variable k:
  - Add 1 if *k* is a component of the first term in the logit difference
  - Subtract 1 if *k* is a component of the second term in the logit difference

In MR-BRT, we ran random effects meta-regression of the logit difference (or log ratio) with all the *k* dummy variables as covariates, omitting the intercept in the meta-regression. We used a study_id variable for the unique identifier of the reference and alternative studies (or alternative1 to alternative2). The coefficients on the *k* dummy variables represent the pooled logit difference of the *k* alternative definition to the reference taking into account evidence from both direct and indirect comparisons. In the example above, the coefficient on DummyA is the pooled logit difference of B minus A; the coefficient on DummyB is the pooled logit difference of C minus A. The standard error of the pooled logit difference incorporating the between study variance was calculated as:

$$se(\text{logit}(difference_k)) = \sqrt{var_k + \gamma^2}$$

Where:

$se(\text{logit}(difference_k)$ is the standard error of the pooled logit difference of alternative *k* to the reference

var_k is the variance of the coefficient on dummy variable *k*

γ^2 is the between-study variance

If both between and within study pairs were available, we examined whether there was a systematic difference between these. If there was a significant difference, we made judgement call as to whether within-study or between study data comparisons were most appropriate. In general, this was the within-study data, however, there were important measurement or conceptual reasons for choosing between-study data. For example, for crosswalks between self-reported height and weight compared to measured height and weight, between-study comparisons may be preferable if respondents knew they would be measured and, therefore, were less likely to misreport their height and weight.
We also examined whether there were systematic differences in the adjustments by key demographics (age, sex, geographic location, year) and other potential factors that may lead to variation in crosswalks. This could only be done at present in a direct comparison model and not in a network. We did this when there was a strong rationale, eg, biological plausibility, for variation by such characteristics.

After obtaining the pooled logit difference or log ratio estimates, we predicted adjustments based on the statistical model, including uncertainty in the adjustment and sampling error of each data point. For non-significant logit differences or log ratios we still applied the adjustments if there was a conceptual reason to believe that the alternative definition is biased. This expands the variance of these alternative definition data points.

Interpreting the coefficients of a logit difference model is not so straightforward as the adjustment to alternative data points is dependent of its value. For instance, the figure below on the left, shows the MR-BRT fit using a spline function by age to the logit differences of all overlapping pairs. The graph on the right indicates the adjustment by age for a hypothetical data point of 5%. The larger logit difference at younger ages, and to a lesser extent older ages, leads to a greater downward (in this case) adjustment of the 5% data point than at the mid age range.

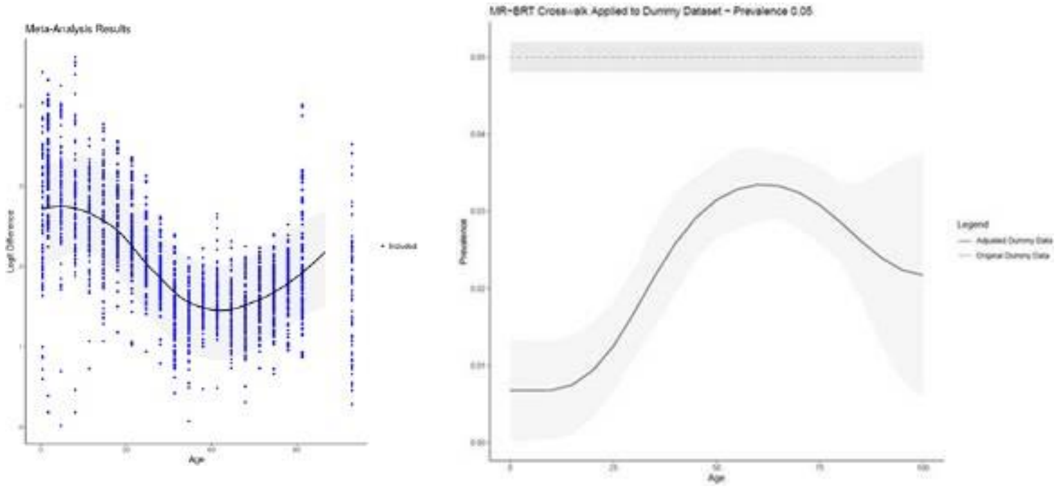

<u>DisMod-MR 2.1 estimation</u>[3]

Estimation of sequelae and causes

The most extensively used estimation method is the Bayesian meta-regression method DisMod-MR 2.1. For some causes such as HIV/AIDS or measles, disease-specific natural history models have been used for which the underlying three state model in DisMod-MR 2.1 (susceptible, cases, dead) is insufficient to capture the complexity of a disease process. For some diseases with a range of sequelae differentiated by severity, such as COPD or diabetes mellitus, DisMod-MR 2.1 was used to meta-analyse the data on overall prevalence with separate DisMod-MR 2.1 models of the proportions of cases with different severity levels or sequelae. Likewise, DisMod-MR 2.1 was used to meta-analyse data on the proportions of liver cancer and cirrhosis due to underlying aetiologies such as hepatitis B, hepatitis C, and alcohol use.

DisMod-MR 2.1 description

Until GBD 2010, non-fatal estimates in burden of disease assessments were based on a single data source on prevalence, incidence, remission, or a mortality risk selected by the researcher as most relevant to a particular location and time. For GBD 2010, we set a more ambitious goal: to evaluate all available information on a disease that passes a minimum quality standard. That required a different analytical tool that would be able to pool disparate information presented for varying age groupings and from data sources by using different methods. The DisMod-MR 1.0 tool used in GBD 2010 evaluated and pooled all available data, adjusted data for systematic bias associated with methods that varied from the reference, and produced estimates by world regions with UIs by using Bayesian statistical methods. For GBD 2013, the improved DisMod-MR 2.0 increased computational speed, which allowed computations to be consistent between all disease parameters at the country rather than the region level. The hundred-fold increase in speed of DisMod-MR 2.0 was partly

due to a more efficient rewrite of the code in C++ but also to changing to a model specification by using log rates rather than a negative binomial model used in DisMod-MR 1.0. In cross-validation tests, the log rates specification worked as well or better than the negative binomial specification.[33] The sequence of estimation occurs at five levels: global, super-region, region, country and, where applicable, subnational location. The super-region priors are generated at the global level with mixed-effects, nonlinear regression by using all available data; the super-region fit, in turn, informs the region fit, and so on down the cascade. The wrapper gives analysts the choice to branch the cascade in terms of time and sex at different levels depending on data density. The default used in most models is to branch by sex after the global fit but to retain all years of data until the lowest level in the cascade is reached.

The computational engine is limited to three levels of random effects; we differentiate estimates at the super-region, region and country level. In GBD 2013, the subnational units of China, the UK and Mexico were treated as “countries” to enable a random effect to be estimated for every location with contributing data. However, the lack of a hierarchy between country and subnational units meant that the fit to country data contributed as much to the estimation of a subnational unit as the fits for all other countries in the region. We found inconsistency between the country fit and the aggregation of subnational estimates when the country’s epidemiology varied from the average of the region. Adding an additional level of random effects required a prohibitively comprehensive rewrite of the underlying DisMod-MR engine. Instead, we added a fifth layer to the cascade, with subnational estimation informed by the country fit and country covariates, plus an adjustment based on the average of the residuals between the subnational location’s available data and its prior. This technique mimicked the impact of a random effect on estimates between subnationals.

In GBD 2015, we also improved how country covariates differentiate non-fatal estimates for diseases with sparse data. The coefficients for country covariates are re-estimated at each level of the cascade. For a given location, country coefficients are calculated by using both data and prior information available for that location. In the absence of data, the coefficient of its parent location is used to utilise the predictive power of our covariates in data-sparse situations.
For GBD 2016, the computational engine (DisMod-MR 2.1) remained substantively unchanged from GBD 2015. We changed the prediction year set to generate fits for the years 1990, 1995, 2000, 2005, 2010, and 2016. We updated the age prediction sets to include age groups 80–84 years, 85–89 years, 90–94 years, and 95 years and older to comply with changes across all functional areas of the GBD. We also expanded the set of locations where subnational units are modelled; the set now includes Brazil, China, England, India, Indonesia, Japan, Kenya, Mexico, South Africa, Sweden, and the US.

In GBD 2017, we continued to use DisMod-MR 2.1 because no substantial changes were made. Updates to computation include extending the terminal prediction year to 2017 and additional subnational units in Ethiopia, Iran, New Zealand, Norway, and Russia. Saudi Arabia was also modelled only at the national level in 2017.

In GBD 2019, no substantial changes were made to DisMod-MR 2.1 but we made more substantial changes to how we use the tool. First, we added the year 2019 as an additional year of estimation. Second, we also included the option again to have random effects on cause-specific mortality rates (CSMR) and EMR This functionality had been dropped a couple of GBD rounds earlier. Third, as we did all our adjustments for alternative case definition and study methods as well as adjustments to both sex data points prior to entering data into DisMod-MR 2.1, we no longer used the functionality in DisMod-MR 2.1 to estimate coefficients for study covariates.

Fourth, based on simulation testing we found that coverage improved and errors reduced when passing down priors with a wider setting of minimum coefficient of variation (which determines the uncertainty around priors and hence how ‘informative’ the priors are) than had generally been used in past GBD iterations. We settled on a default value of 0.8 where in the past values of 0.4 or less had been more commonly used. We made some exceptions for high prevalent conditions where a lower minimum coefficient of variation (CV) setting achieved the task of making priors less informative but not completely uninformative.

We carried out simulation testing using DisMod-MR 2.1 based on an internally consistent set of 15,601 data points for

prevalence, incidence, excess mortality, CSMR, and remission. The dataset was generated by the simulation capability of the DisMod-AT tool that is under development. We aimed to test what level of minimum CV would create the best fit based on the following three performance statistics:

(1) Coverage, ie, the proportion of data point mean values that fall between the 2.5th and 97.5th percentile of the draws of the fit values;
(2) Root mean square error: the square root of the mean of the squares of the difference between data point mean values and the mean fit value; and
(3) Bias: the difference between the mean fit value and the data point mean value.

We created different datasets culling the initial complete set with values at every age, sex, and location to more realistic data sparsity scenarios for analysis.

A first strategy was to randomly reduce the dataset to 10%, 5%, 2.5%, 1%, and 0.5% of the original data points. Initial results indicated little variation between the data samples culled to 10%, 5%, 2.5%, and 1%. The 0.5% culled dataset was an exception with markedly worse performance statistics, particularly with regard to bias and RMSE as illustrated in figure 1. We conducted further studies using the datasets culled to 10%, 5%, and 0.5%.

The second strategy was to compare randomly culled dataset for 10%, 5%, and 0.5% with datasets culled to the same percentages, but differentially by SDI, such that we culled all the data in sub-Saharan Africa and for the other super-regions based on the probability diminishing with increasing SDI. This pattern of differential data coverage by SDI is commonly observed in datasets used for modelling. The plots shown in figure 2, generally also show diminished performance for this more realistic scenario of differential sparseness by location based on SDI.

Figure 2. Performance statistics comparing randomly and differentially culled datasets.

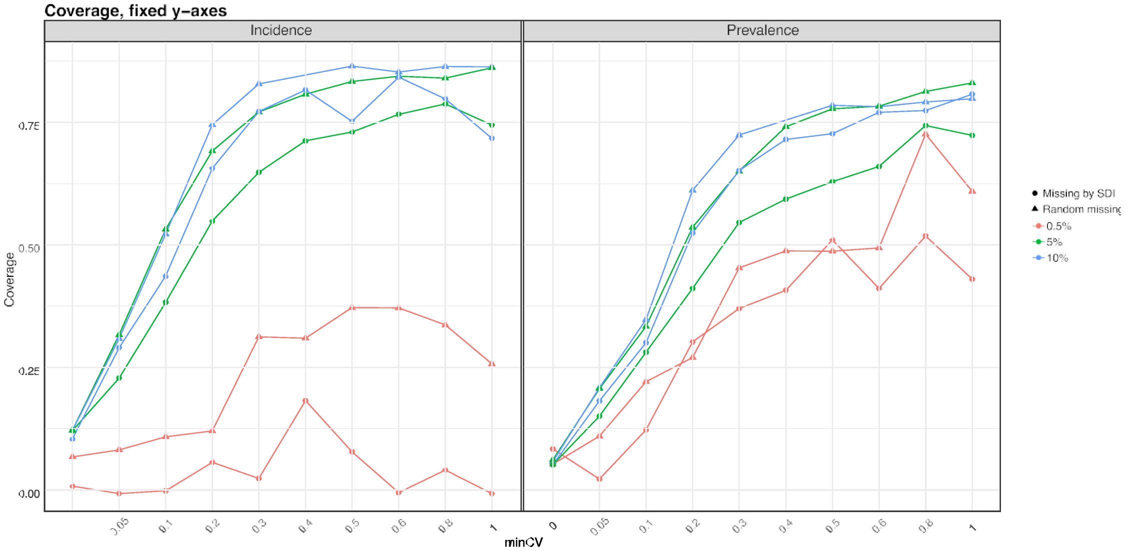

Achoki T, *et al. J Epidemiol Community Health* 2022; 76:471–481. doi: 10.1136/jech-2021-217480

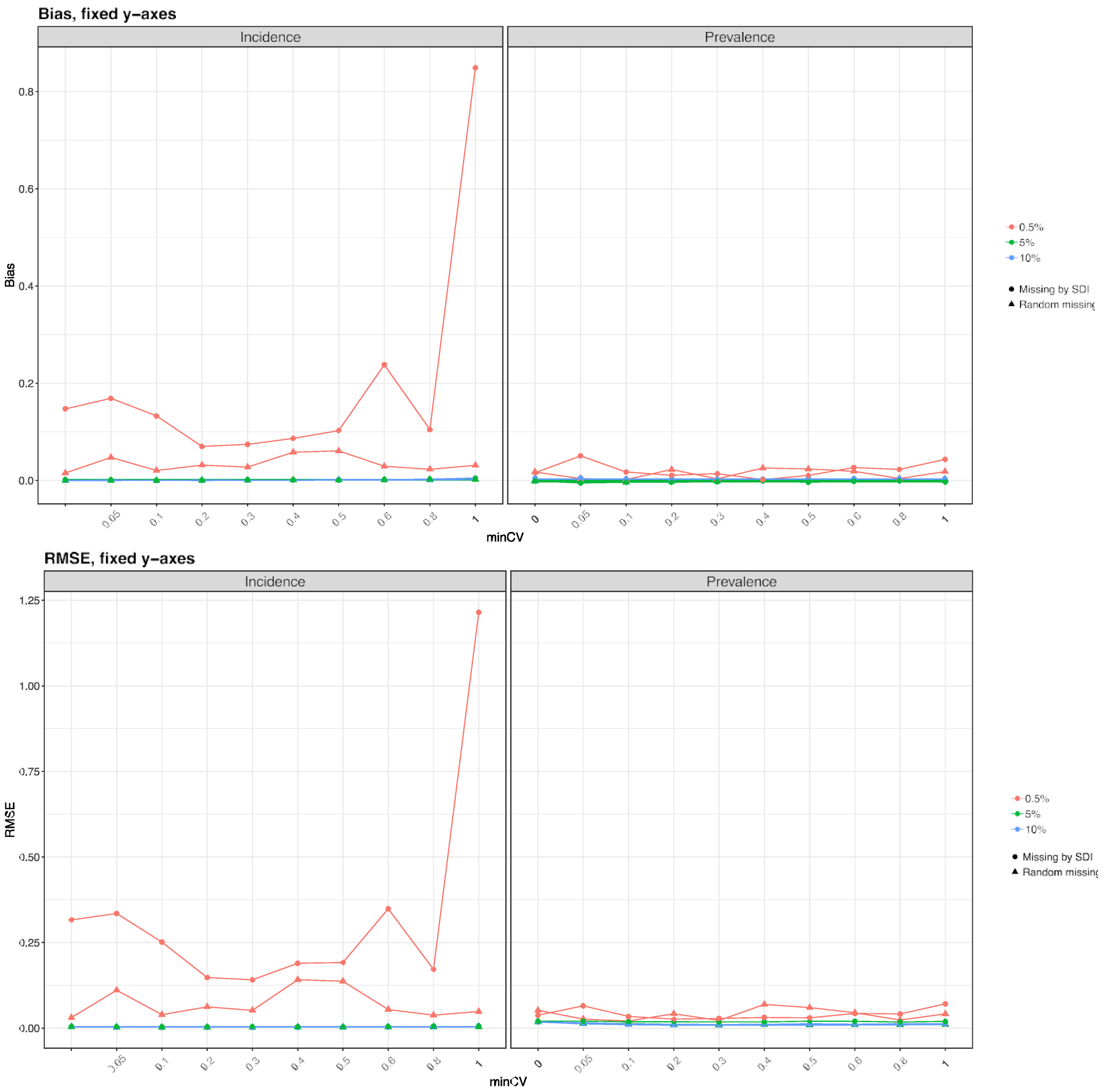

A third strategy was to apply a further distinction of complete culling of either prevalence and CSMR, or incidence data points, using the 10% randomly culled or 10% differentially culled datasets as comparators. In these scenarios, we found that the coverage statistic starts to level off at a value of 0.8 for minimum CV. All three metrics are much worse for datasets with incidence data culled. Performance statistics for this strategy are shown in figure 3.

Figure 3. Performance statistics comparing datasets with specific measures held out vs. randomly or differentially culled datasets.

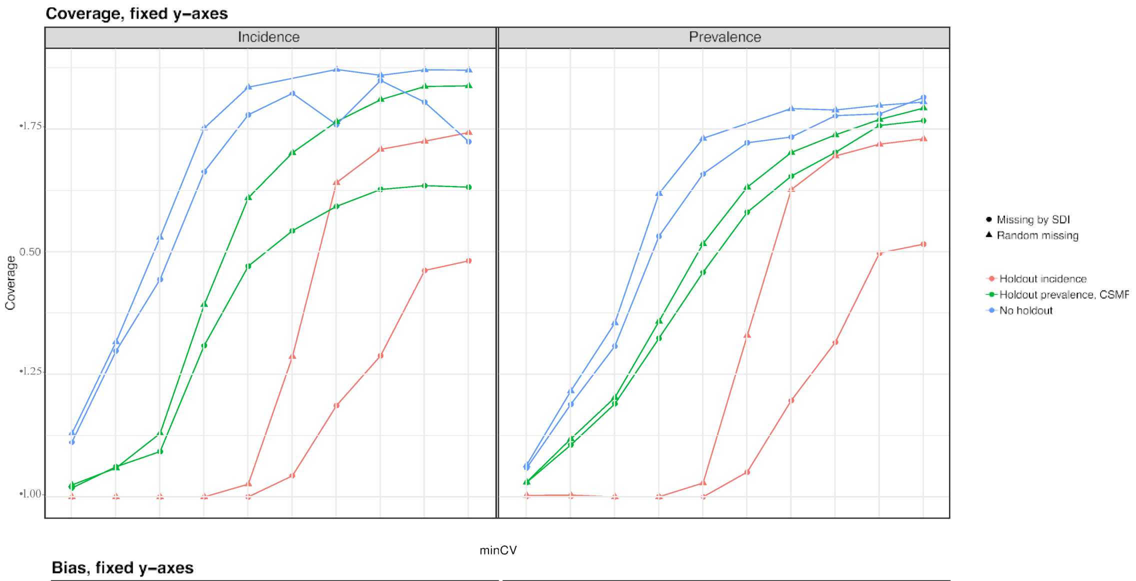

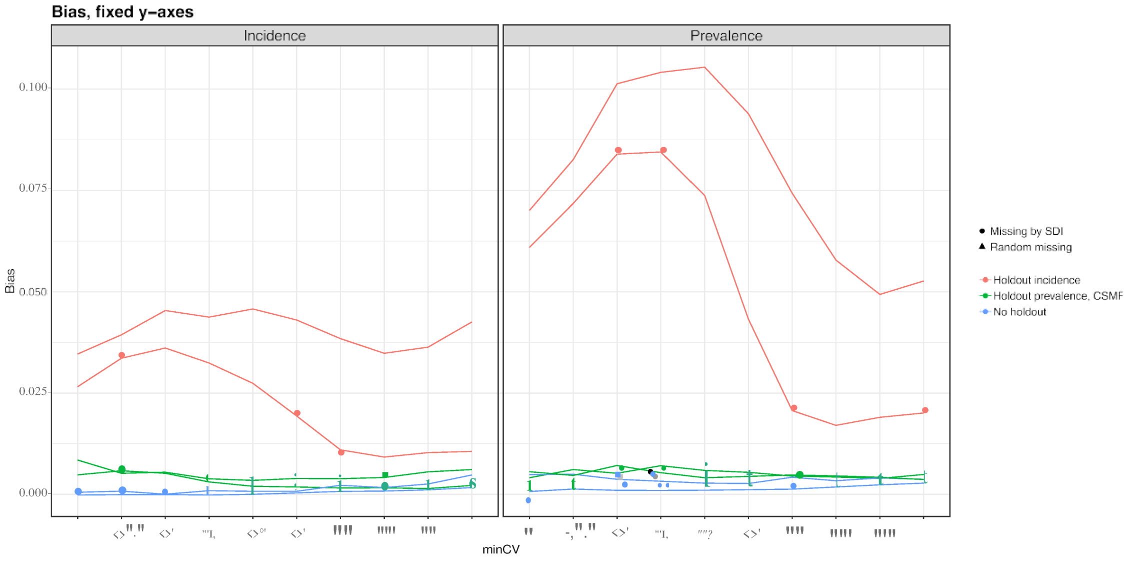

Achoki T, *et al. J Epidemiol Community Health* 2022; 76:471–481. doi: 10.1136/jech-2021-217480

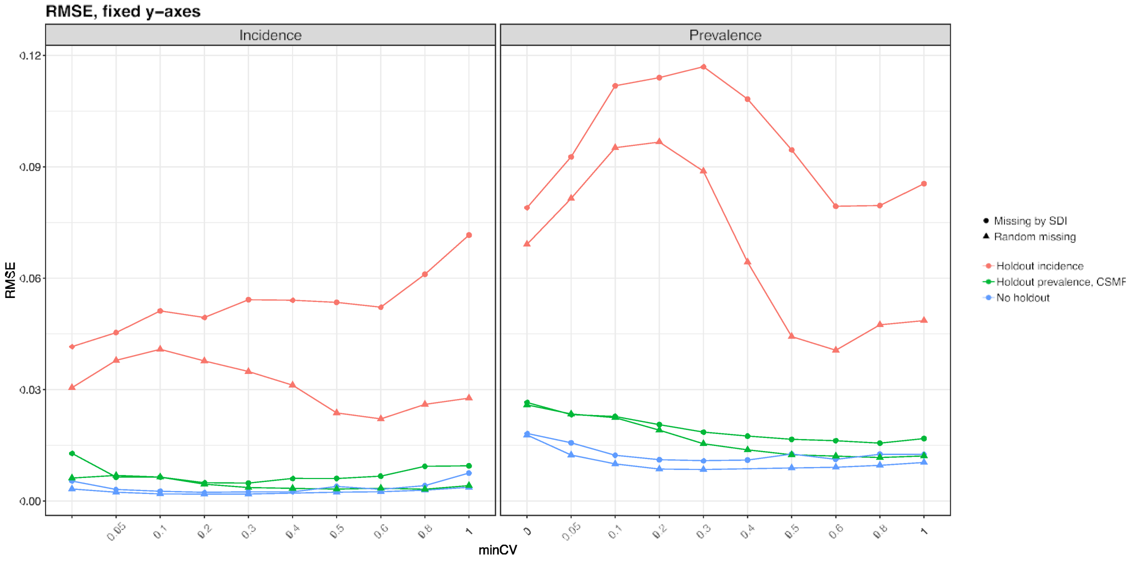

Fifth, we changed our approach to estimating excess mortality rates, the key link in the model between cause-specific mortality rates (CSMR) and incidence and prevalence. In the past two GBD rounds we calculated priors on excess mortality and entered these as data points by matching sex-specific prevalence data with an age width of 20 or less with the corresponding CSMR for the same location and year. For stability sake, we excluded calculation of EMR for prevalence data points of less than 1 in a million. EMR is simply calculated as CSMR divided by prevalence. As with previous GBD years, for diseases with an average duration of less than a year (as indicated by a setting of remission greater than one), we ran an initial global model to get an equivalent prevalence and used the following formula to calculate EMR:

$$EMR = {}^{CSMR * (remission + (ACMR - CSMR) + EMR\_pred)}/_{incidence}$$

where,

ASMR is the all-cause mortality rate

EMR_pred is the EMR fit from an initial global DisMod model

Despite using the log of LDI or the HAQ Index as a covariate with a prior that the coefficient had to be negative, we found many disease models with an implausible distribution of mortality to prevalence (or incidence) ratios implying lower case fatality in locations with lower HAQ Index than in countries with higher HAQ Index. This likely signals an inconsistency between fatal and non-fatal data inputs. For GBD 2019, we decided to run regressions on EMR data (calculated as described above) first using MR-BRT with HAQ Index as a predictor. In general, we tend to think that CSMR estimates are more robust than non-fatal data because of much greater data availability and a lesser task in adjusting cause death data for garbage coding than the complex task of adjusting non-fatal data sources for alternative case definitions and study methods. To indicate that we would reduce the random effects on EMR and the minimum coefficient of variation for priors on EMR being created at each next level down the cascade. However, there were exceptions. For drug use disorders, the risk of overdose deaths is less a function of a country’s quality of health services but driven more by the availability of harm reduction strategies such as opioid substitution therapy and the availability of highly potent opioids such as fentanyl, which have been an important contributor to the large increase in overdose deaths in the USA in the last decade. We settled on a model for opioid use disorder with wider random effects and higher minimum coefficient of variation to give less emphasis on CSMR when enforcing consistency with prevalence data. In a next round, we will work to find covariates that are more relevant to drug overdose deaths such as a grading of harm reduction strategies by country and over time. In the case of COPD, we noted that following the data on CSMR and EMR led to large increases in prevalence estimates in east Asia,

Achoki T, *et al. J Epidemiol Community Health* 2022; 76:471–481. doi: 10.1136/jech-2021-217480

Oceania and, to a lesser extent, south Asia. In the oldest age groups, prevalence estimates would be higher than the prevalence data for these locations and reach a level of close to 80% in the oldest age groups. In these locations, we will pay attention to how garbage codes are being redistributed onto COPD in the next round of GBD.

DisMod-MR 2.1 likelihood estimation

Analysts have the choice of using a Gaussian, log-Gaussian, Laplace, or Log-Laplace likelihood function in DisMod-MR 2.1. The default log-Gaussian equation for the data likelihood is

$$-log[p(y_j|\Phi)] = \log(\sqrt{2\pi}) + \log(\delta_j + s_j) + \frac{1}{2}\left(\frac{\log(a_j + \eta_j) - \log(m_j + \eta_j)}{\delta_j + s_j}\right)^2$$

Where,

y_j is a "measurement value" (ie, data point)

Φ denotes all model random variables

η_j is the offset value, *eta*, for a particular "integrand" (prevalence, incidence, remission, excess mortality rate, with-condition mortality rate, cause-specific mortality rate, relative risk, or standardised mortality ratio)

a_j is the adjusted measurement for data point j, defined by

$$a_j = e^{(-u_j - c_j)} y_j$$

Where:

u_j is the total "area effect" (ie, the sum of the random effects at three levels of the cascade: super-region, region and country) and

c_j is the total covariate effect (ie, the mean combined fixed effects for sex, study level, and country level covariates), defined by

$$c_j = \sum_{k=0}^{K[I(j)]-1} \beta_{I(j),k} \hat{X}_{k,j}$$

with SD

$$s_j = \sum_{l=0}^{L[I(j)]-1} \zeta_{I(j),l} \hat{Z}_{k,j}$$

Where:

k denotes the mean value of each data point in relation to a covariate (also called x-covariate)

$I(j)$ denotes a data point for a particular integrand, j

$\beta_{I(j),k}$ is the multiplier of the kth x-covariate for the ith integrand

$\hat{X}_{k,j}$ is the covariate value corresponding to the data point j for covariate k;

l denotes the SD of each data point in relation to a covariate (also called z-covariate)

$\zeta_{I(j),k}$ is the multiplier of the lth z-covariate for the ith integrand

δ_j is the SD for adjusted measurement j, defined by:

$$\delta_j = log[y_j + e^{(-u_j - c_j)}\eta_j + c_j] - log[y_j + e^{(-u_j - c_j)}\eta_j]$$

Where:

m_j denotes the model for the jth measurement, not counting effects or measurement noise, and defined by:

$$m_j = \frac{1}{B(j) - A(j)} \int_{A(j)}^{B(j)} I_j(a)\, da$$

Where:

$A(j)$ is the lower bound of the age range for a data point

$B(j)$ is the upper bound of the age range for a data point

I_j denotes the function of age corresponding to the integrand for data point j

<u>Impairment and underlying cause estimation</u>[3]

For GBD 2019, as in GBD 2017 and GBD 2016, we estimated the country-age-sex-year prevalence of nine impairments. Impairments in GBD are conditions or specific domains of functional health loss that are spread across many GBD causes

as sequelae and for which there are better data to estimate the occurrence of the overall impairment than for each sequela based on the underlying cause. These impairments included anaemia, epilepsy, hearing loss, heart failure, intellectual disability, infertility, vision loss, Guillain-Barré syndrome, and pelvic inflammatory disease. Overall impairment prevalence was estimated by using DisMod-MR 2.1. We constrained cause-specific estimates of impairments, as in the 19 causes of blindness, to sum to the total prevalence estimated for that impairment. Anaemia, epilepsy, hearing loss, heart failure, and intellectual disability were estimated at different levels of severity. Estimates were made separately for primary infertility (those unable to conceive), secondary infertility (those having trouble conceiving again), and whether the impairment affected men and/or women. In the case of epilepsy, we determined the proportions with idiopathic and secondary epilepsy as well as the proportions with severe and less severe epilepsy by using mixed effects regressions. The sparse data for the proportion of seizure-free, treated epilepsy were pooled in a random effects meta-analysis. DisMod-MR 2.1 models produced country-, age-, sex-, and year-specific severity levels of hearing loss and vision loss. Because of limited information on the severity levels of intellectual disability, we assumed a similar distribution of severity globally based on random effects meta-analysis of IQ-specific data for the overall impairment. This assumption was supplemented by cause-specific severity distributions for chromosomal causes and iodine deficiency; the severity of intellectual disability included in the long-term sequelae of causes including neonatal disorders, meningitis, encephalitis, neonatal tetanus, and malaria was estimated in combined health states of multiple impairments such as motor impairment, blindness, and/or seizures.[44] We changed the name of the intellectual disability impairment to specify that estimates reflect cases arising during the developmental period, which we have defined as ages under 20 years. The severity of heart failure was derived from our Medical Expenditure Panel Surveys (MEPS) analysis and therefore was not specific for country, year, age, or sex.

Impairment squeeze

For impairments like epilepsy, intellectual disability, and blindness, mentioned above in Step 4, we often have better information regarding the total prevalence of the impairment rather than the prevalence of said impairment due to its various causes. For example, we have more data and a better idea of the total number of blind individuals (which we refer to herein as the blindness "envelope") in the world than we do the number of individuals who are blind due to a specific cause like retinopathy of prematurity or cataract. We achieve this consistency by either squeezing or inflating the individual sequela prevalence values so that their sums fit into each appropriate envelope. Blindness, epilepsy, and/or intellectual disability appear in various combinations with motor impairment levels as sequelae for a number of neonatal disorders and infectious diseases like malaria and neonatal tetanus ("Moderate motor impairment with blindness and epilepsy due to neonatal tetanus", for example). This presents an extra challenge because any squeeze or inflation of one of the impairments making up a sequela affects the others.

We set some rules on how to do these adjustments sequentially. First, when the envelope of an impairment is smaller than the sum of all contributing causes, we redistribute the excess prevalent cases of combined impairment sequelae onto the sequelae that only have motor impairment (at a mild, moderate, or severe level) within the same cause grouping. Second, we apply the adjustments in a particular order such that we always fit at least one of the envelopes exactly where the other one or two envelopes may be exceeded by some amount. We first enforce a fit to the epilepsy impairment envelope, then intellectual disability, and last, blindness. Thus, the epilepsy envelope always matches exactly, whereas the intellectual disability and blindness envelopes may occasionally be exceeded on a draw-by-draw basis.

Severity distribution[3]

Sequelae were defined in terms of severity for 169 causes. We generally followed the same approach for estimating the distribution of severity we used in GBD 2017. In cases in which severity was related to a particular impairment, such as mild, moderate, and severe heart failure due to ischaemic heart disease or the newly added cause of pulmonary arterial hypertension, the analysis was driven by impairment estimation methods. Severity levels for causes such as chronic kidney disease, epilepsy and COPD were modelled using DisMod-MR 2.1 or ST-GPR, whereas we performed meta-analyses to estimate the allocation of severity for causes such as rheumatoid arthritis, and multiple sclerosis. For dementia, we changed from using meta-analysis of three age categories to a more flexible model in MR-BRT using a spline on age. That allowed us to

increase the number of studies informing severity from 7 to 67. For gallbladder and biliary diseases, we performed a meta-analysis of six community-based studies of the proportion of cases of gallbladder disease identified by ultrasonography who are symptomatic. In previous rounds, inpatient admission for gall bladder and biliary disease as a primary diagnosis were taken to represent symptomatic cases. For the new cancer sites included in GBD 2019, we used the same strategy as for all other cancer sites. For the newly added sites of osteoarthritis of the hand and sites other than hip or knee, we assumed the same severity distribution as for osteoarthritis of the knee.

For many causes, we continue to have inadequate data on severity from surveys or the epidemiological literature. For those diseases, we made use of three population surveys: the MEPS 2000–2014, the [US] National Epidemiological Survey on Alcohol and Related Conditions (NESARC) 2000–2001 and 2004–2005, and the Australian National Survey of Mental Health and Wellbeing of Adults (NSMHWB) 1997.[45–47] Each dataset contained individual-level measurements of functional health status made by using the 12-Item Short Form Health Survey (SF-12) as well as diagnostic information on the causes affecting each individual.

To use the data collected by measuring the distribution of severity with the SF-12, the individual SF-12 summary scores were mapped to an equivalent DW. A convenience sample of respondents was asked to complete SF-12 for the hypothetical individual living in a health state described by using a selection of 60 of the 235 health states with their lay descriptions from the GBD DW surveys reflecting the full range of severity. Each of these health states has a measured DW associated with it on a zero to one scale. We collected 1980 usable responses in total. To deal with heterogeneity in responses, we excluded from the statistical analysis responses that were more than two median absolute deviations from the median for each health state. After correcting for outliers, the rank order correlation between SF-12 scores for the hypothetical individuals in each health state characterised by the lay description with the measured DW was -0.815. The health states served as random effect groups such that the composite score would be equal to the intercept plus the random effect estimated for that health state, or

$$DW_i = \alpha + U_{health\ state}$$

The final relationship between SF-12 score and DW is depicted in figure A:

Achoki T, et al. J Epidemiol Community Health 2022; 76:471–481. doi: 10.1136/jech-2021-217480

Figure A. SF-12 composite scores and disability weights for 60 health states with fitted loess regression

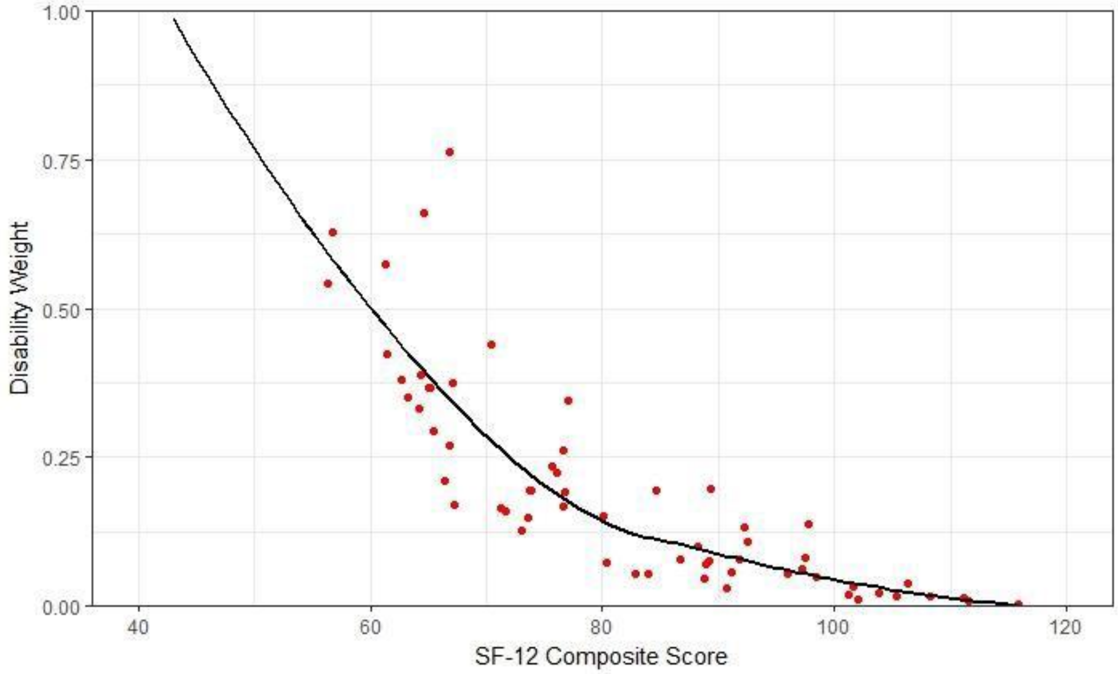

To generate a smooth mapping from SF-12 combined scores to the GBD DW space, we used locally estimated scatterplot smoothing regression on the random effects for each health state. Because DWs are defined in the range from 0 to 1, we truncated the function at a combined SF-12 score of 116.36 (any combined score above this level was set to 0) and truncated the function at 42.7 so that any combined score less than that value was set to 1. All SF-12 survey data were thus transformed into DW space.

The second stage of the analysis was to build models predicting the transformed SF-12 scores as a function of the number of causes suffered by each individual. First, variable selection was performed by using least absolute shrinkage and selection operator (LASSO) regression to penalize the regression coefficients of highly correlated causes. The tuning parameter, λ, controls the strength of the least-squares penalty. When λ=0, LASSO regression returns the same results as ordinary least-squares regression. Higher values of λ impose a stronger penalty and constrain a greater number of model parameters to 0. A ten-fold cross-validation was used to find the value of the λ that minimized the mean cross-validated error. This process resulted in a λ value of 0.0013 and eliminated 10 causes from the analysis. Transformed SF-12 scores into the DW scale for the remaining 190 causes were then modelled for each measure *m* of each individual *i* over *n* total causes in the survey as follows:

$$logit(DW)_{im} = \beta_0 + \beta_1 Condition1_{im} + \cdots + \beta_n Condition\ n_{im}$$

This equation effectively assumes that comorbid causes act to change SF-12 scores in a multiplicative fashion rather than an additive fashion.

To estimate the comorbidity-corrected effect of each cause (ie, in isolation) on total disability, we compared the predicted DW without the cause of interest (counterfactual DW) with the predicted DW including the cause of interest. Following the multiplicative comorbidity equation, the joint effect can be written

$$Condition\ specificDW = 1 - \frac{1 - predictedDW_m}{1 - counterfactualDW_m}$$

Achoki T, *et al. J Epidemiol Community Health* 2022; 76:471–481. doi: 10.1136/jech-2021-217480

The mean of this cause-specific effect over all observations is the population marginal effect of a cause.

Using the model above, we estimate a counterfactual DW – the total individual DW excluding the effect of the cause of interest. We compared the observed distribution of functional health status with this counterfactual distribution to determine the marginal effect of the cause of interest. In other words, we estimated the health state for each individual and for each cause as the cumulative individual weight minus the effects of all comorbid causes.

$$Health\ state\ DW = 1 - \frac{1 - individual\ cumulativeDW_m}{1 - counterfactualDW_m}$$

The estimation strategy for health state-specific severity distributions for which there are multiple severity categories involved binning individuals' weights into severity cut-offs (eg, mild, moderate, and severe) for which DWs were derived. These bins were defined by using results from the GBD Disability Weights Studies[48] for causes that had multiple health states defined. Cut-offs were taken as the midpoints between levels of health state and cases distributed into severity bins accordingly. Cases were considered asymptomatic if the counterfactual weight was equal to or greater than the individual cumulative weight.

Disability weights[3]

To compute YLDs for a particular health outcome in a given population, the number of people living with that outcome is multiplied by a DW that represents the magnitude of health loss associated with the outcome. DWs are measured on a scale from 0 to 1; 0 implies a state equivalent to full health, and 1, a state equivalent to death.

DWs used in GBD studies before GBD 2010 have been criticized for the method used (ie, person trade-off), the small elite panel of international public health experts who determined the weights, and the lack of consistency over time as the GBD cause list expanded and additional DWs from a study in the Netherlands[49] were added or others were derived by ad-hoc methods.

GBD 2010 disability weights measurement study

For GBD 2010, a primary data collection effort focused on measuring health loss rather than welfare loss by using a standardised approach of simple comparison questions directed to the general public across diverse communities.

Multi-country household surveys were conducted between Oct 28, 2009 and June 23, 2010 in five countries (Bangladesh, Indonesia, Peru, Tanzania, and the USA) selected to provide diversity across culture, language, and socioeconomic status.

Personal face-to-face computer-assisted interviews were conducted for all household surveys except for the survey in the US, which was conducted by computer-assisted telephone interview. Households were randomly selected by using a multistage stratified sampling design for which the probability of selection was proportional to the population size. In all cases, samples were designed to be representative of a given geographical area and, in the USA, to provide national representation.

For every contacted household, an adult respondent age 18 years or older was randomly selected by the survey program by means of the Kish approach. For face-to-face interviews, as many as three visits were made to selected households to establish contact. When a respondent was identified, as many as three return visits were made to do the survey at a time when the respondent was available. For the US telephone surveys, repeated calls were made up to seven times.

A web-based survey was posted at a dedicated URL between July 26, 2010 and May 16, 2011. The survey was initially available in English and subsequently available in Spanish and Mandarin. Recruitment of respondents occurred through several channels, such as news items and editorials in scientific journals, announcements at scientific meetings, postings on websites of institutions participating in the GBD, and social networking and communication mobilisation channels as well as direct contact with individuals and groups with known global health interests by tapping into the professional networks of the study investigators and their colleagues. Participants in the web-based survey were required to be ages 18 or older.

Achoki T, *et al. J Epidemiol Community Health* 2022; 76:471–481. doi: 10.1136/jech-2021-217480

Household surveys obtained oral informed consent from all participants; written informed consent was obtained from participants in the web survey. Ethical review board approval was obtained from each household survey site and the University of Washington, Seattle, WA.

Standardised survey instruments were developed to obtain comparative assessments of the full array of disease and injury sequelae, parsimoniously captured in 220 unique health states. Lay descriptions of health states formed the basis for all comparisons. These descriptions used simple, non-clinical vocabulary that emphasised the major functional consequences and symptoms associated with each health state. Development of these descriptions involved an iterative process of detailed consultation with experts participating in the GBD 2010 study; the goals was to capture the most relevant details of each health state while avoiding ambiguity and ensuring consistency. When possible, health states were grounded in standard clinical classifications systems. For example, the Canadian Cardiovascular Society grading scale was referenced for descriptions of stages of angina,[50] and the New York Heart Association functional classification was referenced for severity of heart failure.[51] Pilot testing indicated that the lay descriptions in face-to-face interviews should not exceed 30 words.

A paired comparison question formed the basis of all surveys. The questions in the survey were framed with the following statement, "A person's health may limit how well parts of his body or mind work. As a result, some people are not able to do all of the things in life that others may do, and some people are more severely limited than others. I am going to ask you a series of questions about different health problems. In each question, I will describe two different people…" Descriptions of two hypothetical people, each with a particular health state, were presented to respondents who were then asked which person they regarded as healthier. Health pairs in all surveys were selected by a randomizing computer algorithm. In the five household surveys, paired comparisons were presented for a subset of 108 health states pertaining to chronic conditions. The framing of chronic and acute conditions is different as they were presented as causing life-long or temporary health loss. We chose to only field health states that could be framed as lasting a lifetime in the household surveys as we hypothesized that presenting differently framed comparisons would be difficult to convey in face-to-face interviews. In the web survey, we considered this more feasible because respondents could read and refer to the framing of the question for each pair-wise comparison. All 220 health states were thus evaluated in the web survey.

In addition, the web survey included questions relating to population health and health programs specifically—such as "Imagine two different health programs. The first program prevented 1000 people from getting an illness that causes rapid death. The second program prevented 2000 people from getting an illness that is not fatal but causes lifelong health problems resulting in moderate to severe disability. Which program would you say produced the greater overall health benefits?" This information was used to anchor the results from the pair-wise comparisons on the 0–1 DW scale.

GBD 2013 European disability weights measurement study

The GBD 2010 DWs were critically dependent on the ways that outcomes were described to survey respondents. Descriptions for health states were designed to balance validity and parsimony, and this approach necessarily meant that some details of different health states had to be omitted. Because lay descriptions were developed collaboratively through individual expert groups organised around a particular set of health issues, some amount of variability in language and detail inevitably occurred. Criticisms and suggestions for improvement came from a number of commentators on the GBD 2010 DWs measurement study.[52–54]

GBD 2013 expanded the list of disease and injury causes and sequelae mapped to 235 unique health states. Additional data for the European Disability Weights Measurement Study were collected between September 23, 2013 and November 11, 2013 in Hungary, Italy, the Netherlands, and Sweden. The initiation of these surveys was connected to a project sponsored by the European Centre for Disease Prevention and Control (the Burden of Communicable Diseases in Europe project).[55] The four selected countries were chosen to be representative of the four regions of Europe (east, south, middle, and north) in terms of age, sex, and education of the respondents. Respondents were recruited from standing internet panels in each country on the basis of quota sampling with reference to age, sex, and education in such a way as to maintain the population representativeness of these characteristics. Eligible participants were 18–65 years old and were preselected in the Netherlands, where the age, sex, and education of respondents were already known, or in the other three countries, invited to participate via a web-link and then selected on the basis of their individual characteristics.

Achoki T, *et al. J Epidemiol Community Health* 2022; 76:471–481. doi: 10.1136/jech-2021-217480

The protocol for the European DWs measurement study followed the protocol that was developed and implemented in the GBD 2010 DWs measurement study. Lay descriptions for some health states that lacked mention of an important symptom or for which consistency of wording across different levels of severity had been noted were reworded. The European DWs measurement study included 255 health states, of which 183 were used in the analyses of GBD 2013. Those 183 consisted of 135 of the 220 health states that were included in the European DWs measurement study with unmodified lay descriptions and 30 from GBD 2010 for which alternative lay descriptions were included. DWs were estimated for additional sequelae that were incorporated into GBD 2013 but had not been included in GBD 2010.

Finding high correlation in resulting DW values between the country surveys and the web survey, we analysed the results of all surveys together. We ran probit regression analyses on the answers to the pair-wise comparison questions by using dummies for each health state with a value of 1 for the first state in a pair, –1 for the second state in a pair, and 0 for all states other than the pair. This method formalizes the intuition that if two health states in a pair produce similar health loss, the answers are likely to be evenly split; a pair of health states with very different health loss get many more responses favouring one over the other. The statistical methods infer the distances between values attached to different health states based on the frequencies of responses to the paired comparisons.

A second analytic step is needed to anchor the resulting estimates onto the 0–1 DWs scale. We anchored results from the probit regression analysis onto the 0–1 scale by using population health equivalence data from the GBD 2010 web survey by using a linear regression of the probit coefficients from the analysis of paired comparisons on the logit-transformed DW estimates derived from interval regression of the population health equivalence responses. Using numerical integration, we then estimated mean values for DWs on the natural 0–1 scale. Uncertainty was estimated by bootstrapping with 1000 samples.

A complete listing of the lay descriptions and values for the 440 health states (including combined health states) used in GBD 2019 is provided in appendix table 12.

Comorbidity correction (COMO)[3]

(52)The final stage in the estimation of YLDs is a micro-simulation, which adjusts for comorbidity. We refer to this micro-simulation process as “COMO” (for comorbidity correction). For GBD 2019, we estimated the co-occurrence of different diseases by simulating 40,000 individuals in each location-age-sex-year combination as exposed to the independent probability of having any of the sequelae included in GBD 2019 based on disease prevalence. We tested the contribution of dependent and independent comorbidity in the US MEPS data and found that independent comorbidity was the dominant factor even though well-known examples of dependent comorbidity exist, such as clustering of conditions like diabetes and stroke or anxiety and alcohol use disorders. Age was the main predictor of comorbidity such that age-specific micro-simulations accommodated most of the required comorbidity correction.[56]

The two components necessary for the computation of YLDs, prevalence of each disease sequelae and DWs, are the two inputs into COMO. The prevalence values are primarily produced by using DisMod-MR 2.1. The DWs have been described earlier in this appendix.

The micro-simulation, as performed for each age-sex-location-year, can best be represented as a four-step process. First, simulants are exposed to independent probabilities of having each sequela, where the probability is equal to the prevalence estimate. For each simulant, the probability of having a disease sequela is equal to the estimated prevalence from that draw from the uncertainty distribution. Each simulant is determined to have or not have the disease sequelae based on a draw from a binomial distribution. From this simulation, simulants end up having from no to multiple disease sequelae. Second, the DW for each simulant is estimated on the basis of the disease sequelae that they have acquired. The formula for the cumulative DW for a simulant is one minus the multiplicative sum of one minus each DW present

$$Simulant\ DW_l = 1 - \prod_{k=i}^{j}(1 - DW_k)$$

Achoki T, *et al. J Epidemiol Community Health* 2022; 76:471–481. doi: 10.1136/jech-2021-217480

Where:

DW_k is the DW for the kth disease sequela that the simulant *l* has acquired.

Once the simulant DW is computed, the DW attributable to each sequela for the simulant is calculated by using the following formula:

$$ADW_{lk} = \frac{DW_k}{\sum_{k=i}^{k=j} DW_k} * Simulant\ DW_l$$

Where:

ADW_{lk} is the attributable DW for disease sequela *k* in simulant *l*

DW_k is the DW for disease sequela *k*

Simulant DW_l is the DW for simulant *l* from the combination of all sequelae that they have acquired.

This formula apportions the overall simulant DW to each condition in proportion to the DW of each condition in isolation. Finally, YLDs per capita in an age-sex-country-year are computed by taking the sum of the attributable DWs for a disease sequela across simulants.

$$YLD\ Rate_k = \frac{\sum_{l=1}^{n} ADW_{lk}}{n}$$

The actual number of YLDs from disease sequela *k* in an age-sex-location-year is then computed as the YLD rate *k* times the appropriate age-sex-location-year population.

By repeating the simulation process for each age-sex-country-year 1000 times, the uncertainty in the prevalence of each disease sequela and the DW is propagated into the final comorbidity corrected YLD results. We selected 40,000 simulants for each age-sex-location-year group on the basis of simulation testing, which has shown that results are stable for YLDs at this number of simulants even in the younger age groups when prevalence is relatively low. Mean results for YLDs that reflect 40 million simulants (40,000 simulants multiplied by 1000 iterations to capture uncertainty) are very stable in each age-sex-location-year. For any given location-year-age-sex group, sequelae with a prevalence of less than one in 20,000 were excluded from the micro-simulation.

<u>YLD computation, uncertainty, and residual YLDs</u>[3]

For GBD 2019, we computed YLDs by sequela as prevalence multiplied by the DW for the health state associated with that sequela. The uncertainty ranges reported around YLDs incorporate uncertainty in prevalence and uncertainty in the DW. To do this, we take the 1000 samples of comorbidity-corrected YLDs and 1000 samples of the DW to generate 1000 samples of the YLD distribution. We assume no correlation in the uncertainty in prevalence and DWs. The 95% uncertainty interval is reported as the 25th and 975th values of the distribution. UIs for YLDs at different points in time (1990, 1995, 2000, 2005, 2010, and 2016) for a given disease or sequela are correlated because of the shared uncertainty in the DW. For this reason, changes in YLDs over time can be significant even if the UIs of the two estimates of YLDs largely overlap because significance is determined by the uncertainty around the prevalence estimates.

Section 2.6: Estimation Process for DALYs[4]

<u>Computing DALYs</u>

To estimate DALYs for GBD 2019, we started by estimating cause-specific mortality and non-fatal health loss. For each year for which YLDs have been estimated, we computed DALYs by adding YLLs and YLDs for each age-sex-location. Uncertainty in YLLs was assumed to be independent of uncertainty in YLDs. We calculated 1000 draws for DALYs by summing the first draw of the 1000 draws for YLLs and YLDs and then repeating for each subsequent draw. 95% UIs were computed by using the 25th and 975th ordered draw of the DALY uncertainty distribution. We calculated DALYs as the sum of YLLs and YLDs for each cause, location, age group, sex, and year. For more information, please refer to the following figure A.

Figure A. DALY burden estimation for GBD 2019

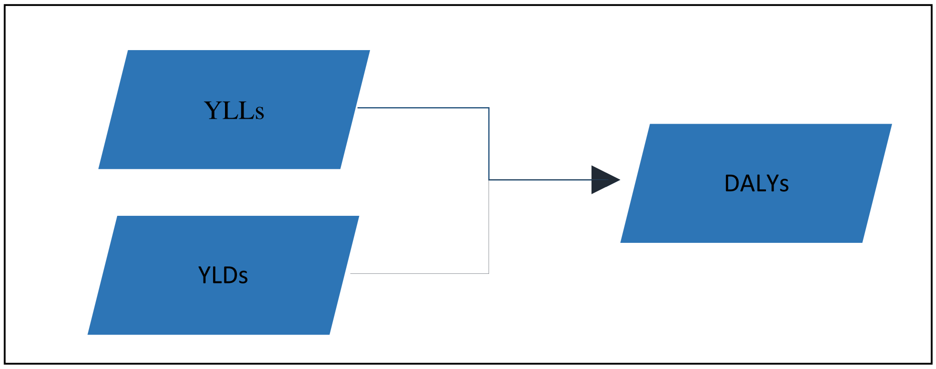

Section 2.7: SDI Analysis[4]

<u>SDI definition</u>

The Socio-demographic Index (SDI) is a composite indicator of background social and economic conditions that influence health outcomes in each location. In short, it is the geometric mean of 0 to 1 indices of total fertility rate (TFR) for those younger than 25 years old (TFU25), mean education for those 15 years old and older (EDU15+), and lag-distributed income (LDI) per capita.

<u>Development of revised SDI indicator</u>

SDI was originally constructed for GBD 2015 by using the Human Development Index (HDI) methodology, wherein a 0 to 1 index value was determined for each of the original three covariate inputs (TFR in ages 15 to 49 years, EDU15+, and LDI per capita) by using the observed minima and maxima over the estimation period to set the scales.[57]

In response to feedback from collaborators and the evolution of the GBD, we have refined the indicator with each GBD cycle. Beginning in GBD 2017, along with our expanded estimation of age-specific fertility, we replaced TFR with TFU25 as one of the three component indices. The TFU25 provides a better measure of women's status in society because it focuses on ages at which childbearing disrupts the pursuit of education and entrance into the workforce. In addition, we observed that in highly developed countries, the TFU25 has tended to decline consistently over time despite rebounds in TFR driven by increasing fertility at older ages. The concordance correlation coefficient between SDI based on the GBD 2016 method and the updated method for GBD 2017 was 0.981.

During GBD 2016, we moved from using relative index scales to using absolute scales to enhance the stability of SDI interpretation over time because we noticed that the measure was highly sensitive to the addition of subnational units that tended to stretch the empirical minima and maxima.[18] We selected the minima and maxima of the scales by examining the relationships each of the inputs had with life expectancy at birth and under-5 mortality and by identifying points of limiting returns at both high and low values if they occurred before theoretical limits (eg, a TFU25 of 0) were reached.

Thus, for each covariate input, an index score of 0 represents the minimum level of each covariate input past which selected health outcomes can get no worse, and an index score of 1 represents the maximum level of each covariate input past which selected health outcomes cease to improve. As a composite, a location with an SDI of 0 would have a theoretical minimum level of development relevant to these health outcomes, and a location with an SDI of 1 would have a theoretical maximum

level of development relevant to these health outcomes. For GBD 2019, final SDI values were multiplied by 100 for reporting, in order to improve understanding of and broader engagement with the values. As such, GBD 2019 SDI is calculated as it was in 2017, but multiplied by 100 at the end (see example calculation below).

We summarise the final scales for GBD 2019 in the table below:

Input	Lower Bound	Upper Bound
TFU25	0	3
LDI per capita	250 USD (5.52 log USD)[b]	60,000 USD (11.00 log USD)
EDU15+	0 years	17 years

[b] The minimum for the LDI scale was originally set at the theoretical limit of 0 USD because we did not observe an asymptotic relationship between log(LDI) and E_0 or 5q0 (probability of death from birth to age 5 years) at lower values of log(LDI). Empirically, however, we also did not observe an LDI less than 350 USD (5.86 log USD) for the estimation period 1970–2016. In log-space, this observation meant that approximately half of our scale was not being utilised, thereby compressing the observed variation in LDI and diminishing its meaningful contribution to SDI. Accordingly, we set the lower limit on LDI to 250 USD (5.52 log USD) to ensure we were fully utilising the range of the scale to capture its variation across space and time, as is the case with the other two inputs.

Using scales described above, we computed the index scores underlying SDI as follows:

$$I_{Cly} = \max\left(\frac{C_{ly} - C_{low}}{C_{high} - C_{low}}, 0.005\right)$$

Where:

I_{cly} is the index for covariate *C*, location *l*, and year *y* and is equal to the difference between the value of that covariate in that location-year and the lower bound of the covariate divided by the difference between the upper and lower bounds for that covariate

If the values of input covariates fell outside the upper or lower bounds (eg, LDI per capita was greater than 60,000 USD), they were mapped to the respective upper or lower bounds. We also note that the index value for TFU25 was computed as $1 - I_{TFU25ly}$ because lower TFU25s correspond to higher levels of development and thus higher index scores. For GBD 2019, we expanded the computation of SDI to 1062 national and subnational locations spanning the time period 1990–2019.

The composite SDI is the geometric mean of these three indices for a given location-year. The cut-off values used to determine quintiles for analysis were then computed by using country-level estimates of SDI for the year 2019, excluding countries with populations less than 1 million.

Example calculation

We present the equation used to calculate SDI for a hypothetical country in the year 2010:

$$TFU25 = 1.09;\ Mean\ educ\ yrs\ pc = 8.23;\ lnLDI = 9.60$$

$$I_{TFU25} = 1 - \frac{1.09 - 0}{3 - 0} = .637$$

$$I_{Educ} = \frac{8.23 - 0}{17 - 0} = .484$$

$$I_{lnLDI} = \frac{9.60 - 5.52}{11.00 - 5.52} = .744$$

$$SDI = \sqrt[3]{I_{TFU25} * I_{Educ} * I_{lnLDI}} = \sqrt[3]{.637 * .484 * .744} = .611$$

$$I_{lnLDI} = \frac{9.58 - 5.52}{11.00 - 5.52} = .741$$

$$SDI = \sqrt[3]{I_{TFR} * I_{Educ} * I_{lnLDI}} = \sqrt{.855 * .543 * .741} = .701$$

$$SDI = 0.701 * 100 = 70.1$$

Section 3: Burden of Disease Estimation for South Africa

The following section provides a more detailed discussion of the differences in burden of disease estimation for South Africa, comparing GBD 2019 methods and the South African Medical Research Council (SAMRC) methods per their *Lancet Global Health* article.[6] We have attempted to provide an overview of key modeling differences as well as highlight improvements to GBD methodology which address prior critiques to GBD.

Segment	GBD	SAMRC[6]
Demography	The GBD team use HIV-free mortality to provide a background mortality rate for the population as one of our model inputs. The description of HIV-free mortality can be found in the link below, as HIV and shocks are both removed from the envelope.<br><br>GBD also provides internally consistent estimates on fertility, mortality and population, all of which have direct impact on the totality of mortality for South Africa. The GBD 2019 Demographics capstone paper[7] has a detailed description on the modeling process. Specific to mortality estimation, we include four major components: U5MR, DDM, 45q15 and model life table. A space-time Gaussian Process Regression is used to generate a time series of U5MR using direct and indirect estimates of U5MR from surveys, censuses and vital registration sources. We also employ a bias adjustment process to account for sampling and non-sampling errors. We synthesize completeness assessment of vital registration systems from traditional Death Distribution Methods and two new variants based on our Bayesian population estimation model. In this process completeness from U5MR is used as a covariate to provide additional information content too. A similar ST-GPR model is applied in estimating adult mortality rate, 45q15, as well where input data are based on DDM adjusted VR data and sibling survival data. The estimated U5MR, 45q15 and crude death rate due to HIV are used as entry parameters to a new model life table system developed for GBD which is flexible in selecting standard age pattern of mortality based on distance in time and location between the location-year we are estimating and the empirical life table database that we have amassed that encompass a much wider variety of age patters of mortality from not only high income countries but also present day or recent experience from developing countries.<br><br>Additional detail on GBD methodology for HIV/AIDS estimation can be found on page 60 for fatal estimation and page 483 for non-fatal estimation.[1]	"We used underlying cause of death data from death notifications for 1997–2012 obtained from Statistics South Africa. These data were adjusted for completeness using indirect demographic techniques for adults and comparison with survey and census estimates for child mortality.<br><br>Completeness of reporting of deaths in children younger than 5 years was estimated by comparing uncorrected rates with rates derived from census and survey data, constraining the trend in completeness of reporting to be monotonically increasing over time. Completeness was estimated for adults with death distribution methods and for adolescents by interpolating between the child and adult estimates. Provincial estimates were rebalanced to ensure that for each sex the sum of the deaths in the provinces, allowing for changes to the provincial boundaries during the study period, was the same as the estimate for the country as a whole. More detail about the estimation of completeness can be found in the technical report on the cleaning and validation of the data.<br><br>Rob E Dorrington led the demographic analysis."

Shocks	For GBD 2019, CODEm models use an HIV/AIDS- and shock-free envelope. To be comparable, cause fractions must also be HIV/AIDS- and shock-free. Cause fractions were uploaded to the CoD database as the number of deaths due to the cause over an adjusted sample in which the number of deaths due to "HIV/AIDS" (and "conflict and terrorism", "police conflict and executions", and "exposure to forces of nature") were removed. To do this, we:<br>1. generate HIV- and shock-free cause fractions<br>2. remove HIV/AIDS deaths from maternal mortality sources<br>3. HIV/AIDS correction of sibling history, census, and survey data<br>4. HIV/AIDS correction of other maternal mortality data<br>Additional detail on each sub-step listed can be found in Vos et al.'s supplementary methods appendix.[1]	Not found in the paper

Section 4: Author Contributions

Managing the estimation or publications process
Alexandra Walker

Writing the first draft of the manuscript
Tom Achoki, Benn Sartorius, David Watkins, Andre Pascal Kengne, Tolu Oni, and Charles Shey Wiysonge

Primary responsibility for applying analytical methods to produce estimates
Mohsen Naghavi

Primary responsibility for seeking, cataloguing, extracting, or cleaning data; designing or coding figures and tables
Scott D Glenn

Providing data or critical feedback on data sources
Benn Sartorius, Andre Pascal Kengne, Charles Shey Wiysonge, Olatunji O Adetokunboh, Obasanjo Afolabi Bolarinwa, Candy T Day, Oluchi Ezekannagha, Themba G Ginindza, Chidozie C D Iwu, Nuworza Kugbey, Chukwudi A Nnaji, JulianDavid Pillay, Karen Sliwa, Liesl J Zuhlke, Christopher J L Murray, and Mohsen Naghavi.

Development of methods or computational machinery
Benn Sartorius, Christopher J L Murray, and Mohsen Naghavi.

Providing critical feedback on methods or results
Tom Achoki, Benn Sartorius, David Watkins, Andre Pascal Kengne, Tolu Oni, Charles Shey Wiysonge, Olatunji O Adetokunboh, Tesleem Kayode Babalola, Obasanjo Afolabi Bolarinwa, Richard G Cowden, Oluchi Ezekannagha, ThembaG Ginindza, Chidozie C D Iwu, Innocent Karangwa, Patrick DMC Katoto, Nuworza Kugbey, Phetole Walter Mahasha, Tivani Phosa Mashamba-Thompson, George A Mensah, Duduzile Edith Ndwandwe, Chukwudi A Nnaji, Thomas Elliot Nyirenda, Julius Nyerere Odhiambo, Kwaku Oppong Asante, Julian David Pillay, Aletta Elisabeth Schutte, Soraya Seedat,Ushotanefe Useh, Heather J Zar, Liesl J Zuhlke, Simon I Hay, Christopher J L Murray, and Mohsen Naghavi.

Drafting the manuscript or revising it critically for important intellectual content
Tom Achoki, Benn Sartorius, David Watkins, Andre Pascal Kengne, Tolu Oni, Charles Shey Wiysonge, Olatunji O Adetokunboh, Mareli M Claassens, Richard G Cowden, Themba G Ginindza, Chidozie C D Iwu, Chinwe Juliana Iwu, Patrick DMC Katoto, Nuworza Kugbey, Desmond Kuupiel, George A Mensah, Mpiko Ntsekhe, Julius Nyerere Odhiambo,Charles D H Parry, Julian David Pillay, Aletta Elisabeth Schutte, Karen Sliwa, Dan J Stein, Frank C Tanser, Heather J Zar,Liesl J Zuhlke, Bongani M Mayosi, Simon I Hay, Christopher J L Murray, and Mohsen Naghavi.

Extracting, cleaning, or cataloging data; designing or coding figures and tables
Scott D Glenn

Management of the overall research enterprise
Benn Sartorius, Alexandra Walker, Simon I Hay, Christopher J L Murray, and Mohsen Naghavi.

Achoki T, *et al. J Epidemiol Community Health* 2022; 76:471–481. doi: 10.1136/jech-2021-217480

Section 5: References

1 Vos T, Lim SS, Abbafati C, *et al.* Global burden of 369 diseases and injuries in 204 countries and territories, 1990–2019: a systematic analysis for the Global Burden of Disease Study 2019. *The Lancet* 2020; **396**: 1204–22.
2 Roth GA, Abate D, Abate KH, *et al.* Global, regional, and national age-sex-specific mortality for 282 causes of death in 195 countries and territories, 1980–2017: a systematic analysis for the Global Burden of Disease Study 2017. *Lancet* 2018; **392**: 1736–88.
3 James SL, Abate D, Abate KH, *et al.* Global, regional, and national incidence, prevalence, and years lived with disability for 354 diseases and injuries for 195 countries and territories, 1990–2017: a systematic analysis for the Global Burden of Disease Study 2017. *The Lancet* 2018; **392**: 1789–858.
4 Kyu HH, Abate D, Abate KH, *et al.* Global, regional, and national disability-adjusted life-years (DALYs) for 359 diseases and injuries and healthy life expectancy (HALE) for 195 countries and territories, 1990–2017: a systematic analysis for the Global Burden of Disease Study 2017. *Lancet* 2018; **392**: 1859–922.
5 Stevens GA, Alkema L, Black RE, *et al.* Guidelines for Accurate and Transparent Health Estimates Reporting: the GATHER statement. *The Lancet* 2016; **388**: e19–23.
6 Pillay-van Wyk V, Msemburi W, Laubscher R, *et al.* Mortality trends and differentials in South Africa from 1997 to 2012: second National Burden of Disease Study. *The Lancet Global Health* 2016; **4**: e642–53.
7 Wang H, Abbas KM, Abbasifard M, *et al.* Global age-sex-specific fertility, mortality, healthy life expectancy (HALE), and population estimates in 204 countries and territories, 1950–2019: a comprehensive demographic analysis for the Global Burden of Disease Study 2019. *The Lancet* 2020; **396**: 1160–203.
8 Todd S, Barr S, Passmore AP. Cause of death in Alzheimer's disease: a cohort study. *QJM* 2013; **106**: 747–53.
9 Brunnström HR, Englund EM. Cause of death in patients with dementia disorders. *Eur J Neurol* 2009; **16**: 488–92.
10 Keene J, Hope T, Fairburn CG, Jacoby R. Death and dementia. *Int J Geriatr Psychiatry* 2001; **16**: 969–74.
11 Thomas BM, Starr JM, Whalley LJ. Death certification in treated cases of presenile Alzheimer's disease and vascular dementia in Scotland. *Age Ageing* 1997; **26**: 401–6.
12 Naghavi M, Makela S, Foreman K, O'Brien J, Pourmalek F, Lozano R. Algorithms for enhancing public health utility of national causes-of-death data. *Population Health Metrics* 2010; **8**: 9.
13 Barker B, Degenhardt L, National Drug and Alcohol Research Centre (Australia). Accidental drug-induced deaths in Australia 1997-2001. Sydney, Australia: National Drug and Alcohol Research Centre, University of New South Wales, 2003. .
14 Roxburgh A, Burns L. Accidental drug-induced deaths due to opioids in Australia, 2011. Sydney, Australia: National Drug and Alcohol Research Centre, University of New South Wales, 2015. .
15 Roxburgh A, Burns L. Cocaine and methamphetamine related drug-induced deaths in Australia, 2011. Sydney, Australia: National Drug and Alcohol Research Centre, University of New South Wales, 2015. .
16 Naghavi M, Wang H, Lozano R, *et al.* Global, regional, and national age–sex specific all-cause and cause-specific mortality for 240 causes of death, 1990–2013: a systematic analysis for the Global Burden of Disease Study 2013. *The Lancet* 2015; **385**: 117–71.
17 Lozano R, Naghavi M, Foreman K, *et al.* Global and regional mortality from 235 causes of death for 20 age groups in 1990 and 2010: a systematic analysis for the Global Burden of Disease Study 2010. *Lancet* 2012; **380**: 2095–128.
18 Wang H, Abajobir AA, Abate KH, *et al.* Global, regional, and national under-5 mortality, adult mortality, age-specific mortality, and life expectancy, 1970–2016: a systematic analysis for the Global Burden of Disease Study 2016. *The Lancet* 2017; **390**: 1084–150.
19 Calvert C, Ronsmans C. The contribution of HIV to pregnancy-related mortality: a systematic review and meta-analysis. *Aids* 2013; **27**: 1631–9.
20 Figueroa-Damián R. Pregnancy outcome in women infected with the human immunodeficiency virus. *Salud Publica Mex* 1999; **41**: 362–7.
21 Ryder RW, Nsuami M, Nsa W, *et al.* Mortality in HIV-1-seropositive women, their spouses and their newly born children during 36 months of follow-up in Kinshasa, Zaïre. *AIDS* 1994; **8**: 667–72.

22 Zvandasara P, Saungweme G, Mlambo JT, Moyo J. Post Caesarean section infective morbidity in HIV-positive women at a tertiary training hospital in Zimbabwe. *Cent Afr J Med* 2007; **53**: 43–7.
23 Chilongozi D, Wang L, Brown L, *et al.* Morbidity and mortality among a cohort of human immunodeficiency virus type 1-infected and uninfected pregnant women and their infants from Malawi, Zambia, and Tanzania. *Pediatr Infect Dis J* 2008; **27**: 808–14.
24 Leroy V, Ladner J, Nyiraziraje M, *et al.* Effect of HIV-1 infection on pregnancy outcome in women in Kigali, Rwanda, 1992-1994. Pregnancy and HIV Study Group. *AIDS* 1998; **12**: 643–50.
25 Kourtis A, Bansil P, McPheeters M, Meikle S, Posner S, Jamieson D. Hospitalizations of pregnant HIV-infected women in the USA prior to and during the era of HAART, 1994–2003. *AIDS* 2006; **20**: 1823–31.
26 Ticconi C, Mapfumo M, Dorrucci M, *et al.* Effect of Maternal HIV and Malaria Infection on Pregnancy and Perinatal Outcome in Zimbabwe. *Jaids Journal of Acquired Immune Deficiency Syndromes* 2003; **34**: 289–94.
27 Brown T, Peerapatanapokin W. The Asian Epidemic Model: a process model for exploring HIV policy and programme alternatives in Asia. *Sexually Transmitted Infections* 2004; **80**: i19–24.
28 DerSimonian R, Laird N. Meta-analysis in clinical trials. *Controlled Clinical Trials* 1986; **7**: 177–88.
29 Lozano R, Lopez AD, Atkinson C, Naghavi M, Flaxman AD, Murray CJ. Performance of physician-certified verbal autopsies: multisite validation study using clinical diagnostic gold standards. *Population Health Metrics* 2011; **9**: 32.
30 Foreman KJ, Lozano R, Lopez AD, Murray CJ. Modeling causes of death: an integrated approach using CODEm. *Population Health Metrics* 2012; **10**: 1.
31 Bell RM, Koren Y. Lessons from the Netflix Prize Challenge. *SIGKDD Explor Newsl* 2007; **9**: 75–9.
32 Bell RM, Koren Y, Volinsky C. All Together Now: A Perspective on the Netflix Prize. *CHANCE* 2010; **23**: 24–9.
33 Flaxman AD, Vos T, Murray CJL, Kiyono P, editors. An integrative metaregression framework for descriptive epidemiology, 1 edition. Seattle: University of Washington Press, 2015.
34 Murray CJL, Callender CSKH, Kulikoff XR, *et al.* Population and fertility by age and sex for 195 countries and territories, 1950–2017: a systematic analysis for the Global Burden of Disease Study 2017. *Lancet* 2018; **392**: 1995–2051.
35 GBD 2019 Demographics Collaborators. Global, regional, and national age-sex-specific fertility, mortality, and population estimates, 1950–2019: a comprehensive demographic analysis for the Global Burden of Disease Study 2019. *The Lancet* In Press.
36 Zheng P, Aravkin AY, Barber R, Sorensen RJD, Murray CJL. Trimmed Constrained Mixed Effects Models: Formulations and Algorithms. *arXiv:190910700 [math, stat]* 2019; published online Sept 23. http://arxiv.org/abs/1909.10700 (accessed Nov 15, 2019).
37 Aravkin A, Davis D. Trimmed Statistical Estimation via Variance Reduction. *Mathematics of OR* 2019; published online July 5. https://pubsonline.informs.org/doi/10.1287/moor.2019.0992 (accessed Nov 15, 2019).
38 Rousseeuw P. Multivariate estimation with high breakdown point. 1985. DOI:10.1007/978-94-009-5438-0_20.
39 Wächter A, Biegler LT. On the implementation of an interior-point filter line-search algorithm for large-scale nonlinear programming. *Math Program* 2006; **106**: 25–57.
40 Boor C de. A Practical Guide to Splines. New York: Springer-Verlag, 1978 https://www.springer.com/gp/book/9780387953663 (accessed Nov 15, 2019).
41 Friedman JH. Multivariate Adaptive Regression Splines. *Ann Statist* 1991; **19**: 1–67.
42 Pya N, Wood SN. Shape constrained additive models. *Stat Comput* 2015; **25**: 543–59.
43 Efron B, Tibshirani RJ. An Introduction to the Bootstrap, 1 edition. New York: Chapman and Hall/CRC, 1993.
44 Burstein R, Fleming T, Haagsma J, Salomon JA, Vos T, Murray CJL. Estimating distributions of health state severity for the global burden of disease study. *Population Health Metrics* 2015; **13**: 31.
45 Medical Expenditure Panel Survey Home. https://meps.ahrq.gov/mepsweb/ (accessed Nov 15, 2019).
46 NIAAA Publications. http://pubs.niaaa.nih.gov/publications/arh29-2/74-78.htm (accessed Nov 15, 2019).
47 4326.0 - Mental Health and Wellbeing: Profile of Adults, Australia, 1997. 1998; published online March 12. http://www.abs.gov.au/ausstats/abs@.nsf/ProductsbyReleaseDate/D5A0AC778746378FCA2574EA00122887?OpenDocument (accessed Nov 15, 2019).
48 Salomon JA, Haagsma JA, Davis A, *et al.* Disability weights for the Global Burden of Disease 2013 study. *The Lancet Global Health* 2015; **3**: e712–23.
49 Stouthard MEA, Essink-Bot ML, Bonsel GJ, *et al.* Disability weights for diseases in the Netherlands. AmsterdamInst. Sociale Geneeskunde, 1997 https://dare.uva.nl/search?identifier=e7cbed27-7fab-4104-9b44-1657515747c2 (accessed Nov 15, 2019).
50 Campeau L. The Canadian Cardiovascular Society grading of angina pectoris revisited 30 years later. *Can J Cardiol* 2002; **18**: 371–9.
51 Dolgin M, Committee NYHAC. Nomenclature and criteria for diagnosis of diseases of the heart and great vessels, 9th ed. Boston, MA: Little Brown & Co, 1994 https://trove.nla.gov.au/version/13288061 (accessed Nov 15, 2019).
52 Nord E. Disability weights in the Global Burden of Disease 2010: Unclear meaning and overstatement of

international agreement. *Health Policy* 2013; **111**: 99–104.
53 Taylor HR, Jonas JB, Keeffe J, *et al.* Disability weights for vision disorders in Global Burden of Disease study. *The Lancet* 2013; **381**: 23.
54 Voigt K, King NB. Disability weights in the global burden of disease 2010 study: two steps forward, one step back? *Bull World Health Organ* 2014; **92**: 226–8.
55 Kretzschmar M, Mangen M-JJ, Pinheiro P, *et al.* New Methodology for Estimating the Burden of Infectious Diseases in Europe. *PLOS Medicine* 2012; **9**: e1001205.
56 Vos T, Flaxman AD, Naghavi M, *et al.* Years lived with disability (YLDs) for 1160 sequelae of 289 diseases and injuries 1990–2010: a systematic analysis for the Global Burden of Disease Study 2010. *The Lancet* 2012; **380**: 2163–96.
57 Wang H, Naghavi M, Allen C, *et al.* Global, regional, and national life expectancy, all-cause mortality, and cause-specific mortality for 249 causes of death, 1980–2015: a systematic analysis for the Global Burden of Disease Study 2015. *The Lancet* 2016; **388**: 1459–544.
58 Dorrington R, Bradshaw D, Laubscher R, Nannan N. Rapid Mortality Surveillance Report 2017. Cape Town: South African Medical Research Council https://www.samrc.ac.za/sites/default/files/files/2019-02-06/RapidMortalitySurveillanceReport2017.pdf.

Achoki T, *et al. J Epidemiol Community Health* 2022; 76:471–481. doi: 10.1136/jech-2021-217480

Section 6: Figures and Tables

Figure S1: Absolute change in mortality by province, 1990-2007 versus 2007-2019. Panel A: all-cause mortality. Panel B: HIV/AIDS

A: All-cause mortality

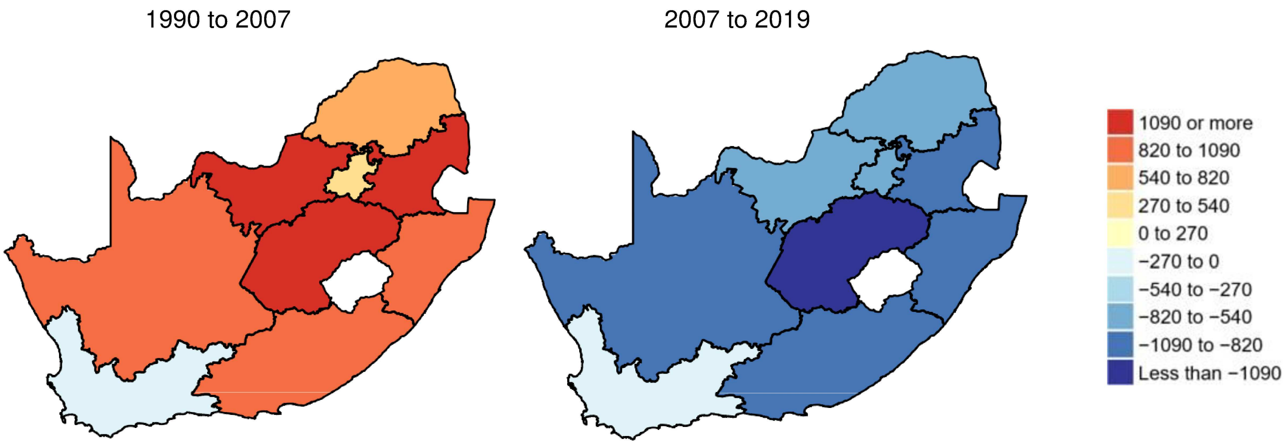

B: HIV/AIDS mortality

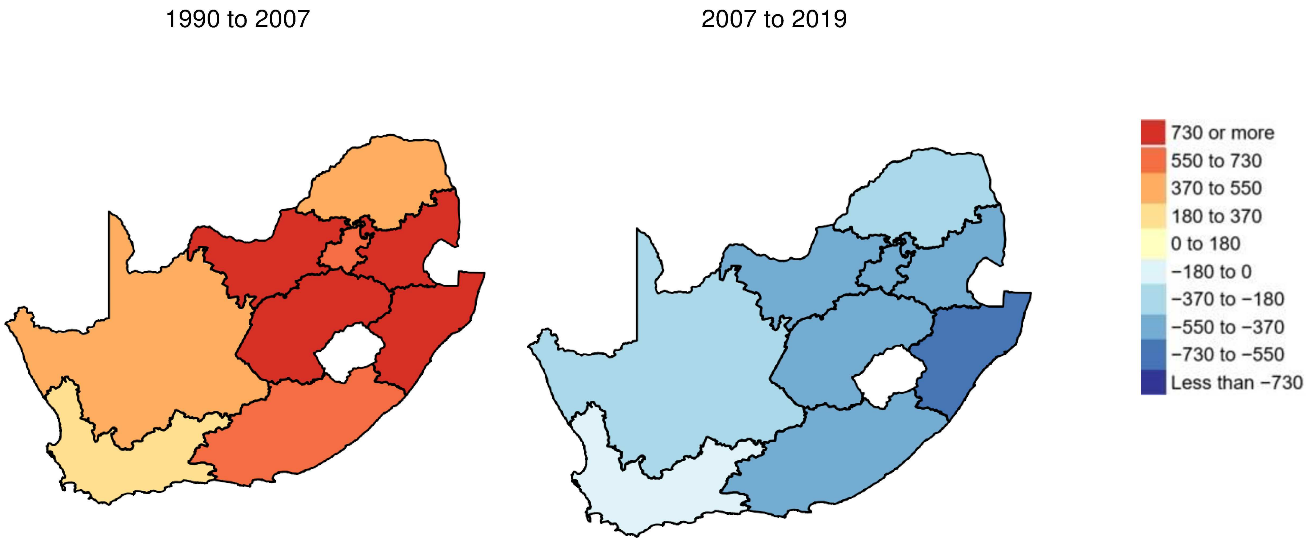

Achoki T, *et al. J Epidemiol Community Health* 2022; 76:471–481. doi: 10.1136/jech-2021-217480

Figure S2: Under-5 mortality rates per 1000 live births (5q0), both sexes

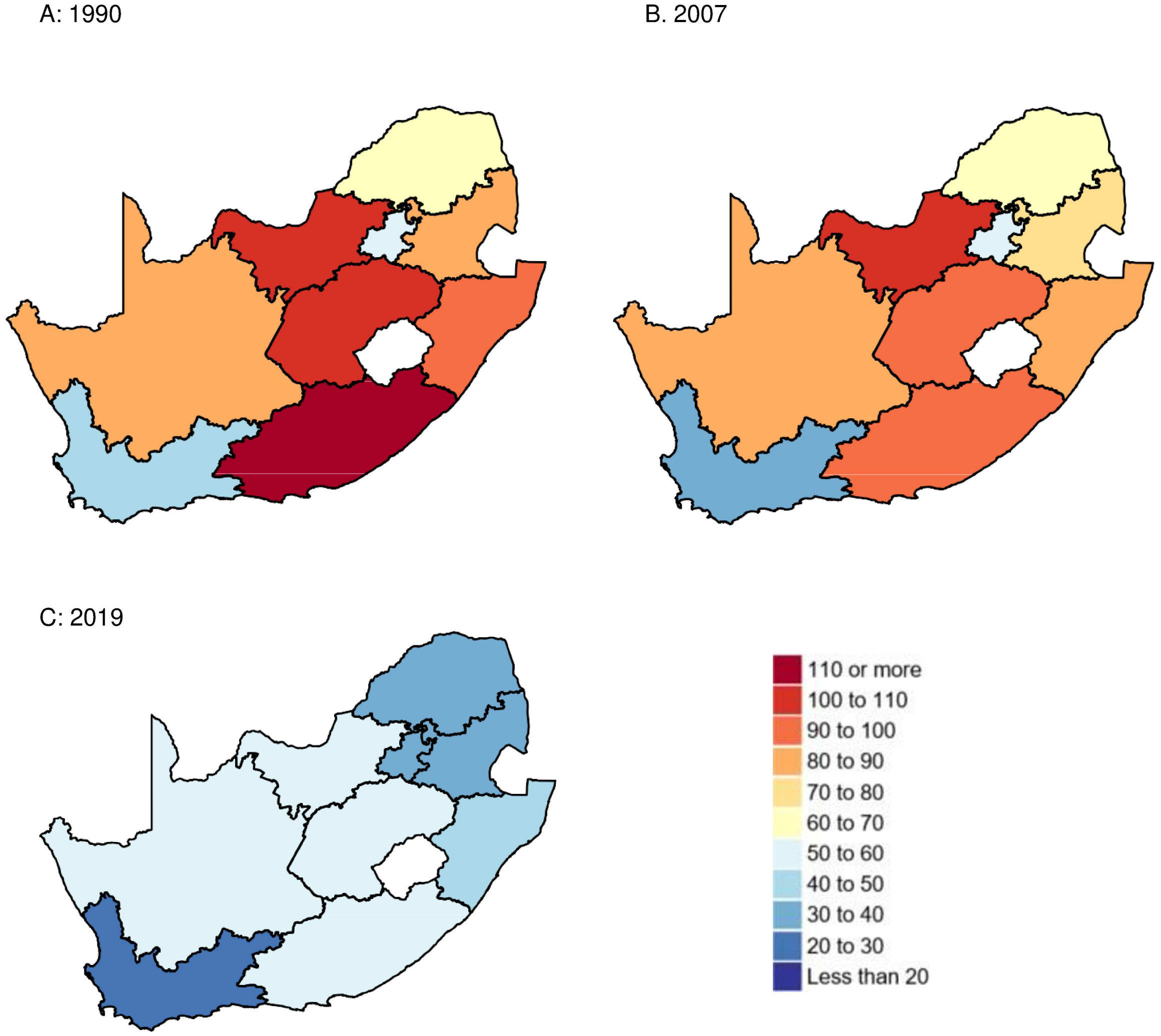

Achoki T, *et al. J Epidemiol Community Health* 2022; 76:471–481. doi: 10.1136/jech-2021-217480

Figure S3: Maternal mortality rates per 100 000 live births

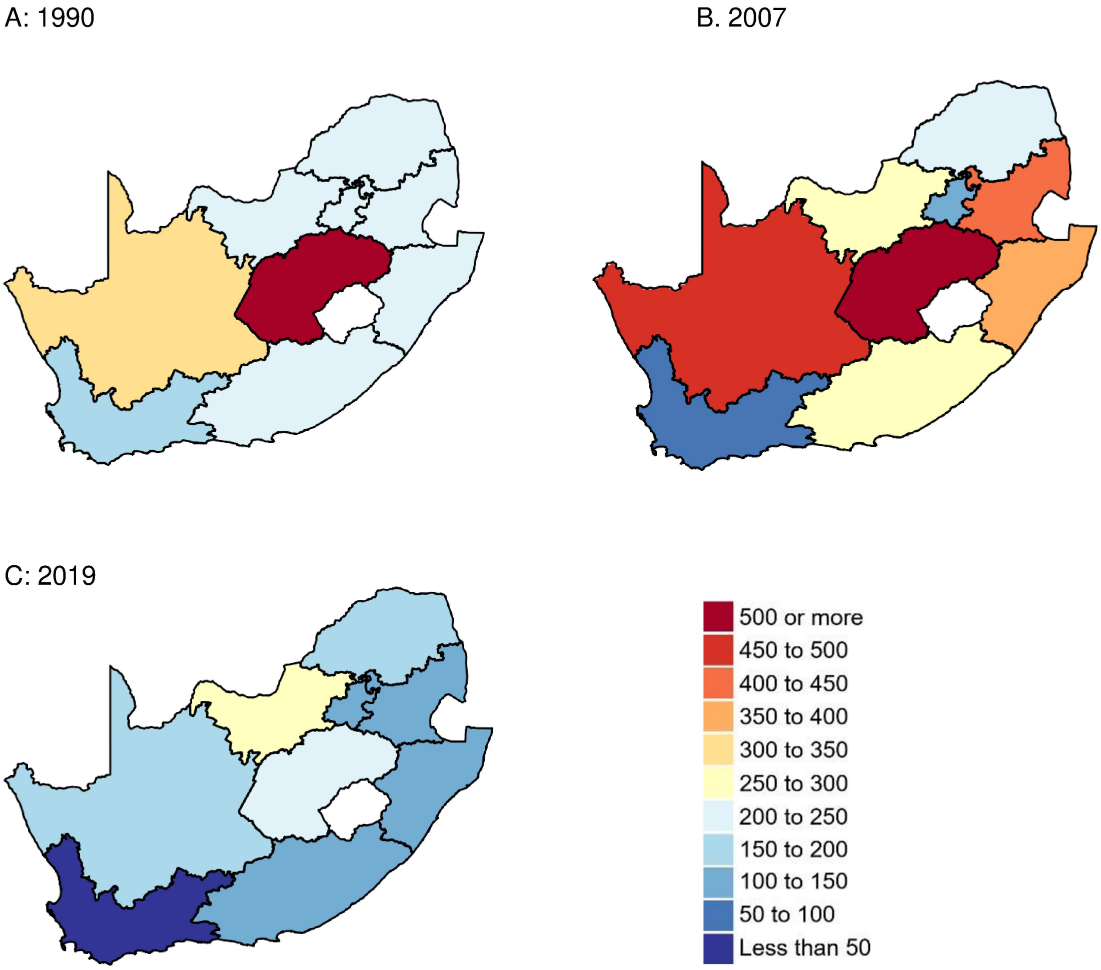

Figure S4: Decomposition of DALYs into causes of YLLs and YLDs by province. Panel A: 1990. Panel B: 2007. Panel C. 2019.

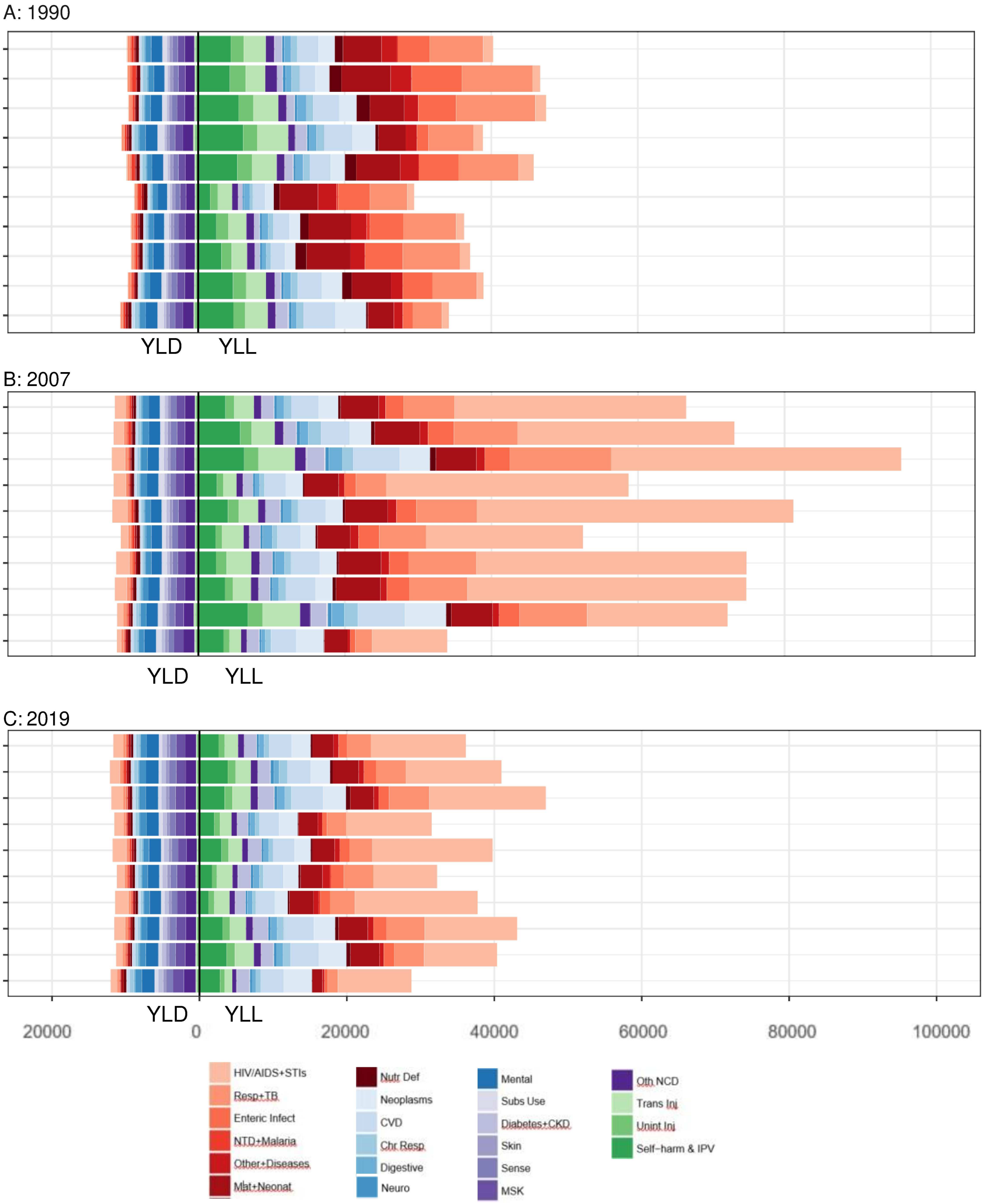

Achoki T, *et al. J Epidemiol Community Health* 2022; 76:471–481. doi: 10.1136/jech-2021-217480

Figure S5: Health access and quality (HAQ) and life expectancy at birth, 1990-2019, South Africa, provinces, andSADC member states

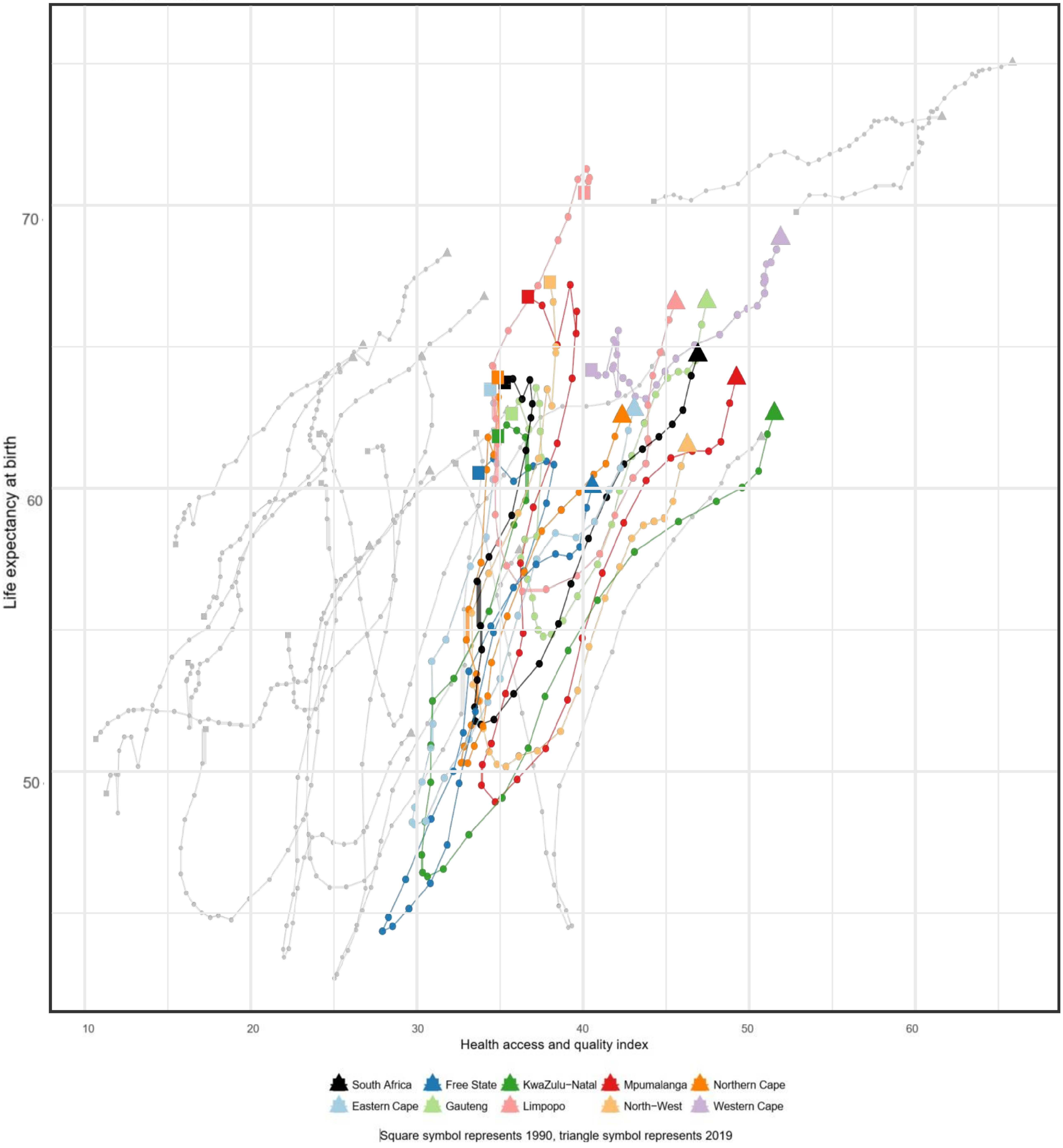

Achoki T, *et al. J Epidemiol Community Health* 2022; 76:471–481. doi: 10.1136/jech-2021-217480

Figure S6: Contribution of various causes of death to changes in life expectancy for males and females by province. Panel A: 1990-2007. Panel B: 1990-2019. Panel C: 2007-2019.

Panel A:

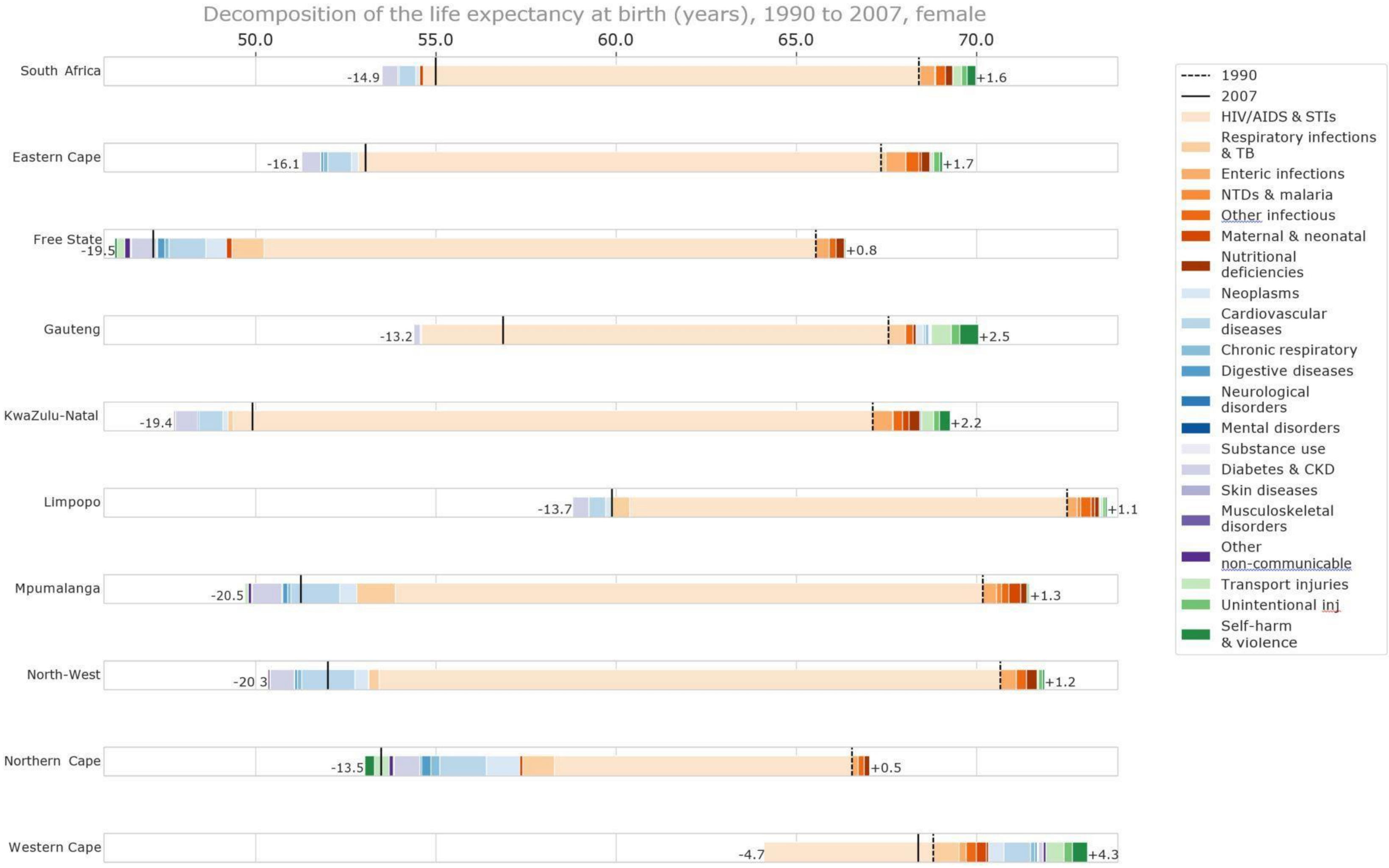

Panel B:

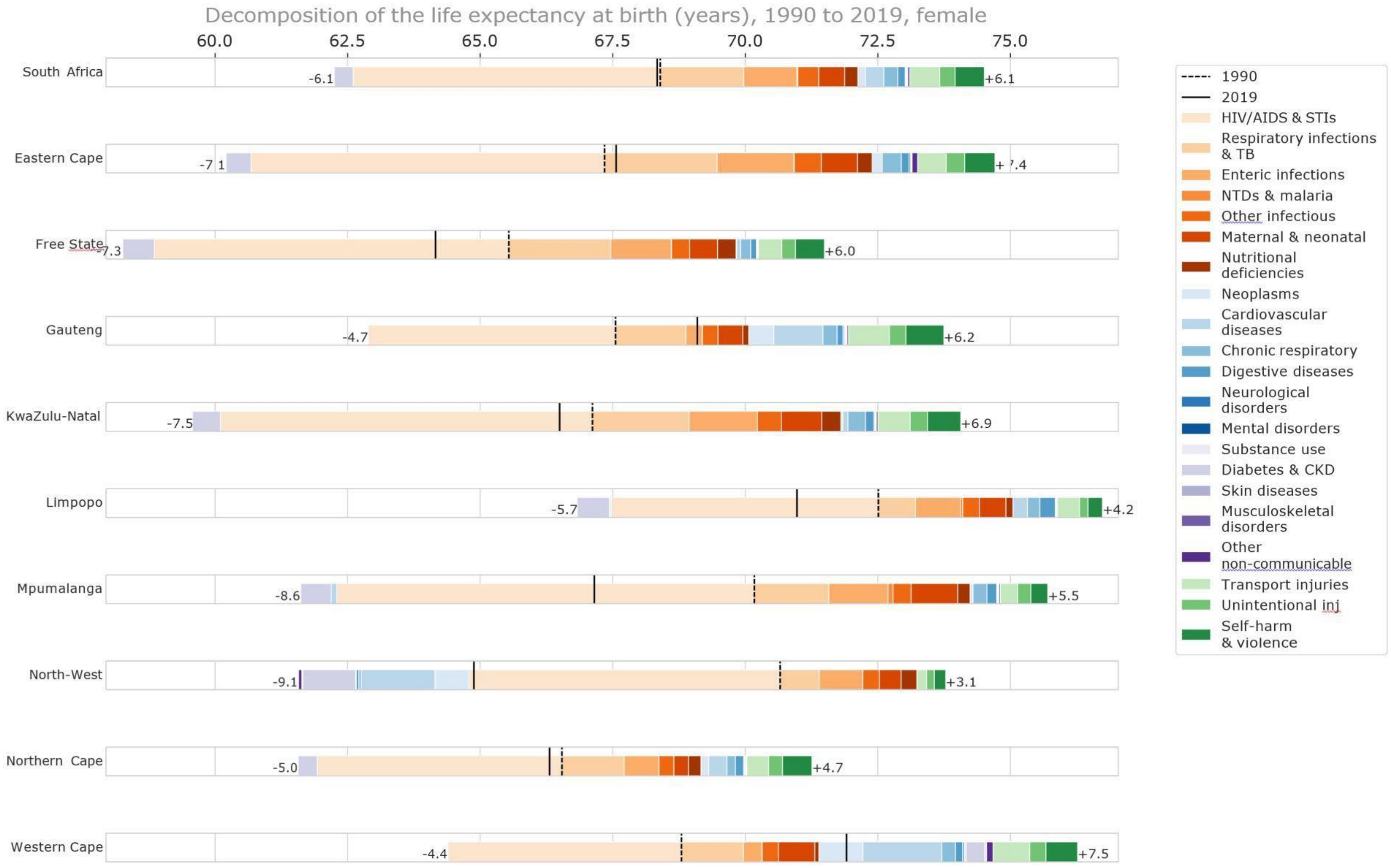
Decomposition of the life expectancy at birth (years), 1990 to 2019, female
60.0
62.5
65.0
67.5
70.0
72.5
75.0
South Africa
-6.1
+6.1
Eastern Cape
Free State
+6.0
Gauteng
-4.7
+6.2
KwaZulu-Natal
-7.5
+6.9
Limpopo
-5.7
+4.2
Mpumalanga
-8.6
+5.5
North-West
-9.1
+3.1
Northern Cape
-5.0
+4.7
Western Cape
-4.4
+7.5
1990
2019
HIV/AIDS & STIs
Respiratory infections & TB
Enteric infections
NTDs & malaria
Other infectious
Maternal & neonatal
Nutritional deficiencies
Neoplasms
Cardiovascular diseases
Chronic respiratory
Digestive diseases
Neurological disorders
Mental disorders
Substance use
Diabetes & CKD
Skin diseases
Musculoskeletal disorders
Other non-communicable
Transport injuries
Unintentional inj
Self-harm & violence

Panel C:

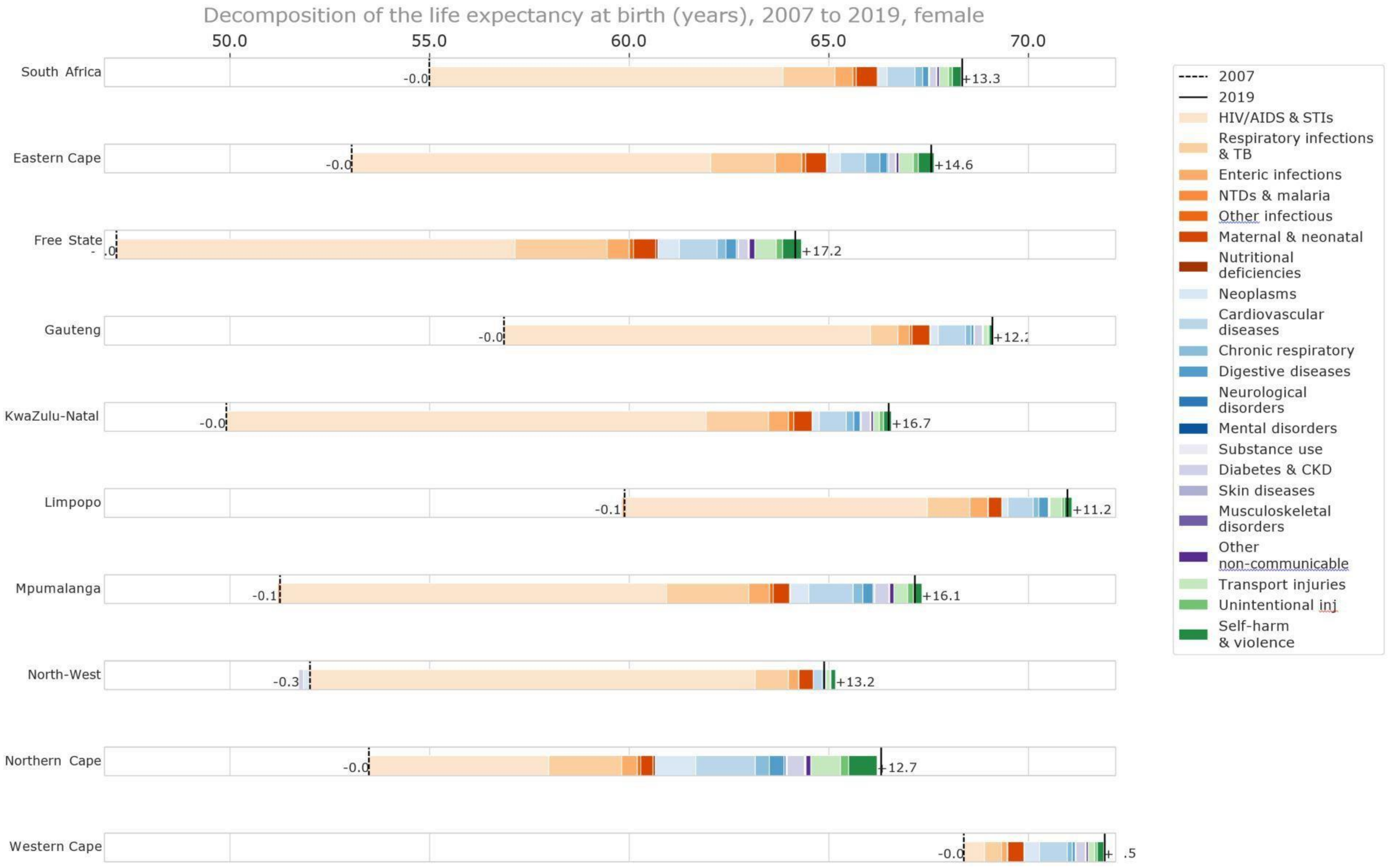
Decomposition of the life expectancy at birth (years), 2007 to 2019, female
50.0
55.0
60.0
65.0
70.0
South Africa
-0.0
+13.3
Eastern Cape
-0.0
+14.6
Free State
+17.2
Gauteng
-0.0
KwaZulu-Natal
-0.0
+16.7
Limpopo
-0.1
+11.2
Mpumalanga
-0.1
+16.1
North-West
-0.3
+13.2
Northern Cape
-0.0
+12.7
Western Cape
-0.0
2007
2019
HIV/AIDS & STIs
Respiratory infections & TB
Enteric infections
NTDs & malaria
Other infectious
Maternal & neonatal
Nutritional deficiencies
Neoplasms
Cardiovascular diseases
Chronic respiratory
Digestive diseases
Neurological disorders
Mental disorders
Substance use
Diabetes & CKD
Skin diseases
Musculoskeletal disorders
Other non-communicable
Transport injuries
Unintentional inj
Self-harm & violence

Panel A:

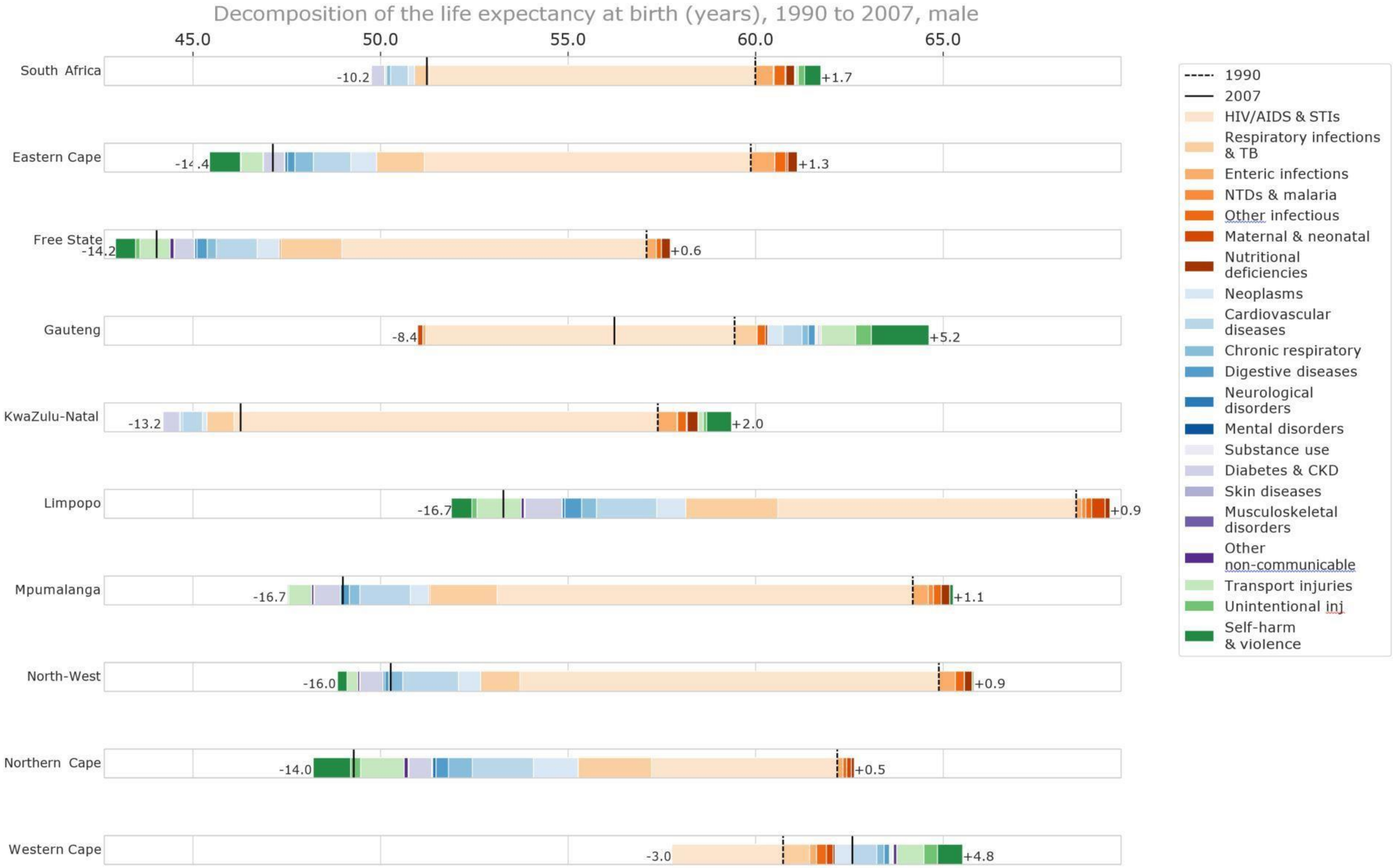
Decomposition of the life expectancy at birth (years), 1990 to 2007, male
45.0
50.0
55.0
60.0
65.0
South Africa
-10.2
+1.7
Eastern Cape
+1.3
Free State
-14.2
+0.6
Gauteng
-8.4
+5.2
KwaZulu-Natal
-13.2
+2.0
Limpopo
-16.7
+0.9
Mpumalanga
-16.7
+1.1
North-West
-16.0
+0.9
Northern Cape
-14.0
+0.5
Western Cape
-3.0
+4.8
1990
2007
HIV/AIDS & STIs
Respiratory infections & TB
Enteric infections
NTDs & malaria
Other infectious
Maternal & neonatal
Nutritional deficiencies
Neoplasms
Cardiovascular diseases
Chronic respiratory
Digestive diseases
Neurological disorders
Mental disorders
Substance use
Diabetes & CKD
Skin diseases
Musculoskeletal disorders
Other non-communicable
Transport injuries
Unintentional inj
Self-harm & violence

Panel B:

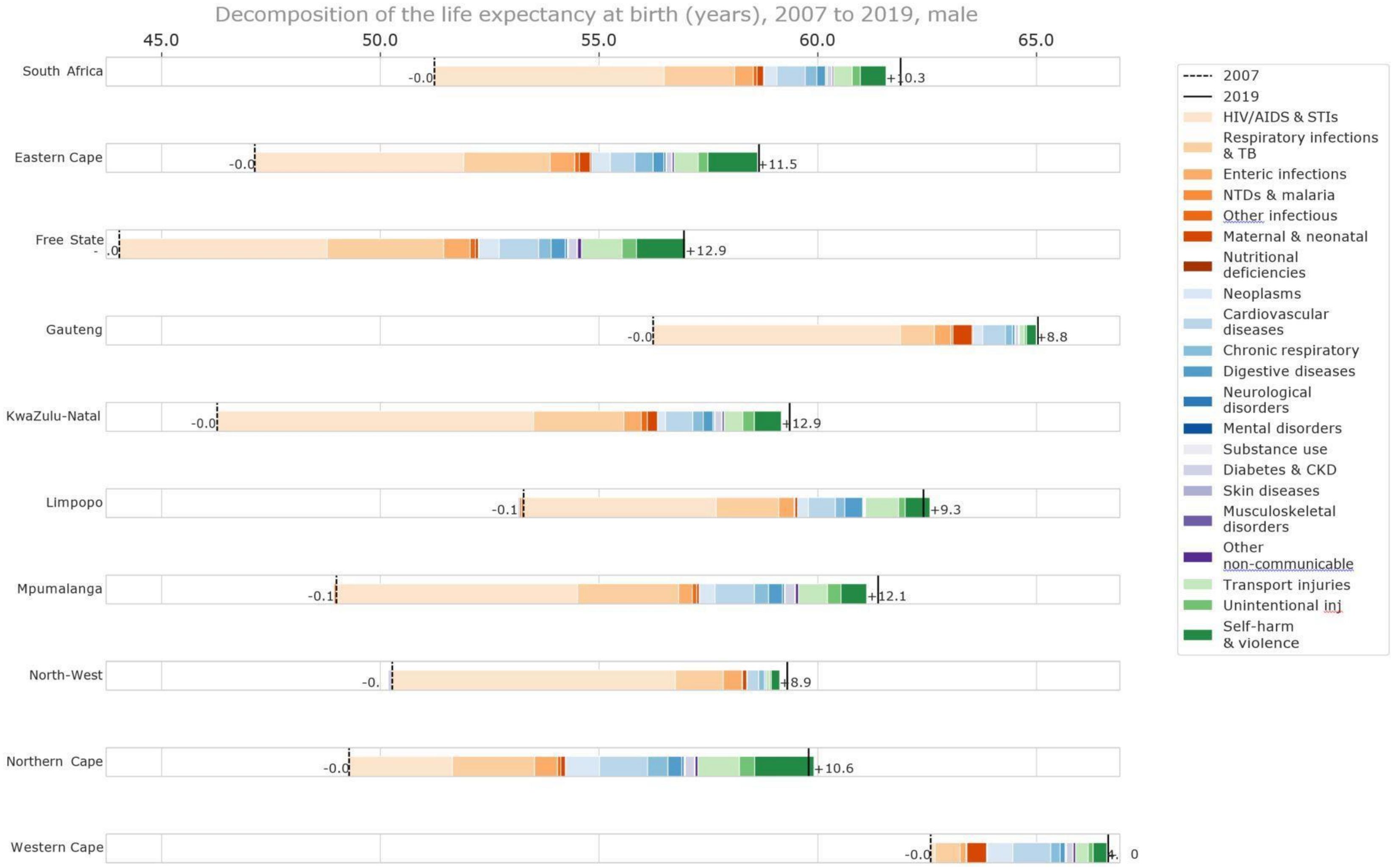
Decomposition of the life expectancy at birth (years), 2007 to 2019, male
45.0
50.0
55.0
60.0
65.0
South Africa
-0.0
+10.3
Eastern Cape
-0.0
+11.5
Free State
+12.9
Gauteng
-0.0
+8.8
KwaZulu-Natal
-0.0
+12.9
Limpopo
-0.1
+9.3
Mpumalanga
-0.1
+12.1
North-West
+8.9
Northern Cape
-0.0
+10.6
Western Cape
-0.0
2007
2019
HIV/AIDS & STIs
Respiratory infections & TB
Enteric infections
NTDs & malaria
Other infectious
Maternal & neonatal
Nutritional deficiencies
Neoplasms
Cardiovascular diseases
Chronic respiratory
Digestive diseases
Neurological disorders
Mental disorders
Substance use
Diabetes & CKD
Skin diseases
Musculoskeletal disorders
Other non-communicable
Transport injuries
Unintentional inj
Self-harm & violence

Panel C:

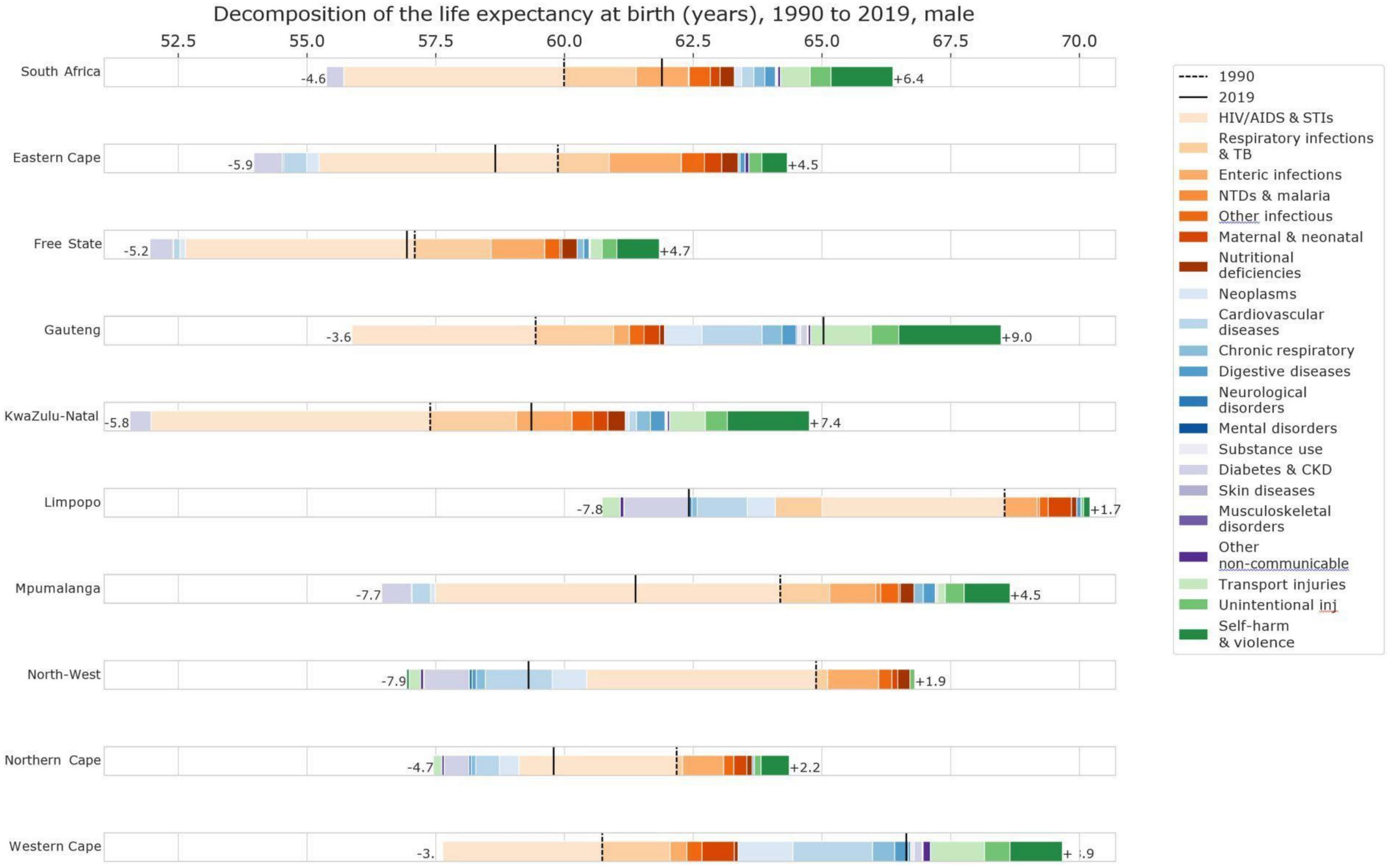
Decomposition of the life expectancy at birth (years), 1990 to 2019, male
52.5
55.0
57.5
60.0
62.5
65.0
67.5
70.0
South Africa
-4.6
+6.4
Eastern Cape
-5.9
+4.5
Free State
-5.2
+4.7
Gauteng
-3.6
+9.0
KwaZulu-Natal
-5.8
+7.4
Limpopo
-7.8
+1.7
Mpumalanga
-7.7
+4.5
North-West
-7.9
+1.9
Northern Cape
-4.7
+2.2
Western Cape
1990
2019
HIV/AIDS & STIs
Respiratory infections & TB
Enteric infections
NTDs & malaria
Other infectious
Maternal & neonatal
Nutritional deficiencies
Neoplasms
Cardiovascular diseases
Chronic respiratory
Digestive diseases
Neurological disorders
Mental disorders
Substance use
Diabetes & CKD
Skin diseases
Musculoskeletal disorders
Other non-communicable
Transport injuries
Unintentional inj
Self-harm & violence

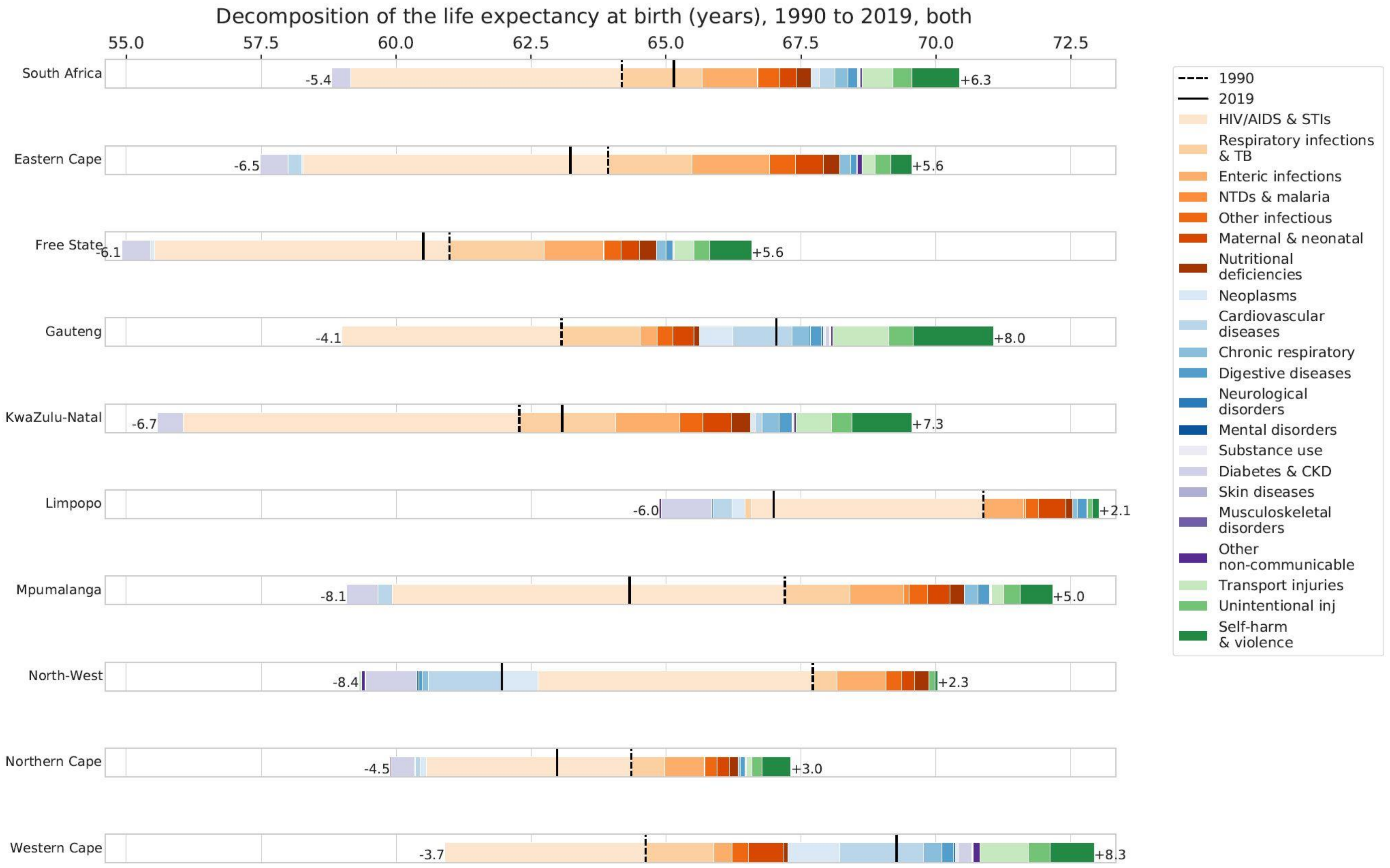
Decomposition of the life expectancy at birth (years), 1990 to 2019, both
55.0
57.5
60.0
62.5
65.0
67.5
70.0
72.5
South Africa
-5.4
+6.3
Eastern Cape
-6.5
+5.6
Free State
-6.1
+5.6
Gauteng
-4.1
+8.0
KwaZulu-Natal
-6.7
+7.3
Limpopo
-6.0
+2.1
Mpumalanga
-8.1
+5.0
North-West
-8.4
+2.3
Northern Cape
-4.5
+3.0
Western Cape
-3.7
+8.3
1990
2019
HIV/AIDS & STIs
Respiratory infections & TB
Enteric infections
NTDs & malaria
Other infectious
Maternal & neonatal
Nutritional deficiencies
Neoplasms
Cardiovascular diseases
Chronic respiratory
Digestive diseases
Neurological disorders
Mental disorders
Substance use
Diabetes & CKD
Skin diseases
Musculoskeletal disorders
Other non-communicable
Transport injuries
Unintentional inj
Self-harm & violence

Table S1: GATHER checklist of information that should be included in reports of global health estimates, with description of compliance and location information for "Health trends, inequalities, and opportunities in South Africa's provinces, 1990—2019: Findings from the Global Burden of Disease 2019 Study."

#	Checklist item	Description of compliance	Reference
	Objectives and funding		
1	Define the indicator(s), populations (including age, sex, and geographic entities), and time period(s) for which estimateswere made.	Narrative provided in paper providing description of indicators, population, definitions, and time period	Main text
2	List the funding sources for the work.	Funding source listed in paper	Role of Funder and Author Contributions section
	Data Inputs		
	For all data inputs from multiple sources that are synthesized as part of the study:		
3	Describe how the data were identified and how the datawere accessed.	Narrative of data seeking methods provided	Main text (Methods)
4	Specify the inclusion and exclusion criteria. Identify all ad-hoc exclusions.	Narrative about inclusion/exclusion provided	Main text (Methods)
5	Provide information on all included data sources and their main characteristics. For each data source used, report reference information or contact name/institution, population represented, data collection method, year(s) of data collection, sex and age range, diagnostic criteria ormeasurement method, and sample size, as relevant.	Complete list of data sources included alongside interactive, online tool providing metadata for data sources	Appendix and online at https://ghdx.healthdata.org/gbd-2019
6	Identify and describe any categories of input data that have potentially important biases (e.g., based on characteristicslisted in item 5).	Summary of limitations provided in narrative	Main text (Methods and Discussion)
	For data inputs that contribute to the analysis but were not synthesized as part of the study:		
7	Describe and give sources for any other data inputs.	Included in online data source tool, available at publication https://ghdx.healthdata.org/gbd-2019	Online data sources tool; references
	For all data inputs:		
8	Provide all data inputs in a file format from which data canbe efficiently extracted (e.g., a spreadsheet rather than a PDF), including all relevant meta-data listed in item 5. For any data inputs that cannot be shared because of ethical or legal reasons, such as third-party ownership, provide a contact name or the name of the institution that retains the right to the data.	Downloads of input data available through online tools, including data visualization tools and results query tools available upon publication at https://ghdx.healthdata.org/gbd-2019; results not available in tools are available upon request	Online data visualization tools, results query tools, and the Global Health Data Exchange, https://ghdx.healthdata.org
	Data analysis		
9	Provide a conceptual overview of the data analysis method. A diagram may be helpful.	Narrative overview provided, diagrams provided in GBD 2019 methodological write-ups and Supplementary Appendix	Main text (Methods), Supplementary Appendix, GBD 2019 Methods appendices, https://ghdx.healthdata.org/gbd-2019
10	Provide a detailed description of all steps of the analysis, including mathematical formulae. This description shouldcover, as relevant, data cleaning, data pre-processing, data adjustments and weighting of data sources, and mathematical or statistical model(s).	Narrative overview provided, detailed description in GBD 2019 methodological write-ups	Main text (Methods), Supplementary Appendix, GBD 2019 Methods appendices, https://ghdx.healthdata.org/gbd-2019
11	Describe how candidate models were evaluated and howthe final model(s) were selected.	Narrative overview provided, detailed description in GBD 2019 methodological write-ups	Main text (Methods), Supplementary Appendix, GBD 2019 Methods appendices, https://ghdx.healthdata.org/gbd-2019
12	Provide the results of an evaluation of model performance,if done, as well as the results of any relevant sensitivity analysis.	Narrative overview provided, detailed description in GBD 2019 methodological write-ups	Main text (Methods), GBD 2019 Methods appendices, https://ghdx.healthdata.org/gbd-2019

13	Describe methods for calculating uncertainty of the estimates. State which sources of uncertainty were, andwere not, accounted for in the uncertainty analysis.	Narrative overview provided, detailed description in GBD 2019 methodological write-ups	Main text (Methods), GBD 2019 Methods appendices, https://ghdx.healthdata.org/gbd-2019
14	State how analytic or statistical source code used togenerate estimates can be accessed.	Access statement provided with GBD2019	Code is provided in an online repository, https://ghdx.healthdata.org/gbd-2019/code
15	Provide published estimates in a file format from which data can be efficiently extracted.	Results are available upon publication online through data visualization tools,the Global Health Data Exchange, andonline results query tool	Main text (Results), Appendix, and online data tools, https://ghdx.healthdata.org/gbd-2019
16	Report a quantitative measure of the uncertainty of the estimates (e.g. uncertainty intervals).	Uncertainty intervals are provided withall results	Main text (Results), Appendix, and online data tools, https://ghdx.healthdata.org/gbd-2019
17	Interpret results in light of existing evidence. If updating aprevious set of estimates, describe the reasons for changes in estimates.	Discussion of methodological changes between GBD cycles provided in the narrative; discussion of existing evidence in summary boxes, main text	Main text (Methods,Discussion) and Summary boxes
18	Discuss limitations of the estimates. Include a discussion of any modelling assumptions or data limitations that affectinterpretation of the estimates.	Discussion of limitations provided in the narrative	Main text (Discussion)

Achoki T, *et al. J Epidemiol Community Health* 2022; 76:471–481. doi: 10.1136/jech-2021-217480

Table S2: GBD 2019 South Africa subnational data sources

Data sources used in GBD 2019 South Africa national and subnational estimation. All information presented in this table can be accessed on the Global Health Data Exchange (GHDx): http://ghdx.healthdata.org/geography/south-africa

NID	Data Type	Title	Citation	GBD Component
422985	Administrative data	Euromonitor Passport - Alcoholic Drinks Statistics	Euromonitor International. Euromonitor Passport - Alcoholic Drinks Statistics . London, United Kingdom: Euromonitor International.	1 - Covariates
282698	Administrative data	Euromonitor Passport - Fresh Foods Market Statistics	Euromonitor International. Euromonitor Passport - Fresh Foods Market Statistics. London, United Kingdom: Euromonitor International.	1 - Covariates
422982	Administrative data	Euromonitor Passport - Smoking Tobacco Statistics	Euromonitor International. Euromonitor Passport - Smoking Tobacco Statistics . London, United Kingdom: Euromonitor International.	1 - Covariates
238501	Administrative data	FAO Supply Utilization Accounts 1961-2013	FAO Supply Utilization Accounts 1961-2013. Personal Correspondence with Dr. Josef Schmidhuber, 2016. [Unpublished].	1 - Covariates
244478	Administrative data	FAOSTAT Commodity Balances - Crops Primary Equivalent	Food and Agriculture Organization of the United Nations (FAO). FAOSTAT Commodity Balances - Crops Primary Equivalent. Rome, Italy: Food and Agriculture Organization of the United Nations (FAO).	1 - Covariates
318161	Administrative data	Global Fortification Data Exchange - Map: Count of Nutrients In Fortification Standards	Food Fortification Initiative (United States), Global Alliance for Improved Nutrition (GAIN), Iodine Global Network (Canada), Micronutrient Forum (Canada). Global Fortification Data Exchange - Map: Count of Nutrients In Fortification Standards.	1 - Covariates
312214	Administrative data	Insecticide Treated Net Procurement Data, Personal Communication with the World Health Organization 2016	World Health Organization (WHO). Insecticide Treated Net Procurement Data, Personal Communication with the World Health Organization 2016.	1 - Covariates
354827	Administrative data	Insecticide Treated Net Procurement Data, Personal Communication with the World Health Organization 2017	World Health Organization (WHO). Insecticide Treated Net Procurement Data, Personal Communication with the World Health Organization 2017.	1 - Covariates
425630	Administrative data	Insecticide Treated Nets Manufactured by Country, Personal Communication with the World Health Organization 2019	World Health Organization (WHO). Insecticide Treated Nets Manufactured by Country, Personal Communication with the World Health Organization 2019.	1 - Covariates
425631	Administrative data	National Malaria Control Program Insecticide Treated Nets Data, Personal Communication with the World Health Organization 2019	World Health Organization (WHO). National Malaria Control Program Insecticide Treated Nets Data, Personal Communication with the World Health Organization 2019.	1 - Covariates
376303	Administrative data	Organization for Economic Co-operation and Development Data - Hospital Beds	Organization for Economic Co-operation and Development (OECD). Organization for Economic Co-operation and Development Data - Hospital Beds. Paris, France: Organization for Economic Co-operation and Development (OECD).	1 - Covariates
432158	Administrative data	Protection At Birth (PAB) Against Tetanus with Tetanus Toxoid 1980-2017	World Health Organization (WHO). Protection At Birth (PAB) Against Tetanus with Tetanus Toxoid 1980-2017. Geneva, Switzerland: World Health Organization (WHO).	1 - Covariates
240107	Administrative data	South Africa Gross Domestic Product, Third Quarter 2012	Statistics South Africa. South Africa Gross Domestic Product, Third Quarter 2012. Pretoria, South Africa: Statistics South Africa, 2012.	1 - Covariates
244475	Administrative data	USDA Global Tobacco Database 1960-2005	U.S. Department of Agriculture (USDA). USDA Global Tobacco Database 1960-2005. Washington D.C. , United States: U.S. Department of Agriculture (USDA).	1 - Covariates
413763	Administrative data	WHO and UNICEF Reported Official Target Population, Number of Doses Administered and Official Coverage 1966-2018	United Nations Children's Fund (UNICEF), World Health Organization (WHO). WHO and UNICEF Reported Official Target Population, Number of Doses Administered and Official Coverage 1966-2018. Geneva, Switzerland: World Health Organization (WHO), 2019.	1 - Covariates
238445	Administrative data	WHO Global Health Observatory - Recorded Alcohol Per Capita Consumption 1960-1979	World Health Organization (WHO). WHO Global Health Observatory - Recorded Alcohol Per Capita Consumption 1960-1979. Geneva, Switzerland: World Health Organization (WHO).	1 - Covariates
238448	Administrative data	WHO Global Health Observatory - Recorded Alcohol Per Capita Consumption 1980-1999	World Health Organization (WHO). WHO Global Health Observatory - Recorded Alcohol Per Capita Consumption 1980-1999. Geneva, Switzerland: World Health Organization (WHO).	1 - Covariates
238452	Administrative data	WHO Global Health Observatory - Recorded Alcohol Per Capita Consumption 2000-2009 by country	World Health Organization (WHO). WHO Global Health Observatory - Recorded Alcohol Per Capita Consumption 2000-2009 by country. Geneva, Switzerland: World Health Organization (WHO).	1 - Covariates
151561	Administrative data	World Development Indicators - Vitamin A Supplementation Coverage Rate	World Bank. World Development Indicators - Vitamin A Supplementation Coverage Rate. Washington DC, United States of America: World Bank.	1 - Covariates

80287	Administrative data	World Mineral Production 2007-2011	British Geological Survey, Natural Environment Research Council. World Mineral Production 2007-2011. Nottingham, United Kingdom: British Geological Survey, Natural Environment Research Council, 2013.	1 - Covariates
26975	Administrative data	World Road Statistics 1963-1999	International Road Federation. World Road Statistics 1963-1999. Geneva, Switzerland: International Road Federation.	1 - Covariates
306491	Administrative data	World Road Statistics 2009 Database and World Road Statistics 2007	International Road Federation. World Road Statistics 2009 Database and World Road Statistics 2007.	1 - Covariates
242123	Administrative data	World Road Statistics 2015	International Road Federation. World Road Statistics 2015. Geneva, Switzerland: International Road Federation, 2015.	1 - Covariates
410939	Administrative data	World Road Statistics 2018	International Road Federation. World Road Statistics 2018. Geneva, Switzerland: International Road Federation, 2018.	1 - Covariates
43146	Census	South Africa Census 1996 - IPUMS	Central Statistical Service (South Africa), Minnesota Population Center. South Africa Census 1996 from the Integrated Public Use Microdata Series, International: [Machine-readable database]. Minneapolis: University of Minnesota.	1 - Covariates
43152	Census	South Africa Census 2001 - IPUMS	Statistics South Africa, Minnesota Population Center. South Africa Census 2001 from the Integrated Public Use Microdata Series, International: [Machine-readable database]. Minneapolis: University of Minnesota.	1 - Covariates
43158	Census	South Africa Community Survey 2007 - IPUMS	Statistics South Africa, Minnesota Population Center. South Africa Community Survey 2007 from the Integrated Public Use Microdata Series, International: [Machine-readable database]. Minneapolis: University of Minnesota.	1 - Covariates
12146	Census	South Africa Population and Housing Census 2011	Statistics South Africa. South Africa Population and Housing Census 2011.	1 - Covariates
227194	Census	South Africa Population and Housing Census 2011 - IPUMS	Minnesota Population Center, Statistics South Africa. South Africa Population and Housing Census 2011 from the Integrated Public Use Microdata Series, International: [Machine-readable database]. Minneapolis: University of Minnesota, 2015.	1 - Covariates
394317	Demographic surveillance	Child Health and Mortality Prevention Surveillance Network Program	Centers for Disease Control and Prevention (CDC), Child Health and Mortality Prevention Surveillance (CHAMPS) Network, Deloitte Consulting LLP, Emory Global Health Institute (EGHI), International Association of National Public Health Institutes (IANPHI), Task Force for Global Health, Emory University. Child Health and Mortality Prevention Surveillance (CHAMPS) Network Program.	1 - Covariates
93667	Demographic surveillance	Contributions of the Africa Centre Demographic Surveillance to the Community	Muhwava W. Contributions of the Africa Centre Demographic Surveillance to the Community. Umbiko. 2011; 12. 3-4.	1 - Covariates
11781	Demographic surveillance	South Africa - Agincourt Health and Socio-Demographic Surveillance System	MRC/Wits Rural Public Health and Health Transitions Research Unit (Agincourt), INDEPTH. South Africa - Agincourt Health and Socio-Demographic Surveillance System.	1 - Covariates
114558	Disease registry	Cancer in South Africa Full Report 2003	National Cancer Registry (South Africa). Cancer in South Africa Full Report 2003 . Johannesburg, South Africa: National Institute for Occupational Health (South Africa).	1 - Covariates
114560	Disease registry	Cancer in South Africa Full Report 2004	National Cancer Registry (South Africa). Cancer in South Africa Full Report 2004. Johannesburg, South Africa: National Institute for Occupational Health (South Africa).	1 - Covariates
264234	Disease registry	Cancer in South Africa Full Report 2011	National Cancer Registry (South Africa). Cancer in South Africa Full Report 2011. Johannesburg, South Africa: National Cancer Registry (South Africa).	1 - Covariates
199034	Disease registry	South Africa - PROMEC Cancer Registry 2003-2007 - CI5	South Africa - PROMEC Cancer Registry 2003-2007 - CI5.	1 - Covariates
113829	Disease registry	South Africa National Cancer Registry Incidence 2005	National Cancer Registry (South Africa). South Africa National Cancer Registry Incidence 2005. Johannesburg, South Africa: National Institute for Occupational Health (South Africa).	1 - Covariates
128995	Disease registry	South Africa PROMEC Cancer Registry Incidence 1998-2002	PROMEC Unit, South African Medical Research Council. South Africa PROMEC Cancer Registry Incidence 1998-2002.	1 - Covariates
160104	Disease registry	South African Birth Defects Surveillance Systems Data 1993-1997 - WHO	South African Birth Defects Surveillance Systems Data 1993-1997 - WHO	1 - Covariates
160059	Disease registry	South African Birth Defects Surveillance Systems Data 2001 - ICBDMS	South African Birth Defects Surveillance Systems Data 2001 - ICBDMS	1 - Covariates
268573	Disease registry	South African Birth Defects Surveillance Systems Data 2002 - ICBDMS	South African Birth Defects Surveillance Systems Data 2002 - ICBDMS	1 - Covariates

160135	Disease registry	South African Birth Defects Surveillance Systems Data 2003 - ICBDSR	South African Birth Defects Surveillance Systems Data 2003 - ICBDSR	1 - Covariates
424709	Environmental monitoring	Particulate Matter 2.5 and 10 Surface Monitor Station Expanded Database 2008-2017	Shaddick G, Thomas ML. Particulate Matter 2.5 and 10 Surface Monitor Station Expanded Database 2008-2017. [Unpublished].	1 - Covariates
133004	Environmental monitoring	South Africa Air Quality Information System Database	Department of Environmental Affairs (South Africa), South African Weather Service. South Africa Air Quality Information System Database. Pretoria, South Africa: South African Weather Service.	1 - Covariates
340570	Epi surveillance	Compendium of Short Reports on Selected Outbreaks in the WHO African Region, October 2017	World Health Organization Regional Office for Africa (WHO-AFRO). Compendium of Short Reports on Selected Outbreaks in the WHO African Region, October 2017. Brazaville, Congo: World Health Organization Regional Office for Africa (WHO-AFRO), 2017.	1 - Covariates
310754	Epi surveillance	Malaria Atlas Project Annual Parasite Incidence Database	Malaria Atlas Project. Malaria Atlas Project Annual Parasite Incidence Database.	1 - Covariates
311041	Epi surveillance	Notifiable Medical Conditions Report for South Africa November 2009	Department of Health (South Africa). Notifiable Medical Conditions Report for South Africa November 2009.	1 - Covariates
340584	Epi surveillance	Weekly Bulletins on Outbreaks and Other Emergencies 2017	World Health Organization Regional Office for Africa (WHO-AFRO). Weekly Bulletins on Outbreaks and Other Emergencies 2017. Brazaville, Congo: World Health Organization Regional Office for Africa (WHO-AFRO), 2017.	1 - Covariates
340865	Epi surveillance	Weekly Bulletins on Outbreaks and Other Emergencies 2018	World Health Organization Regional Office for Africa (WHO-AFRO). Weekly Bulletins on Outbreaks and Other Emergencies 2018. Brazaville , Congo: World Health Organization Regional Office for Africa (WHO-AFRO), 2018.	1 - Covariates
303730	Epi surveillance	WHO Global Health Observatory - Cholera: Number of Reported Deaths by Country	World Health Organization (WHO). WHO Global Health Observatory - Cholera: Number of Reported Deaths by Country. Geneva, Switzerland: World Health Organization (WHO).	1 - Covariates
310156	Estimate	Global Burden of Disease Study 2016 (GBD 2016) Covariates 1980-2016	Global Burden of Disease Collaborative Network. Global Burden of Disease Study 2016 (GBD 2016) Covariates 1980-2016. Seattle, United States: Institute for Health Metrics and Evaluation (IHME), 2017.	1 - Covariates
439727	Estimate	Global Health Spending 1995-2017	Global Burden of Disease Collaborative Network. Global Health Spending 1995-2017. Seattle, United States of America: Institute for Health Metrics and Evaluation (IHME), 2020.	1 - Covariates
419512	Estimate	Haemoglobin C (HbC) Allele Frequency Layer	Malaria Atlas Project. Haemoglobin C (HbC) Allele Frequency Layer. Oxford, United Kingdom: Malaria Atlas Project, 2013.	1 - Covariates
144369	Estimate	International Labour Organization Database (ILOSTAT) - Employment by Sex and Economic Activity	International Labour Organization (ILO). International Labour Organization Database (ILOSTAT) - Employment by Sex and Economic Activity. International Labour Organization (ILO).	1 - Covariates
144370	Estimate	International Labour Organization Database (ILOSTAT) - Employment by Sex and Occupation	International Labour Organization (ILO). International Labour Organization Database (ILOSTAT) - Employment by Sex and Occupation. International Labour Organization (ILO).	1 - Covariates
309568	Estimate	International Labour Organization Database (ILOSTAT) - Employment to Population Ratio by Sex and Age	International Labour Organization (ILO). International Labour Organization Database (ILOSTAT) - Employment to Population Ratio by Sex and Age. International Labour Organization (ILO).	1 - Covariates
447544	Estimate	Maddison Project Database 2018	Maddison Project Database, version 2018. Bolt, Jutta, Robert Inklaar, Herman de Jong and Jan Luiten van Zanden (2018), â€œRebasing â€˜Maddisonâ€™: new income comparisons and the shape of long-run economic developmentâ€• , Maddison Project Working paper 10	1 - Covariates
83626	Estimate	National Accounts Main Aggregates Database	United Nations Statistics Division (UNSD). National Accounts Main Aggregates Database. New York City, United States of America: United Nations Statistics Division (UNSD).	1 - Covariates
447532	Estimate	Penn World Table Version 9.1	Feenstra, Robert C., Robert Inklaar and Marcel P. Timmer (2015), The Next Generation of the Penn World Table" American Economic Review	105(10)
96480	Estimate	Sickle Cell and Thalassemias Prevalence Data, Personal Correspondence with David Weatherall	Weatherall D. Sickle Cell and Thalassemias Prevalence Data, Personal Correspondence with David Weatherall. [Unpublished].	1 - Covariates
419511	Estimate	Sickle Haemoglobin (Hbs) Allele Frequency Layer	Malaria Atlas Project. Sickle Haemoglobin (Hbs) Allele Frequency Layer. Oxford, United Kingdom: Malaria Atlas Project, 2013.	1 - Covariates
376304	Estimate	WHO Global Health Observatory - Hospital Bed Density Data by Country	World Health Organization (WHO). WHO Global Health Observatory - Hospital Bed Density Data by Country . Geneva, Switzerland: World Health Organization (WHO).	1 - Covariates
144319	Estimate	WHO PCT Databank - Schistosomiasis	World Health Organization (WHO). WHO PCT Databank - Schistosomiasis. Geneva, Switzerland: World Health Organization	1 - Covariates

			(WHO).	
144329	Estimate	WHO PCT Databank - Soil-transmitted Helminthiases	World Health Organization (WHO). WHO PCT Databank - Soil-transmitted Helminthiases. Geneva, Switzerland: World Health Organization (WHO).	1 - Covariates
447546	Estimate	World Development Indicators - Gross Domestic Product (GDP)	World Bank. World Development Indicators - Gross Domestic Product (GDP). Washington DC, United States of America: World Bank.	1 - Covariates
239152	Estimate	World Development Indicators - Hospital Beds (per 1,000 People)	World Bank. World Development Indicators - Hospital Beds (per 1,000 People). Washington DC, United States of America: World Bank.	1 - Covariates
69895	Estimate	World Economic Outlook Database	International Monetary Fund (IMF). World Economic Outlook Database. Washington, D.C., United States of America: International Monetary Fund (IMF).	1 - Covariates
135380	Event data	Armed Conflict Location and Event Dataset, Realtime - Robert S. Strauss Center	Climate Change and African Political Stability Project (CCAPS). Armed Conflict Location and Event Dataset, Realtime - Robert S. Strauss Center as referenced in Raleigh, Clionadh, Andrew Linke, Havard Hegre and Joakim Karlsen. 2010. Introducing ACLED-Armed Conflict Location and Event Data. Journal of Peace Research 47(5), 651-60.	1 - Covariates
327302	Event data	Battle Deaths Dataset Version 3.1, 2009	Peace Research Institute Oslo (PRIO). Battle Deaths Dataset Version 3.1, 2009. Oslo, Norway: Peace Research Institute Oslo (PRIO), 2009.	1 - Covariates
13769	Event data	EM-DAT: The OFDA/CRED International Disaster Database	Centre for Research on the Epidemiology of Disasters (CRED). EM-DAT: The OFDA/CRED International Disaster Database. Brussels, Belgium: Catholic University of Leuven.	1 - Covariates
427585	Event data	Floods Kill At Least 60 In Africa (Durban, South Africa)	de Greef K. Floods Kill At Least 60 In Africa (Durban, South Africa). New York Times [Internet]. 2019 Apr 24; Africa.	1 - Covariates
328214	Event data	Global Terrorism Database	National Consortium for the Study of Terrorism and Responses to Terrorism (START). Global Terrorism Database. College Park , MD, United States of America: University of Maryland, 2018.	1 - Covariates
283521	Event data	List of Terrorist Incidents in 2016	Wikipedia. List of Terrorist Incidents in 2016. San Francisco, United States of America: Wikipedia.	1 - Covariates
326896	Event data	UCDP Georeferenced Event Dataset, Version 17.1, 2016	Department of Peace and Conflict Research, Uppsala University. UCDP Georeferenced Event Dataset, Version 17.1, 2016. Uppsala, Sweden: Department of Peace and Conflict Research, Uppsala University, 2017.	1 - Covariates
90706	Financial record	WHO Global Health Expenditure Database	World Health Organization (WHO). WHO Global Health Expenditure Database - National Health Accounts Indicators. Geneva, Switzerland: World Health Organization (WHO).	1 - Covariates
318160	Legislation	Global Fortification Data Exchange - Chart: Year When Food Fortification Mandated	Food Fortification Initiative (United States), Global Alliance for Improved Nutrition (GAIN), Iodine Global Network (Canada), Micronutrient Forum (Canada). Global Fortification Data Exchange - Chart: Year When Food Fortification Mandated.	1 - Covariates
318159	Legislation	Global Fortification Data Exchange - Map: Legislation and Standards	Food Fortification Initiative (United States), Global Alliance for Improved Nutrition (GAIN), Iodine Global Network (Canada), Micronutrient Forum (Canada). Global Fortification Data Exchange - Map: Legislation and Standards.	1 - Covariates
268292	Report	Abortion Policies: A Global Review	United Nations Population Division. Abortion Policies: A Global Review. New York, United States of America: United Nations (UN), 2002.	1 - Covariates
306513	Report	BP Statistical Review of World Energy 2016	BP. BP Statistical Review of World Energy 2016. London, United Kingdom: BP, 2016.	1 - Covariates
93752	Report	Global Status Report on Road Safety 2009	World Health Organization (WHO). Global Status Report on Road Safety 2009. Geneva, Switzerland: World Health Organization (WHO), 2009.	1 - Covariates
346038	Report	Global Status Report on Road Safety 2013	World Health Organization (WHO). Global Status Report on Road Safety 2013. Geneva, Switzerland: World Health Organization (WHO), 2013.	1 - Covariates
346039	Report	Global Status Report on Road Safety 2015	World Health Organization (WHO). Global Status Report on Road Safety 2015. Geneva, Switzerland: World Health Organization (WHO), 2015.	1 - Covariates
401595	Report	Global Status Report on Road Safety 2018	World Health Organization (WHO). Global Status Report on Road Safety 2018. Geneva, Switzerland: World Health Organization (WHO), 2018.	1 - Covariates
143198	Report	HIV/AIDS Epidemiological Surveillance Update for the WHO African Region 2002	World Health Organization Regional Office for Africa (WHO-AFRO). HIV/AIDS Epidemiological Surveillance Update for the WHO African Region 2002. Brazaville , Congo: World Health Organization Regional Office for Africa (WHO-AFRO), 2003.	1 - Covariates

448075	Report	Leaded Petrol Phase-out: Global Status, March 2018	United Nations Environment Programme. Leaded Petrol Phase-out: Global Status, March 2018. Nairobi, Kenya: United Nations Environment Programme.	1 - Covariates
358284	Report	Overview of Leaded Gasoline and Sulfur Levels in Gasoline and Diesel	International Fuel Quality Center (IFQC). Overview of Leaded Gasoline and Sulfur Levels in Gasoline and Diesel . 2002.	1 - Covariates
157571	Report	Plasmodium Falciparum Infection in Children Aged 2-15 Years	South African Medical Research Council Health GIS Centre. Plasmodium Falciparum Infection in Children Aged 2-15 Years. Cape Town, South Africa: South African Medical Research Council, 2000.	1 - Covariates
311039	Report	Prevalence and Distribution of Malaria in South Africa - Part 1	Department of Health (South Africa). Prevalence and Distribution of Malaria in South Africa - Part 1. Pretoria, South Africa: Department of Health (South Africa), 1995.	1 - Covariates
157558	Report	South Africa Malaria Control Programme Monthly Report: Period 01-05-95 to 31-05-95	Department of Health (South Africa). South Africa Malaria Control Programme Monthly Report: Period 01-05-95 to 31-05-95. Jozini, South Africa: Department of Health (South Africa), 1995.	1 - Covariates
311040	Report	South Africa Millennium Development Goals 6: Combat HIV/AIDS, Malaria and Other Diseases 2015	Statistics South Africa. South Africa Millennium Development Goals 6: Combat HIV/AIDS, Malaria and Other Diseases 2015. Pretoria, South Africa: Statistics South Africa, 2015.	1 - Covariates
356498	Report	South Africa National Malaria Programme Performance Review 2009	Department of Health (South Africa). South Africa National Malaria Programme Performance Review 2009. 2009.	1 - Covariates
154459	Report	The Future of the Global Muslim Population	Pew Research Center. The Future of the Global Muslim Population. Washington, DC, United States: Pew Research Center, 2011.	1 - Covariates
124685	Report	WHO World Mental Health Surveys: Global Perspectives on the Epidemiology of Mental Disorders	World Health Organization (WHO). WHO World Mental Health Surveys: Global Perspectives on the Epidemiology of Mental Disorders. Cambridge, United Kingdom: Cambridge University Press, 2008.	1 - Covariates
268293	Report	World Abortion Policies 2007	United Nations Population Division. World Abortion Policies 2007. New York, United States of America: United Nations (UN), 2007.	1 - Covariates
268294	Report	World Abortion Policies 2013	United Nations Population Division. World Abortion Policies 2013. New York, United States of America: United Nations (UN), 2013.	1 - Covariates
80289	Report	World Drug Report 2012	United Nations Office on Drugs and Crime (UNODC). World Drug Report 2012. Vienna, Austria: United Nations Office on Drugs and Crime (UNODC), 2012.	1 - Covariates
264188	Report	World Malaria Report 2015	World Health Organization (WHO). World Malaria Report 2015. Geneva, Switzerland: World Health Organization (WHO), 2015.	1 - Covariates
284569	Report	World Malaria Report 2016	World Malaria Report 2016. Geneva: World Health Organization; 2016. Licence: CC BY-NC-SA 3.0 IGO.	1 - Covariates
343613	Report	World Malaria Report 2017	World Malaria Report 2017. Geneva: World Health Organization; 2017. Licence: CC BY-NC-SA 3.0 IGO.	1 - Covariates
142681	Scientific literature	A cross-sectional study of vascular risk factors in a rural South African population: data from the Southern African Stroke Prevention Initiative (SASPI)	Thorogood M, Connor M, Tollman S, Lewando Hundt G, Fowkes G, Marsh J. A cross-sectional study of vascular risk factors in a rural South African population: data from the Southern African Stroke Prevention Initiative (SASPI). BMC Public Health. 2007; 326.	1 - Covariates
278291	Scientific literature	A follow-up cross-sectional study of environmental lead exposure in early childhood in urban South Africa	Naicker N, Mathee A, Barnes B. A follow-up cross-sectional study of environmental lead exposure in early childhood in urban South Africa. S Afr Med J. 2013; 103(12): 935-8.	1 - Covariates
303208	Scientific literature	A High Burden of Hypertension in the Urban Black Population of Cape Town: The Cardiovascular Risk in Black South Africans (CRIBSA) Study	Peer N, Steyn K, Lombard C, Gwebushe N, Levitt N. A High Burden of Hypertension in the Urban Black Population of Cape Town: The Cardiovascular Risk in Black South Africans (CRIBSA) Study. PLoS One. 2013; 8(11): e78567.	1 - Covariates
146931	Scientific literature	A study of airborne radon levels in Paarl houses (South Africa) and associated source terms, using electret ion chambers and gamma-ray spectrometry	Lindsay R, Newman RT, Speelman WJ. A study of airborne radon levels in Paarl houses (South Africa) and associated source terms, using electret ion chambers and gamma-ray spectrometry. Appl Radiat Isot. 2008; 66(11): 1611-4.	1 - Covariates
131758	Scientific literature	A study of pediatric blood lead levels in a lead mining area in South Africa	Von Schirnding Y, Mathee A, Kibel M, Robertson P, Strauss N, Blignaut R. A study of pediatric blood lead levels in a lead mining area in South Africa. Environ Res. 2003; 93(3): 259-63.	1 - Covariates
131757	Scientific literature	A survey of blood lead levels among young Johannesburg school children	Mathee A, von Schirnding YER, Levin J, Ismail A, Huntley R, Cantrell A. A survey of blood lead levels among young Johannesburg school children. Environ Res. 2002; 90(3): 181-4.	1 - Covariates
214569	Scientific literature	Adherence to highly active antiretroviral therapy assessed by pharmacy claims predicts survival in	Nachega JB, Hislop M, Dowdy DW, Lo M, Omer SB, Regensberg L, Chaisson RE, Maartens G. Adherence to highly active antiretroviral therapy assessed by pharmacy claims predicts survival in HIV-	1 - Covariates

		HIV-infected South African adults	infected South African adults. J Acquir Immune Defic Syndr. 2006; 43(1): 78-84.	
229739	Scientific literature	Age differences in the prevalence and co-morbidity of DSM-IV major depressive episodes: results from the WHO World Mental Health Survey Initiative	Kessler RC, Birnbaum HG, Shahly V, Bromet E, Hwang I, McLaughlin KA, Sampson N, Andrade LH, de Girolamo G, Demyttenaere K, Haro JM, Karam AN, Kostyuchenko S, Kovess V, Lara C, Levinson D, Matschinger H, Nakane Y, Browne MO, Ormel J, Posada-Villa J, Sagar R, Stein DJ. Age differences in the prevalence and co-morbidity of DSM-IV major depressive episodes: results from the WHO World Mental Health Survey Initiative. Depress Anxiety. 2010; 27(4): 351â€“64.	1 - Covariates
267580	Scientific literature	Age in antiretroviral therapy programmes in South Africa: a retrospective, multicentre, observational cohort study	Cornell M, Johnson LF, Schomaker M, Tanser F, Maskew M, Wood R, Prozesky H, Giddy J, Stinson K, Egger M, Boulle A, Myer L, International Epidemiologic Databases to Evaluate AIDS-Southern Africa Collaboration. Age in antiretroviral therapy programmes in South Africa: a retrospective, multicentre, observational cohort study. Lancet HIV. 2015; 2(9): e368-375.	1 - Covariates
335046	Scientific literature	All-cause mortality in HIV-positive adults starting combination antiretroviral therapy: correcting for loss to follow-up	Anderegg N, Johnson LF, Zaniewski E, Althoff KN, Balestre E, Law M, Nash D, Shepherd BE, Yiannoutsos CT, Egger M, IeDEA, MeSH consortia. All-cause mortality in HIV-positive adults starting combination antiretroviral therapy: correcting for loss to follow-up. AIDS. 2017; 31 Suppl 1: S31â€“40.	1 - Covariates
255129	Scientific literature	Anaemia among the inhabitants of a rural area in northern Natal	Mayet FGH, Schutte CHJ, Reinach SG. Anaemia among the inhabitants of a rural area in northern Natal. S Afr Med J. 1985; 67(12): 458-62.	1 - Covariates
144642	Scientific literature	Anthropometric profile of a black population of the Cape Peninsula in South Africa	Steyn K, Bourne L, Jooste P, Fourie JM, Rossouw K, Lombard C. Anthropometric profile of a black population of the Cape Peninsula in South Africa. East Afr Med J. 1998; 75(1): 35-40.	1 - Covariates
209344	Scientific literature	Antiretroviral Treatment Outcomes amongst Older Adults in a Large Multicentre Cohort in South Africa	Fatti G, Mothibi E, Meintjes G, Grimwood A. Antiretroviral Treatment Outcomes amongst Older Adults in a Large Multicentre Cohort in South Africa. PLoS One. 2014; 9(6): e100273.	1 - Covariates
132104	Scientific literature	Assessment of age-related bone loss in normal South African women by means of the Hologic QDR 1000 system	Kalla AA, Fataar AB, Bewerunge L. Assessment of age-related bone loss in normal South African women by means of the Hologic QDR 1000 system. S Afr Med J. 1994; 84(7): 398-404.	1 - Covariates
279074	Scientific literature	Association Between Passive Smoking and Infection With Mycobacterium tuberculosis in Children	den Boon S, Verver S, Marais BJ, Enarson DA, Lombard CJ, Bateman ED, Irusen E, Jithoo A, Gie RP, Borgdorff MW, Beyers N. Association Between Passive Smoking and Infection With Mycobacterium tuberculosis in Children. Pediatrics. 2007; 119(4): 734-9.	1 - Covariates
214505	Scientific literature	Better antiretroviral therapy outcomes at primary healthcare facilities: an evaluation of three tiers of ART services in four South African provinces	Fatti G, Grimwood A, Bock P. Better antiretroviral therapy outcomes at primary healthcare facilities: an evaluation of three tiers of ART services in four South African provinces. PLoS One. 2010; 5(9): e12888.	1 - Covariates
131753	Scientific literature	Blood lead levels in a remote, unpolluted rural area in South Africa	Grobler SR, Rossouw RJ, Maresky LS. Blood lead levels in a remote, unpolluted rural area in South Africa. S Afr Med J. 1985; 68(5): 323-4.	1 - Covariates
131748	Scientific literature	Blood lead levels in preschool children in Cape Town	Deveaux P, Kibel MA, Dempster WS, Pocock F, Formenti K. Blood lead levels in preschool children in Cape Town. S Afr Med J. 1986; 69(7): 421-4.	1 - Covariates
402198	Scientific literature	Cancer of childhood in sub-Saharan Africa	Stefan C, Bray F, Ferlay J, Liu B, Maxwell Parkin D. Cancer of childhood in sub-Saharan Africa. ecancer. 2017; 11: 755.	1 - Covariates
214553	Scientific literature	CD4 count slope and mortality in HIV-infected patients on antiretroviral therapy: multicohort analysis from South Africa	Hoffmann CJ, Schomaker M, Fox MP, Mutevedzi P, Giddy J, Prozesky H, Wood R, Garone DB, Egger M, Boulle A, IeDEA Southern Africa Collaboration. CD4 count slope and mortality in HIV-infected patients on antiretroviral therapy: multicohort analysis from South Africa. J Acquir Immune Defic Syndr. 2013; 63(1): 34-41.	1 - Covariates
116206	Scientific literature	Changes in malaria morbidity and mortality in Mpumalanga Province, South Africa (2001-2009): a retrospective study	Ngomane L, de Jager C. Changes in malaria morbidity and mortality in Mpumalanga Province, South Africa (2001-2009): a retrospective study. Malar J. 2012; 19.	1 - Covariates
214519	Scientific literature	Changes in programmatic outcomes during 7 years of scale-up at a community-based antiretroviral treatment service in South Africa	Nglazi MD, Lawn SD, Kaplan R, Kranzer K, Orrell C, Wood R, Bekker L-G. Changes in programmatic outcomes during 7 years of scale-up at a community-based antiretroviral treatment service in South Africa. J Acquir Immune Defic Syndr. 2011; 56(1): e1-8.	1 - Covariates
131133	Scientific literature	Changing patterns of under- and over-nutrition in South African children-future risks of non-communicable	Jinabhai CC, Taylor M, Sullivan KR. Changing patterns of under- and over-nutrition in South African children-future risks of non-communicable diseases. Ann Trop Paediatr. 2005; 25(1): 3-15.	1 - Covariates

		diseases		
214535	Scientific literature	Changing predictors of mortality over time from cART start: implications for care	Hoffmann CJ, Fielding KL, Johnston V, Charalambous S, Innes C, Moore RD, Chaisson RE, Grant AD, Churchyard GJ. Changing predictors of mortality over time from cART start: implications for care. J Acquir Immune Defic Syndr. 2011; 58(3): 269-76.	1 - Covariates
279361	Scientific literature	Changing prevalence of tuberculosis infection with increasing age in high-burden townships in South Africa	Wood R, Liang H, Wu H, Middelkoop K, Oni T, Rangaka MX, Wilkinson RJ, Bekker L-G, Lawn SD. Changing prevalence of tuberculosis infection with increasing age in high-burden townships in South Africa. Int J Tuberc Lung Dis. 2010; 14(4): 406â€“412.	1 - Covariates
214556	Scientific literature	Comparison of tenofovir, zidovudine, or stavudine as part of first-line antiretroviral therapy in a resource-limited-setting: a cohort study	Velen K, Lewis JJ, Charalambous S, Grant AD, Churchyard GJ, Hoffmann CJ. Comparison of tenofovir, zidovudine, or stavudine as part of first-line antiretroviral therapy in a resource-limited-setting: a cohort study. PLoS One. 2013; 8(5): e64459.	1 - Covariates
112021	Scientific literature	Coronary risk factors in the coloured population of the Cape Peninsula	Steyn K, Jooste PL, Langenhoven ML, BenadÃ© AJ, Rossouw JE, Steyn M, Jordaan PC, Parry CD. Coronary risk factors in the coloured population of the Cape Peninsula. S Afr Med J. 1985; 67(16): 619-25.	1 - Covariates
257166	Scientific literature	Cystic Echinococcosis Endemicity Estimates	Cystic Echinococcosis Endemicity Estimates identified through systematic review and personal communication, as provided by GBD 2015 expert. [Unpublished].	1 - Covariates
279328	Scientific literature	Decreasing household contribution to TB transmission with age: a retrospective geographic analysis of young people in a South African township	Middelkoop K, Bekker L-G, Morrow C, Lee N, Wood R. Decreasing household contribution to TB transmission with age: a retrospective geographic analysis of young people in a South African township. BMC Infect Dis. 2014; 14(1): 1.	1 - Covariates
273000	Scientific literature	Delay and poor diagnosis of Down syndrome in KwaZulu-Natal, South Africa: A retrospective review of postnatal cytogenetic testing	Willoughby M, Aldous C, Patrick M, Kavonic S, Christianson A. Delay and poor diagnosis of Down syndrome in KwaZulu-Natal, South Africa: A retrospective review of postnatal cytogenetic testing. S Afr Med J. 2016; 106(6): 626-9.	1 - Covariates
271108	Scientific literature	Depression and associated factors in older adults in South Africa	Peltzer K, Phaswana-Mafuya N. Depression and associated factors in older adults in South Africa. Glob Health Action. 2013; 6: 1â€“9.	1 - Covariates
271129	Scientific literature	Depression, disability and functional status among community-dwelling older adults in South Africa: evidence from the first South African National Income Dynamics Study	Tomita A, Burns JK. Depression, disability and functional status among community-dwelling older adults in South Africa: evidence from the first South African National Income Dynamics Study. Int J Geriatr Psychiatry. 2013; 28(12): 1270â€“9.	1 - Covariates
247417	Scientific literature	Determinants of the components of arterial pressure among older adults--the role of anthropometric and clinical factors: a multi-continent study	Tyrovolas S, Koyanagi A, Garin N, Olaya B, Ayuso-Mateos JL, Miret M, Chatterji S, Tobiasz-Adamczyk B, Koskinen S, Leonardi M, Haro JM. Determinants of the components of arterial pressure among older adults--the role of anthropometric and clinical factors: a multi-continent study. Atherosclerosis. 2015; 238(2): 240â€“9.	1 - Covariates
112861	Scientific literature	Diabetes and other disorders of glycemia in a rural South African community: prevalence and associated risk factors	Motala AA, Esterhuizen T, Gouws E, Pirie FJ, Omar MA. Diabetes and other disorders of glycemia in a rural South African community: prevalence and associated risk factors. Diabetes Care. 2008; 31(9): 1783-8.	1 - Covariates
307272	Scientific literature	Dietary adequacies among South African adults in rural KwaZulu-Natal	Kolahdooz F, Spearing K, Sharma S. Dietary adequacies among South African adults in rural KwaZulu-Natal. PLoS One. 2013; 8(6:e67184): e67184.	1 - Covariates
293926	Scientific literature	Dietary iron overload as a risk factor for hepatocellular carcinoma in Black Africans	Mandishona E, MacPhail AP, Gordeuk VR, Kedda M-A, Paterson AC, Rouault TA, Kew MC. Dietary iron overload as a risk factor for hepatocellular carcinoma in Black Africans . Hepatology. 1998; 27(6): 1563-6.	1 - Covariates
131755	Scientific literature	Distribution of blood lead levels in schoolchildren in selected Cape Peninsula suburbs subsequent to reductions in petrol lead	Von Schirnding Y, Mathee A, Robertson P, Strauss N, Kibel M. Distribution of blood lead levels in schoolchildren in selected Cape Peninsula suburbs subsequent to reductions in petrol lead. S Afr Med J. 2001; 91(10): 870-2.	1 - Covariates
214508	Scientific literature	Early loss of HIV-infected patients on potent antiretroviral therapy programmes in lower-income countries	Brinkhof MWG, Dabis F, Myer L, Bangsberg DR, Boulle A, Nash D, Schechter M, Laurent C, Keiser O, May M, Sprinz E, Egger M, Anglaret X, ART-LINC, IeDEA. Early loss of HIV-infected patients on potent antiretroviral therapy programmes in lower-income countries. Bull World Health Organ. 2008; 86(7): 559-67.	1 - Covariates
131752	Scientific literature	Effect of the reduction of petrol lead on the blood lead levels of South Africans	Maresky LS, Grobler SR. Effect of the reduction of petrol lead on the blood lead levels of South Africans. Sci Total Environ. 1993; 136(1-2): 43-8.	1 - Covariates
131566	Scientific literature	Effects of Reducing Lead in Gasoline:? An Analysis of the International Experience	Thomas VM, Socolow RH, Fanelli JJ, Spiro TG. Effects of Reducing Lead in Gasoline:? An Analysis of the International Experience. Environ Sci Technol. 1999; 33(22): 3942-8.	1 - Covariates

122583	Scientific literature	Environmental lead exposure and socio-behavioural adjustment in the early teens: the birth to twenty cohort	Naicker N, Richter L, Mathee A, Becker P, Norris SA. Environmental lead exposure and socio-behavioural adjustment in the early teens: the birth to twenty cohort. Sci Total Environ. 2012; 120-5.	1 - Covariates
311721	Scientific literature	Epidemiology of malaria in South Africa: from control to elimination.(Epidemiology)(Report)	Maharaj R, Raman J, Morris N, Moonasar D, Durrheim DN, Seocharan I, Kruger P, Shandukani B, Kleinschmidt I. Epidemiology of malaria in South Africa: from control to elimination.(Epidemiology)(Report). S Afr Med J. 2013; 103(10): 779â€"83.	1 - Covariates
143220	Scientific literature	Epidemiology ofÂ syphilisÂ inÂ pregnancyÂ in rural South Africa: opportunities for control	Wilkinson D,Â Sach M,Â Connolly C. Epidemiology ofÂ syphilisÂ inÂ pregnancyÂ in rural South Africa: opportunities for control. Trop Med Int Health. 1997; 2(1): 57-62.	1 - Covariates
131759	Scientific literature	Estimating the burden of disease attributable to lead exposure in South Africa in 2000	Norman R, Mathee A, Barnes B, van der Merwe L, Bradshaw D. Estimating the burden of disease attributable to lead exposure in South Africa in 2000. S Afr Med J. 2007; 97(8 Pt 2): 773-80.	1 - Covariates
271323	Scientific literature	Estimation of national, regional and global prevalence of alcohol use during pregnancy and fetal alcohol syndrome: a systematic review and meta-analysis	Popova S, Lange S, Probst C, Gmel G, Rehm J, Centre for Addiction and Mental Health (Canada). Estimation of national, regional and global prevalence of alcohol use during pregnancy and fetal alcohol syndrome: a systematic review and meta-analysis. Lancet Glob Health. [Forthcoming]	1 - Covariates
247355	Scientific literature	Evaluation of waist-to-height ratio to predict 5 year cardiometabolic risk in sub-Saharan African adults	Ware LJ, Rennie KL, Kruger HS, Kruger IM, Greeff M, Fourie CMT, Huisman HW, Scheepers JDW, Uys AS, Kruger R, Van Rooyen JM, Schutte R, Schutte AE. Evaluation of waist-to-height ratio to predict 5 year cardiometabolic risk in sub-Saharan African adults. Nutr Metab Cardiovasc Dis. 2014; 24(8): 900â€"7.	1 - Covariates
311739	Scientific literature	Exploring the seasonality of reported treated malaria cases in Mpumalanga, South Africa	Silal SP, Barnes KI, Kok G, Mabuza A, Little F. Exploring the seasonality of reported treated malaria cases in Mpumalanga, South Africa. PLoS One. 2013; 8(10): e76640.	1 - Covariates
209317	Scientific literature	Factors associated with mortality in HIV-infected people in rural and urban South Africa	Otwombe KN, Petzold M, Modisenyane T, Martinson NA, Chirwa T. Factors associated with mortality in HIV-infected people in rural and urban South Africa. Glob Health Action. 2014; 25488.	1 - Covariates
214502	Scientific literature	Gender differences in survival among adult patients starting antiretroviral therapy in South Africa: a multicentre cohort study	Cornell M, Schomaker M, Garone DB, Giddy J, Hoffmann CJ, Lessells R, Maskew M, Prozesky H, Wood R, Johnson LF, Egger M, Boulle A, Myer L, International Epidemiologic Databases to Evaluate AIDS Southern Africa Collaboration. Gender differences in survival among adult patients starting antiretroviral therapy in South Africa: a multicentre cohort study. PLoS Med. 2012; 9(9): e1001304.	1 - Covariates
127450	Scientific literature	Glaucoma in Zulus: a population-based cross-sectional survey in a rural district in South Africa	Rotchford AP, Johnson GJ. Glaucoma in Zulus: a population-based cross-sectional survey in a rural district in South Africa. Arch Ophthalmol. 2002; 120(4): 471-8.	1 - Covariates
125860	Scientific literature	Hepatitis C virus infection in chronic liver disease in Natal	Soni PN, Tait DR, Gopaul W, Sathar MA, Simjee AE. Hepatitis C virus infection in chronic liver disease in Natal. S Afr Med J. 1996; 86(1): 80-3.	1 - Covariates
328837	Scientific literature	High diversity of Mycobacterium tuberculosis genotypes in South Africa and preponderance of mixed infections among ST53 isolates	Stavrum R, Mphahlele M, OvreÃ¥s K, Muthivhi T, Fourie PB, Weyer K, Grewal HMS. High diversity of Mycobacterium tuberculosis genotypes in South Africa and preponderance of mixed infections among ST53 isolates. J Clin Microbiol. 2009; 47(6): 1848â€"56.	1 - Covariates
280018	Scientific literature	Illicit drug use in South Africa: Findings from a 2008 national population-based survey	Peltzer K, Ramlagan S. Illicit drug use in South Africa: Findings from a 2008 national population-based survey. S Afr J Psychiatry. 2010; 16(1): a230.	1 - Covariates
110035	Scientific literature	Inherited haemoglobin variants in a South African population	Bird AR, Ellis P, Wood K, Mathew C, Karabus C. Inherited haemoglobin variants in a South African population. J Med Genet. 1987; 24(4): 215-9.	1 - Covariates
255133	Scientific literature	Iron, folate and vitamin B12 nutrition and anaemia in black preschool children in the northern Transvaal	Van der Westhuyzen J, Van Tonder SV, Gilbertson I, Metz J. Iron, folate and vitamin B12 nutrition and anaemia in black preschool children in the northern Transvaal. S Afr Med J. 1986; 70(3): 143-6.	1 - Covariates
131751	Scientific literature	Lead absorption in Cape children: a preliminary report	White NW, Dempster WS, Pocock F, Kibel MA. Lead absorption in Cape children: a preliminary report. S Afr Med J. 1982; 62(22): 799-802.	1 - Covariates
278441	Scientific literature	Lead exposure in young school children in South African subsistence fishing communities	Mathee A, Khan T, Naicker N, Kootbodien T, Naidoo S, Becker P. Lead exposure in young school children in South African subsistence fishing communities. Environ Res. 2013; 126: 179â€"83.	1 - Covariates
278439	Scientific literature	Lead in Paint: Three Decades Later and Still a Hazard for African Children?	Mathee A, RÃ¶llin H, Levin J, Naik I. Lead in Paint: Three Decades Later and Still a Hazard for African Children?. Environ Health Perspect. 2007; 115(3): 321â€"2.	1 - Covariates
131756	Scientific literature	Lead poisoning of children in Africa, II. Kwazulu/Natal, South Africa	Nriagu J, Jinabhai CC, Naidoo R, Coutsoudis A. Lead poisoning of children in Africa, II. Kwazulu/Natal, South Africa. Sci Total	1 - Covariates

			Environ. 1997; 197(1-3): 1-11.	
131749	Scientific literature	Lead reduction of petrol and blood lead concentrations of athletes	Grobler SR, Maresky LS, Kotze TJ. Lead reduction of petrol and blood lead concentrations of athletes. Arch Environ Health. 1992; 47(2): 139-42.	1 - Covariates
214900	Scientific literature	Long term outcomes of antiretroviral therapy in a large HIV/AIDS care clinic in urban South Africa: a prospective cohort study	Sanne IM, Westreich D, Macphail AP, Rubel D, Majuba P, Van Rie A. Long term outcomes of antiretroviral therapy in a large HIV/AIDS care clinic in urban South Africa: a prospective cohort study. J Int AIDS Soc. 2009; 38.	1 - Covariates
214515	Scientific literature	Long-term antiretroviral treatment outcomes in seven countries in the Caribbean	Koenig SP, Rodriguez LA, Bartholomew C, Edwards A, Carmichael TE, Barrow G, CabiÃ© A, Hunter R, Vasquez-Mora G, Quava-Jones A, Adomakoh N, Peter Figueroa J, Liautaud B, Torres M, Pape JW. Long-term antiretroviral treatment outcomes in seven countries in the Caribbean. J Acquir Immune Defic Syndr. 2012; 59(4): e60-71.	1 - Covariates
214531	Scientific literature	Low haemoglobin predicts early mortality among adults starting antiretroviral therapy in an HIV care programme in South Africa: a cohort study	Russell EC, Charalambous S, Pemba L, Churchyard GJ, Grant AD, Fielding K. Low haemoglobin predicts early mortality among adults starting antiretroviral therapy in an HIV care programme in South Africa: a cohort study. BMC Public Health. 2010; 433.	1 - Covariates
311749	Scientific literature	Malaria control in South Africa 2000-2010: beyond MDG6	Moonasar D, Nuthulaganti T, Kruger P, Mabuza A, Rasiswi E, Benson F, Maharaj R. Malaria control in South Africa 2000-2010: beyond MDG6. Malar J. 2012; 11: 294.	1 - Covariates
131750	Scientific literature	Maternal and fetal blood lead levels	Karimi PG, Moodley J, Jinabhai CC, Nriagu J. Maternal and fetal blood lead levels. S Afr Med J. 1999; 89(6): 676-9.	1 - Covariates
358489	Scientific literature	Minimal diversity of drug-resistant Mycobacterium tuberculosis strains, South Africa	Gandhi NR, Brust JCM, Moodley P, Weissman D, Heo M, Ning Y, Moll AP, Friedland GH, Sturm AW, Shah NS. Minimal diversity of drug-resistant Mycobacterium tuberculosis strains, South Africa. Emerg Infect Dis. 2014; 20(3): 426â€“33.	1 - Covariates
93645	Scientific literature	Mortality and loss to follow-up among HAART initiators in rural South Africa	MacPherson P, Moshabela M, Martinson N, Pronyk P. Mortality and loss to follow-up among HAART initiators in rural South Africa. Trans R Soc Trop Med Hyg. 2009; 103(6): 588-93.	1 - Covariates
214568	Scientific literature	Mortality and morbidity among HIV type-1-infected patients during the first 5 years of a multicountry HIV workplace programme in Africa	Van der Borght SF, Clevenbergh P, Rijckborst H, Nsalou P, Onyia N, Lange JM, de Wit TFR, Van der Loeff MFS. Mortality and morbidity among HIV type-1-infected patients during the first 5 years of a multicountry HIV workplace programme in Africa. Antivir Ther. 2009; 14(1): 63-74.	1 - Covariates
214562	Scientific literature	Mortality during the first year of potent antiretroviral therapy in HIV-1-infected patients in 7 sites throughout Latin America and the Caribbean	Tuboi SH, Schechter M, McGowan CC, Cesar C, Krolewiecki A, Cahn P, Wolff M, Pape JW, Padgett D, Madero JS, Gotuzzo E, Masys DR, Shepherd BE. Mortality during the first year of potent antiretroviral therapy in HIV-1-infected patients in 7 sites throughout Latin America and the Caribbean. J Acquir Immune Defic Syndr. 2009; 51(5): 615-23.	1 - Covariates
209349	Scientific literature	Mortality in patients with HIV-1 infection starting antiretroviral therapy in South Africa, Europe, or North America: a collaborative analysis of prospective studies	Boulle A, Schomaker M, May MT, Hogg RS, Shepherd BE, Monge S, Keiser O, Lampe FC, Giddy J, Ndirangu J, Garone D, Fox M, Ingle SM, Reiss P, Dabis F, Costagliola D, Castagna A, Ehren K, Campbell C, Gill MJ, Saag M, Justice AC, Guest J, Crane HM, Egger M, Sterne JAC. Mortality in patients with HIV-1 infection starting antiretroviral therapy in South Africa, Europe, or North America: a collaborative analysis of prospective studies. PLoS Med. 2014; 11(9): e1001718.	1 - Covariates
156877	Scientific literature	Mortality in women of reproductive age in rural South Africa	Nabukalu D, Klipstein-Grobusch K, Herbst K, Newell M-L. Mortality in women of reproductive age in rural South Africa. Glob Health Action. 2013; 22834.	1 - Covariates
214567	Scientific literature	Mortality of HIV-infected patients starting antiretroviral therapy in sub-Saharan Africa: comparison with HIV-unrelated mortality	Brinkhof MWG, Boulle A, Weigel R, Messou E, Mathers C, Orrell C, Dabis F, Pascoe M, Egger M, International Epidemiological Databases to Evaluate AIDS (IeDEA). Mortality of HIV-infected patients starting antiretroviral therapy in sub-Saharan Africa: comparison with HIV-unrelated mortality. PLoS Med. 2009; 6(4): e1000066.	1 - Covariates
329696	Scientific literature	Mycobacterium tuberculosis transmission is not related to household genotype in a setting of high endemicity	Marais BJ, Hesseling AC, Schaaf HS, Gie RP, van Helden PD, Warren RM. Mycobacterium tuberculosis transmission is not related to household genotype in a setting of high endemicity. J Clin Microbiol. 2009; 47(5): 1338â€“43.	1 - Covariates
279493	Scientific literature	No decrease in annual risk of tuberculosis infection in endemic area in Cape Town, South Africa	Kritzinger FE, den Boon S, Verver S, Enarson DA, Lombard CJ, Borgdorff MW, Gie RP, Beyers N. No decrease in annual risk of tuberculosis infection in endemic area in Cape Town, South Africa. Trop Med Int Health. 2009; 14(2): 136â€“42.	1 - Covariates
419398	Scientific literature	Nurses at risk for occupationally acquired blood-borne virus infection	Mosendane T, Kew MC, Osih R, Mahomed A. Nurses at risk for occupationally acquired blood-borne virus infection at a South	1 - Covariates

		at a South African academic hospital	African academic hospital. S Afr Med J. 2012; 102(3 Pt 1): 1536.	
144174	Scientific literature	Nutritional status and dietary intakes of children aged 2-5 years and their caregivers in a rural South African community	Faber M, Jogessar VB, BenadÃ© AJ. Nutritional status and dietary intakes of children aged 2-5 years and their caregivers in a rural South African community. Int J Food Sci Nutr. 2001; 52(5): 401-11.	1 - Covariates
142680	Scientific literature	Obesity and overweight in South African primary school children -- the Health of the Nation Study	Armstrong MEG, Lambert MI, Sharwood KA, Lambert EV. Obesity and overweight in South African primary school children -- the Health of the Nation Study. S Afr Med J. 2006; 96(5): 439-44.	1 - Covariates
128455	Scientific literature	Occurrence and clinical implications of red-cell glucose-6-phosphate dehydrogenase deficiency in South African racial groups	Bernstein RE. Occurrence and clinical implications of red-cell glucose-6-phosphate dehydrogenase deficiency in South African racial groups. S Afr Med J. 1963; 447-51.	1 - Covariates
143219	Scientific literature	On-site rapid antenatal syphilis screening with an immunochromatographic strip improves case detection and treatment in rural South African clinics	Bronzan RN,Â Mwesigwa-Kayongo DC,Â Narkunas D,Â Schmid GP,Â Neilsen GA,Â Ballard RC,Â Karuhije P,Â Ddamba J,Â Nombekela E,Â Hoyi G,Â Dlali P,Â Makwedini N,Fehler HG,Â Blandford JM,Â Ryan C. On-site rapid antenatal syphilis screening with an immunochromatographic strip improves case detection and treatment in rural South African clinics. Sex Transm Dis. 2007; 34(7 Suppl): S55-60.	1 - Covariates
214903	Scientific literature	Outcomes of antiretroviral treatment in programmes with and without routine viral load monitoring in Southern Africa	Keiser O, Chi BH, Gsponer T, Boulle A, Orrell C, Phiri S, Maxwell N, Maskew M, Prozesky H, Fox MP, Westfall A, Egger M, IeDEA Southern Africa Collaboration. Outcomes of antiretroviral treatment in programmes with and without routine viral load monitoring in Southern Africa. AIDS. 2011; 25(14): 1761-9.	1 - Covariates
214911	Scientific literature	Outcomes of antiretroviral treatment program in Ethiopia: retention of patients in care is a major challenge and varies across health facilities	Assefa Y, Kiflie A, Tesfaye D, Mariam DH, Kloos H, Edwin W, Laga M, Van Damme W. Outcomes of antiretroviral treatment program in Ethiopia: retention of patients in care is a major challenge and varies across health facilities. BMC Health Serv Res. 2011; 81.	1 - Covariates
214514	Scientific literature	Outcomes of antiretroviral treatment programs in rural Southern Africa	Wandeler G, Keiser O, Pfeiffer K, Pestilli S, Fritz C, Labhardt ND, Mbofana F, Mudyiradima R, Emmel J, Egger M, Ehmer J, SolidarMed ART program and IeDEA-Southern Africa. Outcomes of antiretroviral treatment programs in rural Southern Africa. J Acquir Immune Defic Syndr. 2012; 59(2): e9-16.	1 - Covariates
144701	Scientific literature	Physical activity, change in blood pressure and predictors of mortality in older South Africans--a 2-year follow-up study	Charlton KE, Lambert EV, Kreft J. Physical activity, change in blood pressure and predictors of mortality in older South Africans--a 2-year follow-up study. S Afr Med J. 1997; 87(9): 1124-30.	1 - Covariates
278257	Scientific literature	Prenatal exposure to manganese in South African coastal communities	Rollin HB, Kootbodien T, Theodorou P, Odland JO. Prenatal exposure to manganese in South African coastal communities. Environ Sci Process Impacts. 2014; 16(8): 1903-12.	1 - Covariates
271104	Scientific literature	Prevalence and factors associated with depressive symptoms among young women and men in the Eastern Cape Province, South Africa	Nduna M, Jewkes RK, Dunkle KL, Jama Shai NP, Colman I. Prevalence and factors associated with depressive symptoms among young women and men in the Eastern Cape Province, South Africa. J Child Adolesc Ment Health. 2013; 25(1): 43â€“54.	1 - Covariates
124311	Scientific literature	Prevalence of cardiovascular diseases and associated risk factors in a rural black population of South Africa	Alberts M, Urdal P, Steyn K, Stensvold I, Tverdal A, Nel JH, Steyn NP. Prevalence of cardiovascular diseases and associated risk factors in a rural black population of South Africa. Eur J Prev Cardiol. 2005; 12(4): 347-54.	1 - Covariates
120216	Scientific literature	Prevalence of minor psychiatric disorders in an adult African rural community in South Africa	Bhagwanjee A PA, Paruk Z, Petersen I, Subedar H. Prevalence of minor psychiatric disorders in an adult African rural community in South Africa. Psychol Med. 1998; 28(5): 1137-47.	1 - Covariates
134231	Scientific literature	Prevalence of vaginitis, syphilis and HIV infection in women in the Orange Free State	Cronje HS, Joubert G, Muir A, Chapman RD, Divall P, Bam RH. Prevalence of vaginitis, syphilis and HIV infection in women in the Orange Free State. S Afr Med J. 1994; 84(9): 602-5.	1 - Covariates
138472	Scientific literature	Prevalence, Causes and Socio-Economic Determinants of Vision Loss in Cape Town, South Africa. Atashili J, editor	Cockburn N, Steven D, Lecuona K, Joubert F, Rogers G, Cook C, Polack S. Prevalence, Causes and Socio-Economic Determinants of Vision Loss in Cape Town, South Africa. Atashili J, editor. PLoS One. 2012; 7(2): e30718.	1 - Covariates
128454	Scientific literature	Primaquine-sensitivity of red cells in various races in Southern Africa	Charlton RW, Bothwell TH. Primaquine-sensitivity of red cells in various races in Southern Africa. BMJ. 1961; 1(5230): 941-4.	1 - Covariates
214510	Scientific literature	Prognosis of patients with HIV-1 infection starting antiretroviral therapy in sub-Saharan Africa: a collaborative analysis of scale-up programmes	May M, Boulle A, Phiri S, Messou E, Myer L, Wood R, Keiser O, Sterne JAC, Dabis F, Egger M, IeDEA Southern Africa and West Africa. Prognosis of patients with HIV-1 infection starting antiretroviral therapy in sub-Saharan Africa: a collaborative analysis of scale-up programmes. Lancet. 2010; 376(9739): 449-57.	1 - Covariates
342267	Scientific literature	Psychoactive substance use among young people: findings of a multi-center study in three African countries	Nkowane, M.A., Rocha-Silva, L., Saxena, S., Mbatia, J., Ndubani, P. and Weir-Smith, G. Psychoactive substance use among young people: findings of a multi-center study in three African countries.	1 - Covariates

			Contemp Drug Probs. 2004; 31(2): 329-356.	
303385	Scientific literature	Rapid assessment of avoidable blindness in the northern eThekwini district of KwaZulu-Natal Province, South Africa	Govender P, Ramson P, Visser L, Naidoo KS. Rapid assessment of avoidable blindness in the northern eThekwini district of KwaZulu-Natal Province, South Africa. Afr Vis Eye Health. 2015; 74(1): 1-7.	1 - Covariates
93598	Scientific literature	Rates and causes of child mortality in an area of high HIV prevalence in rural South Africa	Garrib A, Jaffar S, Knight S, Bradshaw D, Bennish ML. Rates and causes of child mortality in an area of high HIV prevalence in rural South Africa. Trop Med Int Health. 2006; 11(12): 1841-8.	1 - Covariates
214541	Scientific literature	Reducing mortality with cotrimoxazole preventive therapy at initiation of antiretroviral therapy in South Africa	Hoffmann CJ, Fielding KL, Charalambous S, Innes C, Chaisson RE, Grant AD, Churchyard GJ. Reducing mortality with cotrimoxazole preventive therapy at initiation of antiretroviral therapy in South Africa. AIDS. 2010; 24(11): 1709-16.	1 - Covariates
131754	Scientific literature	Reductions in blood lead levels among school children following the introduction of unleaded petrol in South Africa	Mathee A, RÃ¶llin H, von Schirnding Y, Levin J, Naik I. Reductions in blood lead levels among school children following the introduction of unleaded petrol in South Africa. Environ Res. 2006; 100(3): 319-22.	1 - Covariates
354012	Scientific literature	Reviewing South Africaâ€™s malaria elimination strategy (2012â€“2018): progress, challenges and priorities	Raman J, Morris N, Frean J, Brooke B, Blumberg L, Kruger P, Mabusa A, Raswiswi E, Shandukani B, Misani E, Groepe M-A, Moonasar D. Reviewing South Africaâ€™s malaria elimination strategy (2012â€“2018): progress, challenges and priorities. Malar J. 2016; 15(1).	1 - Covariates
341237	Scientific literature	Risk-taking behaviour of Cape Peninsula high-school students. Part V. Drug use	Flisher AJ, Ziervogel CF, Chalton DO, Leger PH, Robertson BA. Risk-taking behaviour of Cape Peninsula high-school students. Part V. Drug use. S Afr Med J. 1993; 83(7): 483â€“5.	1 - Covariates
214532	Scientific literature	Scale-up of a decentralized HIV treatment programme in rural KwaZulu-Natal, South Africa: does rapid expansion affect patient outcomes?	Mutevedzi PC, Lessells RJ, Heller T, BÃ¤rnighausen T, Cooke GS, Newell M-L. Scale-up of a decentralized HIV treatment programme in rural KwaZulu-Natal, South Africa: does rapid expansion affect patient outcomes?. Bull World Health Organ. 2010; 88(8): 593-600.	1 - Covariates
279855	Scientific literature	SCHISTOSOMA-HAEMATOBIUM IN TRANSKEI - A PRELIMINARY SURVEY CONDUCTED IN THE NGQELENI DISTRICT	MQOQI N, DYE A. SCHISTOSOMA-HAEMATOBIUM IN TRANSKEI - A PRELIMINARY SURVEY CONDUCTED IN THE NGQELENI DISTRICT. S Afr J Sci. 1992; 88(8): 445â€“7.	1 - Covariates
279326	Scientific literature	Screening for TB in high school adolescents in a high burden setting in South Africa	Mahomed H, Ehrlich R, Hawkridge T, Hatherill M, Geiter L, Kafaar F, Abrahams DA, Mulenga H, Tameris M, Geldenhuys H, Hanekom WA, Verver S, Hussey GD. Screening for TB in high school adolescents in a high burden setting in South Africa. Tuberc (Edinb). 2013; 93(3): 357â€“62.	1 - Covariates
218643	Scientific literature	Serological screening for sexually transmitted infections in pregnancy: is there any value in re-screening for HIV and syphilis at the time of delivery?	Qolohle DC, Hoosen AA, Moodley J, Smith AN, Mlisana KP. Serological screening for sexually transmitted infections in pregnancy: is there any value in re-screening for HIV and syphilis at the time of delivery?. Genitourin Med. 1995; 71(2): 65-7.	1 - Covariates
214517	Scientific literature	Seven-year experience of a primary care antiretroviral treatment programme in Khayelitsha, South Africa	Boulle A, Van Cutsem G, Hilderbrand K, Cragg C, Abrahams M, Mathee S, Ford N, Knight L, Osler M, Myers J, Goemaere E, Coetzee D, Maartens G. Seven-year experience of a primary care antiretroviral treatment programme in Khayelitsha, South Africa. AIDS. 2010; 24(4): 563-72.	1 - Covariates
264304	Scientific literature	Sex differences in obesity incidence: 20-year prospective cohort in South Africa	Lundeen EA, Norris SA, Adair LS, Richter LM, Stein AD. Sex differences in obesity incidence: 20-year prospective cohort in South Africa. Pediatr Obes. 2016; 11(1): 75â€“80.	1 - Covariates
143218	Scientific literature	Sexually transmitted diseases in South Africa	Pham-Kanter GB, Steinberg MH, Ballard RC. Sexually transmitted diseases in South Africa. Genitourin Med. 1996; 72(3): 160-71.	1 - Covariates
294625	Scientific literature	SODIUM INTAKES AROUND THE WORLD [Internet]. Paris: World Health Organization;	Brown I, Elliott P. SODIUM INTAKES AROUND THE WORLD [Internet]. Paris: World Health Organization;. 2006; 85.	1 - Covariates
342293	Scientific literature	Substance use Among Rural Secondary School Pupils in the Northern Province, South Africa	Lily Peltzer K. Substance use Among Rural Secondary School Pupils in the Northern Province, South Africa. J Psychol Afr. 1999; 9(1): 58â€“65.	1 - Covariates
342295	Scientific literature	Substance Use Among Urban Secondary School Pupils in the Northern Province, South Africa	Peltzer K, Cherlan M, Cherlan L. Substance Use Among Urban Secondary School Pupils in the Northern Province, South Africa. South Afr J Child Adolesc Ment Health. 1999; 11(1): 49â€“55.	1 - Covariates
341239	Scientific literature	Substance use by adolescents in Cape Town: prevalence and correlates	Flisher AJ, Parry CDH, Evans J, Muller M, Lombard C. Substance use by adolescents in Cape Town: prevalence and correlates. J Adolesc Health. 2003; 32(1): 58â€“65.	1 - Covariates
213399	Scientific literature	Survival from HIV-1 seroconversion in Southern Africa: a retrospective cohort study in nearly 2000 gold-	Glynn JR, Sonnenberg P, Nelson G, Bester A, Shearer S, Murray J. Survival from HIV-1 seroconversion in Southern Africa: a retrospective cohort study in nearly 2000 gold-miners over 10 years	1 - Covariates

		miners over 10 years of follow-up	of follow-up. AIDS. 2007; 21(5): 625-32.	
138327	Scientific literature	Syphilis in pregnancy--prevalence at different levels of health care in Durban	Devjee J, Moodley J, Singh M. Syphilis in pregnancy--prevalence at different levels of health care in Durban. S Afr Med J. 2006; 96(11): 1182-4.	1 - Covariates
143221	Scientific literature	Syphilis in pregnant patients and their offspring	Bam RH,Â CronjÃ© HS,Â Muir A,Â Griessel DJ,Â Hoek BB. Syphilis in pregnant patients and their offspring. Int J Gynaecol Obstet. 1994; 44(2): 113-8.	1 - Covariates
214901	Scientific literature	Temporal changes in programme outcomes among adult patients initiating antiretroviral therapy across South Africa, 2002-2007	Cornell M, Grimsrud A, Fairall L, Fox MP, van Cutsem G, Giddy J, Wood R, Prozesky H, Mohapi L, Graber C, Egger M, Boulle A, Myer L, International Epidemiologic Databases to Evaluate AIDS Southern Africa (IeDEA-SA) Collaboration. Temporal changes in programme outcomes among adult patients initiating antiretroviral therapy across South Africa, 2002-2007. AIDS. 2010; 24(14): 2263-70.	1 - Covariates
336701	Scientific literature	The association between body composition, 25(OH)D, and PTH and bone mineral density in black African and Asian Indian population groups	George JA, Micklesfield LK, Norris SA, Crowther NJ. The association between body composition, 25(OH)D, and PTH and bone mineral density in black African and Asian Indian population groups. J Clin Endocrinol Metab. 2014; 99(6): 2146â€“54.	1 - Covariates
142679	Scientific literature	The determinants of overweight and obesity among 10- to 15-year-old schoolchildren in the North West Province, South Africa - the THUSA BANA (Transition and Health during Urbanisation of South Africans; BANA, children) study	Kruger R, Kruger HS, Macintyre UE. The determinants of overweight and obesity among 10- to 15-year-old schoolchildren in the North West Province, South Africa - the THUSA BANA (Transition and Health during Urbanisation of South Africans; BANA, children) study. Public Health Nutr. 2006; 9(3): 351-8.	1 - Covariates
144649	Scientific literature	The epidemic of obesity in South Africa: a study in a disadvantaged community	Temple NJ, Steyn K, Hoffman M, Levitt NS, Lombard CJ. The epidemic of obesity in South Africa: a study in a disadvantaged community. Ethn Dis. 2001; 11(3): 431-7.	1 - Covariates
126026	Scientific literature	The epidemiology of major depression in South Africa: results from the South African stress and health study	Tomlinson M, Grimsrud AT, Stein DJ, Williams DR, Myer L. The epidemiology of major depression in South Africa: results from the South African stress and health study. S Afr Med J. 2009; 99(5 Pt 2): 367-73.	1 - Covariates
294028	Scientific literature	The Epidemiology of Schistosomiasis among Zulu Children in a Rural District in South Africa: Determining Appropriate Community-Based Diagnostic Tools	Taylor M. The Epidemiology of Schistosomiasis among Zulu Children in a Rural District in South Africa: Determining Appropriate Community-Based Diagnostic Tools. South Afr J Epidemiol Infect. 2004; 19: 90-95.	1 - Covariates
311726	Scientific literature	The feasibility of malaria elimination in South Africa	Maharaj Rajendra, Morris Natashia, Seocharan Ishen, Kruger Philip, Moonasar Devanand, Mabuza Aaron, Raswiswi Eric, Raman Jaishree. The feasibility of malaria elimination in South Africa. Malar J. 2012; 11(1): 423.	1 - Covariates
214554	Scientific literature	The interplay between CD4 cell count, viral load suppression and duration of antiretroviral therapy on mortality in a resource-limited setting	Brennan AT, Maskew M, Sanne I, Fox MP. The interplay between CD4 cell count, viral load suppression and duration of antiretroviral therapy on mortality in a resource-limited setting. Trop Med Int Health. 2013; 18(5): 619-31.	1 - Covariates
144167	Scientific literature	The nutritional status of a rural community in KwaZulu-Natal, South Africa: the Ndunakazi project	Oelofse A, Faber M, BenadÃ© JG, BenadÃ© AJ, Kenoyer DG. The nutritional status of a rural community in KwaZulu-Natal, South Africa: the Ndunakazi project. Cent Afr J Med. 1999; 45(1): 14-9.	1 - Covariates
112860	Scientific literature	The prevalence and identification of risk factors for NIDDM in urban Africans in Cape Town, South Africa	Levitt NS, Katzenellenbogen JM, Bradshaw D, Hoffman MN, Bonnici F. The prevalence and identification of risk factors for NIDDM in urban Africans in Cape Town, South Africa. Diabetes Care. 1993; 16(4): 601-7.	1 - Covariates
420038	Scientific literature	The prevalence and transmission of hepatitis B virus infection in urban, rural and institutionalized black children of Natal/KwaZulu, South Africa	Abdool Karim SS, Coovadia HM, Windsor IM, Thejpal R, van den Ende J, Fouche A. The prevalence and transmission of hepatitis B virus infection in urban, rural and institutionalized black children of Natal/KwaZulu, South Africa. Int J Epidemiol. 1988; 17(1): 16873.	1 - Covariates
144959	Scientific literature	The prevalence of diabetes mellitus and impaired glucose tolerance in a group of urban South African blacks	Omar MA, Seedat MA, Motala AA, Dyer RB, Becker P. The prevalence of diabetes mellitus and impaired glucose tolerance in a group of urban South African blacks. S Afr Med J. 1993; 83(9): 641-3.	1 - Covariates
354782	Scientific literature	The prevalence of Plasmodium falciparum in sub-Saharan Africa since 1900	Snow RW, Sartorius B, Kyalo D, Maina J, Amratia P, Mundia CW, Bejon P, Noor AM. The prevalence of Plasmodium falciparum in sub-Saharan Africa since 1900. Nature. 2017; 550(7677): 515â€“8.	1 - Covariates
127448	Scientific literature	The prevalence of primary angle closure glaucoma and open angle glaucoma in Mamre, western Cape, South Africa	Salmon JF, Mermoud A, Ivey A, Swanevelder SA, Hoffman M. The prevalence of primary angle closure glaucoma and open angle glaucoma in Mamre, western Cape, South Africa. Arch Ophthalmol. 1993; 111(9): 1263-9.	1 - Covariates

294027	Scientific literature	The prevalence of Schistosoma haematobium in school children in Bizana District of Transkei	Mayanja FJLB, Edginton ME. The prevalence of Schistosoma haematobium in school children in Bizana District of Transkei. South Afr J Epidemiol Infect. 1992; 7: 20-21.	1 - Covariates
264260	Scientific literature	The relationship between BMI and dietary intake of primary school children from a rural area of South Africa: The Ellisras longitudinal study	Van Den Ende C, Twisk JWR, Monyeki KD. The relationship between BMI and dietary intake of primary school children from a rural area of South Africa: The Ellisras longitudinal study. Am J Hum Biol. 2014; 26(5): 701â€"6.	1 - Covariates
335324	Scientific literature	The Relationship between Physical Activity and Plasma Glucose Level amongst Ellisras Rural Young Adult Males and Females: Ellisras Longitudinal Study	Matshipi M, Monyeki KD, Kemper H. The Relationship between Physical Activity and Plasma Glucose Level amongst Ellisras Rural Young Adult Males and Females: Ellisras Longitudinal Study. Int J Environ Res Public Health. 2017; 14(2).	1 - Covariates
283258	Scientific literature	The relative roles of hepatitis B and C viruses in the etiology of hepatocellular carcinoma in southern African blacks	Kew MC, Yu MC, Kedda MA, Coppin A, Sarkin A, Hodkinson J. The relative roles of hepatitis B and C viruses in the etiology of hepatocellular carcinoma in southern African blacks. Gastroenterology. 1997; 112(1): 184-7.	1 - Covariates
267694	Scientific literature	Treatment outcomes of HIV-positive patients on first-line antiretroviral therapy in private versus public HIV clinics in Johannesburg, South Africa	Moyo F, Chasela C, Brennan AT, Ebrahim O, Sanne IM, Long L, Evans D. Treatment outcomes of HIV-positive patients on first-line antiretroviral therapy in private versus public HIV clinics in Johannesburg, South Africa. Clin Epidemiol. 2016; 8: 37â€"47.	1 - Covariates
141078	Scientific literature	TreatmentÂ ofÂ maternalÂ syphilisÂ inÂ ruralÂ South Africa: effect of multiple doses of benzathine penicillin on pregnancy loss	Myer L,Â Abdool Karim SS,Â Lombard C,Â Wilkinson D. TreatmentÂ ofÂ maternalÂ syphilisÂ inÂ ruralÂ South Africa: effect of multiple doses of benzathine penicillin on pregnancy loss. Trop Med Int Health. 2004; 9(11): 1216-21.	1 - Covariates
335001	Scientific literature	Twelve-year mortality in adults initiating antiretroviral therapy in South Africa	Cornell M, Johnson LF, Wood R, Tanser F, Fox MP, Prozesky H, Schomaker M, Egger M, Davies MA, Boulle A, International Epidemiology Databases to Evaluate AIDS-Southern Africa collaboration. Twelve-year mortality in adults initiating antiretroviral therapy in South Africa. J Int AIDS Soc. 2017; 20(1): 21902.	1 - Covariates
145692	Scientific literature	Viral hepatitis in South African healthcare workers at increased risk of occupational exposure to blood-borne viruses	Vardas E, Ross MH, Sharp G, McAnerney J, Sim J. Viral hepatitis in South African healthcare workers at increased risk of occupational exposure to blood-borne viruses. J Hosp Infect. 2002; 50(1): 6-12.	1 - Covariates
214542	Scientific literature	Virologic, immunologic, and clinical response to highly active antiretroviral therapy: the gender issue revisited	Moore AL, Kirk O, Johnson AM, Katlama C, Blaxhult A, Dietrich M, Colebunders R, Chiesi A, Lungren JD, Phillips AN, EuroSIDA group. Virologic, immunologic, and clinical response to highly active antiretroviral therapy: the gender issue revisited. J Acquir Immune Defic Syndr. 2003; 32(4): 452-61.	1 - Covariates
313644	Survey	Slovenia International Social Survey Programme: National Identity II 2004	ISSP Research Group (2012): International Social Survey Programme: National Identity II - ISSP 2003. GESIS Data Archive, Cologne. ZA3910 Data file Version 2.1.0, doi:10.4232/1.11449.	1 - Covariates
377874	Survey	South Africa - Agincourt Health and Aging in Africa Baseline Survey 2014-2015	Berkman, Lisa. Health and Aging in Africa: A Longitudinal Study of an INDEPTH Community in South Africa [HAALSI] Baseline Survey: Agincourt, South Africa, 2015. Ann Arbor, MI: Inter-university Consortium for Political and Social Research [distributor], 2017-10-30. https://doi.org/10.3886/ICPSR36633.v2	1 - Covariates
135119	Survey	South Africa - Agincourt INDEPTH Study on Global Ageing and Adult Health 2006-2007	MRC/Wits Rural Public Health and Health Transitions Research Unit (Agincourt), World Health Organization (WHO). South Africa - Agincourt INDEPTH Study on Global Ageing and Adult Health 2006-2007. Geneva, Switzerland: World Health Organization (WHO).	1 - Covariates
135825	Survey	South Africa - Agincourt Integrated Family Survey 2002	Case, A. 2003. Agincourt Integrated Family Survey 2002. [dataset] Version 1. Cape Town: DataFirst [distributor].	1 - Covariates
135826	Survey	South Africa - Agincourt Integrated Family Survey 2004	Case, A. 2005. Agincourt Integrated Family Survey 2004. [dataset] Version 1. Cape Town: DataFirst [distributor].	1 - Covariates
143217	Survey	South Africa - Eastern Cape HIV and Syphilis Antenatal Sero-Surveillance Survey 2007	Department of Health (South Africa), Eastern Cape Department of Health (South Africa). South Africa - Eastern Cape HIV and Syphilis Antenatal Sero-Surveillance Survey 2007.	1 - Covariates
11928	Survey	South Africa - KwaZulu and Natal Income Dynamics Study 1993	International Food Policy Research Institute (IFPRI), University of Natal, University of Wisconsin, Southern Africa Labour Development Research Unit (SALDRU), School of Economics, University of Cape Town. South Africa KwaZulu-Natal Income Dynamics Study 1993. Durban, South Africa: University of Natal.	1 - Covariates
261887	Survey	South Africa - Venda Malnutrition and Enteric Disease Study 2009-2014	Fogarty International Center, National Institutes of Health (NIH), Foundation for the National Institutes of Health (FNIH), University of Venda. South Africa - Venda Malnutrition and Enteric Disease Study 2009-2014.	1 - Covariates
342458	Survey	South Africa - Western Cape Survey on Substance Use, Risk Behavior and	Department of Social Development, Western Cape Government (South Africa), South African Medical Research Council, United	1 - Covariates

		Mental Health Among Grade 8-10 Learners in Schools 2011	Nations Office on Drugs and Crime (UNODC). South Africa - Western Cape Survey on Substance Use, Risk Behavior and Mental Health Among Grade 8-10 Learners in Schools 2011.	
340420	Survey	South Africa Afrobarometer Round 1 2000 - ICPSR	Mattes, Robert, Davids, Yul Derek, and Africa, Cherrel. Afrobarometer: Round I Survey of South Africa, July-August 2000. Inter-university Consortium for Political and Social Research [distributor], 2005-12-15. https://doi.org/10.3886/ICPSR03934.v1	1 - Covariates
340618	Survey	South Africa Afrobarometer: The Quality of Democracy and Governance in South Africa Round 3 2006 - ICPSR	Mattes, Robert, Bratton, Michael, and Gyimah-Boadi, E. Afrobarometer Round 3: The Quality of Democracy and Governance in South Africa, 2005. Ann Arbor, MI: Inter-university Consortium for Political and Social Research [distributor], 2008-10-17. https://doi.org/10.3886/ICPSR22211.v1	1 - Covariates
340668	Survey	South Africa Afrobarometer: The Quality of Democracy and Governance in South Africa Round 4 2008 - ICPSR	Ndetlanya, Mcebisi, Ismail, Zenobia, Graham, Paul, Gyimah-Boadi, Emmanuel, Logan, Carolyn, Bratton, Michael, and Mattes, Robert. Afrobarometer Round 4: The Quality of Democracy and Governance in South Africa, 2008. Ann Arbor, MI: Inter-university Consortium for Political and Social Research [distributor], 2012-08-06. https://doi.org/10.3886/ICPSR34011.v1	1 - Covariates
340197	Survey	South Africa Afrobarometer: The Quality of Democracy and Governance in South Africa Round 5 2011 - ICPSR	Kapery, Washeelah, Gyimah-Boadi, E., Bratton, Michael, Mattes, Robert, Logan, Carolyn, and Dulani, Boniface. Afrobarometer Round 5: The Quality of Democracy and Governance in South Africa, 2011. Ann Arbor, MI: Inter-university Consortium for Political and Social Research [distributor], 2015-07-24. https://doi.org/10.3886/ICPSR35563.v1	1 - Covariates
340864	Survey	South Africa Afrobarometer: The Quality of Democracy and Governance in South Africa Round 6 2015	Ghana Center for Democratic Development (CDD-Ghana), Institute for Development Studies, University of Nairobi, Institute for Empirical Research in Political Economy (IERPE), Institute for Justice and Reconciliation (South Africa), Michigan State University, Plus 94 Research (South Africa). South Africa Afrobarometer: The Quality of Democracy and Governance in South Africa Round 6 2015. Afrobarometer, 2017.	1 - Covariates
139810	Survey	South Africa Anthropometric, Vitamin A, Iron and Immunisation Coverage Status in Children Aged 6 to 71 Months 1994	South African Vitamin A Consultative Group (SAVACG). South Africa Anthropometric, Vitamin A, Iron and Immunisation Coverage Status in Children Aged 6 to 71 Months 1994.	1 - Covariates
25100	Survey	South Africa Community Survey 2007	Statistics South Africa. South Africa Community Survey 2007. Pretoria, South Africa: Statistics South Africa.	1 - Covariates
280803	Survey	South Africa Community Survey 2016	Statistics South Africa. South Africa Community Survey 2016. Pretoria, South Africa: Statistics South Africa, 2016.	1 - Covariates
20796	Survey	South Africa Demographic and Health Survey 1998	Department of Health (South Africa), Macro International, Inc, South African Medical Research Council. South Africa Demographic and Health Survey 1998. Fairfax, United States of America: ICF International.	1 - Covariates
20798	Survey	South Africa Demographic and Health Survey 2003-2004	Department of Health (South Africa), Macro International, Inc, South African Medical Research Council. South Africa Demographic and Health Survey 2003-2004.	1 - Covariates
157064	Survey	South Africa Demographic and Health Survey 2016	Department of Health (South Africa), ICF International, South African Medical Research Council, Statistics South Africa. South Africa Demographic and Health Survey 2016. Fairfax, United States of America: ICF International, 2019.	1 - Covariates
115481	Survey	South Africa General Household Survey 2002	Statistics South Africa. South Africa General Household Survey 2002. Pretoria, South Africa: Statistics South Africa.	1 - Covariates
11787	Survey	South Africa General Household Survey 2003	Statistics South Africa. South Africa General Household Survey 2003. Pretoria, South Africa: Statistics South Africa.	1 - Covariates
11788	Survey	South Africa General Household Survey 2004	Statistics South Africa. South Africa General Household Survey 2004. Pretoria, South Africa: Statistics South Africa.	1 - Covariates
11789	Survey	South Africa General Household Survey 2005	Statistics South Africa. South Africa General Household Survey 2005. Pretoria, South Africa: Statistics South Africa.	1 - Covariates
115486	Survey	South Africa General Household Survey 2006	Statistics South Africa. South Africa General Household Survey 2006. Pretoria, South Africa: Statistics South Africa.	1 - Covariates
11790	Survey	South Africa General Household Survey 2007	Statistics South Africa. South Africa General Household Survey 2007. Cape Town, South Africa: DataFirst.	1 - Covariates
115488	Survey	South Africa General Household Survey 2008	Statistics South Africa. South Africa General Household Survey 2008. Pretoria, South Africa: Statistics South Africa.	1 - Covariates
115489	Survey	South Africa General Household Survey 2009	Statistics South Africa. South Africa General Household Survey 2009. Pretoria, South Africa: Statistics South Africa.	1 - Covariates
115490	Survey	South Africa General Household Survey 2010	Statistics South Africa. South Africa General Household Survey 2010. Pretoria, South Africa: Statistics South Africa.	1 - Covariates

115491	Survey	South Africa General Household Survey 2011	Statistics South Africa. South Africa General Household Survey 2011. Cape Town, South Africa: DataFirst.	1 - Covariates
135534	Survey	South Africa General Household Survey 2012	Statistics South Africa. South Africa General Household Survey 2012. Pretoria, South Africa: Statistics South Africa, 2013.	1 - Covariates
238485	Survey	South Africa General Household Survey 2014	Statistics South Africa. South Africa General Household Survey 2014. Cape Town, South Africa: DataFirst, 2015.	1 - Covariates
265084	Survey	South Africa General Household Survey 2015	Statistics South Africa. South Africa General Household Survey 2015. Cape Town, South Africa: DataFirst, 2015.	1 - Covariates
317089	Survey	South Africa General Household Survey 2016	Statistics South Africa. South Africa General Household Survey 2016. Cape Town, South Africa: DataFirst, 2015.	1 - Covariates
11795	Survey	South Africa Global Youth Tobacco Survey 1999	Centers for Disease Control and Prevention (CDC) and World Health Organization (WHO). South Africa Global Youth Tobacco Survey 1999. Atlanta, United States: Centers for Disease Control and Prevention (CDC).	1 - Covariates
11800	Survey	South Africa Global Youth Tobacco Survey 2002	Centers for Disease Control and Prevention (CDC) and World Health Organization (WHO). South Africa Global Youth Tobacco Survey 2002. Atlanta, United States: Centers for Disease Control and Prevention (CDC).	1 - Covariates
110319	Survey	South Africa Global Youth Tobacco Survey 2008	Centers for Disease Control and Prevention (CDC), World Health Organization (WHO). South Africa Global Youth Tobacco Survey 2008. Atlanta, United States: Centers for Disease Control and Prevention (CDC).	1 - Covariates
110320	Survey	South Africa Global Youth Tobacco Survey 2011	Centers for Disease Control and Prevention (CDC), World Health Organization (WHO). South Africa Global Youth Tobacco Survey 2011. Atlanta, United States of America: Centers for Disease Control and Prevention (CDC), 2013.	1 - Covariates
11826	Survey	South Africa Income and Expenditure Survey 2000	Statistics South Africa. South Africa Income and Expenditure Survey 2000. Pretoria, South Africa: Statistics South Africa.	1 - Covariates
11848	Survey	South Africa Income and Expenditure Survey 2005-2006	Statistics South Africa. South Africa Income and Expenditure Survey 2005-2006. Pretoria, South Africa: Statistics South Africa.	1 - Covariates
106158	Survey	South Africa Integrated Family Survey 1999	Southern Africa Labour Development Research Unit (SALDRU), University of Cape Town. South Africa Integrated Family Survey 1999.	1 - Covariates
313734	Survey	South Africa International Social Survey Programme: Citizenship 2004	International Social Survey Programme (ISSP). South Africa International Social Survey Programme: Citizenship 2004. Mannheim, Germany: GESIS - Leibniz Institute for the Social Sciences, 2012.	1 - Covariates
322406	Survey	South Africa International Social Survey Programme: Citizenship II 2015	ISSP Research Group (2016): International Social Survey Programme: Citizenship II - ISSP 2014. GESIS Data Archive, Cologne. ZA6670 Data file Version 2.0.0, doi:10.4232/1.12590	1 - Covariates
313400	Survey	South Africa International Social Survey Programme: Environment III 2010	ISSP Research Group (2012): International Social Survey Programme: Environment III - ISSP 2010. GESIS Data Archive, Cologne. ZA5500 Data file Version 2.0.0, doi:10.4232/1.11418.	1 - Covariates
313462	Survey	South Africa International Social Survey Programme: Family and Changing Gender Roles IV 2012-2013	ISSP Research Group (2016): International Social Survey Programme: Family and Changing Gender Roles IV - ISSP 2012. GESIS Data Archive, Cologne. ZA5900 Data file Version 4.0.0, doi:10.4232/1.12661.	1 - Covariates
142804	Survey	South Africa International Social Survey Programme: Health and Health Care 2011	ISSP Research Group (2009): International Social Survey Programme: Health and Health Care - ISSP 2011. GESIS Data Archive, Cologne. ZA5800 Data file version 3.0.0, doi:10.4232/1.12252.	1 - Covariates
142830	Survey	South Africa International Social Survey Programme: Leisure Time and Sports 2007	ISSP Research Group (2009): International Social Survey Programme: Leisure Time and Sports - ISSP 2007. GESIS Data Archive, Cologne. ZA4850 Data file version 2.0.0, doi:10.4231/1.10079.	1 - Covariates
322368	Survey	South Africa International Social Survey Programme: National Identity III 2013-2014	ISSP Research Group (2015): International Social Survey Programme: National Identity III - ISSP 2013. GESIS Data Archive, Cologne. ZA5950 Data file Version 2.0.0, doi:10.4232/1.12312	1 - Covariates
313314	Survey	South Africa International Social Survey Programme: Religion III 2008	International Social Survey Programme (ISSP). South Africa International Social Survey Programme: Religion III 2008. Mannheim, Germany: GESIS - Leibniz Institute for the Social Sciences, 2012.	1 - Covariates
313246	Survey	South Africa International Social Survey Programme: Role of Government IV 2006	ISSP Research Group (2008): International Social Survey Programme: Role of Government IV - ISSP 2006. GESIS Data Archive, Cologne. ZA4700 Data file Version 1.0.0, doi:10.4232/1.4700.	1 - Covariates
313350	Survey	South Africa International Social	ISSP Research Group (2017): International Social Survey	1 - Covariates

		Survey Programme: Social Inequality IV 2009	Programme: Social Inequality IV - ISSP 2009. GESIS Data Archive, Cologne. ZA5400 Data file Version 4.0.0, doi:10.4232/1.12777.	
313765	Survey	South Africa International Social Survey Programme: Work Orientation III 2005	International Social Survey Programme (ISSP). South Africa International Social Survey Programme: Work Orientation III 2005. Mannheim, Germany: GESIS - Leibniz Institute for the Social Sciences, 2013.	1 - Covariates
322486	Survey	South Africa International Social Survey Programme: Work Orientations IV 2015-2016	The International Social Survey Programme (ISSP) represents a series of cross-national collaborations between organizations conducting similar social science research. ISSP researchers focus on developing survey questions that are relevant and comprehensible in all countries and languages, with survey results that are comparable across nations.Topics covered on this round of the ISSP include: working conditions; labor relations and conflict; and employment and unemployment.	1 - Covariates
12013	Survey	South Africa KwaZulu-Natal Income Dynamics Study 1998	International Food Policy Research Institute (IFPRI), University of Natal, University of Wisconsin, Data Research Africa (DRA), Policy and Praxis, Southern Africa Labour Development Research Unit (SALDRU), School of Economics, University of Cape Town. South Africa KwaZulu-Natal Income Dynamics Study 1998. Durban, South Africa: University of Natal.	1 - Covariates
31142	Survey	South Africa KwaZulu-Natal Income Dynamics Study 2004	University of Kwazulu-Natal, University of Wisconsin, London School of Hygiene and Tropical Medicine, International Food Policy Research Institute (IFPRI), Department of Social Development (South Africa), Norwegian Institute for Urban and Regional Research (NIBR). South Africa KwaZulu-Natal Income Dynamics Study 2004. Durban, South Africa: University of Kwazulu-Natal.	1 - Covariates
12017	Survey	South Africa Labor Force Survey 2004	Statistics South Africa. Labour Force Survey: March 2004. [dataset]. Version 2. Pretoria: Statistics South Africa [producer], 2004. Cape Town: DataFirst [distributor], 2011.	1 - Covariates
280043	Survey	South Africa Labor Market Dynamics 2008	Statistics South Africa. Labour Market Dynamics in South Africa 2008 [dataset]. Version 1.1. Pretoria: Statistics South Africa [producer], 2015. Cape Town: DataFirst [distributor], 2015.	1 - Covariates
280050	Survey	South Africa Labor Market Dynamics 2009	Statistics South Africa. Labour Market Dynamics in South Africa 2009 [dataset]. Version 1.1. Pretoria: Statistics South Africa [producer], 2015. Cape Town: DataFirst [distributor], 2015.	1 - Covariates
280051	Survey	South Africa Labor Market Dynamics 2010	Statistics South Africa. Labour Market Dynamics in South Africa 2010 [dataset]. Version 1.1. Pretoria: Statistics South Africa [producer], 2015. Cape Town: DataFirst [distributor], 2015.	1 - Covariates
280063	Survey	South Africa Labor Market Dynamics 2011	Statistics South Africa. Labour Market Dynamics in South Africa 2011 [dataset]. Version 1.2. Pretoria: Statistics South Africa [producer], 2015. Cape Town: DataFirst [distributor], 2015.	1 - Covariates
280069	Survey	South Africa Labor Market Dynamics 2012	Statistics South Africa. Labour Market Dynamics in South Africa 2012 [dataset]. Version 1.2. Pretoria: Statistics South Africa [producer], 2015. Cape Town: DataFirst [distributor], 2015.	1 - Covariates
280075	Survey	South Africa Labor Market Dynamics 2013	Statistics South Africa. Labour Market Dynamics in South Africa 2013 [dataset]. Version 1. Pretoria: Statistics South Africa [producer], 2014. Cape Town: DataFirst [distributor], 2015.	1 - Covariates
12101	Survey	South Africa Living Standards Measurement Study 1993	Southern Africa Labour Development Research Unit (SALDRU), University of Cape Town, World Bank. South Africa Living Standards Measurement Study 1993. Washington DC, United States of America: World Bank.	1 - Covariates
143233	Survey	South Africa National Antenatal Sentinel HIV and Syphilis Prevalence Survey 2000	Department of Health (South Africa). South Africa National Antenatal Sentinel HIV and Syphilis Prevalence Survey 2000.	1 - Covariates
356401	Survey	South Africa National Antenatal Sentinel HIV and Syphilis Prevalence Survey 2008	Department of Health (South Africa). South Africa National Antenatal Sentinel HIV and Syphilis Prevalence Survey 2008.	1 - Covariates
261367	Survey	South Africa National Food Consumption Survey - Fortification Baseline 2005	Department of Health (South Africa), North-West University, South African Medical Research Council, Stellenbosch University, University of Cape Town, University of Kwazulu-Natal, University of Limpopo, University of Pretoria, University of the Free State, University of the Western Cape. South Africa National Food Consumption Survey - Fortification Baseline 2005.	1 - Covariates
155628	Survey	South Africa National Health and Nutrition Examination Survey 2012	Human Sciences Research Council, South African Medical Research Council. South Africa National Health and Nutrition Examination Survey 2012.	1 - Covariates
150187	Survey	South Africa National HIV and Syphillis Antenatal Sero-Prevalence	Department of Health (South Africa). South Africa National HIV and Syphillis Antenatal Sero-Prevalence Survey 2002.	1 - Covariates

		Survey 2002		
313076	Survey	South Africa National HIV Prevalence, Incidence, and Behavior Survey 2011-2012	Centers for Disease Control and Prevention (CDC), Global Clinical and Viral Laboratory (South Africa), Human Sciences Research Council, National Institute for Communicable Diseases (South Africa), South African Medical Research Council, University of Cape Town. South Africa National HIV Prevalence, Incidence, and Behavior Survey 2011-2012. Pretoria, South Africa: Human Sciences Research Council, 2016.	1 - Covariates
313074	Survey	South Africa National HIV Prevalence, Incidence, Behavior and Communication Survey 2004-2005	Center for AIDS Development, Research and Evaluation (CADRE) (South Africa), Centers for Disease Control and Prevention (CDC), Global Clinical and Viral Laboratory (South Africa), Human Sciences Research Council, Maphume Research Services, National Institute for Communicable Diseases (South Africa). South Africa National HIV Prevalence, Incidence, Behavior and Communication Survey 2004-2005. Pretoria, South Africa: Human Sciences Research Council, 2011.	1 - Covariates
357200	Survey	South Africa National HIV Prevalence, Incidence, Behavior and Communication Survey 2017	Centers for Disease Control and Prevention (CDC), Human Sciences Research Council, National Institute for Communicable Diseases (South Africa), South African Medical Research Council. South Africa National HIV Prevalence, Incidence, Behavior and Communication Survey 2017.	1 - Covariates
27885	Survey	South Africa National Income Dynamics Study - Wave 1 2008	University of Cape Town, Southern Africa Labour and Development Research Unit. National Income Dynamics Study (NIDS) Wave 1 [computer files]. Cape Town: Southern Africa Labour and Development Research Unit [producer], 2009. Cape Town: DataFirst [distributor], 2009	1 - Covariates
133731	Survey	South Africa National Income Dynamics Study - Wave 2 2010-2011	Southern Africa Labour and Development Research Unit. National Income Dynamics Study 2010-2011, Wave 2. Version 1.0. Cape Town: Southern Africa Labour and Development Research Unit [producer], 2012. Cape Town: DataFirst [distributor], 2013.	1 - Covariates
133732	Survey	South Africa National Income Dynamics Study - Wave 3 2012	Southern Africa Labour and Development Research Unit. National Income Dynamics Study 2012, Wave 3 [dataset]. Version 1.2. Cape Town: Southern Africa Labour and Development Research Unit [producer], 2013. Cape Town: DataFirst [distributor], 2013	1 - Covariates
265153	Survey	South Africa National Income Dynamics Study - Wave 4 2014-2015	Southern Africa Labour and Development Research Unit. National Income Dynamics Study 2014 - 2015, Wave 4 [dataset]. Version 1.1. Cape Town: Southern Africa Labour and Development Research Unit [producer], 2016. Cape Town: DataFirst [distributor], 2016. Pretoria: Department of Planning Monitoring and Evaluation [commissioner], 2014	1 - Covariates
257511	Survey	South Africa National Youth Risk Behaviour Survey 2002	Centers for Disease Control and Prevention (CDC), Department of Health (South Africa), National Department of Education (South Africa), South African Medical Research Council. South Africa National Youth Risk Behaviour Survey 2002.	1 - Covariates
257515	Survey	South Africa National Youth Risk Behaviour Survey 2008	Department of Health (South Africa), National Department of Education (South Africa), South African Medical Research Council. South Africa National Youth Risk Behaviour Survey 2008.	1 - Covariates
105306	Survey	South Africa October Household Survey 1994	Central Statistical Service (South Africa). South Africa October Household Survey 1994.	1 - Covariates
106684	Survey	South Africa October Household Survey 1995	Central Statistical Service (South Africa). South Africa October Household Survey 1995.	1 - Covariates
12104	Survey	South Africa October Household Survey 1996	Central Statistical Service (South Africa). South Africa October Household Survey 1996.	1 - Covariates
106686	Survey	South Africa October Household Survey 1997	Central Statistical Service (South Africa). South Africa October Household Survey 1997.	1 - Covariates
12105	Survey	South Africa October Household Survey 1998	Statistics South Africa. South Africa October Household Survey 1998.	1 - Covariates
12106	Survey	South Africa October Household Survey 1999	Statistics South Africa. South Africa October Household Survey 1999. Cape Town, South Africa: DataFirst.	1 - Covariates
286339	Survey	South Africa Programmatic Mapping and Size Estimation of Key Populations 2015	Human Sciences Research Council, Networking HIV, AIDS Community of South Africa (NACOSA). South Africa Programmatic Mapping and Size Estimation of Key Populations 2015.	1 - Covariates
280812	Survey	South Africa Quarterly Labor Force Survey 2008, Quarter 1	Statistics South Africa. Quarterly Labour Force Survey 2008: Q1 [dataset]. Version 2.0. Pretoria: Statistics South Africa [producer], 2008. Cape Town: DataFirst [distributor], 2012.	1 - Covariates
280819	Survey	South Africa Quarterly Labor Force Survey 2009, Quarter 1	Statistics South Africa. Quarterly Labour Force Survey 2009: Q1 [dataset]. Version 2.0. Pretoria: Statistics South Africa [producer], 2009. Cape Town: DataFirst [distributor], 2012.	1 - Covariates

280826	Survey	South Africa Quarterly Labor Force Survey 2010, Quarter 1	Statistics South Africa. Quarterly Labour Force Survey 2010: Q1 [dataset]. Version 3.0. Pretoria: Statistics South Africa [producer], 2010. Cape Town: DataFirst [distributor], 2012.	1 - Covariates
280854	Survey	South Africa Quarterly Labor Force Survey 2011, Quarter 1	Statistics South Africa. Quarterly Labour Force Survey 2011: Q1 [dataset]. Version 1.1. Pretoria: Statistics South Africa [producer], 2011. Cape Town: DataFirst [distributor], 2012.	1 - Covariates
280861	Survey	South Africa Quarterly Labor Force Survey 2012, Quarter 1	Statistics South Africa. Quarterly Labour Force Survey 2012: Q1 [dataset]. Version 1.1. Pretoria: Statistics South Africa [producer], 2012. Cape Town: DataFirst [distributor], 2012.	1 - Covariates
280869	Survey	South Africa Quarterly Labor Force Survey 2013, Quarter 1	Statistics South Africa. Quarterly Labour Force Survey 2013: Q1 [dataset]. Version 1.0. Pretoria: Statistics South Africa [producer], 2013. Cape Town: DataFirst [distributor], 2013.	1 - Covariates
280875	Survey	South Africa Quarterly Labor Force Survey 2014, Quarter 1	Statistics South Africa. Quarterly Labour Force Survey 2014: Q1 [dataset]. Version 1.0. Pretoria: Statistics South Africa [producer], 2014. Cape Town: DataFirst [distributor], 2014.	1 - Covariates
280881	Survey	South Africa Quarterly Labor Force Survey 2015, Quarter 1	Statistics South Africa. Quarterly Labour Force Survey 2015: Q1 [dataset]. Version 1.0. Pretoria: Statistics South Africa [producer], 2015. Cape Town: DataFirst [distributor], 2015.	1 - Covariates
280891	Survey	South Africa Quarterly Labor Force Survey 2016, Quarter 1	Statistics South Africa. Quarterly Labour Force Survey 2016: Q1 [dataset]. Version 1.0. Pretoria: Statistics South Africa [producer], 2016. Cape Town: DataFirst [distributor], 2016.	1 - Covariates
419800	Survey	South Africa Rapid Assessment of Avoidable Blindness Survey 2010	International Centre for Eye Health (ICEH). South Africa Rapid Assessment of Avoidable Blindness Survey 2010. Grootebroek, Netherlands: RAAB Repository, 2014.	1 - Covariates
111488	Survey	South Africa WHO Study on Global AGEing and Adult Health 2007-2008	Department of Health (South Africa), Human Sciences Research Council, World Health Organization (WHO). South Africa WHO Study on Global AGEing and Adult Health 2007-2008. Geneva, Switzerland: World Health Organization (WHO).	1 - Covariates
21864	Survey	South Africa World Health Survey 2002-2003	World Health Organization (WHO). South Africa World Health Survey 2002-2003. Geneva, Switzerland: World Health Organization (WHO), 2005.	1 - Covariates
344630	Survey	South Africa World Poll 2005-2006	Gallup. South Africa World Poll 2005-2006.	1 - Covariates
344740	Survey	South Africa World Poll 2007	Gallup. South Africa World Poll 2007.	1 - Covariates
344867	Survey	South Africa World Poll 2008	Gallup. South Africa World Poll 2008.	1 - Covariates
344987	Survey	South Africa World Poll 2009	Gallup. South Africa World Poll 2009.	1 - Covariates
345165	Survey	South Africa World Poll 2010	Gallup. South Africa World Poll 2010.	1 - Covariates
345541	Survey	South Africa World Poll 2011	Gallup. South Africa World Poll 2011.	1 - Covariates
345934	Survey	South Africa World Poll 2012	Gallup. South Africa World Poll 2012.	1 - Covariates
346334	Survey	South Africa World Poll 2013	Gallup. South Africa World Poll 2013.	1 - Covariates
346494	Survey	South Africa World Poll 2014	Gallup. South Africa World Poll 2014.	1 - Covariates
341342	Survey	South Africa World Poll 2015	Gallup. South Africa World Poll 2015.	1 - Covariates
341648	Survey	South Africa World Poll 2016	Gallup. South Africa World Poll 2016.	1 - Covariates
341860	Survey	South Africa World Poll 2017	Gallup. South Africa World Poll 2017.	1 - Covariates
388162	Survey	South Africa World Poll 2018	Gallup. South Africa World Poll 2018.	1 - Covariates
438053	Survey	UNICEF Maternal and Newborn Health Coverage Database as of November 2019	United Nations Children's Fund (UNICEF). UNICEF Maternal and Newborn Health Coverage Database as of November 2019. New York, United States of America: United Nations Children's Fund (UNICEF), 2019.	1 - Covariates
126610	Survey	United Kingdom - England Adult Psychiatric Morbidity Survey 2006-2007 - UK Data Service	National Centre for Social Research and University of Leicester, Adult Psychiatric Morbidity Survey, 2007 [computer file]. 3rd Edition. Colchester, Essex: UK Data Archive [distributor], January 2011. SN: 6379, http://dx.doi.org/10.5255/UKDA-SN-6379-1	1 - Covariates
22341	Survey	United Kingdom Health Survey for England 1997-1998 - UK Data Service	Joint Health Surveys Unit of Social and Community Planning Research and University College London, Health Survey for England, 1997 [computer file]. 2nd ed. Colchester, Essex: UK Data Archive [distributor], 4 December 2000. SN: 3979.	1 - Covariates
22352	Survey	United Kingdom Health Survey for England 1998-1999 - UK Data Service	National Centre for Social Research, University College London Department of Epidemiology and Public Health, Health Survey for England, 1998 [computer file]. 4th ed. Colchester, Essex: UK Data Archive [distributor], 30 November 2002. SN: 4150.	1 - Covariates

22364	Survey	United Kingdom Health Survey for England 1999-2000 - UK Data Service	National Centre for Social Research and University College London. Department of Epidemiology and Public Health, Health Survey for England, 1999 [computer file]. 3rd Edition. Colchester, Essex: UK Data Archive [distributor], February 2002. SN: 4365.	1 - Covariates
22374	Survey	United Kingdom Health Survey for England 2000-2001 - UK Data Service	National Centre for Social Research, University College London Department of Epidemiology and Public Health, Health Survey for England, 2000 [computer file]. Colchester, Essex: UK Data Archive [distributor], 23 April 2002. SN: 4487.	1 - Covariates
22388	Survey	United Kingdom Health Survey for England 2001-2002 - UK Data Service	National Centre for Social Research and University College London. Department of Epidemiology and Public Health, Health Survey for England, 2001 [computer file]. 2nd Edition. Colchester, Essex: UK Data Archive [distributor], June 2004. SN: 4628.	1 - Covariates
22433	Survey	United Kingdom Health Survey for England 2003-2004 - UK Data Service	National Centre for Social Research and University College London. Department of Epidemiology and Public Health, Health Survey for England, 2003 [computer file]. Colchester, Essex: UK Data Archive [distributor], March 2005. SN: 5098.	1 - Covariates
22449	Survey	United Kingdom Health Survey for England 2004-2005 - UK Data Service	National Centre for Social Research and University College London. Department of Epidemiology and Public Health, Health Survey for England, 2004 [computer file]. Colchester, Essex: UK Data Archive [distributor], July 2006. SN: 5439.	1 - Covariates
22463	Survey	United Kingdom Health Survey for England 2005-2006 - UK Data Service	National Centre for Social Research and University College London. Department of Epidemiology and Public Health, Health Survey for England, 2005 [computer file]. Colchester, Essex: UK Data Archive [distributor], July 2007. SN: 5675.	1 - Covariates
22476	Survey	United Kingdom Health Survey for England 2006-2007 - UK Data Service	National Centre for Social Research and University College London. Department of Epidemiology and Public Health, Health Survey for England, 2006 [computer file]. 4th Edition. Colchester, Essex: UK Data Archive [distributor], July 2011. SN: 5809, http://dx.doi.org/10.5255/UKDA-SN-5809-1	1 - Covariates
95628	Survey	United Kingdom Health Survey for England 2007-2008 - UK Data Service	National Centre for Social Research and University College London. Department of Epidemiology and Public Health, Health Survey for England, 2007 [computer file]. 2nd Edition. Colchester, Essex: UK Data Archive [distributor], April 2010. SN: 6112, http://dx.doi.org/10.5255/UKDA-SN-6112-1	1 - Covariates
95629	Survey	United Kingdom Health Survey for England 2008-2009 - UK Data Service	National Centre for Social Research and University College London. Department of Epidemiology and Public Health, Health Survey for England, 2008 [computer file]. 3rd Edition. Colchester, Essex: UK Data Archive [distributor], July 2011. SN: 6397, http://dx.doi.org/10.5255/UKDA-SN-6397-1	1 - Covariates
95630	Survey	United Kingdom Health Survey for England 2009-2010 - UK Data Service	National Centre for Social Research and University College London. Department of Epidemiology and Public Health, Health Survey for England, 2009 [computer file]. 2nd Edition. Colchester, Essex: UK Data Archive [distributor], July 2011. SN: 6732, http://dx.doi.org/10.5255/UKDA-SN-6732-1	1 - Covariates
130050	Survey	United Kingdom National Survey of Sexual Attitudes and Lifestyles 1999-2001 - UK Data Service	National Centre for Social Research et al. , National Survey of Sexual Attitudes and Lifestyles II, 2000-2001 [computer file]. Colchester, Essex: UK Data Archive [distributor], August 2005. SN: 5223, http://dx.doi.org/10.5255/UKDA-SN-5223-1	1 - Covariates
43713	Survey	WHO Global Database on Vitamin A Deficiency	World Health Organization (WHO). WHO Global Database on Vitamin A Deficiency. Geneva, Switzerland: World Health Organization (WHO).	1 - Covariates
162652	Survey	Zambia Access, Bottlenecks, Costs, and Equity Project 2011-2012	Central Statistical Office (Zambia), Churches Health Association of Zambia (CHAZ), Clinton Health Access Initiative (CHAI), Institute for Health Metrics and Evaluation (IHME), Ministry of Health (Zambia), University of Zambia. Access, Bottlenecks, Costs, and Equity (ABCE) project in Zambia, 2011-2012. Seattle, United States: Institute for Health Metrics and Evaluation (IHME), 2015.	1 - Covariates
106599	Vital registration	South Africa Saving Mothers 2008-2010	National Committee on Confidential Enquiries into Maternal Deaths (South Africa). South Africa Saving Mothers 2008-2010. Pretoria, South Africa: Department of Health (South Africa), 2012.	1 - Covariates
215184	Vital registration	South Africa Saving Mothers 2011-2013	National Committee on Confidential Enquiries into Maternal Deaths (South Africa). South Africa Saving Mothers 2011-2013. Pretoria, South Africa: Department of Health (South Africa), 2014.	1 - Covariates
107077	Vital registration	South Africa Vital Registration - Causes of Death 1997-2005	Department of Home Affairs (South Africa), Statistics South Africa. South Africa Vital Registration - Causes of Death 1997-2005. Pretoria, South Africa: Statistics South Africa.	1 - Covariates
151816	Vital registration	South Africa Vital Registration - Causes of Death 2006	Department of Home Affairs (South Africa), Statistics South Africa. South Africa Vital Registration - Causes of Death 2006. Pretoria, South Africa: Statistics South Africa.	1 - Covariates

151817	Vital registration	South Africa Vital Registration - Causes of Death 2007	Department of Home Affairs (South Africa), Statistics South Africa. South Africa Vital Registration - Causes of Death 2007. Pretoria, South Africa: Statistics South Africa.	1 - Covariates
106583	Vital registration	South Africa Vital Registration - Causes of Death 2008	Department of Home Affairs (South Africa), Statistics South Africa. South Africa Vital Registration - Causes of Death 2008. Pretoria, South Africa: Statistics South Africa.	1 - Covariates
106584	Vital registration	South Africa Vital Registration - Causes of Death 2009	Department of Home Affairs (South Africa), Statistics South Africa. South Africa Vital Registration - Causes of Death 2009. Pretoria, South Africa: Statistics South Africa.	1 - Covariates
151818	Vital registration	South Africa Vital Registration - Causes of Death 2010	Department of Home Affairs (South Africa), Statistics South Africa. South Africa Vital Registration - Causes of Death 2010. Pretoria, South Africa: Statistics South Africa.	1 - Covariates
151819	Vital registration	South Africa Vital Registration - Causes of Death 2011	Department of Home Affairs (South Africa), Statistics South Africa. South Africa Vital Registration - Causes of Death 2011. Pretoria, South Africa: Statistics South Africa.	1 - Covariates
204016	Vital registration	South Africa Vital Registration - Causes of Death 2012	Department of Home Affairs (South Africa), Statistics South Africa. South Africa Vital Registration - Causes of Death 2012. Pretoria, South Africa: Statistics South Africa.	1 - Covariates
204017	Vital registration	South Africa Vital Registration - Causes of Death 2013	Department of Home Affairs (South Africa), Statistics South Africa. South Africa Vital Registration - Causes of Death 2013. Pretoria, South Africa: Statistics South Africa.	1 - Covariates
267740	Vital registration	South Africa Vital Registration - Causes of Death 2014	Department of Home Affairs (South Africa), Statistics South Africa. South Africa Vital Registration - Causes of Death 2014. Pretoria, South Africa: Statistics South Africa.	1 - Covariates
312271	Vital registration	South Africa Vital Registration - Causes of Death 2015	Department of Home Affairs (South Africa), Statistics South Africa. South Africa Vital Registration - Causes of Death 2015. Pretoria, South Africa: Statistics South Africa.	1 - Covariates
399147	Vital registration	South Africa Vital Registration - Causes of Death 2016	Department of Home Affairs (South Africa), Statistics South Africa. South Africa Vital Registration - Causes of Death 2016. Pretoria, South Africa: Statistics South Africa, 2019.	1 - Covariates
12142	Census	South Africa Census 1996	Statistics South Africa. South Africa Census 1996. Pretoria, South Africa: Statistics South Africa.	21 - Mortality
43146	Census	South Africa Census 1996 - IPUMS	Central Statistical Service (South Africa), Minnesota Population Center. South Africa Census 1996 from the Integrated Public Use Microdata Series, International: [Machine-readable database]. Minneapolis: University of Minnesota.	21 - Mortality
43152	Census	South Africa Census 2001 - IPUMS	Statistics South Africa, Minnesota Population Center. South Africa Census 2001 from the Integrated Public Use Microdata Series, International: [Machine-readable database]. Minneapolis: University of Minnesota.	21 - Mortality
43158	Census	South Africa Community Survey 2007 - IPUMS	Statistics South Africa, Minnesota Population Center. South Africa Community Survey 2007 from the Integrated Public Use Microdata Series, International: [Machine-readable database]. Minneapolis: University of Minnesota.	21 - Mortality
12146	Census	South Africa Population and Housing Census 2011	Statistics South Africa. South Africa Population and Housing Census 2011.	21 - Mortality
335911	Demographic surveillance	INDEPTHStats Desktop Version	INDEPTH. INDEPTHStats Desktop Version. Accra, Ghana: INDEPTH.	21 - Mortality
140966	Estimate	United Nations Demographic Yearbook	United Nations Statistics Division (UNSD). United Nations Demographic Yearbook. New York City, United States: United Nations Statistics Division (UNSD).	21 - Mortality
135570	Report	South Africa Rapid Mortality Surveillance Report 2012	South African Medical Research Council. South Africa Rapid Mortality Surveillance Report 2012. Cape Town, South Africa: South African Medical Research Council, 2014.	21 - Mortality
11928	Survey	South Africa - KwaZulu and Natal Income Dynamics Study 1993	International Food Policy Research Institute (IFPRI), University of Natal, University of Wisconsin, Southern Africa Labour Development Research Unit (SALDRU), School of Economics, University of Cape Town. South Africa KwaZulu-Natal Income Dynamics Study 1993. Durban, South Africa: University of Natal.	21 - Mortality
25100	Survey	South Africa Community Survey 2007	Statistics South Africa. South Africa Community Survey 2007. Pretoria, South Africa: Statistics South Africa.	21 - Mortality
280803	Survey	South Africa Community Survey 2016	Statistics South Africa. South Africa Community Survey 2016. Pretoria, South Africa: Statistics South Africa, 2016.	21 - Mortality
164898	Survey	South Africa Demographic and Health Survey 1987-1989	Human Sciences Research Council. South African Demographic and Health Survey, 1987 [Computer file]. S0115. Pretoria: Human Sciences Research Council [producer], 1987. Pretoria: South African Data Archive, National Research Foundation [distributor], 1999.	21 - Mortality
20796	Survey	South Africa Demographic and Health	Department of Health (South Africa), Macro International, Inc, South	21 - Mortality

		Survey 1998	African Medical Research Council. South Africa Demographic and Health Survey 1998. Fairfax, United States of America: ICF International.	
20798	Survey	South Africa Demographic and Health Survey 2003-2004	Department of Health (South Africa), Macro International, Inc, South African Medical Research Council. South Africa Demographic and Health Survey 2003-2004.	21 - Mortality
157064	Survey	South Africa Demographic and Health Survey 2016	Department of Health (South Africa), ICF International, South African Medical Research Council, Statistics South Africa. South Africa Demographic and Health Survey 2016. Fairfax, United States of America: ICF International, 2019.	21 - Mortality
12101	Survey	South Africa Living Standards Measurement Study 1993	Southern Africa Labour Development Research Unit (SALDRU), University of Cape Town, World Bank. South Africa Living Standards Measurement Study 1993. Washington DC, United States of America: World Bank.	21 - Mortality
133731	Survey	South Africa National Income Dynamics Study - Wave 2 2010-2011	Southern Africa Labour and Development Research Unit. National Income Dynamics Study 2010-2011, Wave 2. Version 1.0. Cape Town: Southern Africa Labour and Development Research Unit [producer], 2012. Cape Town: DataFirst [distributor], 2013.	21 - Mortality
265153	Survey	South Africa National Income Dynamics Study - Wave 4 2014-2015	Southern Africa Labour and Development Research Unit. National Income Dynamics Study 2014 - 2015, Wave 4 [dataset]. Version 1.1. Cape Town: Southern Africa Labour and Development Research Unit [producer], 2016. Cape Town: DataFirst [distributor], 2016. Pretoria: Department of Planning Monitoring and Evaluation [commissioner], 2014	21 - Mortality
369644	Survey	South Africa National Income Dynamics Study - Wave 5 2017	Southern Africa Labour and Development Research Unit. National Income Dynamics Study 2017, Wave 5 [dataset]. Version 1.0.0 Pretoria: Department of Planning, Monitoring, and Evaluation [funding agency]. Cape Town: Southern Africa Labour and Development Research Unit [implementer], 2018. Cape Town: DataFirst [distributor], 2018.	21 - Mortality
12103	Survey	South Africa October Household Survey 1993	Central Statistical Service (South Africa). South Africa October Household Survey 1993.	21 - Mortality
106684	Survey	South Africa October Household Survey 1995	Central Statistical Service (South Africa). South Africa October Household Survey 1995.	21 - Mortality
12104	Survey	South Africa October Household Survey 1996	Central Statistical Service (South Africa). South Africa October Household Survey 1996.	21 - Mortality
106686	Survey	South Africa October Household Survey 1997	Central Statistical Service (South Africa). South Africa October Household Survey 1997.	21 - Mortality
12105	Survey	South Africa October Household Survey 1998	Statistics South Africa. South Africa October Household Survey 1998.	21 - Mortality
121967	Vital registration	Country Mortality Data 1980-1999	World Health Organization (WHO). Country Mortality Data 1980-1999.	21 - Mortality
106649	Vital registration	South Africa Mortality and Causes of Death 2010	Department of Health (South Africa), Department of Home Affairs (South Africa), Statistics South Africa. South Africa Mortality and Causes of Death 2010. Pretoria, South Africa: Statistics South Africa.	21 - Mortality
107077	Vital registration	South Africa Vital Registration - Causes of Death 1997-2005	Department of Home Affairs (South Africa), Statistics South Africa. South Africa Vital Registration - Causes of Death 1997-2005. Pretoria, South Africa: Statistics South Africa.	21 - Mortality
151816	Vital registration	South Africa Vital Registration - Causes of Death 2006	Department of Home Affairs (South Africa), Statistics South Africa. South Africa Vital Registration - Causes of Death 2006. Pretoria, South Africa: Statistics South Africa.	21 - Mortality
151817	Vital registration	South Africa Vital Registration - Causes of Death 2007	Department of Home Affairs (South Africa), Statistics South Africa. South Africa Vital Registration - Causes of Death 2007. Pretoria, South Africa: Statistics South Africa.	21 - Mortality
106583	Vital registration	South Africa Vital Registration - Causes of Death 2008	Department of Home Affairs (South Africa), Statistics South Africa. South Africa Vital Registration - Causes of Death 2008. Pretoria, South Africa: Statistics South Africa.	21 - Mortality
106584	Vital registration	South Africa Vital Registration - Causes of Death 2009	Department of Home Affairs (South Africa), Statistics South Africa. South Africa Vital Registration - Causes of Death 2009. Pretoria, South Africa: Statistics South Africa.	21 - Mortality
151818	Vital registration	South Africa Vital Registration - Causes of Death 2010	Department of Home Affairs (South Africa), Statistics South Africa. South Africa Vital Registration - Causes of Death 2010. Pretoria, South Africa: Statistics South Africa.	21 - Mortality
151819	Vital registration	South Africa Vital Registration - Causes of Death 2011	Department of Home Affairs (South Africa), Statistics South Africa. South Africa Vital Registration - Causes of Death 2011. Pretoria, South Africa: Statistics South Africa.	21 - Mortality
204016	Vital registration	South Africa Vital Registration - Causes of Death 2012	Department of Home Affairs (South Africa), Statistics South Africa. South Africa Vital Registration - Causes of Death 2012. Pretoria,	21 - Mortality

			South Africa: Statistics South Africa.	
204017	Vital registration	South Africa Vital Registration - Causes of Death 2013	Department of Home Affairs (South Africa), Statistics South Africa. South Africa Vital Registration - Causes of Death 2013. Pretoria, South Africa: Statistics South Africa.	21 - Mortality
267740	Vital registration	South Africa Vital Registration - Causes of Death 2014	Department of Home Affairs (South Africa), Statistics South Africa. South Africa Vital Registration - Causes of Death 2014. Pretoria, South Africa: Statistics South Africa.	21 - Mortality
312271	Vital registration	South Africa Vital Registration - Causes of Death 2015	Department of Home Affairs (South Africa), Statistics South Africa. South Africa Vital Registration - Causes of Death 2015. Pretoria, South Africa: Statistics South Africa.	21 - Mortality
399147	Vital registration	South Africa Vital Registration - Causes of Death 2016	Department of Home Affairs (South Africa), Statistics South Africa. South Africa Vital Registration - Causes of Death 2016. Pretoria, South Africa: Statistics South Africa, 2019.	21 - Mortality
391090	Vital registration	South Africa Vital Registration Death Data 1993	South Africa Vital Registration Death Data 1993.	21 - Mortality
391091	Vital registration	South Africa Vital Registration Death Data 1994	South Africa Vital Registration Death Data 1994.	21 - Mortality
391092	Vital registration	South Africa Vital Registration Death Data 1995	South Africa Vital Registration Death Data 1995.	21 - Mortality
287600	Vital registration	WHO Mortality Database Version March 2017	World Health Organization (WHO). WHO Mortality Database Version March 2017. Geneva, Switzerland: World Health Organization (WHO).	21 - Mortality
12142	Census	South Africa Census 1996	Statistics South Africa. South Africa Census 1996. Pretoria, South Africa: Statistics South Africa.	22 - Fertility
43146	Census	South Africa Census 1996 - IPUMS	Central Statistical Service (South Africa), Minnesota Population Center. South Africa Census 1996 from the Integrated Public Use Microdata Series, International: [Machine-readable database]. Minneapolis: University of Minnesota.	22 - Fertility
43152	Census	South Africa Census 2001 - IPUMS	Statistics South Africa, Minnesota Population Center. South Africa Census 2001 from the Integrated Public Use Microdata Series, International: [Machine-readable database]. Minneapolis: University of Minnesota.	22 - Fertility
43158	Census	South Africa Community Survey 2007 - IPUMS	Statistics South Africa, Minnesota Population Center. South Africa Community Survey 2007 from the Integrated Public Use Microdata Series, International: [Machine-readable database]. Minneapolis: University of Minnesota.	22 - Fertility
12146	Census	South Africa Population and Housing Census 2011	Statistics South Africa. South Africa Population and Housing Census 2011.	22 - Fertility
227194	Census	South Africa Population and Housing Census 2011 - IPUMS	Minnesota Population Center, Statistics South Africa. South Africa Population and Housing Census 2011 from the Integrated Public Use Microdata Series, International: [Machine-readable database]. Minneapolis: University of Minnesota, 2015.	22 - Fertility
140201	Estimate	United Nations Demographic Yearbook - Historical Supplement 1997	United Nations Statistics Division (UNSD). United Nations Demographic Yearbook - Historical Supplement 1997. New York, United States of America: United Nations (UN).	22 - Fertility
52741	Estimate	United Nations Demographic Yearbook 2006	United Nations Statistics Division (UNSD). United Nations Demographic Yearbook 2006. New York, United States: United Nations (UN), 2008.	22 - Fertility
53065	Estimate	United Nations Demographic Yearbook 2009-2010	United Nations Statistics Division (UNSD). United Nations Demographic Yearbook 2009-2010. New York, United States: United Nations (UN), 2011.	22 - Fertility
121922	Estimate	United Nations Demographic Yearbook 2012	United Nations Statistics Division (UNSD). United Nations Demographic Yearbook 2012. New York, United States: United Nations (UN), 2013.	22 - Fertility
329653	Estimate	United Nations Demographic Yearbook 2016	United Nations Statistics Division (UNSD). United Nations Demographic Yearbook 2016. New York, United States of America: United Nations (UN), 2017.	22 - Fertility
11928	Survey	South Africa - KwaZulu and Natal Income Dynamics Study 1993	International Food Policy Research Institute (IFPRI), University of Natal, University of Wisconsin, Southern Africa Labour Development Research Unit (SALDRU), School of Economics, University of Cape Town. South Africa KwaZulu-Natal Income Dynamics Study 1993. Durban, South Africa: University of Natal.	22 - Fertility
280803	Survey	South Africa Community Survey 2016	Statistics South Africa. South Africa Community Survey 2016. Pretoria, South Africa: Statistics South Africa, 2016.	22 - Fertility
164898	Survey	South Africa Demographic and Health Survey 1987-1989	Human Sciences Research Council. South African Demographic and Health Survey, 1987 [Computer file]. S0115. Pretoria: Human Sciences Research Council [producer], 1987. Pretoria: South African Data Archive, National Research Foundation [distributor], 1999.	22 - Fertility

20796	Survey	South Africa Demographic and Health Survey 1998	Department of Health (South Africa), Macro International, Inc, South African Medical Research Council. South Africa Demographic and Health Survey 1998. Fairfax, United States of America: ICF International.	22 - Fertility
20798	Survey	South Africa Demographic and Health Survey 2003-2004	Department of Health (South Africa), Macro International, Inc, South African Medical Research Council. South Africa Demographic and Health Survey 2003-2004.	22 - Fertility
157064	Survey	South Africa Demographic and Health Survey 2016	Department of Health (South Africa), ICF International, South African Medical Research Council, Statistics South Africa. South Africa Demographic and Health Survey 2016. Fairfax, United States of America: ICF International, 2019.	22 - Fertility
115481	Survey	South Africa General Household Survey 2002	Statistics South Africa. South Africa General Household Survey 2002. Pretoria, South Africa: Statistics South Africa.	22 - Fertility
12101	Survey	South Africa Living Standards Measurement Study 1993	Southern Africa Labour Development Research Unit (SALDRU), University of Cape Town, World Bank. South Africa Living Standards Measurement Study 1993. Washington DC, United States of America: World Bank.	22 - Fertility
133731	Survey	South Africa National Income Dynamics Study - Wave 2 2010-2011	Southern Africa Labour and Development Research Unit. National Income Dynamics Study 2010-2011, Wave 2. Version 1.0. Cape Town: Southern Africa Labour and Development Research Unit [producer], 2012. Cape Town: DataFirst [distributor], 2013.	22 - Fertility
265153	Survey	South Africa National Income Dynamics Study - Wave 4 2014-2015	Southern Africa Labour and Development Research Unit. National Income Dynamics Study 2014 - 2015, Wave 4 [dataset]. Version 1.1. Cape Town: Southern Africa Labour and Development Research Unit [producer], 2016. Cape Town: DataFirst [distributor], 2016. Pretoria: Department of Planning Monitoring and Evaluation [commissioner], 2014	22 - Fertility
369644	Survey	South Africa National Income Dynamics Study - Wave 5 2017	Southern Africa Labour and Development Research Unit. National Income Dynamics Study 2017, Wave 5 [dataset]. Version 1.0.0 Pretoria: Department of Planning, Monitoring, and Evaluation [funding agency]. Cape Town: Southern Africa Labour and Development Research Unit [implementer], 2018. Cape Town: DataFirst [distributor], 2018.	22 - Fertility
12103	Survey	South Africa October Household Survey 1993	Central Statistical Service (South Africa). South Africa October Household Survey 1993.	22 - Fertility
105306	Survey	South Africa October Household Survey 1994	Central Statistical Service (South Africa). South Africa October Household Survey 1994.	22 - Fertility
106684	Survey	South Africa October Household Survey 1995	Central Statistical Service (South Africa). South Africa October Household Survey 1995.	22 - Fertility
106686	Survey	South Africa October Household Survey 1997	Central Statistical Service (South Africa). South Africa October Household Survey 1997.	22 - Fertility
12105	Survey	South Africa October Household Survey 1998	Statistics South Africa. South Africa October Household Survey 1998.	22 - Fertility
196426	Vital registration	South Africa Recorded Live Births 2001	Statistics South Africa. South Africa Recorded Live Births 2001. Pretoria, South Africa: Statistics South Africa.	22 - Fertility
196442	Vital registration	South Africa Recorded Live Births 2011	Statistics South Africa. South Africa Recorded Live Births 2011. Pretoria, South Africa: Statistics South Africa.	22 - Fertility
196443	Vital registration	South Africa Recorded Live Births 2012	Statistics South Africa. South Africa Recorded Live Births 2012. Pretoria, South Africa: Statistics South Africa.	22 - Fertility
196444	Vital registration	South Africa Recorded Live Births 2013	Statistics South Africa. South Africa Recorded Live Births 2013. Pretoria, South Africa: Statistics South Africa.	22 - Fertility
265154	Vital registration	South Africa Recorded Live Births 2014	Department of Home Affairs (South Africa), Statistics South Africa. South Africa Recorded Live Births 2014. Pretoria, South Africa: Statistics South Africa, 2015.	22 - Fertility
332923	Vital registration	South Africa Recorded Live Births 2016	Department of Home Affairs (South Africa), Statistics South Africa. South Africa Recorded Live Births 2016. Pretoria, South Africa: Statistics South Africa.	22 - Fertility
370115	Vital registration	South Africa Recorded Live Births 2017	Department of Home Affairs (South Africa), Statistics South Africa. South Africa Recorded Live Births 2017. Pretoria, South Africa: Statistics South Africa.	22 - Fertility
399147	Vital registration	South Africa Vital Registration - Causes of Death 2016	Department of Home Affairs (South Africa), Statistics South Africa. South Africa Vital Registration - Causes of Death 2016. Pretoria, South Africa: Statistics South Africa, 2019.	22 - Fertility
340517	Vital registration	WHO Mortality Database Version April 2018	World Health Organization (WHO). WHO Mortality Database Version April 2018. Geneva, Switzerland: World Health Organization (WHO).	22 - Fertility
340972	Census	South Africa Census 1951	South Africa Census 1951.	23 - Population

12139	Census	South Africa Census 1985	Statistics South Africa. South Africa Census 1985. Pretoria, South Africa: Statistics South Africa, 2003.	23 - Population
12142	Census	South Africa Census 1996	Statistics South Africa. South Africa Census 1996. Pretoria, South Africa: Statistics South Africa.	23 - Population
43146	Census	South Africa Census 1996 - IPUMS	Central Statistical Service (South Africa), Minnesota Population Center. South Africa Census 1996 from the Integrated Public Use Microdata Series, International: [Machine-readable database]. Minneapolis: University of Minnesota.	23 - Population
12144	Census	South Africa Census 2001	Statistics South Africa. South Africa Census 2001. Pretoria, South Africa: Statistics South Africa.	23 - Population
43152	Census	South Africa Census 2001 - IPUMS	Statistics South Africa, Minnesota Population Center. South Africa Census 2001 from the Integrated Public Use Microdata Series, International: [Machine-readable database]. Minneapolis: University of Minnesota.	23 - Population
12136	Census	South Africa Population and Housing Census 1960	Office of Census and Statistics (Union of South Africa). South Africa Population and Housing Census 1960. Cape Town, South Africa: DataFirst.	23 - Population
12137	Census	South Africa Population and Housing Census 1970	Department of Statistics (South Africa). South Africa Population and Housing Census 1970.	23 - Population
12138	Census	South Africa Population and Housing Census 1980	Central Statistical Service (South Africa). South Africa Population and Housing Census 1980.	23 - Population
12140	Census	South Africa Population and Housing Census 1991	Central Statistical Service (South Africa). South Africa Population and Housing Census 1991. Cape Town, South Africa: DataFirst.	23 - Population
12146	Census	South Africa Population and Housing Census 2011	Statistics South Africa. South Africa Population and Housing Census 2011.	23 - Population
227194	Census	South Africa Population and Housing Census 2011 - IPUMS	Minnesota Population Center, Statistics South Africa. South Africa Population and Housing Census 2011 from the Integrated Public Use Microdata Series, International: [Machine-readable database]. Minneapolis: University of Minnesota, 2015.	23 - Population
419891	Administrative data	WHO Distribution of Measles Cases by Country and by Month 2011-2020	World Health Organization (WHO). WHO Distribution of Measles Cases by Country and by Month 2011-2020.	4 - Causes of Death
155336	Demographic surveillance	Africa, Asia, Oceania - INDEPTH Network Cause-Specific Mortality - Release 2014	INDEPTH. Africa, Asia, Oceania - INDEPTH Network Cause-Specific Mortality - Release 2014. Accra, Ghana: INDEPTH, 2014.	4 - Causes of Death
394317	Demographic surveillance	Child Health and Mortality Prevention Surveillance Network Program	Centers for Disease Control and Prevention (CDC), Child Health and Mortality Prevention Surveillance (CHAMPS) Network, Deloitte Consulting LLP, Emory Global Health Institute (EGHI), International Association of National Public Health Institutes (IANPHI), Task Force for Global Health, Emory University. Child Health and Mortality Prevention Surveillance (CHAMPS) Network Program.	4 - Causes of Death
93667	Demographic surveillance	Contributions of the Africa Centre Demographic Surveillance to the Community	Muhwava W. Contributions of the Africa Centre Demographic Surveillance to the Community. Umbiko. 2011; 12. 3-4.	4 - Causes of Death
11781	Demographic surveillance	South Africa - Agincourt Health and Socio-Demographic Surveillance System	MRC/Wits Rural Public Health and Health Transitions Research Unit (Agincourt), INDEPTH. South Africa - Agincourt Health and Socio-Demographic Surveillance System.	4 - Causes of Death
114558	Disease registry	Cancer in South Africa Full Report 2003	National Cancer Registry (South Africa). Cancer in South Africa Full Report 2003 . Johannesburg, South Africa: National Institute for Occupational Health (South Africa).	4 - Causes of Death
114560	Disease registry	Cancer in South Africa Full Report 2004	National Cancer Registry (South Africa). Cancer in South Africa Full Report 2004. Johannesburg, South Africa: National Institute for Occupational Health (South Africa).	4 - Causes of Death
264234	Disease registry	Cancer in South Africa Full Report 2011	National Cancer Registry (South Africa). Cancer in South Africa Full Report 2011. Johannesburg, South Africa: National Cancer Registry (South Africa).	4 - Causes of Death
116086	Disease registry	Cancer Incidence in Five Continents Volume X Summary Database 2003-2007	Forman D, Bray F, Brewster DH, Gombe Mbalawa C, Kohler B, PiÃ±eros M, Steliarova-Foucher E, Swaminathan R and Ferlay J, eds (2013). Cancer Incidence in Five Continents, Vol. X Summary Database (electronic version). Lyon, IARC. http://ci5.iarc.fr	4 - Causes of Death
318157	Disease registry	Cancer Incidence in Five Continents Volume XI 2008-2012	Bray F, Colombet M, Mery L, PiÃ±eros M, Znaor A, Zanetti R and Ferlay J, editors (2017). Cancer Incidence in Five Continents, Vol. XI (electronic version). Lyon: International Agency for Research on Cancer. Available from: http://ci5.iarc.fr	4 - Causes of Death
113829	Disease registry	South Africa National Cancer Registry Incidence 2005	National Cancer Registry (South Africa). South Africa National Cancer Registry Incidence 2005. Johannesburg, South Africa: National Institute for Occupational Health (South Africa).	4 - Causes of Death
128995	Disease registry	South Africa PROMEC Cancer	PROMEC Unit, South African Medical Research Council. South	4 - Causes of

		Registry Incidence 1998-2002	Africa PROMEC Cancer Registry Incidence 1998-2002.	Death
340570	Epi surveillance	Compendium of Short Reports on Selected Outbreaks in the WHO African Region, October 2017	World Health Organization Regional Office for Africa (WHO-AFRO). Compendium of Short Reports on Selected Outbreaks in the WHO African Region, October 2017. Brazaville, Congo: World Health Organization Regional Office for Africa (WHO-AFRO), 2017.	4 - Causes of Death
229843	Epi surveillance	South Africa GERMS-SA Typhoid and Paratyphoid Tabulations 2003	National Institute for Communicable Diseases (South Africa). South Africa GERMS-SA Typhoid and Paratyphoid Tabulations 2003.	4 - Causes of Death
229844	Epi surveillance	South Africa GERMS-SA Typhoid and Paratyphoid Tabulations 2004	National Institute for Communicable Diseases (South Africa). South Africa GERMS-SA Typhoid and Paratyphoid Tabulations 2004.	4 - Causes of Death
229845	Epi surveillance	South Africa GERMS-SA Typhoid and Paratyphoid Tabulations 2005	National Institute for Communicable Diseases (South Africa). South Africa GERMS-SA Typhoid and Paratyphoid Tabulations 2005.	4 - Causes of Death
229846	Epi surveillance	South Africa GERMS-SA Typhoid and Paratyphoid Tabulations 2006	National Institute for Communicable Diseases (South Africa). South Africa GERMS-SA Typhoid and Paratyphoid Tabulations 2006.	4 - Causes of Death
229847	Epi surveillance	South Africa GERMS-SA Typhoid and Paratyphoid Tabulations 2007	National Institute for Communicable Diseases (South Africa). South Africa GERMS-SA Typhoid and Paratyphoid Tabulations 2007.	4 - Causes of Death
229848	Epi surveillance	South Africa GERMS-SA Typhoid and Paratyphoid Tabulations 2008	National Institute for Communicable Diseases (South Africa). South Africa GERMS-SA Typhoid and Paratyphoid Tabulations 2008.	4 - Causes of Death
229849	Epi surveillance	South Africa GERMS-SA Typhoid and Paratyphoid Tabulations 2009	National Institute for Communicable Diseases (South Africa). South Africa GERMS-SA Typhoid and Paratyphoid Tabulations 2009.	4 - Causes of Death
229850	Epi surveillance	South Africa GERMS-SA Typhoid and Paratyphoid Tabulations 2010	National Institute for Communicable Diseases (South Africa). South Africa GERMS-SA Typhoid and Paratyphoid Tabulations 2010.	4 - Causes of Death
229851	Epi surveillance	South Africa GERMS-SA Typhoid and Paratyphoid Tabulations 2011	National Institute for Communicable Diseases (South Africa). South Africa GERMS-SA Typhoid and Paratyphoid Tabulations 2011.	4 - Causes of Death
229852	Epi surveillance	South Africa GERMS-SA Typhoid and Paratyphoid Tabulations 2012	National Institute for Communicable Diseases (South Africa). South Africa GERMS-SA Typhoid and Paratyphoid Tabulations 2012.	4 - Causes of Death
229853	Epi surveillance	South Africa GERMS-SA Typhoid and Paratyphoid Tabulations 2013	National Institute for Communicable Diseases (South Africa). South Africa GERMS-SA Typhoid and Paratyphoid Tabulations 2013.	4 - Causes of Death
229854	Epi surveillance	South Africa GERMS-SA Typhoid and Paratyphoid Tabulations 2014	National Institute for Communicable Diseases (South Africa). South Africa GERMS-SA Typhoid and Paratyphoid Tabulations 2014.	4 - Causes of Death
340584	Epi surveillance	Weekly Bulletins on Outbreaks and Other Emergencies 2017	World Health Organization Regional Office for Africa (WHO-AFRO). Weekly Bulletins on Outbreaks and Other Emergencies 2017. Brazaville, Congo: World Health Organization Regional Office for Africa (WHO-AFRO), 2017.	4 - Causes of Death
340865	Epi surveillance	Weekly Bulletins on Outbreaks and Other Emergencies 2018	World Health Organization Regional Office for Africa (WHO-AFRO). Weekly Bulletins on Outbreaks and Other Emergencies 2018. Brazaville , Congo: World Health Organization Regional Office for Africa (WHO-AFRO), 2018.	4 - Causes of Death
83133	Epi surveillance	WHO and UNICEF Reported Disease Incidence Time Series	United Nations Children's Fund (UNICEF), World Health Organization (WHO). WHO and UNICEF Reported Disease Incidence Time Series. Geneva, Switzerland: World Health Organization (WHO).	4 - Causes of Death
303730	Epi surveillance	WHO Global Health Observatory - Cholera: Number of Reported Deaths by Country	World Health Organization (WHO). WHO Global Health Observatory - Cholera: Number of Reported Deaths by Country. Geneva, Switzerland: World Health Organization (WHO).	4 - Causes of Death
126384	Epi surveillance	WHO Tuberculosis Case Notifications	World Health Organization (WHO). WHO Tuberculosis Case Notifications. Geneva, Switzerland: World Health Organization (WHO).	4 - Causes of Death
96480	Estimate	Sickle Cell and Thalassemias Prevalence Data, Personal Correspondence with David Weatherall	Weatherall D. Sickle Cell and Thalassemias Prevalence Data, Personal Correspondence with David Weatherall. [Unpublished].	4 - Causes of Death
135380	Event data	Armed Conflict Location and Event Dataset, Realtime - Robert S. Strauss Center	Climate Change and African Political Stability Project (CCAPS). Armed Conflict Location and Event Dataset, Realtime - Robert S. Strauss Center as referenced in Raleigh, Clionadh, Andrew Linke, Havard Hegre and Joakim Karlsen. 2010. Introducing ACLED-Armed Conflict Location and Event Data. Journal of Peace Research 47(5), 651-60.	4 - Causes of Death
327302	Event data	Battle Deaths Dataset Version 3.1, 2009	Peace Research Institute Oslo (PRIO). Battle Deaths Dataset Version 3.1, 2009. Oslo, Norway: Peace Research Institute Oslo (PRIO), 2009.	4 - Causes of Death
13769	Event data	EM-DAT: The OFDA/CRED International Disaster Database	Centre for Research on the Epidemiology of Disasters (CRED). EM-DAT: The OFDA/CRED International Disaster Database. Brussels, Belgium: Catholic University of Leuven.	4 - Causes of Death
427585	Event data	Floods Kill At Least 60 In Africa (Durban, South Africa)	de Greef K. Floods Kill At Least 60 In Africa (Durban, South Africa). New York Times [Internet]. 2019 Apr 24; Africa.	4 - Causes of Death
328214	Event data	Global Terrorism Database	National Consortium for the Study of Terrorism and Responses to Terrorism (START). Global Terrorism Database. College Park , MD,	4 - Causes of Death

			United States of America: University of Maryland, 2018.	
283521	Event data	List of Terrorist Incidents in 2016	Wikipedia. List of Terrorist Incidents in 2016. San Francisco, United States of America: Wikipedia.	4 - Causes of Death
326896	Event data	UCDP Georeferenced Event Dataset, Version 17.1, 2016	Department of Peace and Conflict Research, Uppsala University. UCDP Georeferenced Event Dataset, Version 17.1, 2016. Uppsala, Sweden: Department of Peace and Conflict Research, Uppsala University, 2017.	4 - Causes of Death
284465	Modeled data	IHME GBD Cancer Incidence and Mortality Estimates	Institute for Health Metrics and Evaluation (IHME). IHME GBD Cancer Incidence and Mortality Estimates.	4 - Causes of Death
143198	Report	HIV/AIDS Epidemiological Surveillance Update for the WHO African Region 2002	World Health Organization Regional Office for Africa (WHO-AFRO). HIV/AIDS Epidemiological Surveillance Update for the WHO African Region 2002. Brazaville , Congo: World Health Organization Regional Office for Africa (WHO-AFRO), 2003.	4 - Causes of Death
299749	Report	South Africa District Health Barometer 2015-2016	Health Systems Trust. South Africa District Health Barometer 2015-2016. Durban, South Africa: Health Systems Trust, 2016.	4 - Causes of Death
154459	Report	The Future of the Global Muslim Population	Pew Research Center. The Future of the Global Muslim Population. Washington, DC, United States: Pew Research Center, 2011.	4 - Causes of Death
342867	Report	WHO International Travel and Health, Annex 1, Countries With Risk of Yellow Fever Transmission and Countries Requiring Yellow Fever Vaccination 2017 Update	World Health Organization (WHO). WHO International Travel and Health, Annex 1, Countries With Risk of Yellow Fever Transmission and Countries Requiring Yellow Fever Vaccination 2017 Update. Geneva, Switzerland: World Health Organization (WHO), 2017.	4 - Causes of Death
93640	Scientific literature	'A bothersome death' -- narrative accounts of infant mortality in Cape Town, South Africa	Lerer LB, Butchart A, Blanche MT. 'A bothersome death' -- narrative accounts of infant mortality in Cape Town, South Africa. Soc Sci Med. 1995; 40(7): 945-53.	4 - Causes of Death
124755	Scientific literature	A review of maternal deaths in South Africa during 1998. National Committee on Confidential Enquiries into Maternal Deaths	Moodley J. A review of maternal deaths in South Africa during 1998. National Committee on Confidential Enquiries into Maternal Deaths. S Afr Med J. 2000; 90(4): 367-73.	4 - Causes of Death
214569	Scientific literature	Adherence to highly active antiretroviral therapy assessed by pharmacy claims predicts survival in HIV-infected South African adults	Nachega JB, Hislop M, Dowdy DW, Lo M, Omer SB, Regensberg L, Chaisson RE, Maartens G. Adherence to highly active antiretroviral therapy assessed by pharmacy claims predicts survival in HIV-infected South African adults. J Acquir Immune Defic Syndr. 2006; 43(1): 78-84.	4 - Causes of Death
422973	Scientific literature	Aetiology and outcome of pneumonia in human immunodeficiency virus-infected children hospitalized in South Africa	Zar HJ, Hanslo D, Tannenbaum E, Klein M, Argent A, Eley B, Burgess J, Magnus K, Bateman ED, Hussey G. Aetiology and outcome of pneumonia in human immunodeficiency virus-infected children hospitalized in South Africa. Acta Paediatr. 2001; 90(2): 119-25.	4 - Causes of Death
267580	Scientific literature	Age in antiretroviral therapy programmes in South Africa: a retrospective, multicentre, observational cohort study	Cornell M, Johnson LF, Schomaker M, Tanser F, Maskew M, Wood R, Prozesky H, Giddy J, Stinson K, Egger M, Boulle A, Myer L, International Epidemiologic Databases to Evaluate AIDS-Southern Africa Collaboration. Age in antiretroviral therapy programmes in South Africa: a retrospective, multicentre, observational cohort study. Lancet HIV. 2015; 2(9): e368-375.	4 - Causes of Death
335046	Scientific literature	All-cause mortality in HIV-positive adults starting combination antiretroviral therapy: correcting for loss to follow-up	Anderegg N, Johnson LF, Zaniewski E, Althoff KN, Balestre E, Law M, Nash D, Shepherd BE, Yiannoutsos CT, Egger M, IeDEA, MeSH consortia. All-cause mortality in HIV-positive adults starting combination antiretroviral therapy: correcting for loss to follow-up. AIDS. 2017; 31 Suppl 1: S31â€“40.	4 - Causes of Death
317971	Scientific literature	An association between decreasing incidence of invasive non-typhoidal salmonellosis and increased use of antiretroviral therapy, Gauteng Province, South Africa, 2003â€“2013. Vermund SH, editor	Keddy KH, Takuva S, Musekiwa A, Puren AJ, Sooka A, Karstaedt A, Klugman KP, Angulo FJ. An association between decreasing incidence of invasive non-typhoidal salmonellosis and increased use of antiretroviral therapy, Gauteng Province, South Africa, 2003â€“2013. Vermund SH, editor. PLoS One. 2017; 12(3): e0173091.	4 - Causes of Death
209344	Scientific literature	Antiretroviral Treatment Outcomes amongst Older Adults in a Large Multicentre Cohort in South Africa	Fatti G, Mothibi E, Meintjes G, Grimwood A. Antiretroviral Treatment Outcomes amongst Older Adults in a Large Multicentre Cohort in South Africa. PLoS One. 2014; 9(6): e100273.	4 - Causes of Death
139385	Scientific literature	Bacterial meningitis in children at Kalafong Hospital, 1990-1995	Grobler AC, Hay IT. Bacterial meningitis in children at Kalafong Hospital, 1990-1995. S Afr Med J. 1997; 87(8 Suppl): 1052-4.	4 - Causes of Death
214505	Scientific literature	Better antiretroviral therapy outcomes at primary healthcare facilities: an evaluation of three tiers of ART services in four South African provinces	Fatti G, Grimwood A, Bock P. Better antiretroviral therapy outcomes at primary healthcare facilities: an evaluation of three tiers of ART services in four South African provinces. PLoS One. 2010; 5(9): e12888.	4 - Causes of Death
402198	Scientific literature	Cancer of childhood in sub-Saharan Africa	Stefan C, Bray F, Ferlay J, Liu B, Maxwell Parkin D. Cancer of childhood in sub-Saharan Africa. ecancer. 2017; 11: 755.	4 - Causes of Death

351083	Scientific literature	Cardiac function in an African dialysis population with a low prevalence of pre-existing cardiovascular disease	Luyckx VA, Yip A, Sofianou L, Jhangri GS, Mueller TF, Naicker S. Cardiac function in an African dialysis population with a low prevalence of pre-existing cardiovascular disease. Ren Fail. 2009; 31(3): 211â€“20.	4 - Causes of Death
214553	Scientific literature	CD4 count slope and mortality in HIV-infected patients on antiretroviral therapy: multicohort analysis from South Africa	Hoffmann CJ, Schomaker M, Fox MP, Mutevedzi P, Giddy J, Prozesky H, Wood R, Garone DB, Egger M, Boulle A, IeDEA Southern Africa Collaboration. CD4 count slope and mortality in HIV-infected patients on antiretroviral therapy: multicohort analysis from South Africa. J Acquir Immune Defic Syndr. 2013; 63(1): 34-41.	4 - Causes of Death
214519	Scientific literature	Changes in programmatic outcomes during 7 years of scale-up at a community-based antiretroviral treatment service in South Africa	Nglazi MD, Lawn SD, Kaplan R, Kranzer K, Orrell C, Wood R, Bekker L-G. Changes in programmatic outcomes during 7 years of scale-up at a community-based antiretroviral treatment service in South Africa. J Acquir Immune Defic Syndr. 2011; 56(1): e1-8.	4 - Causes of Death
214535	Scientific literature	Changing predictors of mortality over time from cART start: implications for care	Hoffmann CJ, Fielding KL, Johnston V, Charalambous S, Innes C, Moore RD, Chaisson RE, Grant AD, Churchyard GJ. Changing predictors of mortality over time from cART start: implications for care. J Acquir Immune Defic Syndr. 2011; 58(3): 269-76.	4 - Causes of Death
214556	Scientific literature	Comparison of tenofovir, zidovudine, or stavudine as part of first-line antiretroviral therapy in a resource-limited-setting: a cohort study	Velen K, Lewis JJ, Charalambous S, Grant AD, Churchyard GJ, Hoffmann CJ. Comparison of tenofovir, zidovudine, or stavudine as part of first-line antiretroviral therapy in a resource-limited-setting: a cohort study. PLoS One. 2013; 8(5): e64459.	4 - Causes of Death
257166	Scientific literature	Cystic Echinococcosis Endemicity Estimates	Cystic Echinococcosis Endemicity Estimates identified through systematic review and personal communication, as provided by GBD 2015 expert. [Unpublished].	4 - Causes of Death
107297	Scientific literature	Determining causes of mortality in children enrolled in a vaccine field trial in a rural area in the Western Cape Province of South Africa	Moyo S, Hawkridge T, Mahomed H, Workman L, Minnies D, Geiter LJ, Verver S, Kibel M, Hussey GD. Determining causes of mortality in children enrolled in a vaccine field trial in a rural area in the Western Cape Province of South Africa. J Paediatr Child Health. 2007; 43(3): 178-83.	4 - Causes of Death
293926	Scientific literature	Dietary iron overload as a risk factor for hepatocellular carcinoma in Black Africans	Mandishona E, MacPhail AP, Gordeuk VR, Kedda M-A, Paterson AC, Rouault TA, Kew MC. Dietary iron overload as a risk factor for hepatocellular carcinoma in Black Africans . Hepatology. 1998; 27(6): 1563-6.	4 - Causes of Death
214508	Scientific literature	Early loss of HIV-infected patients on potent antiretroviral therapy programmes in lower-income countries	Brinkhof MWG, Dabis F, Myer L, Bangsberg DR, Boulle A, Nash D, Schechter M, Laurent C, Keiser O, May M, Sprinz E, Egger M, Anglaret X, ART-LINC, IeDEA. Early loss of HIV-infected patients on potent antiretroviral therapy programmes in lower-income countries. Bull World Health Organ. 2008; 86(7): 559-67.	4 - Causes of Death
156912	Scientific literature	Effect of HIV infection on pregnancy-related mortality in sub-Saharan Africa: secondary analyses of pooled community-based data from the network for Analysing Longitudinal Population-based HIV/AIDS data on Africa (ALPHA)	Zaba B, Calvert C, Marston M, Isingo R, Nakiyingi-Miiro J, Lutalo T, Crampin A, Robertson L, Herbst K, Newell M-L, Todd J, Byass P, Boerma T, Ronsmans C. Effect of HIV infection on pregnancy-related mortality in sub-Saharan Africa: secondary analyses of pooled community-based data from the network for Analysing Longitudinal Population-based HIV/AIDS data on Africa (ALPHA). Lancet. 2013; 381(9879): 1763-71.	4 - Causes of Death
143220	Scientific literature	Epidemiology ofÂ syphilisÂ inÂ pregnancyÂ in rural South Africa: opportunities for control	Wilkinson D,Â Sach M,Â Connolly C. Epidemiology ofÂ syphilisÂ inÂ pregnancyÂ in rural South Africa: opportunities for control. Trop Med Int Health. 1997; 2(1): 57-62.	4 - Causes of Death
307804	Scientific literature	Factors associated with an increased case-fatality rate in HIV-infected and non-infected South African gold miners with pulmonary tuberculosis	Churchyard GJ, Kleinschmidt I, Corbett EL, Murray J, Smit J, De Cock KM. Factors associated with an increased case-fatality rate in HIV-infected and non-infected South African gold miners with pulmonary tuberculosis. Int J Tuberc Lung Dis. 2000; 4(8): 705â€“712.	4 - Causes of Death
209317	Scientific literature	Factors associated with mortality in HIV-infected people in rural and urban South Africa	Otwombe KN, Petzold M, Modisenyane T, Martinson NA, Chirwa T. Factors associated with mortality in HIV-infected people in rural and urban South Africa. Glob Health Action. 2014; 25488.	4 - Causes of Death
214502	Scientific literature	Gender differences in survival among adult patients starting antiretroviral therapy in South Africa: a multicentre cohort study	Cornell M, Schomaker M, Garone DB, Giddy J, Hoffmann CJ, Lessells R, Maskew M, Prozesky H, Wood R, Johnson LF, Egger M, Boulle A, Myer L, International Epidemiologic Databases to Evaluate AIDS Southern Africa Collaboration. Gender differences in survival among adult patients starting antiretroviral therapy in South Africa: a multicentre cohort study. PLoS Med. 2012; 9(9): e1001304.	4 - Causes of Death
125860	Scientific literature	Hepatitis C virus infection in chronic liver disease in Natal	Soni PN, Tait DR, Gopaul W, Sathar MA, Simjee AE. Hepatitis C virus infection in chronic liver disease in Natal. S Afr Med J. 1996; 86(1): 80-3.	4 - Causes of Death
236777	Scientific literature	HIV infection in critically ill obstetrical patients	de Groot MR, Corporaal LJ, CronjÃ© HS, Joubert G. HIV infection in critically ill obstetrical patients. Int J Gynaecol Obstet. 2003;	4 - Causes of Death

			81(1): 9-16.	
307836	Scientific literature	Human immunodeficiency virus and the outcome of treatment for new and recurrent pulmonary tuberculosis in African patients	Murray J, Sonnenberg P, Shearer SC, Godfrey-Faussett P. Human immunodeficiency virus and the outcome of treatment for new and recurrent pulmonary tuberculosis in African patients. Am J Respir Crit Care Med. 1999; 159(3): 733â€“740.	4 - Causes of Death
293963	Scientific literature	Immunogenicity, efficacy and serological correlate of protection of Salmonella typhi Vi capsular polysaccharide vaccine three years after immunization	Klugman KP, Koornhof HJ, Robbins JB, Le Cam NN. Immunogenicity, efficacy and serological correlate of protection of Salmonella typhi Vi capsular polysaccharide vaccine three years after immunization. Vaccine. 1996; 14(5): 435â€“8.	4 - Causes of Death
141713	Scientific literature	ImpactÂ of theÂ 1996-1997Â supplementaryÂ measlesÂ vaccinationÂ campaignsÂ inÂ South Africa	Uzicanin A,Â Eggers R,Â Webb E,Â Harris B,Â Durrheim D,Â Ogunbanjo G,Â Isaacs V,Â Hawkridge A,Â Biellik R,Â Strebel P. ImpactÂ of theÂ 1996-1997Â supplementaryÂ measlesÂ vaccinationÂ campaignsÂ inÂ South Africa. Int J Epidemiol. 2002; 31(5): 968-76.	4 - Causes of Death
317974	Scientific literature	Incidence of invasive salmonella disease in sub-Saharan Africa: a multicentre population-based surveillance study	Marks F, von Kalckreuth V, Aaby P, Adu-Sarkodie Y, El Tayeb MA, Ali M, Aseffa A, Baker S, Biggs HM, Bjerregaard-Andersen M, others. Incidence of invasive salmonella disease in sub-Saharan Africa: a multicentre population-based surveillance study. Lancet Glob Health. 2017; 5(3): e310-323.	4 - Causes of Death
422961	Scientific literature	Increased disease burden and antibiotic resistance of bacteria causing severe community-acquired lower respiratory tract infections in human immunodeficiency virus type 1-infected children	Madhi SA, Petersen K, Madhi A, Khoosal M, Klugman KP. Increased disease burden and antibiotic resistance of bacteria causing severe community-acquired lower respiratory tract infections in human immunodeficiency virus type 1-infected children. Clin Infect Dis. 2000; 31(1): 170-6.	4 - Causes of Death
110035	Scientific literature	Inherited haemoglobin variants in a South African population	Bird AR, Ellis P, Wood K, Mathew C, Karabus C. Inherited haemoglobin variants in a South African population. J Med Genet. 1987; 24(4): 215-9.	4 - Causes of Death
107310	Scientific literature	Injury mortality in rural South Africa 2000 - 2007: rates and associated factors	Garrib A, Herbst AJ, Hosegood V, Newell M-L. Injury mortality in rural South Africa 2000 - 2007: rates and associated factors. Trop Med Int Health. 2011; 16(4): 439-46.	4 - Causes of Death
408097	Scientific literature	Invasive Pneumococcal Disease in Neonates Prior to Pneumococcal Conjugate Vaccine Use in South Africa: 2003-2008	Moodley K, Coovadia YM, Cohen C, Meiring S, Lengana S, De Gouveia L, von Mollendorf C, Crowther-Gibson P, Quan V, Eley B, Reubenson G, Nana T, von Gottberg A. Invasive Pneumococcal Disease in Neonates Prior to Pneumococcal Conjugate Vaccine Use in South Africa: 2003-2008. Pediatr Infect Dis J. 2019; 38(4): 424-430.	4 - Causes of Death
214900	Scientific literature	Long term outcomes of antiretroviral therapy in a large HIV/AIDS care clinic in urban South Africa: a prospective cohort study	Sanne IM, Westreich D, Macphail AP, Rubel D, Majuba P, Van Rie A. Long term outcomes of antiretroviral therapy in a large HIV/AIDS care clinic in urban South Africa: a prospective cohort study. J Int AIDS Soc. 2009; 38.	4 - Causes of Death
214515	Scientific literature	Long-term antiretroviral treatment outcomes in seven countries in the Caribbean	Koenig SP, Rodriguez LA, Bartholomew C, Edwards A, Carmichael TE, Barrow G, CabiÃ© A, Hunter R, Vasquez-Mora G, Quava-Jones A, Adomakoh N, Peter Figueroa J, Liautaud B, Torres M, Pape JW. Long-term antiretroviral treatment outcomes in seven countries in the Caribbean. J Acquir Immune Defic Syndr. 2012; 59(4): e60-71.	4 - Causes of Death
214531	Scientific literature	Low haemoglobin predicts early mortality among adults starting antiretroviral therapy in an HIV care programme in South Africa: a cohort study	Russell EC, Charalambous S, Pemba L, Churchyard GJ, Grant AD, Fielding K. Low haemoglobin predicts early mortality among adults starting antiretroviral therapy in an HIV care programme in South Africa: a cohort study. BMC Public Health. 2010; 433.	4 - Causes of Death
124814	Scientific literature	Maternal deaths at King Edward VIII Hospital, Durban. A review of 258 consecutive cases	Melrose EB. Maternal deaths at King Edward VIII Hospital, Durban. A review of 258 consecutive cases. S Afr Med J. 1984; 65(5): 161-5.	4 - Causes of Death
236768	Scientific literature	Maternal mortality associated with tuberculosis-HIV-1 co-infection in Durban, South Africa	Khan M, Pillay T, Moodley JM, Connolly CA, Durban Perinatal TB HIV-1 Study Group. Maternal mortality associated with tuberculosis-HIV-1 co-infection in Durban, South Africa. AIDS. 2001; 15(14): 1857-1863.	4 - Causes of Death
334495	Scientific literature	Measles in a South African paediatric intensive care unit: Again!	Coetzee S, Morrow BM, Argent AC. Measles in a South African paediatric intensive care unit: Again!. J Paediatr Child Health. 2014; 50: 379â€“85.	4 - Causes of Death
93645	Scientific literature	Mortality and loss to follow-up among HAART initiators in rural South Africa	MacPherson P, Moshabela M, Martinson N, Pronyk P. Mortality and loss to follow-up among HAART initiators in rural South Africa. Trans R Soc Trop Med Hyg. 2009; 103(6): 588-93.	4 - Causes of Death
214568	Scientific literature	Mortality and morbidity among HIV type-1-infected patients during the first 5 years of a multicountry HIV	Van der Borght SF, Clevenbergh P, Rijckborst H, Nsalou P, Onyia N, Lange JM, de Wit TFR, Van der Loeff MFS. Mortality and morbidity among HIV type-1-infected patients during the first 5	4 - Causes of Death

		workplace programme in Africa	years of a multicountry HIV workplace programme in Africa. Antivir Ther. 2009; 14(1): 63-74.	
214562	Scientific literature	Mortality during the first year of potent antiretroviral therapy in HIV-1-infected patients in 7 sites throughout Latin America and the Caribbean	Tuboi SH, Schechter M, McGowan CC, Cesar C, Krolewiecki A, Cahn P, Wolff M, Pape JW, Padgett D, Madero JS, Gotuzzo E, Masys DR, Shepherd BE. Mortality during the first year of potent antiretroviral therapy in HIV-1-infected patients in 7 sites throughout Latin America and the Caribbean. J Acquir Immune Defic Syndr. 2009; 51(5): 615-23.	4 - Causes of Death
209349	Scientific literature	Mortality in patients with HIV-1 infection starting antiretroviral therapy in South Africa, Europe, or North America: a collaborative analysis of prospective studies	Boulle A, Schomaker M, May MT, Hogg RS, Shepherd BE, Monge S, Keiser O, Lampe FC, Giddy J, Ndirangu J, Garone D, Fox M, Ingle SM, Reiss P, Dabis F, Costagliola D, Castagna A, Ehren K, Campbell C, Gill MJ, Saag M, Justice AC, Guest J, Crane HM, Egger M, Sterne JAC. Mortality in patients with HIV-1 infection starting antiretroviral therapy in South Africa, Europe, or North America: a collaborative analysis of prospective studies. PLoS Med. 2014; 11(9): e1001718.	4 - Causes of Death
156877	Scientific literature	Mortality in women of reproductive age in rural South Africa	Nabukalu D, Klipstein-Grobusch K, Herbst K, Newell M-L. Mortality in women of reproductive age in rural South Africa. Glob Health Action. 2013; 22834.	4 - Causes of Death
214567	Scientific literature	Mortality of HIV-infected patients starting antiretroviral therapy in sub-Saharan Africa: comparison with HIV-unrelated mortality	Brinkhof MWG, Boulle A, Weigel R, Messou E, Mathers C, Orrell C, Dabis F, Pascoe M, Egger M, International Epidemiological Databases to Evaluate AIDS (IeDEA). Mortality of HIV-infected patients starting antiretroviral therapy in sub-Saharan Africa: comparison with HIV-unrelated mortality. PLoS Med. 2009; 6(4): e1000066.	4 - Causes of Death
128455	Scientific literature	Occurrence and clinical implications of red-cell glucose-6-phosphate dehydrogenase deficiency in South African racial groups	Bernstein RE. Occurrence and clinical implications of red-cell glucose-6-phosphate dehydrogenase deficiency in South African racial groups. S Afr Med J. 1963; 447-51.	4 - Causes of Death
143219	Scientific literature	On-site rapid antenatal syphilis screening with an immunochromatographic strip improves case detection and treatment in rural South African clinics	Bronzan RN,Â Mwesigwa-Kayongo DC,Â Narkunas D,Â Schmid GP,Â Neilsen GA,Â Ballard RC,Â Karuhije P,Â Ddamba J,Â Nombekela E,Â Hoyi G,Â Dlali P,Â Makwedini N,Fehler HG,Â Blandford JM,Â Ryan C. On-site rapid antenatal syphilis screening with an immunochromatographic strip improves case detection and treatment in rural South African clinics. Sex Transm Dis. 2007; 34(7 Suppl): S55-60.	4 - Causes of Death
214903	Scientific literature	Outcomes of antiretroviral treatment in programmes with and without routine viral load monitoring in Southern Africa	Keiser O, Chi BH, Gsponer T, Boulle A, Orrell C, Phiri S, Maxwell N, Maskew M, Prozesky H, Fox MP, Westfall A, Egger M, IeDEA Southern Africa Collaboration. Outcomes of antiretroviral treatment in programmes with and without routine viral load monitoring in Southern Africa. AIDS. 2011; 25(14): 1761-9.	4 - Causes of Death
214911	Scientific literature	Outcomes of antiretroviral treatment program in Ethiopia: retention of patients in care is a major challenge and varies across health facilities	Assefa Y, Kiflie A, Tesfaye D, Mariam DH, Kloos H, Edwin W, Laga M, Van Damme W. Outcomes of antiretroviral treatment program in Ethiopia: retention of patients in care is a major challenge and varies across health facilities. BMC Health Serv Res. 2011; 81.	4 - Causes of Death
214514	Scientific literature	Outcomes of antiretroviral treatment programs in rural Southern Africa	Wandeler G, Keiser O, Pfeiffer K, Pestilli S, Fritz C, Labhardt ND, Mbofana F, Mudyiradima R, Emmel J, Egger M, Ehmer J, SolidarMed ART program and IeDEA-Southern Africa. Outcomes of antiretroviral treatment programs in rural Southern Africa. J Acquir Immune Defic Syndr. 2012; 59(2): e9-16.	4 - Causes of Death
307876	Scientific literature	Predictors of mortality and treatment success during treatment for rifampicin-resistant tuberculosis within the South African National TB Programme, 2009 to 2011: a cohort analysis of the national case register	Schnippel K, Shearer K, Evans D, Berhanu R, Dlamini S, Ndjeka N. Predictors of mortality and treatment success during treatment for rifampicin-resistant tuberculosis within the South African National TB Programme, 2009 to 2011: a cohort analysis of the national case register. Int J Infect Dis. 2015; 39: 89â€“94.	4 - Causes of Death
134231	Scientific literature	Prevalence of vaginitis, syphilis and HIV infection in women in the Orange Free State	Cronje HS, Joubert G, Muir A, Chapman RD, Divall P, Bam RH. Prevalence of vaginitis, syphilis and HIV infection in women in the Orange Free State. S Afr Med J. 1994; 84(9): 602-5.	4 - Causes of Death
128454	Scientific literature	Primaquine-sensitivity of red cells in various races in Southern Africa	Charlton RW, Bothwell TH. Primaquine-sensitivity of red cells in various races in Southern Africa. BMJ. 1961; 1(5230): 941-4.	4 - Causes of Death
214510	Scientific literature	Prognosis of patients with HIV-1 infection starting antiretroviral therapy in sub-Saharan Africa: a collaborative analysis of scale-up programmes	May M, Boulle A, Phiri S, Messou E, Myer L, Wood R, Keiser O, Sterne JAC, Dabis F, Egger M, IeDEA Southern Africa and West Africa. Prognosis of patients with HIV-1 infection starting antiretroviral therapy in sub-Saharan Africa: a collaborative analysis of scale-up programmes. Lancet. 2010; 376(9739): 449-57.	4 - Causes of Death
93598	Scientific literature	Rates and causes of child mortality in an area of high HIV prevalence in rural South Africa	Garrib A, Jaffar S, Knight S, Bradshaw D, Bennish ML. Rates and causes of child mortality in an area of high HIV prevalence in rural South Africa. Trop Med Int Health. 2006; 11(12): 1841-8.	4 - Causes of Death

214541	Scientific literature	Reducing mortality with cotrimoxazole preventive therapy at initiation of antiretroviral therapy in South Africa	Hoffmann CJ, Fielding KL, Charalambous S, Innes C, Chaisson RE, Grant AD, Churchyard GJ. Reducing mortality with cotrimoxazole preventive therapy at initiation of antiretroviral therapy in South Africa. AIDS. 2010; 24(11): 1709-16.	4 - Causes of Death
422969	Scientific literature	Role of Streptococcus pneumoniae in hospitalization for acute community-acquired pneumonia associated with culture-confirmed Mycobacterium tuberculosis in children: a pneumococcal conjugate vaccine probe study	Moore DP, Klugman KP, Madhi SA. Role of Streptococcus pneumoniae in hospitalization for acute community-acquired pneumonia associated with culture-confirmed Mycobacterium tuberculosis in children: a pneumococcal conjugate vaccine probe study. Pediatr Infect Dis J. 2010; 29(12): 1099-04.	4 - Causes of Death
214532	Scientific literature	Scale-up of a decentralized HIV treatment programme in rural KwaZulu-Natal, South Africa: does rapid expansion affect patient outcomes?	Mutevedzi PC, Lessells RJ, Heller T, BÃ¤rnighausen T, Cooke GS, Newell M-L. Scale-up of a decentralized HIV treatment programme in rural KwaZulu-Natal, South Africa: does rapid expansion affect patient outcomes?. Bull World Health Organ. 2010; 88(8): 593-600.	4 - Causes of Death
218643	Scientific literature	Serological screening for sexually transmitted infections in pregnancy: is there any value in re-screening for HIV and syphilis at the time of delivery?	Qolohle DC, Hoosen AA, Moodley J, Smith AN, Mlisana KP. Serological screening for sexually transmitted infections in pregnancy: is there any value in re-screening for HIV and syphilis at the time of delivery?. Genitourin Med. 1995; 71(2): 65-7.	4 - Causes of Death
214517	Scientific literature	Seven-year experience of a primary care antiretroviral treatment programme in Khayelitsha, South Africa	Boulle A, Van Cutsem G, Hilderbrand K, Cragg C, Abrahams M, Mathee S, Ford N, Knight L, Osler M, Myers J, Goemaere E, Coetzee D, Maartens G. Seven-year experience of a primary care antiretroviral treatment programme in Khayelitsha, South Africa. AIDS. 2010; 24(4): 563-72.	4 - Causes of Death
143218	Scientific literature	Sexually transmitted diseases in South Africa	Pham-Kanter GB, Steinberg MH, Ballard RC. Sexually transmitted diseases in South Africa. Genitourin Med. 1996; 72(3): 160-71.	4 - Causes of Death
131283	Scientific literature	South African measles outbreak 2009-2010 as experienced by a paediatric hospital	Le Roux DM, le Roux SM, Nuttall JJ, Eley BS. South African measles outbreak 2009-2010 as experienced by a paediatric hospital. S Afr Med J. 2012; 102(9): 760-4.	4 - Causes of Death
408051	Scientific literature	Streptococcus pneumoniae Serotypes and Mortality in Adults and Adolescents in South Africa: Analysis of National Surveillance Data, 2003 - 2008	Cohen C, Naidoo N, Meiring S, de Gouveia L, von Mollendorf C, Walaza S, Naicker P, Madhi SA, Feldman C, Klugman KP, Dawood H, von Gottberg A, GERMS-SA. Streptococcus pneumoniae Serotypes and Mortality in Adults and Adolescents in South Africa: Analysis of National Surveillance Data, 2003 - 2008. PLoS One. 2015; 10(10): 1-19.	4 - Causes of Death
213399	Scientific literature	Survival from HIV-1 seroconversion in Southern Africa: a retrospective cohort study in nearly 2000 gold-miners over 10 years of follow-up	Glynn JR, Sonnenberg P, Nelson G, Bester A, Shearer S, Murray J. Survival from HIV-1 seroconversion in Southern Africa: a retrospective cohort study in nearly 2000 gold-miners over 10 years of follow-up. AIDS. 2007; 21(5): 625-32.	4 - Causes of Death
138327	Scientific literature	Syphilis in pregnancy--prevalence at different levels of health care in Durban	Devjee J, Moodley J, Singh M. Syphilis in pregnancy--prevalence at different levels of health care in Durban. S Afr Med J. 2006; 96(11): 1182-4.	4 - Causes of Death
143221	Scientific literature	Syphilis in pregnant patients and their offspring	Bam RH,Â CronjÃ© HS,Â Muir A,Â Griessel DJ,Â Hoek BB. Syphilis in pregnant patients and their offspring. Int J Gynaecol Obstet. 1994; 44(2): 113-8.	4 - Causes of Death
214901	Scientific literature	Temporal changes in programme outcomes among adult patients initiating antiretroviral therapy across South Africa, 2002-2007	Cornell M, Grimsrud A, Fairall L, Fox MP, van Cutsem G, Giddy J, Wood R, Prozesky H, Mohapi L, Graber C, Egger M, Boulle A, Myer L, International Epidemiologic Databases to Evaluate AIDS Southern Africa (IeDEA-SA) Collaboration. Temporal changes in programme outcomes among adult patients initiating antiretroviral therapy across South Africa, 2002-2007. AIDS. 2010; 24(14): 2263-70.	4 - Causes of Death
350587	Scientific literature	The dangers of rationing dialysis treatment: The dilemma facing a developing country	Moosa MR, Kidd M. The dangers of rationing dialysis treatment: The dilemma facing a developing country. Kidney Int. 2006; 70(6): 1107â€“14.	4 - Causes of Death
415048	Scientific literature	The effects of syphilis on the outcome of pregnancy in the native; a clinical and statistical survey	SACHS SB. The effects of syphilis on the outcome of pregnancy in the native; a clinical and statistical survey. S Afr Med J. 1951; 25(15): 255-9.	4 - Causes of Death
357887	Scientific literature	The Incremental Value of Repeated Induced Sputum and Gastric Aspirate Samples for the Diagnosis of Pulmonary Tuberculosis in Young Children With Acute Community-Acquired Pneumonia	Moore DP, Higdon MM, Hammitt LL, Prosperi C, DeLuca AN, Da Silva P, Baillie VL, Adrian PV, Mudau A, Deloria Knoll M, Feikin DR, Murdoch DR, Oâ€™Brien KL, Madhi SA. The Incremental Value of Repeated Induced Sputum and Gastric Aspirate Samples for the Diagnosis of Pulmonary Tuberculosis in Young Children With Acute Community-Acquired Pneumonia. Clin Infect Dis. 2017; 64(suppl-3): S309â€“16.	4 - Causes of Death
214554	Scientific literature	The interplay between CD4 cell count, viral load suppression and duration of	Brennan AT, Maskew M, Sanne I, Fox MP. The interplay between CD4 cell count, viral load suppression and duration of antiretroviral	4 - Causes of Death

		antiretroviral therapy on mortality in a resource-limited setting	therapy on mortality in a resource-limited setting. Trop Med Int Health. 2013; 18(5): 619-31.	
283258	Scientific literature	The relative roles of hepatitis B and C viruses in the etiology of hepatocellular carcinoma in southern African blacks	Kew MC, Yu MC, Kedda MA, Coppin A, Sarkin A, Hodkinson J. The relative roles of hepatitis B and C viruses in the etiology of hepatocellular carcinoma in southern African blacks. Gastroenterology. 1997; 112(1): 184-7.	4 - Causes of Death
422856	Scientific literature	Treatment Outcomes of Children With HIV Infection and Drug-resistant TB in Three Provinces in South Africa, 2005-2008	Hall EW, Morris SB, Moore BK, Erasmus L, Odendaal R, Menzies H, van der Walt M, Smith SE. Treatment Outcomes of Children With HIV Infection and Drug-resistant TB in Three Provinces in South Africa, 2005-2008. Pediatr Infect Dis J. 2017; 36(12): e322-e327.	4 - Causes of Death
267694	Scientific literature	Treatment outcomes of HIV-positive patients on first-line antiretroviral therapy in private versus public HIV clinics in Johannesburg, South Africa	Moyo F, Chasela C, Brennan AT, Ebrahim O, Sanne IM, Long L, Evans D. Treatment outcomes of HIV-positive patients on first-line antiretroviral therapy in private versus public HIV clinics in Johannesburg, South Africa. Clin Epidemiol. 2016; 8: 37â€“47.	4 - Causes of Death
141078	Scientific literature	TreatmentÂ ofÂ maternalÂ syphilisÂ inÂ ruralÂ South Africa: effect of multiple doses of benzathine penicillin on pregnancy loss	Myer L,Â Abdool Karim SS,Â Lombard C,Â Wilkinson D. TreatmentÂ ofÂ maternalÂ syphilisÂ inÂ ruralÂ South Africa: effect of multiple doses of benzathine penicillin on pregnancy loss. Trop Med Int Health. 2004; 9(11): 1216-21.	4 - Causes of Death
335001	Scientific literature	Twelve-year mortality in adults initiating antiretroviral therapy in South Africa	Cornell M, Johnson LF, Wood R, Tanser F, Fox MP, Prozesky H, Schomaker M, Egger M, Davies MA, Boulle A, International Epidemiology Databases to Evaluate AIDS-Southern Africa collaboration. Twelve-year mortality in adults initiating antiretroviral therapy in South Africa. J Int AIDS Soc. 2017; 20(1): 21902.	4 - Causes of Death
141714	Scientific literature	UnsustainabilityÂ of aÂ measlesÂ immunisationÂ campaign-- riseÂ inÂ measlesÂ incidenceÂ withinÂ 2Â yearsÂ of the campaign	Abdool Karim SS,Â Abdool Karim Q,Â Dilraj A,Â Chamane M. UnsustainabilityÂ of aÂ measlesÂ immunisationÂ campaign-- riseÂ inÂ measlesÂ incidenceÂ withinÂ 2Â yearsÂ of the campaign. S Afr Med J. 1993; 83(5): 322-3.	4 - Causes of Death
93627	Scientific literature	Validation and application of verbal autopsies in a rural area of South Africa	Kahn K, Tollman SM, Garenne M, Gear JS. Validation and application of verbal autopsies in a rural area of South Africa. Trop Med Int Health. 2000; 5(11): 824-31.	4 - Causes of Death
274704	Scientific literature	Verbal autopsy-based cause-specific mortality trends in rural KwaZulu-Natal, South Africa, 2000-2009	Herbst AJ, Mafojane T, Newell M-L. Verbal autopsy-based cause-specific mortality trends in rural KwaZulu-Natal, South Africa, 2000-2009. Popul Health Metr. 2011; 9: 47.	4 - Causes of Death
214542	Scientific literature	Virologic, immunologic, and clinical response to highly active antiretroviral therapy: the gender issue revisited	Moore AL, Kirk O, Johnson AM, Katlama C, Blaxhult A, Dietrich M, Colebunders R, Chiesi A, Lungren JD, Phillips AN, EuroSIDA group. Virologic, immunologic, and clinical response to highly active antiretroviral therapy: the gender issue revisited. J Acquir Immune Defic Syndr. 2003; 32(4): 452-61.	4 - Causes of Death
141162	Scientific literature	Whooping cough â€“ aÂ neglected diseaseÂ inÂ southern Africa	Ramkissoon A,Â Coovadia HM,Â Loening WE. Whooping cough â€“ aÂ neglected diseaseÂ inÂ southern Africa. S Afr Med J. 1989; 75(12): 560-1.	4 - Causes of Death
270670	Survey	Global Atlas of Helminth Infections - Soil Transmitted Helminths	London School of Hygiene and Tropical Medicine. Global Atlas of Helminth Infections - Soil Transmitted Helminths. London, United Kingdom: London School of Hygiene and Tropical Medicine.	4 - Causes of Death
143217	Survey	South Africa - Eastern Cape HIV and Syphilis Antenatal Sero-Surveillance Survey 2007	Department of Health (South Africa), Eastern Cape Department of Health (South Africa). South Africa - Eastern Cape HIV and Syphilis Antenatal Sero-Surveillance Survey 2007.	4 - Causes of Death
20796	Survey	South Africa Demographic and Health Survey 1998	Department of Health (South Africa), Macro International, Inc, South African Medical Research Council. South Africa Demographic and Health Survey 1998. Fairfax, United States of America: ICF International.	4 - Causes of Death
157064	Survey	South Africa Demographic and Health Survey 2016	Department of Health (South Africa), ICF International, South African Medical Research Council, Statistics South Africa. South Africa Demographic and Health Survey 2016. Fairfax, United States of America: ICF International, 2019.	4 - Causes of Death
143233	Survey	South Africa National Antenatal Sentinel HIV and Syphilis Prevalence Survey 2000	Department of Health (South Africa). South Africa National Antenatal Sentinel HIV and Syphilis Prevalence Survey 2000.	4 - Causes of Death
356401	Survey	South Africa National Antenatal Sentinel HIV and Syphilis Prevalence Survey 2008	Department of Health (South Africa). South Africa National Antenatal Sentinel HIV and Syphilis Prevalence Survey 2008.	4 - Causes of Death
150187	Survey	South Africa National HIV and Syphillis Antenatal Sero-Prevalence Survey 2002	Department of Health (South Africa). South Africa National HIV and Syphillis Antenatal Sero-Prevalence Survey 2002.	4 - Causes of Death
313076	Survey	South Africa National HIV Prevalence, Incidence, and Behavior Survey 2011-2012	Centers for Disease Control and Prevention (CDC), Global Clinical and Viral Laboratory (South Africa), Human Sciences Research Council, National Institute for Communicable Diseases (South Africa), South African Medical Research Council, University of	4 - Causes of Death

			Cape Town. South Africa National HIV Prevalence, Incidence, and Behavior Survey 2011-2012. Pretoria, South Africa: Human Sciences Research Council, 2016.	
313074	Survey	South Africa National HIV Prevalence, Incidence, Behavior and Communication Survey 2004-2005	Center for AIDS Development, Research and Evaluation (CADRE) (South Africa), Centers for Disease Control and Prevention (CDC), Global Clinical and Viral Laboratory (South Africa), Human Sciences Research Council, Maphume Research Services, National Institute for Communicable Diseases (South Africa). South Africa National HIV Prevalence, Incidence, Behavior and Communication Survey 2004-2005. Pretoria, South Africa: Human Sciences Research Council, 2011.	4 - Causes of Death
357200	Survey	South Africa National HIV Prevalence, Incidence, Behavior and Communication Survey 2017	Centers for Disease Control and Prevention (CDC), Human Sciences Research Council, National Institute for Communicable Diseases (South Africa), South African Medical Research Council. South Africa National HIV Prevalence, Incidence, Behavior and Communication Survey 2017.	4 - Causes of Death
292791	Survey	WHO Global Project on Anti-Tuberculosis Drug Resistance Surveillance Data 1988-2015	World Health Organization (WHO). WHO Global Project on Anti-Tuberculosis Drug Resistance Surveillance Data 1988-2015.	4 - Causes of Death
106599	Vital registration	South Africa Saving Mothers 2008-2010	National Committee on Confidential Enquiries into Maternal Deaths (South Africa). South Africa Saving Mothers 2008-2010. Pretoria, South Africa: Department of Health (South Africa), 2012.	4 - Causes of Death
215184	Vital registration	South Africa Saving Mothers 2011-2013	National Committee on Confidential Enquiries into Maternal Deaths (South Africa). South Africa Saving Mothers 2011-2013. Pretoria, South Africa: Department of Health (South Africa), 2014.	4 - Causes of Death
107077	Vital registration	South Africa Vital Registration - Causes of Death 1997-2005	Department of Home Affairs (South Africa), Statistics South Africa. South Africa Vital Registration - Causes of Death 1997-2005. Pretoria, South Africa: Statistics South Africa.	4 - Causes of Death
151816	Vital registration	South Africa Vital Registration - Causes of Death 2006	Department of Home Affairs (South Africa), Statistics South Africa. South Africa Vital Registration - Causes of Death 2006. Pretoria, South Africa: Statistics South Africa.	4 - Causes of Death
151817	Vital registration	South Africa Vital Registration - Causes of Death 2007	Department of Home Affairs (South Africa), Statistics South Africa. South Africa Vital Registration - Causes of Death 2007. Pretoria, South Africa: Statistics South Africa.	4 - Causes of Death
106583	Vital registration	South Africa Vital Registration - Causes of Death 2008	Department of Home Affairs (South Africa), Statistics South Africa. South Africa Vital Registration - Causes of Death 2008. Pretoria, South Africa: Statistics South Africa.	4 - Causes of Death
106584	Vital registration	South Africa Vital Registration - Causes of Death 2009	Department of Home Affairs (South Africa), Statistics South Africa. South Africa Vital Registration - Causes of Death 2009. Pretoria, South Africa: Statistics South Africa.	4 - Causes of Death
151818	Vital registration	South Africa Vital Registration - Causes of Death 2010	Department of Home Affairs (South Africa), Statistics South Africa. South Africa Vital Registration - Causes of Death 2010. Pretoria, South Africa: Statistics South Africa.	4 - Causes of Death
151819	Vital registration	South Africa Vital Registration - Causes of Death 2011	Department of Home Affairs (South Africa), Statistics South Africa. South Africa Vital Registration - Causes of Death 2011. Pretoria, South Africa: Statistics South Africa.	4 - Causes of Death
204016	Vital registration	South Africa Vital Registration - Causes of Death 2012	Department of Home Affairs (South Africa), Statistics South Africa. South Africa Vital Registration - Causes of Death 2012. Pretoria, South Africa: Statistics South Africa.	4 - Causes of Death
204017	Vital registration	South Africa Vital Registration - Causes of Death 2013	Department of Home Affairs (South Africa), Statistics South Africa. South Africa Vital Registration - Causes of Death 2013. Pretoria, South Africa: Statistics South Africa.	4 - Causes of Death
267740	Vital registration	South Africa Vital Registration - Causes of Death 2014	Department of Home Affairs (South Africa), Statistics South Africa. South Africa Vital Registration - Causes of Death 2014. Pretoria, South Africa: Statistics South Africa.	4 - Causes of Death
312271	Vital registration	South Africa Vital Registration - Causes of Death 2015	Department of Home Affairs (South Africa), Statistics South Africa. South Africa Vital Registration - Causes of Death 2015. Pretoria, South Africa: Statistics South Africa.	4 - Causes of Death
399147	Vital registration	South Africa Vital Registration - Causes of Death 2016	Department of Home Affairs (South Africa), Statistics South Africa. South Africa Vital Registration - Causes of Death 2016. Pretoria, South Africa: Statistics South Africa, 2019.	4 - Causes of Death
312214	Administrative data	Insecticide Treated Net Procurement Data, Personal Communication with the World Health Organization 2016	World Health Organization (WHO). Insecticide Treated Net Procurement Data, Personal Communication with the World Health Organization 2016.	5 - Nonfatal Health Outcomes
354827	Administrative data	Insecticide Treated Net Procurement Data, Personal Communication with the World Health Organization 2017	World Health Organization (WHO). Insecticide Treated Net Procurement Data, Personal Communication with the World Health Organization 2017.	5 - Nonfatal Health Outcomes
425630	Administrative	Insecticide Treated Nets	World Health Organization (WHO). Insecticide Treated Nets	5 - Nonfatal

	data	Manufactured by Country, Personal Communication with the World Health Organization 2019	Manufactured by Country, Personal Communication with the World Health Organization 2019.	Health Outcomes
425631	Administrative data	National Malaria Control Program Insecticide Treated Nets Data, Personal Communication with the World Health Organization 2019	World Health Organization (WHO). National Malaria Control Program Insecticide Treated Nets Data, Personal Communication with the World Health Organization 2019.	5 - Nonfatal Health Outcomes
419891	Administrative data	WHO Distribution of Measles Cases by Country and by Month 2011-2020	World Health Organization (WHO). WHO Distribution of Measles Cases by Country and by Month 2011-2020.	5 - Nonfatal Health Outcomes
43152	Census	South Africa Census 2001 - IPUMS	Statistics South Africa, Minnesota Population Center. South Africa Census 2001 from the Integrated Public Use Microdata Series, International: [Machine-readable database]. Minneapolis: University of Minnesota.	5 - Nonfatal Health Outcomes
43158	Census	South Africa Community Survey 2007 - IPUMS	Statistics South Africa, Minnesota Population Center. South Africa Community Survey 2007 from the Integrated Public Use Microdata Series, International: [Machine-readable database]. Minneapolis: University of Minnesota.	5 - Nonfatal Health Outcomes
394317	Demographic surveillance	Child Health and Mortality Prevention Surveillance Network Program	Centers for Disease Control and Prevention (CDC), Child Health and Mortality Prevention Surveillance (CHAMPS) Network, Deloitte Consulting LLP, Emory Global Health Institute (EGHI), International Association of National Public Health Institutes (IANPHI), Task Force for Global Health, Emory University. Child Health and Mortality Prevention Surveillance (CHAMPS) Network Program.	5 - Nonfatal Health Outcomes
93667	Demographic surveillance	Contributions of the Africa Centre Demographic Surveillance to the Community	Muhwava W. Contributions of the Africa Centre Demographic Surveillance to the Community. Umbiko. 2011; 12. 3-4.	5 - Nonfatal Health Outcomes
11781	Demographic surveillance	South Africa - Agincourt Health and Socio-Demographic Surveillance System	MRC/Wits Rural Public Health and Health Transitions Research Unit (Agincourt), INDEPTH. South Africa - Agincourt Health and Socio-Demographic Surveillance System.	5 - Nonfatal Health Outcomes
114558	Disease registry	Cancer in South Africa Full Report 2003	National Cancer Registry (South Africa). Cancer in South Africa Full Report 2003 . Johannesburg, South Africa: National Institute for Occupational Health (South Africa).	5 - Nonfatal Health Outcomes
114560	Disease registry	Cancer in South Africa Full Report 2004	National Cancer Registry (South Africa). Cancer in South Africa Full Report 2004. Johannesburg, South Africa: National Institute for Occupational Health (South Africa).	5 - Nonfatal Health Outcomes
264234	Disease registry	Cancer in South Africa Full Report 2011	National Cancer Registry (South Africa). Cancer in South Africa Full Report 2011. Johannesburg, South Africa: National Cancer Registry (South Africa).	5 - Nonfatal Health Outcomes
116086	Disease registry	Cancer Incidence in Five Continents Volume X Summary Database 2003-2007	Forman D, Bray F, Brewster DH, Gombe Mbalawa C, Kohler B, PiÃ±eros M, Steliarova-Foucher E, Swaminathan R and Ferlay J, eds (2013). Cancer Incidence in Five Continents, Vol. X Summary Database (electronic version). Lyon, IARC. http://ci5.iarc.fr	5 - Nonfatal Health Outcomes
318157	Disease registry	Cancer Incidence in Five Continents Volume XI 2008-2012	Bray F, Colombet M, Mery L, PiÃ±eros M, Znaor A, Zanetti R and Ferlay J, editors (2017). Cancer Incidence in Five Continents, Vol. XI (electronic version). Lyon: International Agency for Research on Cancer. Available from: http://ci5.iarc.fr	5 - Nonfatal Health Outcomes
199034	Disease registry	South Africa - PROMEC Cancer Registry 2003-2007 - CI5	South Africa - PROMEC Cancer Registry 2003-2007 - CI5.	5 - Nonfatal Health Outcomes
113829	Disease registry	South Africa National Cancer Registry Incidence 2005	National Cancer Registry (South Africa). South Africa National Cancer Registry Incidence 2005. Johannesburg, South Africa: National Institute for Occupational Health (South Africa).	5 - Nonfatal Health Outcomes
128995	Disease registry	South Africa PROMEC Cancer Registry Incidence 1998-2002	PROMEC Unit, South African Medical Research Council. South Africa PROMEC Cancer Registry Incidence 1998-2002.	5 - Nonfatal Health Outcomes
160104	Disease registry	South African Birth Defects Surveillance Systems Data 1993-1997 - WHO	South African Birth Defects Surveillance Systems Data 1993-1997 - WHO	5 - Nonfatal Health Outcomes
160059	Disease registry	South African Birth Defects Surveillance Systems Data 2001 - ICBDMS	South African Birth Defects Surveillance Systems Data 2001 - ICBDMS	5 - Nonfatal Health Outcomes
268573	Disease registry	South African Birth Defects Surveillance Systems Data 2002 - ICBDMS	South African Birth Defects Surveillance Systems Data 2002 - ICBDMS	5 - Nonfatal Health Outcomes
160135	Disease registry	South African Birth Defects Surveillance Systems Data 2003 - ICBDSR	South African Birth Defects Surveillance Systems Data 2003 - ICBDSR	5 - Nonfatal Health Outcomes

243618	Disease registry	South African Renal Registry Annual Report 2012	South African Renal Society. South African Renal Registry Annual Report 2012. Durban, South Africa: South African Renal Society, 2014.	5 - Nonfatal Health Outcomes
243627	Disease registry	South African Renal Registry Annual Report 2013	South African Renal Society. South African Renal Registry Annual Report 2013. Durban, South Africa: South African Renal Society, 2015.	5 - Nonfatal Health Outcomes
340570	Epi surveillance	Compendium of Short Reports on Selected Outbreaks in the WHO African Region, October 2017	World Health Organization Regional Office for Africa (WHO-AFRO). Compendium of Short Reports on Selected Outbreaks in the WHO African Region, October 2017. Brazaville, Congo: World Health Organization Regional Office for Africa (WHO-AFRO), 2017.	5 - Nonfatal Health Outcomes
130412	Epi surveillance	Fetal Alcohol Syndrome - South Africa, 2001	Centers for Disease Control and Prevention (CDC). Fetal Alcohol Syndrome - South Africa, 2001. Morb Mortal Wkly Rep. 2003; 52(28): 660-2.	5 - Nonfatal Health Outcomes
137638	Epi surveillance	Global leprosy situation, 2004 - Weekly Epidemiological Record 2005	World Health Organization (WHO). Global leprosy situation, 2004. Wkly Epidemiol Rec. 2005: 80(13): 118-24.	5 - Nonfatal Health Outcomes
137645	Epi surveillance	Global leprosy situation, 2005 - Weekly Epidemiological Record 2005	World Health Organization (WHO). Global leprosy situation, 2005. Wkly Epidemiol Rec. 2005: 80(34): 289-95.	5 - Nonfatal Health Outcomes
137665	Epi surveillance	Global leprosy situation, 2007 - Weekly Epidemiological Record 2007	World Health Organization (WHO). Global leprosy situation, 2007. Wkly Epidemiol Rec. 2007: 82(25): 225-32.	5 - Nonfatal Health Outcomes
137680	Epi surveillance	Global leprosy situation, beginning of 2008 - Weekly Epidemiological Record 2008	World Health Organization (WHO). Global leprosy situation, beginning of 2008. Wkly Epidemiol Rec. 2008: 83(33): 293-300.	5 - Nonfatal Health Outcomes
137697	Epi surveillance	Global leprosy: update on the 2012 situation - Weekly Epidemiological Record 2013	World Health Organization (WHO). Global leprosy: update on the 2012 situation. Wkly Epidemiol Rec. 2013: 88(35): 365-79.	5 - Nonfatal Health Outcomes
310754	Epi surveillance	Malaria Atlas Project Annual Parasite Incidence Database	Malaria Atlas Project. Malaria Atlas Project Annual Parasite Incidence Database.	5 - Nonfatal Health Outcomes
311041	Epi surveillance	Notifiable Medical Conditions Report for South Africa November 2009	Department of Health (South Africa). Notifiable Medical Conditions Report for South Africa November 2009.	5 - Nonfatal Health Outcomes
229843	Epi surveillance	South Africa GERMS-SA Typhoid and Paratyphoid Tabulations 2003	National Institute for Communicable Diseases (South Africa). South Africa GERMS-SA Typhoid and Paratyphoid Tabulations 2003.	5 - Nonfatal Health Outcomes
229844	Epi surveillance	South Africa GERMS-SA Typhoid and Paratyphoid Tabulations 2004	National Institute for Communicable Diseases (South Africa). South Africa GERMS-SA Typhoid and Paratyphoid Tabulations 2004.	5 - Nonfatal Health Outcomes
229845	Epi surveillance	South Africa GERMS-SA Typhoid and Paratyphoid Tabulations 2005	National Institute for Communicable Diseases (South Africa). South Africa GERMS-SA Typhoid and Paratyphoid Tabulations 2005.	5 - Nonfatal Health Outcomes
229846	Epi surveillance	South Africa GERMS-SA Typhoid and Paratyphoid Tabulations 2006	National Institute for Communicable Diseases (South Africa). South Africa GERMS-SA Typhoid and Paratyphoid Tabulations 2006.	5 - Nonfatal Health Outcomes
229847	Epi surveillance	South Africa GERMS-SA Typhoid and Paratyphoid Tabulations 2007	National Institute for Communicable Diseases (South Africa). South Africa GERMS-SA Typhoid and Paratyphoid Tabulations 2007.	5 - Nonfatal Health Outcomes
229848	Epi surveillance	South Africa GERMS-SA Typhoid and Paratyphoid Tabulations 2008	National Institute for Communicable Diseases (South Africa). South Africa GERMS-SA Typhoid and Paratyphoid Tabulations 2008.	5 - Nonfatal Health Outcomes
229849	Epi surveillance	South Africa GERMS-SA Typhoid and Paratyphoid Tabulations 2009	National Institute for Communicable Diseases (South Africa). South Africa GERMS-SA Typhoid and Paratyphoid Tabulations 2009.	5 - Nonfatal Health Outcomes
229850	Epi surveillance	South Africa GERMS-SA Typhoid and Paratyphoid Tabulations 2010	National Institute for Communicable Diseases (South Africa). South Africa GERMS-SA Typhoid and Paratyphoid Tabulations 2010.	5 - Nonfatal Health Outcomes
229851	Epi surveillance	South Africa GERMS-SA Typhoid and Paratyphoid Tabulations 2011	National Institute for Communicable Diseases (South Africa). South Africa GERMS-SA Typhoid and Paratyphoid Tabulations 2011.	5 - Nonfatal Health Outcomes
229852	Epi surveillance	South Africa GERMS-SA Typhoid and Paratyphoid Tabulations 2012	National Institute for Communicable Diseases (South Africa). South Africa GERMS-SA Typhoid and Paratyphoid Tabulations 2012.	5 - Nonfatal Health Outcomes
229853	Epi surveillance	South Africa GERMS-SA Typhoid and Paratyphoid Tabulations 2013	National Institute for Communicable Diseases (South Africa). South Africa GERMS-SA Typhoid and Paratyphoid Tabulations 2013.	5 - Nonfatal Health Outcomes

229854	Epi surveillance	South Africa GERMS-SA Typhoid and Paratyphoid Tabulations 2014	National Institute for Communicable Diseases (South Africa). South Africa GERMS-SA Typhoid and Paratyphoid Tabulations 2014.	5 - Nonfatal Health Outcomes
340584	Epi surveillance	Weekly Bulletins on Outbreaks and Other Emergencies 2017	World Health Organization Regional Office for Africa (WHO-AFRO). Weekly Bulletins on Outbreaks and Other Emergencies 2017. Brazaville, Congo: World Health Organization Regional Office for Africa (WHO-AFRO), 2017.	5 - Nonfatal Health Outcomes
340865	Epi surveillance	Weekly Bulletins on Outbreaks and Other Emergencies 2018	World Health Organization Regional Office for Africa (WHO-AFRO). Weekly Bulletins on Outbreaks and Other Emergencies 2018. Brazaville , Congo: World Health Organization Regional Office for Africa (WHO-AFRO), 2018.	5 - Nonfatal Health Outcomes
83133	Epi surveillance	WHO and UNICEF Reported Disease Incidence Time Series	United Nations Children's Fund (UNICEF), World Health Organization (WHO). WHO and UNICEF Reported Disease Incidence Time Series. Geneva, Switzerland: World Health Organization (WHO).	5 - Nonfatal Health Outcomes
138604	Epi surveillance	WHO Global Health Observatory - Cholera: Number of Reported Cases by Country	World Health Organization (WHO). WHO Global Health Observatory - Cholera: Number of Reported Cases by Country. Geneva, Switzerland: World Health Organization (WHO).	5 - Nonfatal Health Outcomes
126384	Epi surveillance	WHO Tuberculosis Case Notifications	World Health Organization (WHO). WHO Tuberculosis Case Notifications. Geneva, Switzerland: World Health Organization (WHO).	5 - Nonfatal Health Outcomes
369109	Estimate	Global Burden of Disease Study 2017 (GBD 2017) Cause-Specific Mortality 1980-2017	Global Burden of Disease Collaborative Network. Global Burden of Disease Study 2017 (GBD 2017) Cause-Specific Mortality 1980-2017. Seattle, United States: Institute for Health Metrics and Evaluation (IHME), 2018.	5 - Nonfatal Health Outcomes
96480	Estimate	Sickle Cell and Thalassemias Prevalence Data, Personal Correspondence with David Weatherall	Weatherall D. Sickle Cell and Thalassemias Prevalence Data, Personal Correspondence with David Weatherall. [Unpublished].	5 - Nonfatal Health Outcomes
136038	Estimate	WHO Global Database on Child Growth and Malnutrition	World Health Organization (WHO). WHO Global Database on Child Growth and Malnutrition. Geneva, Switzerland: World Health Organization (WHO).	5 - Nonfatal Health Outcomes
136039	Estimate	WHO Global Database on Child Growth and Malnutrition - Historical	World Health Organization (WHO). WHO Global Database on Child Growth and Malnutrition - Historical. Geneva, Switzerland: World Health Organization (WHO).	5 - Nonfatal Health Outcomes
143198	Report	HIV/AIDS Epidemiological Surveillance Update for the WHO African Region 2002	World Health Organization Regional Office for Africa (WHO-AFRO). HIV/AIDS Epidemiological Surveillance Update for the WHO African Region 2002. Brazaville , Congo: World Health Organization Regional Office for Africa (WHO-AFRO), 2003.	5 - Nonfatal Health Outcomes
138349	Report	Missed opportunities for treating sexually transmitted infections at a rural primary health care setting in South Africa	Frohlich J, Abdool Karim Q, Abdool Karim S. Missed opportunities for treating sexually transmitted infections at a rural primary health care setting in South Africa. In: South African AIDS Conferece; 3-6 August 2003; Durban, South Africa.	5 - Nonfatal Health Outcomes
157571	Report	Plasmodium Falciparum Infection in Children Aged 2-15 Years	South African Medical Research Council Health GIS Centre. Plasmodium Falciparum Infection in Children Aged 2-15 Years. Cape Town, South Africa: South African Medical Research Council, 2000.	5 - Nonfatal Health Outcomes
311039	Report	Prevalence and Distribution of Malaria in South Africa - Part 1	Department of Health (South Africa). Prevalence and Distribution of Malaria in South Africa - Part 1. Pretoria, South Africa: Department of Health (South Africa), 1995.	5 - Nonfatal Health Outcomes
299749	Report	South Africa District Health Barometer 2015-2016	Health Systems Trust. South Africa District Health Barometer 2015-2016. Durban, South Africa: Health Systems Trust, 2016.	5 - Nonfatal Health Outcomes
157558	Report	South Africa Malaria Control Programme Monthly Report: Period 01-05-95 to 31-05-95	Department of Health (South Africa). South Africa Malaria Control Programme Monthly Report: Period 01-05-95 to 31-05-95. Jozini, South Africa: Department of Health (South Africa), 1995.	5 - Nonfatal Health Outcomes
311040	Report	South Africa Millennium Development Goals 6: Combat HIV/AIDS, Malaria and Other Diseases 2015	Statistics South Africa. South Africa Millennium Development Goals 6: Combat HIV/AIDS, Malaria and Other Diseases 2015. Pretoria, South Africa: Statistics South Africa, 2015.	5 - Nonfatal Health Outcomes
356498	Report	South Africa National Malaria Programme Performance Review 2009	Department of Health (South Africa). South Africa National Malaria Programme Performance Review 2009. 2009.	5 - Nonfatal Health Outcomes
138343	Report	Trends in the prevalence of sexually transmitted infections and HIV in pregnant women in KwaZulu/Natal from 1995 to 2002	Sturm A, Moodley P, Sturm P, Karim F, Khan N. Trends in the prevalence of sexually transmitted infections and HIV in pregnant women in KwaZulu/Natal from 1995-2002. In: South African AIDS Conference; 3-6 August 2003; Durban, South Africa.	5 - Nonfatal Health Outcomes
342867	Report	WHO International Travel and Health, Annex 1, Countries With Risk of	World Health Organization (WHO). WHO International Travel and Health, Annex 1, Countries With Risk of Yellow Fever Transmission	5 - Nonfatal Health

		Yellow Fever Transmission and Countries Requiring Yellow Fever Vaccination 2017 Update	and Countries Requiring Yellow Fever Vaccination 2017 Update. Geneva, Switzerland: World Health Organization (WHO), 2017.	Outcomes
124685	Report	WHO World Mental Health Surveys: Global Perspectives on the Epidemiology of Mental Disorders	World Health Organization (WHO). WHO World Mental Health Surveys: Global Perspectives on the Epidemiology of Mental Disorders. Cambridge, United Kingdom: Cambridge University Press, 2008.	5 - Nonfatal Health Outcomes
264188	Report	World Malaria Report 2015	World Health Organization (WHO). World Malaria Report 2015. Geneva, Switzerland: World Health Organization (WHO), 2015.	5 - Nonfatal Health Outcomes
284569	Report	World Malaria Report 2016	World Malaria Report 2016. Geneva: World Health Organization; 2016. Licence: CC BY-NC-SA 3.0 IGO.	5 - Nonfatal Health Outcomes
343613	Report	World Malaria Report 2017	World Malaria Report 2017. Geneva: World Health Organization; 2017. Licence: CC BY-NC-SA 3.0 IGO.	5 - Nonfatal Health Outcomes
116219	Scientific literature	Accuracy of serological testing for the diagnosis of prevalent neurocysticercosis in outpatients with epilepsy, Eastern Cape Province, South Africa	Foyaca-Sibat H, Cowan LD, Carabin H, Targonska I, Anwary MA, Serrano-Oca-a G, Krecek RC, Willingham AL 3rd. Accuracy of serological testing for the diagnosis of prevalent neurocysticercosis in outpatients with epilepsy, Eastern Cape Province, South Africa. PLoS Negl Trop Dis. 2009; 3(12): e562.	5 - Nonfatal Health Outcomes
214569	Scientific literature	Adherence to highly active antiretroviral therapy assessed by pharmacy claims predicts survival in HIV-infected South African adults	Nachega JB, Hislop M, Dowdy DW, Lo M, Omer SB, Regensberg L, Chaisson RE, Maartens G. Adherence to highly active antiretroviral therapy assessed by pharmacy claims predicts survival in HIV-infected South African adults. J Acquir Immune Defic Syndr. 2006; 43(1): 78-84.	5 - Nonfatal Health Outcomes
94619	Scientific literature	Aetiological factors of infantile diarrhoea: a community-based study	Loening WE, Coovadia YM, Van den Ende J. Aetiological factors of infantile diarrhoea: a community-based study. Ann Trop Paediatr. 1989; 9(4): 248-55.	5 - Nonfatal Health Outcomes
229739	Scientific literature	Age differences in the prevalence and co-morbidity of DSM-IV major depressive episodes: results from the WHO World Mental Health Survey Initiative	Kessler RC, Birnbaum HG, Shahly V, Bromet E, Hwang I, McLaughlin KA, Sampson N, Andrade LH, de Girolamo G, Demyttenaere K, Haro JM, Karam AN, Kostyuchenko S, Kovess V, Lara C, Levinson D, Matschinger H, Nakane Y, Browne MO, Ormel J, Posada-Villa J, Sagar R, Stein DJ. Age differences in the prevalence and co-morbidity of DSM-IV major depressive episodes: results from the WHO World Mental Health Survey Initiative. Depress Anxiety. 2010; 27(4): 351â€“64.	5 - Nonfatal Health Outcomes
267580	Scientific literature	Age in antiretroviral therapy programmes in South Africa: a retrospective, multicentre, observational cohort study	Cornell M, Johnson LF, Schomaker M, Tanser F, Maskew M, Wood R, Prozesky H, Giddy J, Stinson K, Egger M, Boulle A, Myer L, International Epidemiologic Databases to Evaluate AIDS-Southern Africa Collaboration. Age in antiretroviral therapy programmes in South Africa: a retrospective, multicentre, observational cohort study. Lancet HIV. 2015; 2(9): e368-375.	5 - Nonfatal Health Outcomes
335046	Scientific literature	All-cause mortality in HIV-positive adults starting combination antiretroviral therapy: correcting for loss to follow-up	Anderegg N, Johnson LF, Zaniewski E, Althoff KN, Balestre E, Law M, Nash D, Shepherd BE, Yiannoutsos CT, Egger M, IeDEA, MeSH consortia. All-cause mortality in HIV-positive adults starting combination antiretroviral therapy: correcting for loss to follow-up. AIDS. 2017; 31 Suppl 1: S31â€“40.	5 - Nonfatal Health Outcomes
317971	Scientific literature	An association between decreasing incidence of invasive non-typhoidal salmonellosis and increased use of antiretroviral therapy, Gauteng Province, South Africa, 2003â€“2013. Vermund SH, editor	Keddy KH, Takuva S, Musekiwa A, Puren AJ, Sooka A, Karstaedt A, Klugman KP, Angulo FJ. An association between decreasing incidence of invasive non-typhoidal salmonellosis and increased use of antiretroviral therapy, Gauteng Province, South Africa, 2003â€“2013. Vermund SH, editor. PLoS One. 2017; 12(3): e0173091.	5 - Nonfatal Health Outcomes
413889	Scientific literature	An audit of the management of ectopic pregnancies in a district hospital, Gauteng, South Africa	Nzaumvila DK, Govender I, Ogunbanjo GA. An audit of the management of ectopic pregnancies in a district hospital, Gauteng, South Africa. Afr J Prim Health Care Fam Med. 2018; 10(1): e1-e8.	5 - Nonfatal Health Outcomes
416139	Scientific literature	An isolated outbreak of diphtheria in South Africa, 2015	Mahomed S, Archary M, Mutevedzi P, Mahabeer Y, Govender P, Ntshoe G, Kuhn W, Thomas J, Olowolagba A, Blumberg L, McCarthy K, Mlisana K, DU Plessis M, VON Gottberg A, Moodley P. An isolated outbreak of diphtheria in South Africa, 2015. Epidemiol Infect. 2017; 145(10): 2100-2108.	5 - Nonfatal Health Outcomes
255129	Scientific literature	Anaemia among the inhabitants of a rural area in northern Natal	Mayet FGH, Schutte CHJ, Reinach SG. Anaemia among the inhabitants of a rural area in northern Natal. S Afr Med J. 1985; 67(12): 458-62.	5 - Nonfatal Health Outcomes
209344	Scientific literature	Antiretroviral Treatment Outcomes amongst Older Adults in a Large Multicentre Cohort in South Africa	Fatti G, Mothibi E, Meintjes G, Grimwood A. Antiretroviral Treatment Outcomes amongst Older Adults in a Large Multicentre Cohort in South Africa. PLoS One. 2014; 9(6): e100273.	5 - Nonfatal Health Outcomes

229509	Scientific literature	Approaching the prevalence of the full spectrum of fetal alcohol spectrum disorders in a South African population-based study	May PA, Blankenship J, Marais AS, Gossage JP, Kalberg WO, Barnard R, De Vries M, Robinson LK, Adnams CM, Buckley D, Manning M, Jones KL, Parry C, Hoyme HE, Seedat S. Approaching the prevalence of the full spectrum of fetal alcohol spectrum disorders in a South African population-based study. Alcohol Clin Exp Res. 2013; 37(5): 818â€“30.	5 - Nonfatal Health Outcomes
115557	Scientific literature	Association between children's household living conditions and eczema in the Polokwane area, South Africa	Wichmann J, Wolvaardt JE, Maritz C, Voyi KVV. Association between children's household living conditions and eczema in the Polokwane area, South Africa. Health Place. 2008; 14(2): 323-35.	5 - Nonfatal Health Outcomes
279074	Scientific literature	Association Between Passive Smoking and Infection With Mycobacterium tuberculosis in Children	den Boon S, Verver S, Marais BJ, Enarson DA, Lombard CJ, Bateman ED, Irusen E, Jithoo A, Gie RP, Borgdorff MW, Beyers N. Association Between Passive Smoking and Infection With Mycobacterium tuberculosis in Children. Pediatrics. 2007; 119(4): 734-9.	5 - Nonfatal Health Outcomes
222197	Scientific literature	Association of low-risk human papillomavirus infection with male circumcision in young men: results from a longitudinal study conducted in Orange Farm (South Africa)	Tarnaud C, Lissouba P, Cutler E, Puren A, Taljaard D, Auvert B. Association of low-risk human papillomavirus infection with male circumcision in young men: results from a longitudinal study conducted in Orange Farm (South Africa). Infect Dis Obstet Gynecol. 2011; 567408.	5 - Nonfatal Health Outcomes
221944	Scientific literature	Association of oncogenic and nononcogenic human papillomavirus with HIV incidence	Auvert B, Lissouba P, Cutler E, Zarca K, Puren A, Taljaard D. Association of oncogenic and nononcogenic human papillomavirus with HIV incidence. J Acquir Immune Defic Syndr. 2010; 53(1): 111â€“6.	5 - Nonfatal Health Outcomes
221898	Scientific literature	Associations between childhood adversity and depression, substance abuse and HIV and HSV2 incident infections in rural South African youth	Jewkes RK, Dunkle K, Nduna M, Jama PN, Puren A. Associations between childhood adversity and depression, substance abuse and HIV and HSV2 incident infections in rural South African youth. Child Abuse Negl. 2010; 34(11): 833â€“41.	5 - Nonfatal Health Outcomes
221727	Scientific literature	Associations between psychosocial factors and incidence of sexually transmitted disease among South African adolescents	Oâ€™Leary A, Jemmott JB, Jemmott LS, Teitelman A, Heeren GA, Ngwane Z, Icard LD, Lewis DA. Associations between psychosocial factors and incidence of sexually transmitted disease among South African adolescents. Sex Transm Dis. 2015; 42(3): 135â€“9.	5 - Nonfatal Health Outcomes
116695	Scientific literature	Atypical rotavirus identified from young children with diarrhoea in South Africa	Sebata T, Steele AD. Atypical rotavirus identified from young children with diarrhoea in South Africa. J Health Popul Nutr. 2001; 19(3): 199-203.	5 - Nonfatal Health Outcomes
139385	Scientific literature	Bacterial meningitis in children at Kalafong Hospital, 1990-1995	Grobler AC, Hay IT. Bacterial meningitis in children at Kalafong Hospital, 1990-1995. S Afr Med J. 1997; 87(8 Suppl): 1052-4.	5 - Nonfatal Health Outcomes
214505	Scientific literature	Better antiretroviral therapy outcomes at primary healthcare facilities: an evaluation of three tiers of ART services in four South African provinces	Fatti G, Grimwood A, Bock P. Better antiretroviral therapy outcomes at primary healthcare facilities: an evaluation of three tiers of ART services in four South African provinces. PLoS One. 2010; 5(9): e12888.	5 - Nonfatal Health Outcomes
128769	Scientific literature	Burden of end-stage renal disease in sub-Saharan Africa	Naicker S. Burden of end-stage renal disease in sub-Saharan Africa. Clin Nephrol. 2010; 74(Suppl 1): S13-6.	5 - Nonfatal Health Outcomes
229513	Scientific literature	Burden of fetal alcohol syndrome in a rural West Coast area of South Africa	Olivier L, Urban M, Chersich M, Temmerman M, Viljoen D. Burden of fetal alcohol syndrome in a rural West Coast area of South Africa. S Afr Med J. 2013; 103(6): 402â€“5.	5 - Nonfatal Health Outcomes
402198	Scientific literature	Cancer of childhood in sub-Saharan Africa	Stefan C, Bray F, Ferlay J, Liu B, Maxwell Parkin D. Cancer of childhood in sub-Saharan Africa. ecancer. 2017; 11: 755.	5 - Nonfatal Health Outcomes
351083	Scientific literature	Cardiac function in an African dialysis population with a low prevalence of pre-existing cardiovascular disease	Luyckx VA, Yip A, Sofianou L, Jhangri GS, Mueller TF, Naicker S. Cardiac function in an African dialysis population with a low prevalence of pre-existing cardiovascular disease. Ren Fail. 2009; 31(3): 211â€“20.	5 - Nonfatal Health Outcomes
214553	Scientific literature	CD4 count slope and mortality in HIV-infected patients on antiretroviral therapy: multicohort analysis from South Africa	Hoffmann CJ, Schomaker M, Fox MP, Mutevedzi P, Giddy J, Prozesky H, Wood R, Garone DB, Egger M, Boulle A, IeDEA Southern Africa Collaboration. CD4 count slope and mortality in HIV-infected patients on antiretroviral therapy: multicohort analysis from South Africa. J Acquir Immune Defic Syndr. 2013; 63(1): 34-41.	5 - Nonfatal Health Outcomes
116206	Scientific literature	Changes in malaria morbidity and mortality in Mpumalanga Province, South Africa (2001-2009): a retrospective study	Ngomane L, de Jager C. Changes in malaria morbidity and mortality in Mpumalanga Province, South Africa (2001-2009): a retrospective study. Malar J. 2012; 19.	5 - Nonfatal Health Outcomes
214519	Scientific literature	Changes in programmatic outcomes during 7 years of scale-up at a community-based antiretroviral	Nglazi MD, Lawn SD, Kaplan R, Kranzer K, Orrell C, Wood R, Bekker L-G. Changes in programmatic outcomes during 7 years of scale-up at a community-based antiretroviral treatment service in	5 - Nonfatal Health Outcomes

		treatment service in South Africa	South Africa. J Acquir Immune Defic Syndr. 2011; 56(1): e1-8.	
138325	Scientific literature	Changing patterns of knowledge, reported behaviour and sexually transmitted infections in a South African gold mining community	Williams BG, Taljaard D, Campbell CM, Gouws E, Ndhlovu L, Van Dam J, CaraÃ«l M, Auvert B. Changing patterns of knowledge, reported behaviour and sexually transmitted infections in a South African gold mining community. AIDS. 2003; 17(14): 2099-107.	5 - Nonfatal Health Outcomes
214535	Scientific literature	Changing predictors of mortality over time from cART start: implications for care	Hoffmann CJ, Fielding KL, Johnston V, Charalambous S, Innes C, Moore RD, Chaisson RE, Grant AD, Churchyard GJ. Changing predictors of mortality over time from cART start: implications for care. J Acquir Immune Defic Syndr. 2011; 58(3): 269-76.	5 - Nonfatal Health Outcomes
279361	Scientific literature	Changing prevalence of tuberculosis infection with increasing age in high-burden townships in South Africa	Wood R, Liang H, Wu H, Middelkoop K, Oni T, Rangaka MX, Wilkinson RJ, Bekker L-G, Lawn SD. Changing prevalence of tuberculosis infection with increasing age in high-burden townships in South Africa. Int J Tuberc Lung Dis. 2010; 14(4): 406â€“412.	5 - Nonfatal Health Outcomes
154525	Scientific literature	Children with intellectual disability in rural South Africa: prevalence and associated disability	Christianson AL, Zwane ME, Manga P, Rosen E, Venter A, Downs D, Kromberg JGR. Children with intellectual disability in rural South Africa: prevalence and associated disability. J Intellect Disabil Res. 2002; 46(Pt 2): 179-86.	5 - Nonfatal Health Outcomes
357021	Scientific literature	Choleraâ€™s western front	Harris JB, Larocque RC, Charles RC, Mazumder RN, Khan AI, Bardhan PK. Choleraâ€™s western front. Lancet. 2010; 376(9757): 1961â€“5.	5 - Nonfatal Health Outcomes
328196	Scientific literature	Chronic kidney diseases in mixed ancestry south African populations: prevalence, determinants and concordance between kidney function estimators	Matsha TE, Yako YY, Rensburg MA, Hassan MS, Kengne AP, Erasmus RT. Chronic kidney diseases in mixed ancestry south African populations: prevalence, determinants and concordance between kidney function estimators. BMC Nephrol. 2013; 14: 75.	5 - Nonfatal Health Outcomes
270759	Scientific literature	Collective efficacy, alcohol outlet density, and young menâ€™s alcohol use in rural South Africa	Leslie HH, Ahern J, Pettifor AE, Twine R, Kahn K, GÃ³mez-OlivÃ© FX, Lippman SA. Collective efficacy, alcohol outlet density, and young menâ€™s alcohol use in rural South Africa. Health Place. 2015; 34: 190â€“8.	5 - Nonfatal Health Outcomes
350104	Scientific literature	Common risk factors and edentulism in adults, aged 50 years and over, in China, Ghana, India and South Africa: results from the WHO Study on global AGEing and adult health (SAGE)	Kailembo A, Preet R, Stewart Williams J. Common risk factors and edentulism in adults, aged 50 years and over, in China, Ghana, India and South Africa: results from the WHO Study on global AGEing and adult health (SAGE). BMC Oral Health. 2016; 17(1): 29.	5 - Nonfatal Health Outcomes
221840	Scientific literature	Comparison of focus HerpesSelect and Kalon HSV-2 gG2 ELISA serological assays to detect herpes simplex virus type 2 antibodies in a South African population	Delany-Moretlwe S, Jentsch U, Weiss H, Moyes J, Ashley-Morrow R, Stevens W, Mayaud P. Comparison of focus HerpesSelect and Kalon HSV-2 gG2 ELISA serological assays to detect herpes simplex virus type 2 antibodies in a South African population. Sex Transm Infect. 2010; 86(1): 46â€“50.	5 - Nonfatal Health Outcomes
214556	Scientific literature	Comparison of tenofovir, zidovudine, or stavudine as part of first-line antiretroviral therapy in a resource-limited-setting: a cohort study	Velen K, Lewis JJ, Charalambous S, Grant AD, Churchyard GJ, Hoffmann CJ. Comparison of tenofovir, zidovudine, or stavudine as part of first-line antiretroviral therapy in a resource-limited-setting: a cohort study. PLoS One. 2013; 8(5): e64459.	5 - Nonfatal Health Outcomes
221722	Scientific literature	Cross-sectional study of genital, rectal, and pharyngeal Chlamydia and gonorrhea in women in rural South Africa	Peters RPH, Dubbink JH, van der Eem L, Verweij SP, Bos MLA, Ouburg S, Lewis DA, Struthers H, McIntyre JA, MorrÃ© SA. Cross-sectional study of genital, rectal, and pharyngeal Chlamydia and gonorrhea in women in rural South Africa. Sex Transm Dis. 2014; 41(9): 564â€“9.	5 - Nonfatal Health Outcomes
257166	Scientific literature	Cystic Echinococcosis Endemicity Estimates	Cystic Echinococcosis Endemicity Estimates identified through systematic review and personal communication, as provided by GBD 2015 expert. [Unpublished].	5 - Nonfatal Health Outcomes
273750	Scientific literature	Days out of role due to mental and physical illness in the South African stress and health study	Mall S, Lund C, Vilagut G, Alonso J, Williams DR, Stein DJ. Days out of role due to mental and physical illness in the South African stress and health study. Soc Psychiatry Psychiatr Epidemiol. 2015; 50: 461â€“8.	5 - Nonfatal Health Outcomes
279328	Scientific literature	Decreasing household contribution to TB transmission with age: a retrospective geographic analysis of young people in a South African township	Middelkoop K, Bekker L-G, Morrow C, Lee N, Wood R. Decreasing household contribution to TB transmission with age: a retrospective geographic analysis of young people in a South African township. BMC Infect Dis. 2014; 14(1): 1.	5 - Nonfatal Health Outcomes
221569	Scientific literature	Defining hypoxic ischemic encephalopathy in newborn infants: benchmarking in a South African population	Horn AR, Swingler GH, Myer L, Harrison MC, Linley LL, Nelson C, Tooke L, Rhoda NR, Robertson NJ. Defining hypoxic ischemic encephalopathy in newborn infants: benchmarking in a South African population. J Perinat Med. 2013; 41(2): 211-7.	5 - Nonfatal Health Outcomes
273000	Scientific literature	Delay and poor diagnosis of Down syndrome in KwaZulu-Natal, South Africa: A retrospective review of postnatal cytogenetic testing	Willoughby M, Aldous C, Patrick M, Kavonic S, Christianson A. Delay and poor diagnosis of Down syndrome in KwaZulu-Natal, South Africa: A retrospective review of postnatal cytogenetic testing. S Afr Med J. 2016; 106(6): 626-9.	5 - Nonfatal Health Outcomes

119180	Scientific literature	Dental caries in six, 12 and 15 year old Venda children in South Africa	Bajomo AS, Rudolph MJ, Ogunbodede EO. Dental caries in six, 12 and 15 year old Venda children in South Africa. East Afr Med J. 2004; 81(5): 236-43.	5 - Nonfatal Health Outcomes
119057	Scientific literature	Dental caries of 12- and 15-year-old schoolchildren in Gazankulu, South Africa	Chikte UM, Rudolph MJ, Smythe AE. Dental caries of 12- and 15-year-old schoolchildren in Gazankulu, South Africa. Community Dent Oral Epidemiol. 1991; 19(4): 237-8.	5 - Nonfatal Health Outcomes
271108	Scientific literature	Depression and associated factors in older adults in South Africa	Peltzer K, Phaswana-Mafuya N. Depression and associated factors in older adults in South Africa. Glob Health Action. 2013; 6: 1â€“9.	5 - Nonfatal Health Outcomes
271129	Scientific literature	Depression, disability and functional status among community-dwelling older adults in South Africa: evidence from the first South African National Income Dynamics Study	Tomita A, Burns JK. Depression, disability and functional status among community-dwelling older adults in South Africa: evidence from the first South African National Income Dynamics Study. Int J Geriatr Psychiatry. 2013; 28(12): 1270â€“9.	5 - Nonfatal Health Outcomes
127569	Scientific literature	Detection of herpes simplex virus type 2-specific immunoglobulin G antibodies in African sera by using recombinant gG2, Western blotting, and gG2 inhibition	Hogrefe W, Su X, Song J, Ashley R, Kong L. Detection of herpes simplex virus type 2-specific immunoglobulin G antibodies in African sera by using recombinant gG2, Western blotting, and gG2 inhibition. J Clin Microbiol. 2002; 40(10): 3635-40.	5 - Nonfatal Health Outcomes
221976	Scientific literature	Determinants of differential HIV incidence among women in three southern African locations	Mavedzenge SN, Weiss HA, Montgomery ET, Blanchard K, de Bruyn G, Ramjee G, Chipato T, Padian NS, Van Der Straten A. Determinants of differential HIV incidence among women in three southern African locations. J Acquir Immune Defic Syndr. 2011; 58(1): 89â€“99.	5 - Nonfatal Health Outcomes
112861	Scientific literature	Diabetes and other disorders of glycemia in a rural South African community: prevalence and associated risk factors	Motala AA, Esterhuizen T, Gouws E, Pirie FJ, Omar MA. Diabetes and other disorders of glycemia in a rural South African community: prevalence and associated risk factors. Diabetes Care. 2008; 31(9): 1783-8.	5 - Nonfatal Health Outcomes
112294	Scientific literature	Diabetes in rural South Africa â€“ an assessment of care and complications	Rotchford AP, Rotchford KM. Diabetes in rural South Africa â€“ an assessment of care and complications. S Afr Med J. 2002; 92(7): 536-41.	5 - Nonfatal Health Outcomes
139594	Scientific literature	Dietary iodine deficiency in South Africa. Surveys before the introduction of universal salt iodisation	Kalk WJ, Paiker J, van Arb MG, Pick W. Dietary iodine deficiency in South Africa. Surveys before the introduction of universal salt iodisation. S Afr Med J. 1998; 88(3 Endocrinology): 357-8.	5 - Nonfatal Health Outcomes
293926	Scientific literature	Dietary iron overload as a risk factor for hepatocellular carcinoma in Black Africans	Mandishona E, MacPhail AP, Gordeuk VR, Kedda M-A, Paterson AC, Rouault TA, Kew MC. Dietary iron overload as a risk factor for hepatocellular carcinoma in Black Africans . Hepatology. 1998; 27(6): 1563-6.	5 - Nonfatal Health Outcomes
269652	Scientific literature	Does migration affect asthma, rhinoconjunctivitis and eczema prevalence? Global findings from the international study of asthma and allergies in childhood	Garcia-Marcos L, Robertson CF, Ross Anderson H, Ellwood P, Williams HC, Wong GW. Does migration affect asthma, rhinoconjunctivitis and eczema prevalence? Global findings from the international study of asthma and allergies in childhood. Int J Epidemiol. 2014; 43(6): 1846-54.	5 - Nonfatal Health Outcomes
103700	Scientific literature	Ear and hearing disorders in rural grade 2 (Sub B) schoolchildren in the western Cape	Prescott CA, Kibel MA. Ear and hearing disorders in rural grade 2 (Sub B) schoolchildren in the western Cape. S Afr Med J. 1991; 79(2): 90-3.	5 - Nonfatal Health Outcomes
214508	Scientific literature	Early loss of HIV-infected patients on potent antiretroviral therapy programmes in lower-income countries	Brinkhof MWG, Dabis F, Myer L, Bangsberg DR, Boulle A, Nash D, Schechter M, Laurent C, Keiser O, May M, Sprinz E, Egger M, Anglaret X, ART-LINC, IeDEA. Early loss of HIV-infected patients on potent antiretroviral therapy programmes in lower-income countries. Bull World Health Organ. 2008; 86(7): 559-67.	5 - Nonfatal Health Outcomes
222425	Scientific literature	Effect of HSV-2 serostatus on acquisition of HIV by young men: results of a longitudinal study in Orange Farm, South Africa	Sobngwi-Tambekou J, Taljaard D, Lissouba P, Zarca K, Puren A, Lagarde E, Auvert B. Effect of HSV-2 serostatus on acquisition of HIV by young men: results of a longitudinal study in Orange Farm, South Africa. J Infect Dis. 2009; 199(7): 958â€“64.	5 - Nonfatal Health Outcomes
116699	Scientific literature	Electrophoretic typing of nosocomial rotavirus infection in a general paediatric unit showing the continual introduction of community strains	Steele AD, Mnisi YN, Williams MM, Bos P, Aspinall S. Electrophoretic typing of nosocomial rotavirus infection in a general paediatric unit showing the continual introduction of community strains. J Med Virol. 1993; 40(2): 126-32.	5 - Nonfatal Health Outcomes
128762	Scientific literature	End-stage renal disease in sub-Saharan and South Africa	Naicker S. End-stage renal disease in sub-Saharan and South Africa. Kidney Int Suppl. 2003; 83: S119-22.	5 - Nonfatal Health Outcomes
139498	Scientific literature	Endemic goitre in a rural community of KwaZulu-Natal	BenedÃ© JG, Oelofse A, van Stuijvenberg ME, Jooste PL, Weight MJ, BenadÃ© AJ. Endemic goitre in a rural community of KwaZulu-Natal. S Afr Med J. 1997; 87(3): 310-3.	5 - Nonfatal Health Outcomes
270127	Scientific literature	Environmental tobacco smoke and the risk of eczema symptoms among school children in South Africa: a	Shirinde J, Wichmann J, Voyi K. Environmental tobacco smoke and the risk of eczema symptoms among school children in South Africa: a cross-sectional study. BMJ Open. 2015; 5(8): e008234.	5 - Nonfatal Health Outcomes

		cross-sectional study		
229254	Scientific literature	Epidemiology of fetal alcohol spectrum disorders in rural communities in South Africa; Prevalence, child characteristics, and maternal risk factors	Vries MM, Marais AS, WOK DB, Adnams CM, Hasken JM, Seedat S, Parry CDH, May PA. Epidemiology of fetal alcohol spectrum disorders in rural communities in South Africa; Prevalence, child characteristics, and maternal risk factors. Alcohol Clin Exp Res. 2014; 38(Suppl 1): 251a.	5 - Nonfatal Health Outcomes
229510	Scientific literature	Epidemiology of fetal alcohol syndrome in a South African community in the Western Cape Province	May PA, Brooke L, Gossage JP, Croxford J, Adnams C, Jones KL, Robinson L, Viljoen D. Epidemiology of fetal alcohol syndrome in a South African community in the Western Cape Province. Am J Public Health. 2000; 90(12): 1905â€“12.	5 - Nonfatal Health Outcomes
230971	Scientific literature	Epidemiology of influenza virus types and subtypes in South Africa, 2009-2012	Cohen AL, Hellferscee O, Pretorius M, Treurnicht F, Walaza S, Madhi S, Groome M, Dawood H, Variava E, Kahn K, Wolter N, von Gottberg A, Tempia S, Venter M, Cohen C. Epidemiology of influenza virus types and subtypes in South Africa, 2009-2012. Emerg Infect Dis. 2014; 20(7): 1162â€“9.	5 - Nonfatal Health Outcomes
311721	Scientific literature	Epidemiology of malaria in South Africa: from control to elimination.(Epidemiology)(Report)	Maharaj R, Raman J, Morris N, Moonasar D, Durrheim DN, Seocharan I, Kruger P, Shandukani B, Kleinschmidt I. Epidemiology of malaria in South Africa: from control to elimination.(Epidemiology)(Report). S Afr Med J. 2013; 103(10): 779â€“83.	5 - Nonfatal Health Outcomes
339649	Scientific literature	Epidemiology of respiratory syncytial virus-associated acute lower respiratory tract infection hospitalizations among HIV-infected and HIV-uninfected South African children, 2010-2011	Moyes J, Cohen C, Pretorius M, Groome M, von Gottberg A, Wolter N, Walaza S, Haffejee S, Chhagan M, Naby F, Cohen AL, Tempia S, Kahn K, Dawood H, Venter M, Madhi SA, South African Severe Acute Respiratory Illness Surveillance Group. Epidemiology of respiratory syncytial virus-associated acute lower respiratory tract infection hospitalizations among HIV-infected and HIV-uninfected South African children, 2010-2011. J Infect Dis. 2013; 208 Suppl 3: S217-226.	5 - Nonfatal Health Outcomes
233198	Scientific literature	Epidemiology of viral-associated acute lower respiratory tract infection among children <5 years of age in a high HIV prevalence setting, South Africa, 2009-2012	Cohen C, Walaza S, Moyes J, Groome M, Tempia S, Pretorius M, Hellferscee O, Dawood H, Chhagan M, Naby F, Haffejee S, Variava E, Kahn K, Nzenze S, Tshangela A, von Gottberg A, Wolter N, Cohen AL, Kgokong B, Venter M, Madhi SA. Epidemiology of viral-associated acute lower respiratory tract infection among children Pediatr Infect Dis J. 2015; 34(1): 66â€“72.	5 - Nonfatal Health Outcomes
143220	Scientific literature	Epidemiology ofÂ syphilisÂ inÂ pregnancyÂ in rural South Africa: opportunities for control	Wilkinson D,Â Sach M,Â Connolly C. Epidemiology ofÂ syphilisÂ inÂ pregnancyÂ in rural South Africa: opportunities for control. Trop Med Int Health. 1997; 2(1): 57-62.	5 - Nonfatal Health Outcomes
125378	Scientific literature	Epilepsy in rural South African children â€“ prevalence, associated disability and management	Christianson AL, Zwane ME, Manga P, Rosen E, Venter A, Kromberg JG. Epilepsy in rural South African children â€“ prevalence, associated disability and management. S Afr Med J. 2000; 90(3): 262-6.	5 - Nonfatal Health Outcomes
311739	Scientific literature	Exploring the seasonality of reported treated malaria cases in Mpumalanga, South Africa	Silal SP, Barnes KI, Kok G, Mabuza A, Little F. Exploring the seasonality of reported treated malaria cases in Mpumalanga, South Africa. PLoS One. 2013; 8(10): e76640.	5 - Nonfatal Health Outcomes
209317	Scientific literature	Factors associated with mortality in HIV-infected people in rural and urban South Africa	Otwombe KN, Petzold M, Modisenyane T, Martinson NA, Chirwa T. Factors associated with mortality in HIV-infected people in rural and urban South Africa. Glob Health Action. 2014; 25488.	5 - Nonfatal Health Outcomes
229516	Scientific literature	Fetal alcohol syndrome among grade 1 schoolchildren in Northern Cape Province: prevalence and risk factors	Urban M, Chersich MF, Fourie LA, Chetty C, Olivier L, Viljoen D. Fetal alcohol syndrome among grade 1 schoolchildren in Northern Cape Province: prevalence and risk factors. S Afr Med J. 2008; 98(11): 877â€“82.	5 - Nonfatal Health Outcomes
130444	Scientific literature	Fetal alcohol syndrome epidemiology in a South African community: a second study of a very high prevalence area	Viljoen DL, Gossage JP, Brooke L, Adnams CM, Jones KL, Robinson LK, Hoyme HE, Snell C, Khaole NCO, Kodituwakku P, Asante KO, Findlay R, Quinton B, Marais A-S, Kalberg WO, May PA. Fetal alcohol syndrome epidemiology in a South African community: a second study of a very high prevalence area. J Stud Alcohol. 2005; 66(5): 593-604.	5 - Nonfatal Health Outcomes
270424	Scientific literature	Gastroschisis: a multi-centre comparison of management and outcome	Manson J, Ameh E, Canvassar N, Chen T, den Hoeve AV, Lever F, Hesse A, Millar A, Emil S, Ade-Ajayi N. Gastroschisis: a multi-centre comparison of management and outcome. Afr J Paediatr Surg. 2012; 9(1): 17-21.	5 - Nonfatal Health Outcomes
214502	Scientific literature	Gender differences in survival among adult patients starting antiretroviral therapy in South Africa: a multicentre cohort study	Cornell M, Schomaker M, Garone DB, Giddy J, Hoffmann CJ, Lessells R, Maskew M, Prozesky H, Wood R, Johnson LF, Egger M, Boulle A, Myer L, International Epidemiologic Databases to Evaluate AIDS Southern Africa Collaboration. Gender differences in survival among adult patients starting antiretroviral therapy in South Africa: a multicentre cohort study. PLoS Med. 2012; 9(9): e1001304.	5 - Nonfatal Health Outcomes

220509	Scientific literature	Genetic characterization of Cryptosporidium spp. in diarrhoeic children from four provinces in South Africa	Abu Samra N, Thompson PN, Jori F, Frean J, Poonsamy B, du Plessis D, Mogoye B, Xiao L. Genetic characterization of Cryptosporidium spp. in diarrhoeic children from four provinces in South Africa. Zoonoses Public Health. 2013; 60(2): 154-9.	5 - Nonfatal Health Outcomes
127450	Scientific literature	Glaucoma in Zulus: a population-based cross-sectional survey in a rural district in South Africa	Rotchford AP, Johnson GJ. Glaucoma in Zulus: a population-based cross-sectional survey in a rural district in South Africa. Arch Ophthalmol. 2002; 120(4): 471-8.	5 - Nonfatal Health Outcomes
166148	Scientific literature	Global numbers of infection and disease burden of soil transmitted helminth infections in 2010 [Unpublished data]	Pullan RL, Smith JL, Jasrasaria R, Brooker SJ. Global numbers of infection and disease burden of soil transmitted helminth infections in 2010 [Unpublished data]. Parasit Vectors. 2014; 7(37). [Unpublished data as provided by the Global Burden of Disease 2010 soil transmitted helminths expert group].	5 - Nonfatal Health Outcomes
111335	Scientific literature	Global variation in the prevalence and severity of asthma symptoms: phase three of the International Study of Asthma and Allergies in Childhood (ISAAC)	Lai CK, Beasley R, Crane J, Foliaki S, Shah J, Weiland S. Global variation in the prevalence and severity of asthma symptoms: phase three of the International Study of Asthma and Allergies in Childhood (ISAAC). Thorax. 2009; 64(6): 476-483.	5 - Nonfatal Health Outcomes
115570	Scientific literature	Global variations in prevalence of eczema symptoms in children from ISAAC Phase Three	Odhiambo JA, Williams HC, Clayton TO, Robertson CF, Asher MI, ISAAC Phase Three Study Group. Global variations in prevalence of eczema symptoms in children from ISAAC Phase Three. J Allergy Clin Immunol. 2009; 124(6): 1251-1258.	5 - Nonfatal Health Outcomes
125860	Scientific literature	Hepatitis C virus infection in chronic liver disease in Natal	Soni PN, Tait DR, Gopaul W, Sathar MA, Simjee AE. Hepatitis C virus infection in chronic liver disease in Natal. S Afr Med J. 1996; 86(1): 80-3.	5 - Nonfatal Health Outcomes
144917	Scientific literature	Hepatitis E in South Africa: evidence for sporadic spread and increased seroprevalence in rural areas	Tucker TJ, Kirsch RE, Louw SJ, Isaacs S, Kannemeyer J, Robson SC. Hepatitis E in South Africa: evidence for sporadic spread and increased seroprevalence in rural areas. J Med Virol. 1996; 50(2): 117-9.	5 - Nonfatal Health Outcomes
221657	Scientific literature	High HIV, HPV, and STI prevalence among young Western Cape, South African women: EVRI HIV prevention preparedness trial	Giuliano AR, Botha MH, Zeier M, Abrahamsen ME, Glashoff RH, van der Laan LE, Papenfuss M, Engelbrecht S, Schim van der Loeff MF, Sudenga SL, Torres BN, Kipping S, Taylor D. High HIV, HPV, and STI prevalence among young Western Cape, South African women: EVRI HIV prevention preparedness trial. J Acquir Immune Defic Syndr. 2015; 68(2): 227â€“35.	5 - Nonfatal Health Outcomes
221674	Scientific literature	High prevalence and incidence of asymptomatic sexually transmitted infections during pregnancy and postdelivery in KwaZulu Natal, South Africa	Moodley D, Moodley P, Sebitloane M, Soowamber D, McNaughton-Reyes HL, Groves AK, Maman S. High prevalence and incidence of asymptomatic sexually transmitted infections during pregnancy and postdelivery in KwaZulu Natal, South Africa. Sex Transm Dis. 2015; 42(1): 43â€“7.	5 - Nonfatal Health Outcomes
138394	Scientific literature	HIV infection among youth in a South African mining town is associated with herpes simplex virus-2 seropositivity and sexual behaviour	Auvert B, Ballard R, Campbell C, CaraÃ«l M, Carton M, Fehler G, Gouws E, MacPhail C, Taljaard D, Van Dam J, Williams B. HIV infection among youth in a South African mining town is associated with herpes simplex virus-2 seropositivity and sexual behaviour. AIDS. 2001; 15(7): 885-98.	5 - Nonfatal Health Outcomes
115558	Scientific literature	Household conditions, eczema symptoms and rhinitis symptoms: relationship with wheeze and severe wheeze in children living in the Polokwane area, South Africa	Wichmann J, Wolvaardt JE, Maritz C, Voyi KVV. Household conditions, eczema symptoms and rhinitis symptoms: relationship with wheeze and severe wheeze in children living in the Polokwane area, South Africa. Matern Child Health J. 2009; 13(1): 107-18.	5 - Nonfatal Health Outcomes
143258	Scientific literature	Human cystic echinococcosis in South Africa	Wahlers K, Menezes CN, Wong M, Mogoye B, Frean J, Romig T, Kern P, Grobusch MP. Human cystic echinococcosis in South Africa. Addict Behav Rep. 2011; 120(3): 179-84.	5 - Nonfatal Health Outcomes
221849	Scientific literature	Human immunodeficiency virus-1 infection correlates strongly with herpes simplex virus-2 (genital herpes) seropositivity in South African and United States blood donations	Benjamin RJ, Busch MP, Fang CT, Notari EP, Puren A, Schoub BD, Tobler LH, Hogrefe W, du P Heyns A, Stramer SL, Crookes RL. Human immunodeficiency virus-1 infection correlates strongly with herpes simplex virus-2 (genital herpes) seropositivity in South African and United States blood donations. Transfusion. 2008; 48(2): 295â€“303.	5 - Nonfatal Health Outcomes
280018	Scientific literature	Illicit drug use in South Africa: Findings from a 2008 national population-based survey	Peltzer K, Ramlagan S. Illicit drug use in South Africa: Findings from a 2008 national population-based survey. S Afr J Psychiatry. 2010; 16(1): a230.	5 - Nonfatal Health Outcomes
293963	Scientific literature	Immunogenicity, efficacy and serological correlate of protection of Salmonella typhi Vi capsular polysaccharide vaccine three years after immunization	Klugman KP, Koornhof HJ, Robbins JB, Le Cam NN. Immunogenicity, efficacy and serological correlate of protection of Salmonella typhi Vi capsular polysaccharide vaccine three years after immunization. Vaccine. 1996; 14(5): 435â€“8.	5 - Nonfatal Health Outcomes
161010	Scientific literature	Impact of hookworm infection and deworming on anaemia in non-pregnant populations: a systematic	Smith JL, Brooker S. Impact of hookworm infection and deworming on anaemia in non-pregnant populations: a systematic review. Trop Med Int Health. 2010; 15(7): 776â€“95.	5 - Nonfatal Health Outcomes

		review		
221896	Scientific literature	Impact of stepping stones on incidence of HIV and HSV-2 and sexual behaviour in rural South Africa: cluster randomised controlled trial	Jewkes R, Nduna M, Levin J, Jama N, Dunkle K, Puren A, Duvvury N. Impact of stepping stones on incidence of HIV and HSV-2 and sexual behaviour in rural South Africa: cluster randomised controlled trial. BMJ. 2008; a506.	5 - Nonfatal Health Outcomes
251360	Scientific literature	Incidence and severity of childhood pneumonia in the first year of life in a South African birth cohort: the Drakenstein Child Health Study	le Roux DM, Myer L, Nicol MP, Zar HJ. Incidence and severity of childhood pneumonia in the first year of life in a South African birth cohort: the Drakenstein Child Health Study. Lancet Glob Health. 2015; 3(2): e95â€“103.	5 - Nonfatal Health Outcomes
317974	Scientific literature	Incidence of invasive salmonella disease in sub-Saharan Africa: a multicentre population-based surveillance study	Marks F, von Kalckreuth V, Aaby P, Adu-Sarkodie Y, El Tayeb MA, Ali M, Aseffa A, Baker S, Biggs HM, Bjerregaard-Andersen M, others. Incidence of invasive salmonella disease in sub-Saharan Africa: a multicentre population-based surveillance study. Lancet Glob Health. 2017; 5(3): e310-323.	5 - Nonfatal Health Outcomes
269738	Scientific literature	Incidence, Remission and Mortality of Convulsive Epilepsy in Rural Northeast South Africa	Wagner RG, Bottomley C, Ngugi AK, Ibinda F, Gomez-Olive FX, Kahn K, Tollman S, Newton CR, , Wagner R, Twine R, Connor M, Collinson M, Masanja H, Mathew A, Kakooza A, Pariyo G, Peterson S, Ndyo-mughenyi D, Odhiambo R, Chengo E, Chabi M, Bauni E, Kamuyu G, Odera VM, Mageto JO, Ae-Ngibise K, Akpalu B, Akpalu A, Agbokey F, Adjei P, Owusu-Agyei S, Kleinschmidt I, Doku VC, Odermatt P, Neville B, Sander JW, White S, Nutman T, Wilkins P, Noh J. Incidence, Remission and Mortality of Convulsive Epilepsy in Rural Northeast South Africa. PLoS One. 2015; 10(6): e0129097.	5 - Nonfatal Health Outcomes
139930	Scientific literature	Increasing prevalence of genital herpes in developing countries: implications for heterosexual HIV transmission and STI control programmes	O'Farrell N. Increasing prevalence of genital herpes in developing countries: implications for heterosexual HIV transmission and STI control programmes. Sex Transm Infect. 1999; 75(6): 377-84.	5 - Nonfatal Health Outcomes
116703	Scientific literature	Infection by enteric adenoviruses, rotaviruses, and other agents in a rural African environment	Tiemessen CT, Wegerhoff FO, Erasmus MJ, Kidd AH. Infection by enteric adenoviruses, rotaviruses, and other agents in a rural African environment. J Med Virol. 1989; 28(3): 176-82.	5 - Nonfatal Health Outcomes
148240	Scientific literature	Infertility profile at King Edward VIII Hospital, Durban, South Africa	Chigumadzi PT, Moodley J, Bagratee J. Infertility profile at King Edward VIII Hospital, Durban, South Africa. Trop Doct. 1998; 28(3): 168-72.	5 - Nonfatal Health Outcomes
230985	Scientific literature	Influenza surveillance in 15 countries in Africa, 2006-2010	Radin JM, Katz MA, Tempia S, Talla Nzussouo N, Davis R, Duque J, Adedeji A, Adjabeng MJ, Ampofo WK, Ayele W, Bakamutumaho B, Barakat A, Cohen AL, Cohen C, Dalhatu IT, Daouda C, Dueger E, Francisco M, Heraud J-M, Jima D, Kabanda A, Kadjo H, Kandeel A, Bi Shamamba SK, Kasolo F, Kronmann KC, Mazaba Liwewe ML, Lutwama JJ, Matonya M, Mmbaga V, Mott JA, Muhimpundu MA, Muthoka P, Njuguna H, Randrianasolo L, Refaey S, Sanders C, Talaat M, Theo A, Valente F, Venter M, Woodfill C, Bresee J, Moen A, Widdowson M-A. Influenza surveillance in 15 countries in Africa, 2006-2010. J Infect Dis. 2012; S14â€“21.	5 - Nonfatal Health Outcomes
110035	Scientific literature	Inherited haemoglobin variants in a South African population	Bird AR, Ellis P, Wood K, Mathew C, Karabus C. Inherited haemoglobin variants in a South African population. J Med Genet. 1987; 24(4): 215-9.	5 - Nonfatal Health Outcomes
120490	Scientific literature	International variation in the prevalence of COPD (the BOLD Study): a population-based prevalence study	Buist AS,Â McBurnie MA,Â Vollmer WM,Â Gillespie S,Â Burney P,Â Mannino DM,Â Menezes AM,Â Sullivan SD,Â Lee TA,Â Weiss KB,Â Jensen RL,Â Marks GB,Â Gulsvik A,Nizankowska-Mogilnicka E, BOLD Collaborative Research Group. International variation in the prevalence of COPD (the BOLD Study): a population-based prevalence study. Lancet. 2007; 9589(9589): 741-50.	5 - Nonfatal Health Outcomes
294182	Scientific literature	Intestinal parasitic infections in black scholars in northern KwaZulu	Schutte CHJ, Eriksson IM, Anderson CB, Lamprecht T. Intestinal parasitic infections in black scholars in northern KwaZulu. S Afr Med J. 1981; 60(4): 137-141.	5 - Nonfatal Health Outcomes
163087	Scientific literature	Intimate partner violence, relationship power inequity, and incidence of HIV infection in young women in South Africa: a cohort study	Jewkes RK, Dunkle K, Nduna M, Shai N. Intimate partner violence, relationship power inequity, and incidence of HIV infection in young women in South Africa: a cohort study. Lancet. 2010; 376(9734): 41â€“8.	5 - Nonfatal Health Outcomes
138326	Scientific literature	Intravaginal practices, HIV and other sexually transmitted diseases among South African women	Myer L, Denny L, De Souza M, Barone MA, Wright TC Jr, Kuhn L. Intravaginal practices, HIV and other sexually transmitted diseases among South African women. Sex Transm Dis. 2004; 31(3): 174-9.	5 - Nonfatal Health Outcomes
408097	Scientific literature	Invasive Pneumococcal Disease in Neonates Prior to Pneumococcal	Moodley K, Coovadia YM, Cohen C, Meiring S, Lengana S, De Gouveia L, von Mollendorf C, Crowther-Gibson P, Quan V, Eley B,	5 - Nonfatal Health

		Conjugate Vaccine Use in South Africa: 2003-2008	Reubenson G, Nana T, von Gottberg A. Invasive Pneumococcal Disease in Neonates Prior to Pneumococcal Conjugate Vaccine Use in South Africa: 2003-2008. Pediatr Infect Dis J. 2019; 38(4): 424-430.	Outcomes
255133	Scientific literature	Iron, folate and vitamin B12 nutrition and anaemia in black preschool children in the northern Transvaal	Van der Westhuyzen J, Van Tonder SV, Gilbertson I, Metz J. Iron, folate and vitamin B12 nutrition and anaemia in black preschool children in the northern Transvaal. S Afr Med J. 1986; 70(3): 143-6.	5 - Nonfatal Health Outcomes
104223	Scientific literature	Is eczema really on the increase worldwide	Williams H, Stewart A, Von Mutius E, Cookson W, Anderson HR. Is eczema really on the increase worldwide. J Allergy Clin Immunol. 2008; 121(4): 947-954.	5 - Nonfatal Health Outcomes
214900	Scientific literature	Long term outcomes of antiretroviral therapy in a large HIV/AIDS care clinic in urban South Africa: a prospective cohort study	Sanne IM, Westreich D, Macphail AP, Rubel D, Majuba P, Van Rie A. Long term outcomes of antiretroviral therapy in a large HIV/AIDS care clinic in urban South Africa: a prospective cohort study. J Int AIDS Soc. 2009; 38.	5 - Nonfatal Health Outcomes
214515	Scientific literature	Long-term antiretroviral treatment outcomes in seven countries in the Caribbean	Koenig SP, Rodriguez LA, Bartholomew C, Edwards A, Carmichael TE, Barrow G, CabiÃ© A, Hunter R, Vasquez-Mora G, Quava-Jones A, Adomakoh N, Peter Figueroa J, Liautaud B, Torres M, Pape JW. Long-term antiretroviral treatment outcomes in seven countries in the Caribbean. J Acquir Immune Defic Syndr. 2012; 59(4): e60-71.	5 - Nonfatal Health Outcomes
214531	Scientific literature	Low haemoglobin predicts early mortality among adults starting antiretroviral therapy in an HIV care programme in South Africa: a cohort study	Russell EC, Charalambous S, Pemba L, Churchyard GJ, Grant AD, Fielding K. Low haemoglobin predicts early mortality among adults starting antiretroviral therapy in an HIV care programme in South Africa: a cohort study. BMC Public Health. 2010; 433.	5 - Nonfatal Health Outcomes
127880	Scientific literature	Magnesium supplementation and perinatal hypoxia: outcome of a parallel group randomised trial in pregnancy	Harrison V, Fawcus S, Jordaan E. Magnesium supplementation and perinatal hypoxia: outcome of a parallel group randomised trial in pregnancy. BJOG. 2007; 114(8): 994-1002.	5 - Nonfatal Health Outcomes
311749	Scientific literature	Malaria control in South Africa 2000-2010: beyond MDG6	Moonasar D, Nuthulaganti T, Kruger P, Mabuza A, Rasiswi E, Benson F, Maharaj R. Malaria control in South Africa 2000-2010: beyond MDG6. Malar J. 2012; 11: 294.	5 - Nonfatal Health Outcomes
413891	Scientific literature	Maternal near-miss audit in the Metro West maternity service, Cape Town, South Africa: A retrospective observational study	Iwuh IA, Fawcus S, Schoeman L. Maternal near-miss audit in the Metro West maternity service, Cape Town, South Africa: A retrospective observational study. S Afr Med J. 2018; 108(3): 171-175.	5 - Nonfatal Health Outcomes
96507	Scientific literature	Morbidity from urinary schistosomiasis in relation to intensity of infection in the Natal Province of South Africa	Cooppan RM, Schutte CH, Mayet FG, Dingle CE, Van Deventer JM, Mosese PG. Morbidity from urinary schistosomiasis in relation to intensity of infection in the Natal Province of South Africa. Am J Trop Med Hyg. 1986; 35(4): 765-76.	5 - Nonfatal Health Outcomes
93645	Scientific literature	Mortality and loss to follow-up among HAART initiators in rural South Africa	MacPherson P, Moshabela M, Martinson N, Pronyk P. Mortality and loss to follow-up among HAART initiators in rural South Africa. Trans R Soc Trop Med Hyg. 2009; 103(6): 588-93.	5 - Nonfatal Health Outcomes
214568	Scientific literature	Mortality and morbidity among HIV type-1-infected patients during the first 5 years of a multicountry HIV workplace programme in Africa	Van der Borght SF, Clevenbergh P, Rijckborst H, Nsalou P, Onyia N, Lange JM, de Wit TFR, Van der Loeff MFS. Mortality and morbidity among HIV type-1-infected patients during the first 5 years of a multicountry HIV workplace programme in Africa. Antivir Ther. 2009; 14(1): 63-74.	5 - Nonfatal Health Outcomes
214562	Scientific literature	Mortality during the first year of potent antiretroviral therapy in HIV-1-infected patients in 7 sites throughout Latin America and the Caribbean	Tuboi SH, Schechter M, McGowan CC, Cesar C, Krolewiecki A, Cahn P, Wolff M, Pape JW, Padgett D, Madero JS, Gotuzzo E, Masys DR, Shepherd BE. Mortality during the first year of potent antiretroviral therapy in HIV-1-infected patients in 7 sites throughout Latin America and the Caribbean. J Acquir Immune Defic Syndr. 2009; 51(5): 615-23.	5 - Nonfatal Health Outcomes
209349	Scientific literature	Mortality in patients with HIV-1 infection starting antiretroviral therapy in South Africa, Europe, or North America: a collaborative analysis of prospective studies	Boulle A, Schomaker M, May MT, Hogg RS, Shepherd BE, Monge S, Keiser O, Lampe FC, Giddy J, Ndirangu J, Garone D, Fox M, Ingle SM, Reiss P, Dabis F, Costagliola D, Castagna A, Ehren K, Campbell C, Gill MJ, Saag M, Justice AC, Guest J, Crane HM, Egger M, Sterne JAC. Mortality in patients with HIV-1 infection starting antiretroviral therapy in South Africa, Europe, or North America: a collaborative analysis of prospective studies. PLoS Med. 2014; 11(9): e1001718.	5 - Nonfatal Health Outcomes
156877	Scientific literature	Mortality in women of reproductive age in rural South Africa	Nabukalu D, Klipstein-Grobusch K, Herbst K, Newell M-L. Mortality in women of reproductive age in rural South Africa. Glob Health Action. 2013; 22834.	5 - Nonfatal Health Outcomes
214567	Scientific literature	Mortality of HIV-infected patients starting antiretroviral therapy in sub-Saharan Africa: comparison with HIV-unrelated mortality	Brinkhof MWG, Boulle A, Weigel R, Messou E, Mathers C, Orrell C, Dabis F, Pascoe M, Egger M, International Epidemiological Databases to Evaluate AIDS (IeDEA). Mortality of HIV-infected patients starting antiretroviral therapy in sub-Saharan Africa: comparison with HIV-unrelated mortality. PLoS Med. 2009; 6(4):	5 - Nonfatal Health Outcomes

			e1000066.	
256499	Scientific literature	Neonatal hyperbilirubinemia and Rhesus disease of the newborn: incidence and impairment estimates for 2010 at regional and global levels	Bhutani VK, Zipursky A, Blencowe H, Khanna R, Sgro M, Ebbesen F, Bell J, Mori R, Slusher TM, Fahmy N, Paul VK, Du L, Okolo AA, de Almeida MF, Olusanya BO, Kumar P, Cousens S, Lawn JE. Neonatal hyperbilirubinemia and Rhesus disease of the newborn: incidence and impairment estimates for 2010 at regional and global levels. Pediatr Res. 2013; 74(Suppl 1): 86-100.	5 - Nonfatal Health Outcomes
161342	Scientific literature	Neurological complications associated with the preeclampsia/eclampsia syndrome	Okanloma KA, Moodley J. Neurological complications associated with the preeclampsia/eclampsia syndrome. Int J Gynaecol Obstet. 2000; 71(3): 223-5.	5 - Nonfatal Health Outcomes
279493	Scientific literature	No decrease in annual risk of tuberculosis infection in endemic area in Cape Town, South Africa	Kritzinger FE, den Boon S, Verver S, Enarson DA, Lombard CJ, Borgdorff MW, Gie RP, Beyers N. No decrease in annual risk of tuberculosis infection in endemic area in Cape Town, South Africa. Trop Med Int Health. 2009; 14(2): 136â€“42.	5 - Nonfatal Health Outcomes
272780	Scientific literature	Norovirus prevalence and estimated viral load in symptomatic and asymptomatic children from rural communities of Vhembe district, South Africa	Kabue JP, Meader E, Hunter PR, Potgieter N. Norovirus prevalence and estimated viral load in symptomatic and asymptomatic children from rural communities of Vhembe district, South Africa. J Clin Virol. 2016; 84: 12â€“8.	5 - Nonfatal Health Outcomes
419398	Scientific literature	Nurses at risk for occupationally acquired blood-borne virus infection at a South African academic hospital	Mosendane T, Kew MC, Osih R, Mahomed A. Nurses at risk for occupationally acquired blood-borne virus infection at a South African academic hospital. S Afr Med J. 2012; 102(3 Pt 1): 1536.	5 - Nonfatal Health Outcomes
144174	Scientific literature	Nutritional status and dietary intakes of children aged 2-5 years and their caregivers in a rural South African community	Faber M, Jogessar VB, BenadÃ© AJ. Nutritional status and dietary intakes of children aged 2-5 years and their caregivers in a rural South African community. Int J Food Sci Nutr. 2001; 52(5): 401-11.	5 - Nonfatal Health Outcomes
128455	Scientific literature	Occurrence and clinical implications of red-cell glucose-6-phosphate dehydrogenase deficiency in South African racial groups	Bernstein RE. Occurrence and clinical implications of red-cell glucose-6-phosphate dehydrogenase deficiency in South African racial groups. S Afr Med J. 1963; 447-51.	5 - Nonfatal Health Outcomes
143219	Scientific literature	On-site rapid antenatal syphilis screening with an immunochromatographic strip improves case detection and treatment in rural South African clinics	Bronzan RN,Â Mwesigwa-Kayongo DC,Â Narkunas D,Â Schmid GP,Â Neilsen GA,Â Ballard RC,Â Karuhije P,Â Ddamba J,Â Nombekela E,Â Hoyi G,Â Dlali P,Â Makwedini N,Fehler HG,Â Blandford JM,Â Ryan C. On-site rapid antenatal syphilis screening with an immunochromatographic strip improves case detection and treatment in rural South African clinics. Sex Transm Dis. 2007; 34(7 Suppl): S55-60.	5 - Nonfatal Health Outcomes
119179	Scientific literature	Oral health in Hlabisa, KwaZulu/Natal--a rural school and community based survey	Brindle R, Wilkinson D, Harrison A, Connolly C, Cleaton-Jones P. Oral health in Hlabisa, KwaZulu/Natal--a rural school and community based survey. Int Dent J. 2000; 50(1): 13-20.	5 - Nonfatal Health Outcomes
119056	Scientific literature	Oral health in South Africa	Van Wyk PJ, van Wyk C. Oral health in South Africa. Int Dent J. 2004; 54(6): 373-7.	5 - Nonfatal Health Outcomes
114969	Scientific literature	Osteoarthrosis in a rural South African Negro population	Solomon L, Beighton P, Lawrence JS. Osteoarthrosis in a rural South African Negro population. Ann Rheum Dis. 1976; 35(3): 274-8.	5 - Nonfatal Health Outcomes
214903	Scientific literature	Outcomes of antiretroviral treatment in programmes with and without routine viral load monitoring in Southern Africa	Keiser O, Chi BH, Gsponer T, Boulle A, Orrell C, Phiri S, Maxwell N, Maskew M, Prozesky H, Fox MP, Westfall A, Egger M, IeDEA Southern Africa Collaboration. Outcomes of antiretroviral treatment in programmes with and without routine viral load monitoring in Southern Africa. AIDS. 2011; 25(14): 1761-9.	5 - Nonfatal Health Outcomes
214911	Scientific literature	Outcomes of antiretroviral treatment program in Ethiopia: retention of patients in care is a major challenge and varies across health facilities	Assefa Y, Kiflie A, Tesfaye D, Mariam DH, Kloos H, Edwin W, Laga M, Van Damme W. Outcomes of antiretroviral treatment program in Ethiopia: retention of patients in care is a major challenge and varies across health facilities. BMC Health Serv Res. 2011; 81.	5 - Nonfatal Health Outcomes
214514	Scientific literature	Outcomes of antiretroviral treatment programs in rural Southern Africa	Wandeler G, Keiser O, Pfeiffer K, Pestilli S, Fritz C, Labhardt ND, Mbofana F, Mudyiradima R, Emmel J, Egger M, Ehmer J, SolidarMed ART program and IeDEA-Southern Africa. Outcomes of antiretroviral treatment programs in rural Southern Africa. J Acquir Immune Defic Syndr. 2012; 59(2): e9-16.	5 - Nonfatal Health Outcomes
138323	Scientific literature	P3.011 Dry Swab Evaluation by Roche 4800 CT/NG and the Presto-Plus: Cross-Sectional Study of Genital, Rectal and Pharyngeal Chlamydia and Gonorrhoea Infection in Women in Rural South Africa	Waaij DJ de, Dubbink J, Eem L van der, Bos MLA, Ouburg S, Lewis DA, Struthers H, McIntyre JA, MorrÃ© SA, Peters RPH. P3.011 Dry Swab Evaluation by Roche 4800 CT/NG and the Presto-Plus: Cross-Sectional Study of Genital, Rectal and Pharyngeal Chlamydia and Gonorrhoea Infection in Women in Rural South Africa. Sex Transm Infect. 2013; 89(Suppl 1): A151-A151.	5 - Nonfatal Health Outcomes
256181	Scientific literature	Peritoneal dialysis in Africa	Abu-Aisha H, Elamin S. Peritoneal dialysis in Africa. Perit Dial Int. 2010; 30(1): 23â€“8.	5 - Nonfatal Health

				Outcomes
140109	Scientific literature	Predominance of heart failure in the Heart of Soweto Study cohort: emerging challenges for urban African communities	Stewart S, Wilkinson D, Hansen C, Vaghela V, Mvungi R, McMurray J, Sliwa K. Predominance of heart failure in the Heart of Soweto Study cohort: emerging challenges for urban African communities. Circulation. 2008; 118(23): 2360-7.	5 - Nonfatal Health Outcomes
122290	Scientific literature	Prevalence and causes of functional low vision in school-age children: results from standardized population surveys in Asia, Africa, and Latin America	Gilbert CE, Ellwein LB. Prevalence and causes of functional low vision in school-age children: results from standardized population surveys in Asia, Africa, and Latin America. Invest Ophthalmol Vis Sci. 2008; 49(3): 877-81.	5 - Nonfatal Health Outcomes
127444	Scientific literature	Prevalence and causes of low vision and blindness in northern KwaZulu	Cook CD, Knight SE, Crofton-Briggs I. Prevalence and causes of low vision and blindness in northern KwaZulu. S Afr Med J. 1993; 83(8): 590-3.	5 - Nonfatal Health Outcomes
419470	Scientific literature	Prevalence and correction of near vision impairment at seven sites in China, India, Nepal, Niger, South Africa, and the United States	He M, Abdou A, Naidoo KS, Sapkota YD, Thulasiraj RD, Varma R, Zhao J, Ellwein LB. Prevalence and correction of near vision impairment at seven sites in China, India, Nepal, Niger, South Africa, and the United States. Am J Ophthalmol. 2012; 154(1): 107-116e1.	5 - Nonfatal Health Outcomes
271104	Scientific literature	Prevalence and factors associated with depressive symptoms among young women and men in the Eastern Cape Province, South Africa	Nduna M, Jewkes RK, Dunkle KL, Jama Shai NP, Colman I. Prevalence and factors associated with depressive symptoms among young women and men in the Eastern Cape Province, South Africa. J Child Adolesc Ment Health. 2013; 25(1): 43â€“54.	5 - Nonfatal Health Outcomes
161079	Scientific literature	Prevalence and impact of dental pain in 8-10-year-olds in the western Cape	Naidoo S, Chikte UM, Sheiham A. Prevalence and impact of dental pain in 8-10-year-olds in the western Cape. SADJ. 2001; 56(11): 521-3.	5 - Nonfatal Health Outcomes
138473	Scientific literature	Prevalence and incidence of blindness due to age-realted cataract in the rural areas of South Africa	Cook D. Prevalence and incidence of blindness due to age-realted cataract in the rural areas of South Africa. S Afr Med J. 1995; 85(1): 26-7.	5 - Nonfatal Health Outcomes
221712	Scientific literature	Prevalence and incidence of Trichomonas vaginalis infections in women participating in a clinical trial in Durban, South Africa	Naidoo S, Wand H. Prevalence and incidence of Trichomonas vaginalis infections in women participating in a clinical trial in Durban, South Africa. Sex Transm Infect. 2013; 89(6): 519â€“22.	5 - Nonfatal Health Outcomes
218930	Scientific literature	Prevalence and risk factors for active convulsive epilepsy in rural northeast South Africa	Wagner RG, Ngugi AK, Twine R, Bottomley C, Kamuyu G, G?mez-Oliv? FX, Connor MD, Collinson MA, Kahn K, Tollman S, Newton CR. Prevalence and risk factors for active convulsive epilepsy in rural northeast South Africa. Epilepsy Res. 2014; 108(4): 782-91.	5 - Nonfatal Health Outcomes
124509	Scientific literature	Prevalence of active convulsive epilepsy in sub-Saharan Africa and associated risk factors: cross-sectional and case-control studies	Ngugi AK, Bottomley C, Kleinschmidt I, Wagner RG, Kakooza-Mwesige A, Ae-Ngibise K, Owusu-Agyei S, Masanja H, Kamuyu G, Odhiambo R, Chengo E, Sander JW, Newton CR, SEEDS group. Prevalence of active convulsive epilepsy in sub-Saharan Africa and associated risk factors: cross-sectional and case-control studies. Lancet Neurol. 2013; 12(3): 253-63.	5 - Nonfatal Health Outcomes
233558	Scientific literature	Prevalence of Campylobacter species, Helicobacter pylori and Arcobacter species in stool samples from the Venda region, Limpopo, South Africa: studies using molecular diagnostic methods	Samie A, Obi CL, Barrett LJ, Powell SM, Guerrant RL. Prevalence of Campylobacter species, Helicobacter pylori and Arcobacter species in stool samples from the Venda region, Limpopo, South Africa: studies using molecular diagnostic methods. J Infect. 2007; 54(6): 558â€“66.	5 - Nonfatal Health Outcomes
124311	Scientific literature	Prevalence of cardiovascular diseases and associated risk factors in a rural black population of South Africa	Alberts M, Urdal P, Steyn K, Stensvold I, Tverdal A, Nel JH, Steyn NP. Prevalence of cardiovascular diseases and associated risk factors in a rural black population of South Africa. Eur J Prev Cardiol. 2005; 12(4): 347-54.	5 - Nonfatal Health Outcomes
116168	Scientific literature	Prevalence of childhood disability in rural KwaZulu-Natal	Couper J. Prevalence of childhood disability in rural KwaZulu-Natal. S Afr Med J. 2002; 92(7): 549-52.	5 - Nonfatal Health Outcomes
150611	Scientific literature	Prevalence of emotional, physical and sexual abuse of women in three South African provinces	Jewkes R, Penn-Kekana L, Levin J, Ratsaka M, Schrieber M. Prevalence of emotional, physical and sexual abuse of women in three South African provinces. S Afr Med J. 2001; 91(5): 421-8.	5 - Nonfatal Health Outcomes
125453	Scientific literature	Prevalence Of Epilepsy And General Knowledge About Neurocysticiercosis At Nkalukeni Village, South Africa	Del Rio-Romero AH, Foyaca-Sibat H, Ibanez-Valdes L, Vega-Novoa E. Prevalence Of Epilepsy And General Knowledge About Neurocysticiercosis At Nkalukeni Village, South Africa. Internet J Neurol. 2005; 3(2).	5 - Nonfatal Health Outcomes
228166	Scientific literature	Prevalence of fetal alcohol syndrome in a South African city with a predominantly Black African population	Urban MF, Olivier L, Viljoen D, Lombard C, Louw JG, Drotsky LM, Temmerman M, Chersich MF. Prevalence of fetal alcohol syndrome in a South African city with a predominantly Black African population. Alcohol Clin Exp Res. 2015; 39(6): 1016â€“26.	5 - Nonfatal Health Outcomes
232780	Scientific literature	Prevalence of gastrointestinal pathogenic bacteria in patients with	Kullin B, Meggersee R, Dâ€™Alton J, Galvao B, Rajabally N, Whitelaw A, Bamford C, Reid SJ, Abratt VR. Prevalence of	5 - Nonfatal Health

		diarrhoea attending Groote Schuur Hospital, Cape Town, South Africa	gastrointestinal pathogenic bacteria in patients with diarrhoea attending Groote Schuur Hospital, Cape Town, South Africa. S Afr Med J. 2015; 105(2): 121â€“5.	Outcomes
121205	Scientific literature	Prevalence of genital mycoplasmas, ureaplasmas and chlamydia in pregnancy	Govender S, Theron GB, Odendaal HJ, Chalkley LJ. Prevalence of genital mycoplasmas, ureaplasmas and chlamydia in pregnancy. J Obstet Gynaecol. 2009; 29(8): 698-701.	5 - Nonfatal Health Outcomes
116800	Scientific literature	Prevalence of intestinal parasitic and bacterial pathogens in diarrhoeal and non-diarroeal human stools from Vhembe district, South Africa	Samie A, Guerrant RL, Barrett L, Bessong PO, Igumbor EO, Obi CL. Prevalence of intestinal parasitic and bacterial pathogens in diarrhoeal and non-diarroeal human stools from Vhembe district, South Africa. J Health Popul Nutr. 2009; 27(6): 739-45.	5 - Nonfatal Health Outcomes
120216	Scientific literature	Prevalence of minor psychiatric disorders in an adult African rural community in South Africa	Bhagwanjee A PA, Paruk Z, Petersen I, Subedar H. Prevalence of minor psychiatric disorders in an adult African rural community in South Africa. Psychol Med. 1998; 28(5): 1137-47.	5 - Nonfatal Health Outcomes
134231	Scientific literature	Prevalence of vaginitis, syphilis and HIV infection in women in the Orange Free State	Cronje HS, Joubert G, Muir A, Chapman RD, Divall P, Bam RH. Prevalence of vaginitis, syphilis and HIV infection in women in the Orange Free State. S Afr Med J. 1994; 84(9): 602-5.	5 - Nonfatal Health Outcomes
138472	Scientific literature	Prevalence, Causes and Socio-Economic Determinants of Vision Loss in Cape Town, South Africa. Atashili J, editor	Cockburn N, Steven D, Lecuona K, Joubert F, Rogers G, Cook C, Polack S. Prevalence, Causes and Socio-Economic Determinants of Vision Loss in Cape Town, South Africa. Atashili J, editor. PLoS One. 2012; 7(2): e30718.	5 - Nonfatal Health Outcomes
128454	Scientific literature	Primaquine-sensitivity of red cells in various races in Southern Africa	Charlton RW, Bothwell TH. Primaquine-sensitivity of red cells in various races in Southern Africa. BMJ. 1961; 1(5230): 941-4.	5 - Nonfatal Health Outcomes
214510	Scientific literature	Prognosis of patients with HIV-1 infection starting antiretroviral therapy in sub-Saharan Africa: a collaborative analysis of scale-up programmes	May M, Boulle A, Phiri S, Messou E, Myer L, Wood R, Keiser O, Sterne JAC, Dabis F, Egger M, IeDEA Southern Africa and West Africa. Prognosis of patients with HIV-1 infection starting antiretroviral therapy in sub-Saharan Africa: a collaborative analysis of scale-up programmes. Lancet. 2010; 376(9739): 449-57.	5 - Nonfatal Health Outcomes
109108	Scientific literature	Prospective hospital-based surveillance to estimate rotavirus disease burden in the Gauteng and North West Province of South Africa during 2003-2005	Mapaseka SL, Dewar JB, van der Merwe L, Geyer A, Tumbo J, Zweygarth M, Bos P, Esona MD, Steele AD, Sommerfelt H. Prospective hospital-based surveillance to estimate rotavirus disease burden in the Gauteng and North West Province of South Africa during 2003-2005. J Infect Dis. 2010; 202(Suppl): 131-138.	5 - Nonfatal Health Outcomes
342267	Scientific literature	Psychoactive substance use among young people: findings of a multi-center study in three African countries	Nkowane, M.A., Rocha-Silva, L., Saxena, S., Mbatia, J., Ndubani, P. and Weir-Smith, G. Psychoactive substance use among young people: findings of a multi-center study in three African countries. Contemp Drug Probs. 2004; 31(2): 329-356.	5 - Nonfatal Health Outcomes
145690	Scientific literature	Racial differences in the seroprevalence of hepatitis A virus infection in Natal/KwaZulu, South Africa	Sathar MA, Soni PN, Fernandes-Costa FJ, Wittenberg DF, Simjee AE. Racial differences in the seroprevalence of hepatitis A virus infection in Natal/KwaZulu, South Africa. J Med Virol. 1994; 44(1): 9-12.	5 - Nonfatal Health Outcomes
303385	Scientific literature	Rapid assessment of avoidable blindness in the northern eThekwini district of KwaZulu-Natal Province, South Africa	Govender P, Ramson P, Visser L, Naidoo KS. Rapid assessment of avoidable blindness in the northern eThekwini district of KwaZulu-Natal Province, South Africa. Afr Vis Eye Health. 2015; 74(1): 1-7.	5 - Nonfatal Health Outcomes
93598	Scientific literature	Rates and causes of child mortality in an area of high HIV prevalence in rural South Africa	Garrib A, Jaffar S, Knight S, Bradshaw D, Bennish ML. Rates and causes of child mortality in an area of high HIV prevalence in rural South Africa. Trop Med Int Health. 2006; 11(12): 1841-8.	5 - Nonfatal Health Outcomes
161004	Scientific literature	Reassessment of the cost of chronic helmintic infection: a meta-analysis of disability-related outcomes in endemic schistosomiasis	King CH, Dickman K, Tisch DJ. Reassessment of the cost of chronic helmintic infection: a meta-analysis of disability-related outcomes in endemic schistosomiasis. Lancet. 2005; 365(9470): 1561â€“9.	5 - Nonfatal Health Outcomes
136314	Scientific literature	Rectal misoprostol in the prevention of postpartum hemorrhage: a placebo-controlled trial	Bamigboye AA, Hofmeyr GJ, Merrell DA. Rectal misoprostol in the prevention of postpartum hemorrhage: a placebo-controlled trial. Am J Obstet Gynecol. 1998; 179(4): 1043-6.	5 - Nonfatal Health Outcomes
214541	Scientific literature	Reducing mortality with cotrimoxazole preventive therapy at initiation of antiretroviral therapy in South Africa	Hoffmann CJ, Fielding KL, Charalambous S, Innes C, Chaisson RE, Grant AD, Churchyard GJ. Reducing mortality with cotrimoxazole preventive therapy at initiation of antiretroviral therapy in South Africa. AIDS. 2010; 24(11): 1709-16.	5 - Nonfatal Health Outcomes
123670	Scientific literature	Respiratory syncytial virus infection: denominator-based studies in Indonesia, Mozambique, Nigeria and South Africa	Robertson SE, Roca A, Alonso P, Simoes EAF, Kartasasmita CB, Olaleye DO, Odaibo GN, Collinson M, Venter M, Zhu Y, Wright PF. Respiratory syncytial virus infection: denominator-based studies in Indonesia, Mozambique, Nigeria and South Africa. Bull World Health Organ. 2004; 82(12): 914-22.	5 - Nonfatal Health Outcomes
221472	Scientific literature	Respiratory viral coinfections identified by a 10-plex real-time reverse-transcription polymerase chain reaction assay in patients hospitalized with severe acute	Pretorius MA, Madhi SA, Cohen C, Naidoo D, Groome M, Moyes J, Buys A, Walaza S, Dawood H, Chhagan M, Haffjee S, Kahn K, Puren A, Venter M. Respiratory viral coinfections identified by a 10-plex real-time reverse-transcription polymerase chain reaction assay in patients hospitalized with severe acute respiratory illness--South	5 - Nonfatal Health Outcomes

		respiratory illness--South Africa, 2009-2010	Africa, 2009-2010. J Infect Dis. 2012; S159-65.	
354012	Scientific literature	Reviewing South Africaâ€™s malaria elimination strategy (2012â€“2018): progress, challenges and priorities	Raman J, Morris N, Frean J, Brooke B, Blumberg L, Kruger P, Mabusa A, Raswiswi E, Shandukani B, Misani E, Groepe M-A, Moonasar D. Reviewing South Africaâ€™s malaria elimination strategy (2012â€“2018): progress, challenges and priorities. Malar J. 2016; 15(1).	5 - Nonfatal Health Outcomes
138324	Scientific literature	Risk of having a sexually transmitted infection in women presenting at a termination of pregnancy clinic in Pretoria, South Africa	De Jongh M, Lekalakala MR, Le Roux M, Hoosen AA. Risk of having a sexually transmitted infection in women presenting at a termination of pregnancy clinic in Pretoria, South Africa. J Obstet Gynaecol. 2010; 30(5): 480-3.	5 - Nonfatal Health Outcomes
341237	Scientific literature	Risk-taking behaviour of Cape Peninsula high-school students. Part V. Drug use	Flisher AJ, Ziervogel CF, Chalton DO, Leger PH, Robertson BA. Risk-taking behaviour of Cape Peninsula high-school students. Part V. Drug use. S Afr Med J. 1993; 83(7): 483â€“5.	5 - Nonfatal Health Outcomes
222161	Scientific literature	Safety and efficacy of the HVTN 503/Phambili study of a clade-B-based HIV-1 vaccine in South Africa: a double-blind, randomised, placebo-controlled test-of-concept phase 2b study	Gray GE, Allen M, Moodie Z, Churchyard G, Bekker L-G, Nchabeleng M, Mlisana K, Metch B, de Bruyn G, Latka MH, Roux S, Mathebula M, Naicker N, Ducar C, Carter DK, Puren A, Eaton N, McElrath MJ, Robertson M, Corey L, Kublin JG, HVTN 503/Phambili study team. Safety and efficacy of the HVTN 503/Phambili study of a clade-B-based HIV-1 vaccine in South Africa: a double-blind, randomised, placebo-controlled test-of-concept phase 2b study. Lancet Infect Dis. 2011; 11(7): 507â€“15.	5 - Nonfatal Health Outcomes
214532	Scientific literature	Scale-up of a decentralized HIV treatment programme in rural KwaZulu-Natal, South Africa: does rapid expansion affect patient outcomes?	Mutevedzi PC, Lessells RJ, Heller T, BÃ¤rnighausen T, Cooke GS, Newell M-L. Scale-up of a decentralized HIV treatment programme in rural KwaZulu-Natal, South Africa: does rapid expansion affect patient outcomes?. Bull World Health Organ. 2010; 88(8): 593-600.	5 - Nonfatal Health Outcomes
279855	Scientific literature	SCHISTOSOMA-HAEMATOBIUM IN TRANSKEI - A PRELIMINARY SURVEY CONDUCTED IN THE NGQELENI DISTRICT	MQOQI N, DYE A. SCHISTOSOMA-HAEMATOBIUM IN TRANSKEI - A PRELIMINARY SURVEY CONDUCTED IN THE NGQELENI DISTRICT. S Afr J Sci. 1992; 88(8): 445â€“7.	5 - Nonfatal Health Outcomes
279326	Scientific literature	Screening for TB in high school adolescents in a high burden setting in South Africa	Mahomed H, Ehrlich R, Hawkridge T, Hatherill M, Geiter L, Kafaar F, Abrahams DA, Mulenga H, Tameris M, Geldenhuys H, Hanekom WA, Verver S, Hussey GD. Screening for TB in high school adolescents in a high burden setting in South Africa. Tuberc (Edinb). 2013; 93(3): 357â€“62.	5 - Nonfatal Health Outcomes
145687	Scientific literature	Sero-epidemiology of hepatitis A in black South African children	Abdool Karim SS, Coutsoudis A. Sero-epidemiology of hepatitis A in black South African children. S Afr Med J. 1993; 83(10): 748-50.	5 - Nonfatal Health Outcomes
218643	Scientific literature	Serological screening for sexually transmitted infections in pregnancy: is there any value in re-screening for HIV and syphilis at the time of delivery?	Qolohle DC, Hoosen AA, Moodley J, Smith AN, Mlisana KP. Serological screening for sexually transmitted infections in pregnancy: is there any value in re-screening for HIV and syphilis at the time of delivery?. Genitourin Med. 1995; 71(2): 65-7.	5 - Nonfatal Health Outcomes
214517	Scientific literature	Seven-year experience of a primary care antiretroviral treatment programme in Khayelitsha, South Africa	Boulle A, Van Cutsem G, Hilderbrand K, Cragg C, Abrahams M, Mathee S, Ford N, Knight L, Osler M, Myers J, Goemaere E, Coetzee D, Maartens G. Seven-year experience of a primary care antiretroviral treatment programme in Khayelitsha, South Africa. AIDS. 2010; 24(4): 563-72.	5 - Nonfatal Health Outcomes
230982	Scientific literature	Severe influenza-associated respiratory infection in high HIV prevalence setting, South Africa, 2009-2011	Cohen C, Moyes J, Tempia S, Groom M, Walaza S, Pretorius M, Dawood H, Chhagan M, Haffejee S, Variava E, Kahn K, Tshangela A, von Gottberg A, Wolter N, Cohen AL, Kgokong B, Venter M, Madhi SA. Severe influenza-associated respiratory infection in high HIV prevalence setting, South Africa, 2009-2011. Emerg Infect Dis. 2013; 19(11): 1766â€“74.	5 - Nonfatal Health Outcomes
143218	Scientific literature	Sexually transmitted diseases in South Africa	Pham-Kanter GB, Steinberg MH, Ballard RC. Sexually transmitted diseases in South Africa. Genitourin Med. 1996; 72(3): 160-71.	5 - Nonfatal Health Outcomes
139928	Scientific literature	Sexually transmitted pathogens in pregnant women in a rural South African community	O'Farrell N, Hoosen AA, Kharsany AB, van den Ende J. Sexually transmitted pathogens in pregnant women in a rural South African community. Genitourin Med. 1989; 65(4): 276-80.	5 - Nonfatal Health Outcomes
143179	Scientific literature	Socio-demographic factors and anthropometric status of 0-71-month-old children and their caregivers in rural districts of the Eastern Cape and KwaZulu-Natal provinces of South Africa	Smuts C, Faber M, Schoeman S, Laubscher J, Oelofse A, Benade A, Dhansay M. Socio-demographic factors and anthropometric status of 0-71-month-old children and their caregivers in rural districts of the Eastern Cape and KwaZulu-Natal provinces of South Africa. S Afr J Clin Nutr. 2008; 21(3): 117-24.	5 - Nonfatal Health Outcomes
408051	Scientific literature	Streptococcus pneumoniae Serotypes and Mortality in Adults and	Cohen C, Naidoo N, Meiring S, de Gouveia L, von Mollendorf C, Walaza S, Naicker P, Madhi SA, Feldman C, Klugman KP, Dawood	5 - Nonfatal Health

		Adolescents in South Africa: Analysis of National Surveillance Data, 2003 - 2008	H, von Gottberg A, GERMS-SA. Streptococcus pneumoniae Serotypes and Mortality in Adults and Adolescents in South Africa: Analysis of National Surveillance Data, 2003 - 2008. PLoS One. 2015; 10(10): 1-19.	Outcomes
342293	Scientific literature	Substance use Among Rural Secondary School Pupils in the Northern Province, South Africa	Lily Peltzer K. Substance use Among Rural Secondary School Pupils in the Northern Province, South Africa. J Psychol Afr. 1999; 9(1): 58â€“65.	5 - Nonfatal Health Outcomes
342295	Scientific literature	Substance Use Among Urban Secondary School Pupils in the Northern Province, South Africa	Peltzer K, Cherlan M, Cherlan L. Substance Use Among Urban Secondary School Pupils in the Northern Province, South Africa. South Afr J Child Adolesc Ment Health. 1999; 11(1): 49â€“55.	5 - Nonfatal Health Outcomes
341239	Scientific literature	Substance use by adolescents in Cape Town: prevalence and correlates	Flisher AJ, Parry CDH, Evans J, Muller M, Lombard C. Substance use by adolescents in Cape Town: prevalence and correlates. J Adolesc Health. 2003; 32(1): 58â€“65.	5 - Nonfatal Health Outcomes
119178	Scientific literature	Surveillance of primary dentition caries in Germiston, South Africa, 1981-97	Cleaton-Jones P, Williams S, Fatti P. Surveillance of primary dentition caries in Germiston, South Africa, 1981-97. Community Dent Oral Epidemiol. 2000; 28(4): 267-73.	5 - Nonfatal Health Outcomes
213399	Scientific literature	Survival from HIV-1 seroconversion in Southern Africa: a retrospective cohort study in nearly 2000 gold-miners over 10 years of follow-up	Glynn JR, Sonnenberg P, Nelson G, Bester A, Shearer S, Murray J. Survival from HIV-1 seroconversion in Southern Africa: a retrospective cohort study in nearly 2000 gold-miners over 10 years of follow-up. AIDS. 2007; 21(5): 625-32.	5 - Nonfatal Health Outcomes
138327	Scientific literature	Syphilis in pregnancy--prevalence at different levels of health care in Durban	Devjee J, Moodley J, Singh M. Syphilis in pregnancy--prevalence at different levels of health care in Durban. S Afr Med J. 2006; 96(11): 1182-4.	5 - Nonfatal Health Outcomes
143221	Scientific literature	Syphilis in pregnant patients and their offspring	Bam RH,Â CronjÃ© HS,Â Muir A,Â Griessel DJ,Â Hoek BB. Syphilis in pregnant patients and their offspring. Int J Gynaecol Obstet. 1994; 44(2): 113-8.	5 - Nonfatal Health Outcomes
127903	Scientific literature	Systemic hypothermia after neonatal encephalopathy: outcomes of neo.nEURO.network RCT	Simbruner G, Mittal RA, Rohlmann F, Muche R, neo.nEURO.network Trial Participants. Systemic hypothermia after neonatal encephalopathy: outcomes of neo.nEURO.network RCT. Pediatrics. 2010; 126(4): e771-8.	5 - Nonfatal Health Outcomes
214901	Scientific literature	Temporal changes in programme outcomes among adult patients initiating antiretroviral therapy across South Africa, 2002-2007	Cornell M, Grimsrud A, Fairall L, Fox MP, van Cutsem G, Giddy J, Wood R, Prozesky H, Mohapi L, Graber C, Egger M, Boulle A, Myer L, International Epidemiologic Databases to Evaluate AIDS Southern Africa (IeDEA-SA) Collaboration. Temporal changes in programme outcomes among adult patients initiating antiretroviral therapy across South Africa, 2002-2007. AIDS. 2010; 24(14): 2263-70.	5 - Nonfatal Health Outcomes
137539	Scientific literature	The causes, treatment, and outcome of acute heart failure in 1006 Africans from 9 countries	Damasceno A, Mayosi BM, Sani M, Ogah OS, Mondo C, Ojji D, Dzudie A, Kouam CK, Suliman A, Schrueder N, Yonga G, Ba SA, Maru F, Alemayehu B, Edwards C, Davison BA, Cotter G, Sliwa K. The causes, treatment, and outcome of acute heart failure in 1006 Africans from 9 countries. Arch Intern Med. 2012; 172(18): 1386-94.	5 - Nonfatal Health Outcomes
141304	Scientific literature	The changing prevalence of asthma, allergic rhinitis and atopic eczema in African adolescents from 1995 to 2002	Zar HJ, Ehrlich RI, Workman L, Weinberg EG. The changing prevalence of asthma, allergic rhinitis and atopic eczema in African adolescents from 1995 to 2002. Pediatr Allergy Immunol. 2007; 18(7): 560-5.	5 - Nonfatal Health Outcomes
350587	Scientific literature	The dangers of rationing dialysis treatment: The dilemma facing a developing country	Moosa MR, Kidd M. The dangers of rationing dialysis treatment: The dilemma facing a developing country. Kidney Int. 2006; 70(6): 1107â€“14.	5 - Nonfatal Health Outcomes
283126	Scientific literature	The distribution of helminth infections along the coastal plain of Kwazulu-Natal province, South Africa	Appleton CC, Maurihungirire M, Gouws E. The distribution of helminth infections along the coastal plain of Kwazulu-Natal province, South Africa. Ann Trop Med Parasitol. 1999; 93(8): 859-68.	5 - Nonfatal Health Outcomes
221946	Scientific literature	The effect of the vaginal diaphragm and lubricant gel on acquisition of HSV-2	De Bruyn G, Shiboski S, van der Straten A, Blanchard K, Chipato T, Ramjee G, Montgomery E, Padian N, MIRA Team. The effect of the vaginal diaphragm and lubricant gel on acquisition of HSV-2. Sex Transm Infect. 2011; 87(4): 301â€“5.	5 - Nonfatal Health Outcomes
140084	Scientific literature	The epidemiology of fetal alcohol syndrome and partial FAS in a South African community	May PA, Gossage JP, Marais A-S, Adnams CM, Hoyme HE, Jones KL, Robinson LK, Khaole NCO, Snell C, Kalberg WO, Hendricks L, Brooke L, Stellavato C, Viljoen DL. The epidemiology of fetal alcohol syndrome and partial FAS in a South African community. Drug Alcohol Depend. 2007; 88(2-3): 259-71.	5 - Nonfatal Health Outcomes
126026	Scientific literature	The epidemiology of major depression in South Africa: results from the South African stress and health study	Tomlinson M, Grimsrud AT, Stein DJ, Williams DR, Myer L. The epidemiology of major depression in South Africa: results from the South African stress and health study. S Afr Med J. 2009; 99(5 Pt 2): 367-73.	5 - Nonfatal Health Outcomes
294028	Scientific literature	The Epidemiology of Schistosomiasis among Zulu Children in a Rural District in South Africa: Determining	Taylor M. The Epidemiology of Schistosomiasis among Zulu Children in a Rural District in South Africa: Determining Appropriate Community-Based Diagnostic Tools. South Afr J	5 - Nonfatal Health Outcomes

		Appropriate Community-Based Diagnostic Tools	Epidemiol Infect. 2004; 19: 90-95.	
311726	Scientific literature	The feasibility of malaria elimination in South Africa	Maharaj Rajendra, Morris Natashia, Seocharan Ishen, Kruger Philip, Moonasar Devanand, Mabuza Aaron, Raswiswi Eric, Raman Jaishree. The feasibility of malaria elimination in South Africa. Malar J. 2012; 11(1): 423.	5 - Nonfatal Health Outcomes
144316	Scientific literature	The global status of schistosomiasis and its control	Chitsulo L, Engels D, Montresor A, Savioli L. The global status of schistosomiasis and its control. Acta Trop. 2000; 77(1): 41-51.	5 - Nonfatal Health Outcomes
127596	Scientific literature	The impact of incident and prevalent herpes simplex virus-2 infection on the incidence of HIV-1 infection among commercial sex workers in South Africa	Ramjee G, Williams B, Gouws E, Van Dyck E, De Deken B, Karim SA. The impact of incident and prevalent herpes simplex virus-2 infection on the incidence of HIV-1 infection among commercial sex workers in South Africa. J Acquir Immune Defic Syndr. 2005; 39(3): 333-9.	5 - Nonfatal Health Outcomes
214554	Scientific literature	The interplay between CD4 cell count, viral load suppression and duration of antiretroviral therapy on mortality in a resource-limited setting	Brennan AT, Maskew M, Sanne I, Fox MP. The interplay between CD4 cell count, viral load suppression and duration of antiretroviral therapy on mortality in a resource-limited setting. Trop Med Int Health. 2013; 18(5): 619-31.	5 - Nonfatal Health Outcomes
116725	Scientific literature	The molecular epidemiology of rotavirus-associated gastro-enteritis in the Transkei, southern Africa	Griffiths FH, Steele AD, Alexander JJ. The molecular epidemiology of rotavirus-associated gastro-enteritis in the Transkei, southern Africa. Ann Trop Paediatr. 1992; 12(3): 259-64.	5 - Nonfatal Health Outcomes
144167	Scientific literature	The nutritional status of a rural community in KwaZulu-Natal, South Africa: the Ndunakazi project	Oelofse A, Faber M, BenadÃ© JG, BenadÃ© AJ, Kenoyer DG. The nutritional status of a rural community in KwaZulu-Natal, South Africa: the Ndunakazi project. Cent Afr J Med. 1999; 45(1): 14-9.	5 - Nonfatal Health Outcomes
112860	Scientific literature	The prevalence and identification of risk factors for NIDDM in urban Africans in Cape Town, South Africa	Levitt NS, Katzenellenbogen JM, Bradshaw D, Hoffman MN, Bonnici F. The prevalence and identification of risk factors for NIDDM in urban Africans in Cape Town, South Africa. Diabetes Care. 1993; 16(4): 601-7.	5 - Nonfatal Health Outcomes
420038	Scientific literature	The prevalence and transmission of hepatitis B virus infection in urban, rural and institutionalized black children of Natal/KwaZulu, South Africa	Abdool Karim SS, Coovadia HM, Windsor IM, Thejpal R, van den Ende J, Fouche A. The prevalence and transmission of hepatitis B virus infection in urban, rural and institutionalized black children of Natal/KwaZulu, South Africa. Int J Epidemiol. 1988; 17(1): 16873.	5 - Nonfatal Health Outcomes
144959	Scientific literature	The prevalence of diabetes mellitus and impaired glucose tolerance in a group of urban South African blacks	Omar MA, Seedat MA, Motala AA, Dyer RB, Becker P. The prevalence of diabetes mellitus and impaired glucose tolerance in a group of urban South African blacks. S Afr Med J. 1993; 83(9): 641-3.	5 - Nonfatal Health Outcomes
286347	Scientific literature	The prevalence of hearing impairment within the Cape Town Metropolitan area	Ramma L, Sebothoma B. The prevalence of hearing impairment within the Cape Town Metropolitan area. S Afr J Commun Disord. 2016; 63(1): 1-10.	5 - Nonfatal Health Outcomes
403878	Scientific literature	The prevalence of osteoarthrosis in a rural African community	Brighton SW, de la Harpe AL, Van Staden DA. The prevalence of osteoarthrosis in a rural African community. Br J Rheumatol. 1985; 24(4): 321-5.	5 - Nonfatal Health Outcomes
354782	Scientific literature	The prevalence of Plasmodium falciparum in sub-Saharan Africa since 1900	Snow RW, Sartorius B, Kyalo D, Maina J, Amratia P, Mundia CW, Bejon P, Noor AM. The prevalence of Plasmodium falciparum in sub-Saharan Africa since 1900. Nature. 2017; 550(7677): 515â€“8.	5 - Nonfatal Health Outcomes
127448	Scientific literature	The prevalence of primary angle closure glaucoma and open angle glaucoma in Mamre, western Cape, South Africa	Salmon JF, Mermoud A, Ivey A, Swanevelder SA, Hoffman M. The prevalence of primary angle closure glaucoma and open angle glaucoma in Mamre, western Cape, South Africa. Arch Ophthalmol. 1993; 111(9): 1263-9.	5 - Nonfatal Health Outcomes
294027	Scientific literature	The prevalence of Schistosoma haematobium in school children in Bizana District of Transkei	Mayanja FJLB, Edginton ME. The prevalence of Schistosoma haematobium in school children in Bizana District of Transkei. South Afr J Epidemiol Infect. 1992; 7: 20-21.	5 - Nonfatal Health Outcomes
222429	Scientific literature	The relationship between age of coital debut and HIV seroprevalence among women in Durban, South Africa: a cohort study	Wand H, Ramjee G. The relationship between age of coital debut and HIV seroprevalence among women in Durban, South Africa: a cohort study. BMJ Open. 2012; e000285.	5 - Nonfatal Health Outcomes
335324	Scientific literature	The Relationship between Physical Activity and Plasma Glucose Level amongst Ellisras Rural Young Adult Males and Females: Ellisras Longitudinal Study	Matshipi M, Monyeki KD, Kemper H. The Relationship between Physical Activity and Plasma Glucose Level amongst Ellisras Rural Young Adult Males and Females: Ellisras Longitudinal Study. Int J Environ Res Public Health. 2017; 14(2).	5 - Nonfatal Health Outcomes
283258	Scientific literature	The relative roles of hepatitis B and C viruses in the etiology of hepatocellular carcinoma in southern African blacks	Kew MC, Yu MC, Kedda MA, Coppin A, Sarkin A, Hodkinson J. The relative roles of hepatitis B and C viruses in the etiology of hepatocellular carcinoma in southern African blacks. Gastroenterology. 1997; 112(1): 184-7.	5 - Nonfatal Health Outcomes
307896	Scientific literature	The Tuberculin Skin Test versus QuantiFERON TB GoldÂ® in Predicting Tuberculosis Disease in an	Mahomed H, Hawkridge T, Verver S, Abrahams D, Geiter L, Hatherill M, Ehrlich R, Hanekom WA, Hussey GD. The Tuberculin Skin Test versus QuantiFERON TB GoldÂ® in Predicting	5 - Nonfatal Health Outcomes

		Adolescent Cohort Study in South Africa	Tuberculosis Disease in an Adolescent Cohort Study in South Africa. PLoS One. 2011; 6(3): e17984.	
127934	Scientific literature	The value of a scoring system for hypoxic ischaemic encephalopathy in predicting neurodevelopmental outcome	Thompson CM, Puterman AS, Linley LL, Hann FM, van der Elst CW, Molteno CD, Malan AF. The value of a scoring system for hypoxic ischaemic encephalopathy in predicting neurodevelopmental outcome. Acta Paediatr. 1997; 86(7): 757-61.	5 - Nonfatal Health Outcomes
267694	Scientific literature	Treatment outcomes of HIV-positive patients on first-line antiretroviral therapy in private versus public HIV clinics in Johannesburg, South Africa	Moyo F, Chasela C, Brennan AT, Ebrahim O, Sanne IM, Long L, Evans D. Treatment outcomes of HIV-positive patients on first-line antiretroviral therapy in private versus public HIV clinics in Johannesburg, South Africa. Clin Epidemiol. 2016; 8: 37â€“47.	5 - Nonfatal Health Outcomes
141078	Scientific literature	TreatmentÂ ofÂ maternalÂ syphilisÂ inÂ ruralÂ South Africa: effect of multiple doses of benzathine penicillin on pregnancy loss	Myer L,Â Abdool Karim SS,Â Lombard C,Â Wilkinson D. TreatmentÂ ofÂ maternalÂ syphilisÂ inÂ ruralÂ South Africa: effect of multiple doses of benzathine penicillin on pregnancy loss. Trop Med Int Health. 2004; 9(11): 1216-21.	5 - Nonfatal Health Outcomes
335001	Scientific literature	Twelve-year mortality in adults initiating antiretroviral therapy in South Africa	Cornell M, Johnson LF, Wood R, Tanser F, Fox MP, Prozesky H, Schomaker M, Egger M, Davies MA, Boulle A, International Epidemiology Databases to Evaluate AIDS-Southern Africa collaboration. Twelve-year mortality in adults initiating antiretroviral therapy in South Africa. J Int AIDS Soc. 2017; 20(1): 21902.	5 - Nonfatal Health Outcomes
229135	Scientific literature	Universal prevention is associated with lower prevalence of fetal alcohol spectrum disorders in Northern Cape, South Africa: a multicentre before-after study	Chersich MF, Urban M, Olivier L, Davies LA, Chetty C, Viljoen D. Universal prevention is associated with lower prevalence of fetal alcohol spectrum disorders in Northern Cape, South Africa: a multicentre before-after study. Alcohol Alcohol. 2012; 47(1): 67â€“74.	5 - Nonfatal Health Outcomes
139929	Scientific literature	Urogenital tract infections in pregnancy at King Edward VIII Hospital, Durban, South Africa	Dietrich M, Hoosen AA, Moodley J, Moodley S. Urogenital tract infections in pregnancy at King Edward VIII Hospital, Durban, South Africa. Genitourin Med. 1992; 68(1): 39-41.	5 - Nonfatal Health Outcomes
121211	Scientific literature	Vaginal tampons as specimen collection device for the molecular diagnosis of non-ulcerative sexually transmitted infections in antenatal clinic attendees	Sturm PDJ, Connolly C, Khan N, Ebrahim S, Sturm AW. Vaginal tampons as specimen collection device for the molecular diagnosis of non-ulcerative sexually transmitted infections in antenatal clinic attendees. Int J STD AIDS. 2004; 15(2): 94-8.	5 - Nonfatal Health Outcomes
409989	Scientific literature	Validation of the 10-item Centre for Epidemiological Studies Depression Scale (CES-D-10) in Zulu, Xhosa and Afrikaans populations in South Africa	Baron EC, Davies T, Lund C. Validation of the 10-item Centre for Epidemiological Studies Depression Scale (CES-D-10) in Zulu, Xhosa and Afrikaans populations in South Africa. BMC Psychiatry. 2017; 17(6).	5 - Nonfatal Health Outcomes
141303	Scientific literature	Validation of the U.K. Working Party diagnostic criteria for atopic eczema in a Xhosa-speaking African population	Chalmers DA, Todd G, Saxe N, Milne JT, Tolosana S, Ngcelwane PN, Hlaba BN, Mngomeni LN, Nonxuba TG, Williams HC. Validation of the U.K. Working Party diagnostic criteria for atopic eczema in a Xhosa-speaking African population. Br J Dermatol. 2007; 156(1): 111-6.	5 - Nonfatal Health Outcomes
145692	Scientific literature	Viral hepatitis in South African healthcare workers at increased risk of occupational exposure to blood-borne viruses	Vardas E, Ross MH, Sharp G, McAnerney J, Sim J. Viral hepatitis in South African healthcare workers at increased risk of occupational exposure to blood-borne viruses. J Hosp Infect. 2002; 50(1): 6-12.	5 - Nonfatal Health Outcomes
214542	Scientific literature	Virologic, immunologic, and clinical response to highly active antiretroviral therapy: the gender issue revisited	Moore AL, Kirk O, Johnson AM, Katlama C, Blaxhult A, Dietrich M, Colebunders R, Chiesi A, Lungren JD, Phillips AN, EuroSIDA group. Virologic, immunologic, and clinical response to highly active antiretroviral therapy: the gender issue revisited. J Acquir Immune Defic Syndr. 2003; 32(4): 452-61.	5 - Nonfatal Health Outcomes
161345	Scientific literature	What measured blood loss tells us about postpartum bleeding: a systematic review	Sloan N, Durocher J, Aldrich T, Blum J, Winikoff B. What measured blood loss tells us about postpartum bleeding: a systematic review. BJOG. 2010; 117(7): 788-800.	5 - Nonfatal Health Outcomes
111334	Scientific literature	Worldwide variations in the prevalence of asthma symptoms: the International Study of Asthma and Allergies in Childhood (ISAAC)	Worldwide variations in the prevalence of asthma symptoms: the International Study of Asthma and Allergies in Childhood (ISAAC). Eur Respir J. 1998; 12(2): 315-35.	5 - Nonfatal Health Outcomes
270670	Survey	Global Atlas of Helminth Infections - Soil Transmitted Helminths	London School of Hygiene and Tropical Medicine. Global Atlas of Helminth Infections - Soil Transmitted Helminths. London, United Kingdom: London School of Hygiene and Tropical Medicine.	5 - Nonfatal Health Outcomes
139748	Survey	Global Burden of Disease 2010 Expert Group Working Document on Sense and Hearing Disorders	Global Burden of Disease 2010 Expert Group Working Document on Sense and Hearing Disorders.	5 - Nonfatal Health Outcomes
135825	Survey	South Africa - Agincourt Integrated Family Survey 2002	Case, A. 2003. Agincourt Integrated Family Survey 2002. [dataset] Version 1. Cape Town: DataFirst [distributor].	5 - Nonfatal Health Outcomes
135826	Survey	South Africa - Agincourt Integrated Family Survey 2004	Case, A. 2005. Agincourt Integrated Family Survey 2004. [dataset] Version 1. Cape Town: DataFirst [distributor].	5 - Nonfatal Health Outcomes

143217	Survey	South Africa - Eastern Cape HIV and Syphilis Antenatal Sero-Surveillance Survey 2007	Department of Health (South Africa), Eastern Cape Department of Health (South Africa). South Africa - Eastern Cape HIV and Syphilis Antenatal Sero-Surveillance Survey 2007.	5 - Nonfatal Health Outcomes
11928	Survey	South Africa - KwaZulu and Natal Income Dynamics Study 1993	International Food Policy Research Institute (IFPRI), University of Natal, University of Wisconsin, Southern Africa Labour Development Research Unit (SALDRU), School of Economics, University of Cape Town. South Africa KwaZulu-Natal Income Dynamics Study 1993. Durban, South Africa: University of Natal.	5 - Nonfatal Health Outcomes
261887	Survey	South Africa - Venda Malnutrition and Enteric Disease Study 2009-2014	Fogarty International Center, National Institutes of Health (NIH), Foundation for the National Institutes of Health (FNIH), University of Venda. South Africa - Venda Malnutrition and Enteric Disease Study 2009-2014.	5 - Nonfatal Health Outcomes
342458	Survey	South Africa - Western Cape Survey on Substance Use, Risk Behavior and Mental Health Among Grade 8-10 Learners in Schools 2011	Department of Social Development, Western Cape Government (South Africa), South African Medical Research Council, United Nations Office on Drugs and Crime (UNODC). South Africa - Western Cape Survey on Substance Use, Risk Behavior and Mental Health Among Grade 8-10 Learners in Schools 2011.	5 - Nonfatal Health Outcomes
141829	Survey	South Africa Anthropometric Survey in Primary Schools 1994	Department of Health (South Africa). South Africa Anthropometric Survey in Primary Schools 1994.	5 - Nonfatal Health Outcomes
139810	Survey	South Africa Anthropometric, Vitamin A, Iron and Immunisation Coverage Status in Children Aged 6 to 71 Months 1994	South African Vitamin A Consultative Group (SAVACG). South Africa Anthropometric, Vitamin A, Iron and Immunisation Coverage Status in Children Aged 6 to 71 Months 1994.	5 - Nonfatal Health Outcomes
20796	Survey	South Africa Demographic and Health Survey 1998	Department of Health (South Africa), Macro International, Inc, South African Medical Research Council. South Africa Demographic and Health Survey 1998. Fairfax, United States of America: ICF International.	5 - Nonfatal Health Outcomes
20798	Survey	South Africa Demographic and Health Survey 2003-2004	Department of Health (South Africa), Macro International, Inc, South African Medical Research Council. South Africa Demographic and Health Survey 2003-2004.	5 - Nonfatal Health Outcomes
157064	Survey	South Africa Demographic and Health Survey 2016	Department of Health (South Africa), ICF International, South African Medical Research Council, Statistics South Africa. South Africa Demographic and Health Survey 2016. Fairfax, United States of America: ICF International, 2019.	5 - Nonfatal Health Outcomes
115481	Survey	South Africa General Household Survey 2002	Statistics South Africa. South Africa General Household Survey 2002. Pretoria, South Africa: Statistics South Africa.	5 - Nonfatal Health Outcomes
11788	Survey	South Africa General Household Survey 2004	Statistics South Africa. South Africa General Household Survey 2004. Pretoria, South Africa: Statistics South Africa.	5 - Nonfatal Health Outcomes
11789	Survey	South Africa General Household Survey 2005	Statistics South Africa. South Africa General Household Survey 2005. Pretoria, South Africa: Statistics South Africa.	5 - Nonfatal Health Outcomes
115486	Survey	South Africa General Household Survey 2006	Statistics South Africa. South Africa General Household Survey 2006. Pretoria, South Africa: Statistics South Africa.	5 - Nonfatal Health Outcomes
11790	Survey	South Africa General Household Survey 2007	Statistics South Africa. South Africa General Household Survey 2007. Cape Town, South Africa: DataFirst.	5 - Nonfatal Health Outcomes
115488	Survey	South Africa General Household Survey 2008	Statistics South Africa. South Africa General Household Survey 2008. Pretoria, South Africa: Statistics South Africa.	5 - Nonfatal Health Outcomes
115489	Survey	South Africa General Household Survey 2009	Statistics South Africa. South Africa General Household Survey 2009. Pretoria, South Africa: Statistics South Africa.	5 - Nonfatal Health Outcomes
115490	Survey	South Africa General Household Survey 2010	Statistics South Africa. South Africa General Household Survey 2010. Pretoria, South Africa: Statistics South Africa.	5 - Nonfatal Health Outcomes
115491	Survey	South Africa General Household Survey 2011	Statistics South Africa. South Africa General Household Survey 2011. Cape Town, South Africa: DataFirst.	5 - Nonfatal Health Outcomes
135534	Survey	South Africa General Household Survey 2012	Statistics South Africa. South Africa General Household Survey 2012. Pretoria, South Africa: Statistics South Africa, 2013.	5 - Nonfatal Health Outcomes
238483	Survey	South Africa General Household Survey 2013	Statistics South Africa. General Household Survey 2013 [dataset]. Version 1. Pretoria. Statistics South Africa [producer], 2014. Cape Town. DataFirst [distributor], 2014.10.25828/a704-wm31	5 - Nonfatal Health Outcomes

317089	Survey	South Africa General Household Survey 2016	Statistics South Africa. South Africa General Household Survey 2016. Cape Town, South Africa: DataFirst, 2015.	5 - Nonfatal Health Outcomes
393799	Survey	South Africa General Household Survey 2017	Statistics South Africa. South Africa General Household Survey 2017. Cape Town, South Africa: DataFirst, 2018.	5 - Nonfatal Health Outcomes
106158	Survey	South Africa Integrated Family Survey 1999	Southern Africa Labour Development Research Unit (SALDRU), University of Cape Town. South Africa Integrated Family Survey 1999.	5 - Nonfatal Health Outcomes
12013	Survey	South Africa KwaZulu-Natal Income Dynamics Study 1998	International Food Policy Research Institute (IFPRI), University of Natal, University of Wisconsin, Data Research Africa (DRA), Policy and Praxis, Southern Africa Labour Development Research Unit (SALDRU), School of Economics, University of Cape Town. South Africa KwaZulu-Natal Income Dynamics Study 1998. Durban, South Africa: University of Natal.	5 - Nonfatal Health Outcomes
31142	Survey	South Africa KwaZulu-Natal Income Dynamics Study 2004	University of Kwazulu-Natal, University of Wisconsin, London School of Hygiene and Tropical Medicine, International Food Policy Research Institute (IFPRI), Department of Social Development (South Africa), Norwegian Institute for Urban and Regional Research (NIBR). South Africa KwaZulu-Natal Income Dynamics Study 2004. Durban, South Africa: University of Kwazulu-Natal.	5 - Nonfatal Health Outcomes
317239	Survey	South Africa Living Conditions Survey 2014-2015	Statistics South Africa. South Africa Living Conditions Survey 2014-2015. Pretoria, South Africa: Statistics South Africa.	5 - Nonfatal Health Outcomes
12101	Survey	South Africa Living Standards Measurement Study 1993	Southern Africa Labour Development Research Unit (SALDRU), University of Cape Town, World Bank. South Africa Living Standards Measurement Study 1993. Washington DC, United States of America: World Bank.	5 - Nonfatal Health Outcomes
143233	Survey	South Africa National Antenatal Sentinel HIV and Syphilis Prevalence Survey 2000	Department of Health (South Africa). South Africa National Antenatal Sentinel HIV and Syphilis Prevalence Survey 2000.	5 - Nonfatal Health Outcomes
356401	Survey	South Africa National Antenatal Sentinel HIV and Syphilis Prevalence Survey 2008	Department of Health (South Africa). South Africa National Antenatal Sentinel HIV and Syphilis Prevalence Survey 2008.	5 - Nonfatal Health Outcomes
261367	Survey	South Africa National Food Consumption Survey - Fortification Baseline 2005	Department of Health (South Africa), North-West University, South African Medical Research Council, Stellenbosch University, University of Cape Town, University of Kwazulu-Natal, University of Limpopo, University of Pretoria, University of the Free State, University of the Western Cape. South Africa National Food Consumption Survey - Fortification Baseline 2005.	5 - Nonfatal Health Outcomes
155628	Survey	South Africa National Health and Nutrition Examination Survey 2012	Human Sciences Research Council, South African Medical Research Council. South Africa National Health and Nutrition Examination Survey 2012.	5 - Nonfatal Health Outcomes
150187	Survey	South Africa National HIV and Syphillis Antenatal Sero-Prevalence Survey 2002	Department of Health (South Africa). South Africa National HIV and Syphillis Antenatal Sero-Prevalence Survey 2002.	5 - Nonfatal Health Outcomes
313076	Survey	South Africa National HIV Prevalence, Incidence, and Behavior Survey 2011-2012	Centers for Disease Control and Prevention (CDC), Global Clinical and Viral Laboratory (South Africa), Human Sciences Research Council, National Institute for Communicable Diseases (South Africa), South African Medical Research Council, University of Cape Town. South Africa National HIV Prevalence, Incidence, and Behavior Survey 2011-2012. Pretoria, South Africa: Human Sciences Research Council, 2016.	5 - Nonfatal Health Outcomes
313074	Survey	South Africa National HIV Prevalence, Incidence, Behavior and Communication Survey 2004-2005	Center for AIDS Development, Research and Evaluation (CADRE) (South Africa), Centers for Disease Control and Prevention (CDC), Global Clinical and Viral Laboratory (South Africa), Human Sciences Research Council, Maphume Research Services, National Institute for Communicable Diseases (South Africa). South Africa National HIV Prevalence, Incidence, Behavior and Communication Survey 2004-2005. Pretoria, South Africa: Human Sciences Research Council, 2011.	5 - Nonfatal Health Outcomes
228102	Survey	South Africa National HIV Prevalence, Incidence, Behavior and Communication Survey 2008-2009	Center for AIDS Development, Research and Evaluation (CADRE) (South Africa), Geospace International (South Africa), Global Clinical and Viral Laboratory (South Africa), Human Sciences Research Council, Maphume Research Services, National Institute for Communicable Diseases (South Africa), South African Medical Research Council. South Africa National HIV Prevalence, Incidence, Behavior and Communication Survey 2008-2009. Pretoria, South Africa: Human Sciences Research Council, 2014.	5 - Nonfatal Health Outcomes

357200	Survey	South Africa National HIV Prevalence, Incidence, Behavior and Communication Survey 2017	Centers for Disease Control and Prevention (CDC), Human Sciences Research Council, National Institute for Communicable Diseases (South Africa), South African Medical Research Council. South Africa National HIV Prevalence, Incidence, Behavior and Communication Survey 2017.	5 - Nonfatal Health Outcomes
27885	Survey	South Africa National Income Dynamics Study - Wave 1 2008	University of Cape Town, Southern Africa Labour and Development Research Unit. National Income Dynamics Study (NIDS) Wave 1 [computer files]. Cape Town: Southern Africa Labour and Development Research Unit [producer], 2009. Cape Town: DataFirst [distributor], 2009	5 - Nonfatal Health Outcomes
133731	Survey	South Africa National Income Dynamics Study - Wave 2 2010-2011	Southern Africa Labour and Development Research Unit. National Income Dynamics Study 2010-2011, Wave 2. Version 1.0. Cape Town: Southern Africa Labour and Development Research Unit [producer], 2012. Cape Town: DataFirst [distributor], 2013.	5 - Nonfatal Health Outcomes
133732	Survey	South Africa National Income Dynamics Study - Wave 3 2012	Southern Africa Labour and Development Research Unit. National Income Dynamics Study 2012, Wave 3 [dataset]. Version 1.2. Cape Town: Southern Africa Labour and Development Research Unit [producer], 2013. Cape Town: DataFirst [distributor], 2013	5 - Nonfatal Health Outcomes
265153	Survey	South Africa National Income Dynamics Study - Wave 4 2014-2015	Southern Africa Labour and Development Research Unit. National Income Dynamics Study 2014 - 2015, Wave 4 [dataset]. Version 1.1. Cape Town: Southern Africa Labour and Development Research Unit [producer], 2016. Cape Town: DataFirst [distributor], 2016. Pretoria: Department of Planning Monitoring and Evaluation [commissioner], 2014	5 - Nonfatal Health Outcomes
257511	Survey	South Africa National Youth Risk Behaviour Survey 2002	Centers for Disease Control and Prevention (CDC), Department of Health (South Africa), National Department of Education (South Africa), South African Medical Research Council. South Africa National Youth Risk Behaviour Survey 2002.	5 - Nonfatal Health Outcomes
419800	Survey	South Africa Rapid Assessment of Avoidable Blindness Survey 2010	International Centre for Eye Health (ICEH). South Africa Rapid Assessment of Avoidable Blindness Survey 2010. Grootebroek, Netherlands: RAAB Repository, 2014.	5 - Nonfatal Health Outcomes
111488	Survey	South Africa WHO Study on Global AGEing and Adult Health 2007-2008	Department of Health (South Africa), Human Sciences Research Council, World Health Organization (WHO). South Africa WHO Study on Global AGEing and Adult Health 2007-2008. Geneva, Switzerland: World Health Organization (WHO).	5 - Nonfatal Health Outcomes
21864	Survey	South Africa World Health Survey 2002-2003	World Health Organization (WHO). South Africa World Health Survey 2002-2003. Geneva, Switzerland: World Health Organization (WHO), 2005.	5 - Nonfatal Health Outcomes
43713	Survey	WHO Global Database on Vitamin A Deficiency	World Health Organization (WHO). WHO Global Database on Vitamin A Deficiency. Geneva, Switzerland: World Health Organization (WHO).	5 - Nonfatal Health Outcomes
292791	Survey	WHO Global Project on Anti-Tuberculosis Drug Resistance Surveillance Data 1988-2015	World Health Organization (WHO). WHO Global Project on Anti-Tuberculosis Drug Resistance Surveillance Data 1988-2015.	5 - Nonfatal Health Outcomes
162652	Survey	Zambia Access, Bottlenecks, Costs, and Equity Project 2011-2012	Central Statistical Office (Zambia), Churches Health Association of Zambia (CHAZ), Clinton Health Access Initiative (CHAI), Institute for Health Metrics and Evaluation (IHME), Ministry of Health (Zambia), University of Zambia. Access, Bottlenecks, Costs, and Equity (ABCE) project in Zambia, 2011-2012. Seattle, United States: Institute for Health Metrics and Evaluation (IHME), 2015.	5 - Nonfatal Health Outcomes
107077	Vital registration	South Africa Vital Registration - Causes of Death 1997-2005	Department of Home Affairs (South Africa), Statistics South Africa. South Africa Vital Registration - Causes of Death 1997-2005. Pretoria, South Africa: Statistics South Africa.	5 - Nonfatal Health Outcomes
151816	Vital registration	South Africa Vital Registration - Causes of Death 2006	Department of Home Affairs (South Africa), Statistics South Africa. South Africa Vital Registration - Causes of Death 2006. Pretoria, South Africa: Statistics South Africa.	5 - Nonfatal Health Outcomes
151817	Vital registration	South Africa Vital Registration - Causes of Death 2007	Department of Home Affairs (South Africa), Statistics South Africa. South Africa Vital Registration - Causes of Death 2007. Pretoria, South Africa: Statistics South Africa.	5 - Nonfatal Health Outcomes
106583	Vital registration	South Africa Vital Registration - Causes of Death 2008	Department of Home Affairs (South Africa), Statistics South Africa. South Africa Vital Registration - Causes of Death 2008. Pretoria, South Africa: Statistics South Africa.	5 - Nonfatal Health Outcomes
106584	Vital registration	South Africa Vital Registration - Causes of Death 2009	Department of Home Affairs (South Africa), Statistics South Africa. South Africa Vital Registration - Causes of Death 2009. Pretoria, South Africa: Statistics South Africa.	5 - Nonfatal Health Outcomes
151818	Vital registration	South Africa Vital Registration - Causes of Death 2010	Department of Home Affairs (South Africa), Statistics South Africa. South Africa Vital Registration - Causes of Death 2010. Pretoria, South Africa: Statistics South Africa.	5 - Nonfatal Health Outcomes

151819	Vital registration	South Africa Vital Registration - Causes of Death 2011	Department of Home Affairs (South Africa), Statistics South Africa. South Africa Vital Registration - Causes of Death 2011. Pretoria, South Africa: Statistics South Africa.	5 - Nonfatal Health Outcomes
204016	Vital registration	South Africa Vital Registration - Causes of Death 2012	Department of Home Affairs (South Africa), Statistics South Africa. South Africa Vital Registration - Causes of Death 2012. Pretoria, South Africa: Statistics South Africa.	5 - Nonfatal Health Outcomes
204017	Vital registration	South Africa Vital Registration - Causes of Death 2013	Department of Home Affairs (South Africa), Statistics South Africa. South Africa Vital Registration - Causes of Death 2013. Pretoria, South Africa: Statistics South Africa.	5 - Nonfatal Health Outcomes
267740	Vital registration	South Africa Vital Registration - Causes of Death 2014	Department of Home Affairs (South Africa), Statistics South Africa. South Africa Vital Registration - Causes of Death 2014. Pretoria, South Africa: Statistics South Africa.	5 - Nonfatal Health Outcomes
312271	Vital registration	South Africa Vital Registration - Causes of Death 2015	Department of Home Affairs (South Africa), Statistics South Africa. South Africa Vital Registration - Causes of Death 2015. Pretoria, South Africa: Statistics South Africa.	5 - Nonfatal Health Outcomes
399147	Vital registration	South Africa Vital Registration - Causes of Death 2016	Department of Home Affairs (South Africa), Statistics South Africa. South Africa Vital Registration - Causes of Death 2016. Pretoria, South Africa: Statistics South Africa, 2019.	5 - Nonfatal Health Outcomes
422985	Administrative data	Euromonitor Passport - Alcoholic Drinks Statistics	Euromonitor International. Euromonitor Passport - Alcoholic Drinks Statistics . London, United Kingdom: Euromonitor International.	6 - Risk Factors
282698	Administrative data	Euromonitor Passport - Fresh Foods Market Statistics	Euromonitor International. Euromonitor Passport - Fresh Foods Market Statistics. London, United Kingdom: Euromonitor International.	6 - Risk Factors
422982	Administrative data	Euromonitor Passport - Smoking Tobacco Statistics	Euromonitor International. Euromonitor Passport - Smoking Tobacco Statistics . London, United Kingdom: Euromonitor International.	6 - Risk Factors
238501	Administrative data	FAO Supply Utilization Accounts 1961-2013	FAO Supply Utilization Accounts 1961-2013. Personal Correspondence with Dr. Josef Schmidhuber, 2016. [Unpublished].	6 - Risk Factors
244478	Administrative data	FAOSTAT Commodity Balances - Crops Primary Equivalent	Food and Agriculture Organization of the United Nations (FAO). FAOSTAT Commodity Balances - Crops Primary Equivalent. Rome, Italy: Food and Agriculture Organization of the United Nations (FAO).	6 - Risk Factors
200195	Administrative data	FAOSTAT Food Balance Sheets, October 2014	Food and Agriculture Organization of the United Nations (FAO). FAOSTAT Food Balance Sheets, October 2014. Rome, Italy: Food and Agriculture Organization of the United Nations (FAO).	6 - Risk Factors
244475	Administrative data	USDA Global Tobacco Database 1960-2005	U.S. Department of Agriculture (USDA). USDA Global Tobacco Database 1960-2005. Washington D.C. , United States: U.S. Department of Agriculture (USDA).	6 - Risk Factors
238445	Administrative data	WHO Global Health Observatory - Recorded Alcohol Per Capita Consumption 1960-1979	World Health Organization (WHO). WHO Global Health Observatory - Recorded Alcohol Per Capita Consumption 1960-1979. Geneva, Switzerland: World Health Organization (WHO).	6 - Risk Factors
238448	Administrative data	WHO Global Health Observatory - Recorded Alcohol Per Capita Consumption 1980-1999	World Health Organization (WHO). WHO Global Health Observatory - Recorded Alcohol Per Capita Consumption 1980-1999. Geneva, Switzerland: World Health Organization (WHO).	6 - Risk Factors
238452	Administrative data	WHO Global Health Observatory - Recorded Alcohol Per Capita Consumption 2000-2009 by country	World Health Organization (WHO). WHO Global Health Observatory - Recorded Alcohol Per Capita Consumption 2000-2009 by country. Geneva, Switzerland: World Health Organization (WHO).	6 - Risk Factors
43146	Census	South Africa Census 1996 - IPUMS	Central Statistical Service (South Africa), Minnesota Population Center. South Africa Census 1996 from the Integrated Public Use Microdata Series, International: [Machine-readable database]. Minneapolis: University of Minnesota.	6 - Risk Factors
43152	Census	South Africa Census 2001 - IPUMS	Statistics South Africa, Minnesota Population Center. South Africa Census 2001 from the Integrated Public Use Microdata Series, International: [Machine-readable database]. Minneapolis: University of Minnesota.	6 - Risk Factors
43158	Census	South Africa Community Survey 2007 - IPUMS	Statistics South Africa, Minnesota Population Center. South Africa Community Survey 2007 from the Integrated Public Use Microdata Series, International: [Machine-readable database]. Minneapolis: University of Minnesota.	6 - Risk Factors
12146	Census	South Africa Population and Housing Census 2011	Statistics South Africa. South Africa Population and Housing Census 2011.	6 - Risk Factors
227194	Census	South Africa Population and Housing Census 2011 - IPUMS	Minnesota Population Center, Statistics South Africa. South Africa Population and Housing Census 2011 from the Integrated Public Use Microdata Series, International: [Machine-readable database]. Minneapolis: University of Minnesota, 2015.	6 - Risk Factors
425016	Environmental monitoring	European Reanalysis-5	European Centre for Medium-Range Weather Forecasts (ECMWF). European Reanalysis-5. United Kingdom: European Centre for Medium-Range Weather Forecasts (ECMWF).	6 - Risk Factors

424709	Environmental monitoring	Particulate Matter 2.5 and 10 Surface Monitor Station Expanded Database 2008-2017	Shaddick G, Thomas ML. Particulate Matter 2.5 and 10 Surface Monitor Station Expanded Database 2008-2017. [Unpublished].	6 - Risk Factors
133004	Environmental monitoring	South Africa Air Quality Information System Database	Department of Environmental Affairs (South Africa), South African Weather Service. South Africa Air Quality Information System Database. Pretoria, South Africa: South African Weather Service.	6 - Risk Factors
144369	Estimate	International Labour Organization Database (ILOSTAT) - Employment by Sex and Economic Activity	International Labour Organization (ILO). International Labour Organization Database (ILOSTAT) - Employment by Sex and Economic Activity. International Labour Organization (ILO).	6 - Risk Factors
144370	Estimate	International Labour Organization Database (ILOSTAT) - Employment by Sex and Occupation	International Labour Organization (ILO). International Labour Organization Database (ILOSTAT) - Employment by Sex and Occupation. International Labour Organization (ILO).	6 - Risk Factors
309568	Estimate	International Labour Organization Database (ILOSTAT) - Employment to Population Ratio by Sex and Age	International Labour Organization (ILO). International Labour Organization Database (ILOSTAT) - Employment to Population Ratio by Sex and Age. International Labour Organization (ILO).	6 - Risk Factors
136038	Estimate	WHO Global Database on Child Growth and Malnutrition	World Health Organization (WHO). WHO Global Database on Child Growth and Malnutrition. Geneva, Switzerland: World Health Organization (WHO).	6 - Risk Factors
136039	Estimate	WHO Global Database on Child Growth and Malnutrition - Historical	World Health Organization (WHO). WHO Global Database on Child Growth and Malnutrition - Historical. Geneva, Switzerland: World Health Organization (WHO).	6 - Risk Factors
420659	Report	WHO Report on the Global Tobacco Epidemic 2019	World Health Organization (WHO). WHO Report on the Global Tobacco Epidemic 2019. Geneva, Switzerland: World Health Organization (WHO), 2019.	6 - Risk Factors
142681	Scientific literature	A cross-sectional study of vascular risk factors in a rural South African population: data from the Southern African Stroke Prevention Initiative (SASPI)	Thorogood M, Connor M, Tollman S, Lewando Hundt G, Fowkes G, Marsh J. A cross-sectional study of vascular risk factors in a rural South African population: data from the Southern African Stroke Prevention Initiative (SASPI). BMC Public Health. 2007; 326.	6 - Risk Factors
278291	Scientific literature	A follow-up cross-sectional study of environmental lead exposure in early childhood in urban South Africa	Naicker N, Mathee A, Barnes B. A follow-up cross-sectional study of environmental lead exposure in early childhood in urban South Africa. S Afr Med J. 2013; 103(12): 935-8.	6 - Risk Factors
303208	Scientific literature	A High Burden of Hypertension in the Urban Black Population of Cape Town: The Cardiovascular Risk in Black South Africans (CRIBSA) Study	Peer N, Steyn K, Lombard C, Gwebushe N, Levitt N. A High Burden of Hypertension in the Urban Black Population of Cape Town: The Cardiovascular Risk in Black South Africans (CRIBSA) Study. PLoS One. 2013; 8(11): e78567.	6 - Risk Factors
146931	Scientific literature	A study of airborne radon levels in Paarl houses (South Africa) and associated source terms, using electret ion chambers and gamma-ray spectrometry	Lindsay R, Newman RT, Speelman WJ. A study of airborne radon levels in Paarl houses (South Africa) and associated source terms, using electret ion chambers and gamma-ray spectrometry. Appl Radiat Isot. 2008; 66(11): 1611-4.	6 - Risk Factors
131758	Scientific literature	A study of pediatric blood lead levels in a lead mining area in South Africa	Von Schirnding Y, Mathee A, Kibel M, Robertson P, Strauss N, Blignaut R. A study of pediatric blood lead levels in a lead mining area in South Africa. Environ Res. 2003; 93(3): 259-63.	6 - Risk Factors
131757	Scientific literature	A survey of blood lead levels among young Johannesburg school children	Mathee A, von Schirnding YER, Levin J, Ismail A, Huntley R, Cantrell A. A survey of blood lead levels among young Johannesburg school children. Environ Res. 2002; 90(3): 181-4.	6 - Risk Factors
255129	Scientific literature	Anaemia among the inhabitants of a rural area in northern Natal	Mayet FGH, Schutte CHJ, Reinach SG. Anaemia among the inhabitants of a rural area in northern Natal. S Afr Med J. 1985; 67(12): 458-62.	6 - Risk Factors
144642	Scientific literature	Anthropometric profile of a black population of the Cape Peninsula in South Africa	Steyn K, Bourne L, Jooste P, Fourie JM, Rossouw K, Lombard C. Anthropometric profile of a black population of the Cape Peninsula in South Africa. East Afr Med J. 1998; 75(1): 35-40.	6 - Risk Factors
335978	Scientific literature	Assessment and 2-year follow-up of some factors associated with severity of respiratory infections in early childhood	Wesley AG, Loening WE. Assessment and 2-year follow-up of some factors associated with severity of respiratory infections in early childhood. S Afr Med J. 1996; 86(4): 365â€“8.	6 - Risk Factors
132104	Scientific literature	Assessment of age-related bone loss in normal South African women by means of the Hologic QDR 1000 system	Kalla AA, Fataar AB, Bewerunge L. Assessment of age-related bone loss in normal South African women by means of the Hologic QDR 1000 system. S Afr Med J. 1994; 84(7): 398-404.	6 - Risk Factors
131753	Scientific literature	Blood lead levels in a remote, unpolluted rural area in South Africa	Grobler SR, Rossouw RJ, Maresky LS. Blood lead levels in a remote, unpolluted rural area in South Africa. S Afr Med J. 1985; 68(5): 323-4.	6 - Risk Factors
131748	Scientific literature	Blood lead levels in preschool children in Cape Town	Deveaux P, Kibel MA, Dempster WS, Pocock F, Formenti K. Blood lead levels in preschool children in Cape Town. S Afr Med J. 1986; 69(7): 421-4.	6 - Risk Factors

334739	Scientific literature	Ceramic water filters impregnated with silver nanoparticles as a point-of-use water-treatment intervention for HIV-positive individuals in Limpopo Province, South Africa: a pilot study of technological performance and human health benefits	Abebe LS, Smith JA, Narkiewicz S, Oyanedel-Craver V, Conaway M, Singo A, Amidou S, Mojapelo P, Brant J, Dillingham R. Ceramic water filters impregnated with silver nanoparticles as a point-of-use water-treatment intervention for HIV-positive individuals in Limpopo Province, South Africa: a pilot study of technological performance and human health benefits. J Water Health. 2014; 12(2): 288â€“300.	6 - Risk Factors
131133	Scientific literature	Changing patterns of under- and over-nutrition in South African children-future risks of non-communicable diseases	Jinabhai CC, Taylor M, Sullivan KR. Changing patterns of under- and over-nutrition in South African children-future risks of non-communicable diseases. Ann Trop Paediatr. 2005; 25(1): 3-15.	6 - Risk Factors
112021	Scientific literature	Coronary risk factors in the coloured population of the Cape Peninsula	Steyn K, Jooste PL, Langenhoven ML, BenadÃ© AJ, Rossouw JE, Steyn M, Jordaan PC, Parry CD. Coronary risk factors in the coloured population of the Cape Peninsula. S Afr Med J. 1985; 67(16): 619-25.	6 - Risk Factors
193943	Scientific literature	Determinants of acute respiratory infections in Soweto--a population-based birth cohort	Kristensen IA, Olsen J. Determinants of acute respiratory infections in Soweto--a population-based birth cohort. S Afr Med J. 2006; 633-40.	6 - Risk Factors
247417	Scientific literature	Determinants of the components of arterial pressure among older adults--the role of anthropometric and clinical factors: a multi-continent study	Tyrovolas S, Koyanagi A, Garin N, Olaya B, Ayuso-Mateos JL, Miret M, Chatterji S, Tobiasz-Adamczyk B, Koskinen S, Leonardi M, Haro JM. Determinants of the components of arterial pressure among older adults--the role of anthropometric and clinical factors: a multi-continent study. Atherosclerosis. 2015; 238(2): 240â€“9.	6 - Risk Factors
112861	Scientific literature	Diabetes and other disorders of glycemia in a rural South African community: prevalence and associated risk factors	Motala AA, Esterhuizen T, Gouws E, Pirie FJ, Omar MA. Diabetes and other disorders of glycemia in a rural South African community: prevalence and associated risk factors. Diabetes Care. 2008; 31(9): 1783-8.	6 - Risk Factors
307272	Scientific literature	Dietary adequacies among South African adults in rural KwaZulu-Natal	Kolahdooz F, Spearing K, Sharma S. Dietary adequacies among South African adults in rural KwaZulu-Natal. PLoS One. 2013; 8(6:e67184): e67184.	6 - Risk Factors
300996	Scientific literature	Dietary intake of primary school children in relation to food production in a rural area in KwaZulu-Natal, South Africa	Faber M, Smuts CM, Benade AJ. Dietary intake of primary school children in relation to food production in a rural area in KwaZulu-Natal, South Africa. Int J Food Sci Nutr. 1999; 50(1): 57-64.	6 - Risk Factors
131755	Scientific literature	Distribution of blood lead levels in schoolchildren in selected Cape Peninsula suburbs subsequent to reductions in petrol lead	Von Schirnding Y, Mathee A, Robertson P, Strauss N, Kibel M. Distribution of blood lead levels in schoolchildren in selected Cape Peninsula suburbs subsequent to reductions in petrol lead. S Afr Med J. 2001; 91(10): 870-2.	6 - Risk Factors
131752	Scientific literature	Effect of the reduction of petrol lead on the blood lead levels of South Africans	Maresky LS, Grobler SR. Effect of the reduction of petrol lead on the blood lead levels of South Africans. Sci Total Environ. 1993; 136(1-2): 43-8.	6 - Risk Factors
131566	Scientific literature	Effects of Reducing Lead in Gasoline:? An Analysis of the International Experience	Thomas VM, Socolow RH, Fanelli JJ, Spiro TG. Effects of Reducing Lead in Gasoline:? An Analysis of the International Experience. Environ Sci Technol. 1999; 33(22): 3942-8.	6 - Risk Factors
305367	Scientific literature	Energy, macro- and micronutrient intake among a true longitudinal group of South African adolescents at two interceptions (2000 and 2003): the Birth-to-Twenty (Bt20) Study	MacKeown JM, Pedro TM, Norris SA. Energy, macro- and micronutrient intake among a true longitudinal group of South African adolescents at two interceptions (2000 and 2003): the Birth-to-Twenty (Bt20) Study. Public Health Nutr. 2007; 10(6): 635â€“43.	6 - Risk Factors
122583	Scientific literature	Environmental lead exposure and socio-behavioural adjustment in the early teens: the birth to twenty cohort	Naicker N, Richter L, Mathee A, Becker P, Norris SA. Environmental lead exposure and socio-behavioural adjustment in the early teens: the birth to twenty cohort. Sci Total Environ. 2012; 120-5.	6 - Risk Factors
131759	Scientific literature	Estimating the burden of disease attributable to lead exposure in South Africa in 2000	Norman R, Mathee A, Barnes B, van der Merwe L, Bradshaw D. Estimating the burden of disease attributable to lead exposure in South Africa in 2000. S Afr Med J. 2007; 97(8 Pt 2): 773-80.	6 - Risk Factors
247355	Scientific literature	Evaluation of waist-to-height ratio to predict 5 year cardiometabolic risk in sub-Saharan African adults	Ware LJ, Rennie KL, Kruger HS, Kruger IM, Greeff M, Fourie CMT, Huisman HW, Scheepers JDW, Uys AS, Kruger R, Van Rooyen JM, Schutte R, Schutte AE. Evaluation of waist-to-height ratio to predict 5 year cardiometabolic risk in sub-Saharan African adults. Nutr Metab Cardiovasc Dis. 2014; 24(8): 900â€“7.	6 - Risk Factors
193734	Scientific literature	Health Impact Of Small-Community Water Supply Reliability	Majuru B, Michael Mokoena M, Jagals P, Hunter PR. Health Impact Of Small-Community Water Supply Reliability. Int J Hyg Environ Health. 2011; 214(2): 162-6.	6 - Risk Factors
357870	Scientific literature	Indoor air pollution from unprocessed solid fuel use and pneumonia risk in children aged under five years: a systematic review and meta-analysis	Dherani M, Pope D, Mascarenhas M, Smith KR, Weber M, Bruce N. Indoor air pollution from unprocessed solid fuel use and pneumonia risk in children aged under five years: a systematic review and meta-analysis. Bull World Health Organ. 2008; 86(5): 390-398C.	6 - Risk Factors

250017	Scientific literature	Intimate partner femicide in South Africa in 1999 and 2009	Abrahams N, Mathews S, Martin LJ, Lombard C, Jewkes R. Intimate partner femicide in South Africa in 1999 and 2009. PLoS Med. 2013; 10(4): e1001412.	6 - Risk Factors
163087	Scientific literature	Intimate partner violence, relationship power inequity, and incidence of HIV infection in young women in South Africa: a cohort study	Jewkes RK, Dunkle K, Nduna M, Shai N. Intimate partner violence, relationship power inequity, and incidence of HIV infection in young women in South Africa: a cohort study. Lancet. 2010; 376(9734): 41â€"8.	6 - Risk Factors
255133	Scientific literature	Iron, folate and vitamin B12 nutrition and anaemia in black preschool children in the northern Transvaal	Van der Westhuyzen J, Van Tonder SV, Gilbertson I, Metz J. Iron, folate and vitamin B12 nutrition and anaemia in black preschool children in the northern Transvaal. S Afr Med J. 1986; 70(3): 143-6.	6 - Risk Factors
131751	Scientific literature	Lead absorption in Cape children: a preliminary report	White NW, Dempster WS, Pocock F, Kibel MA. Lead absorption in Cape children: a preliminary report. S Afr Med J. 1982; 62(22): 799-802.	6 - Risk Factors
278441	Scientific literature	Lead exposure in young school children in South African subsistence fishing communities	Mathee A, Khan T, Naicker N, Kootbodien T, Naidoo S, Becker P. Lead exposure in young school children in South African subsistence fishing communities. Environ Res. 2013; 126: 179â€"83.	6 - Risk Factors
278439	Scientific literature	Lead in Paint: Three Decades Later and Still a Hazard for African Children?	Mathee A, RÃ¶llin H, Levin J, Naik I. Lead in Paint: Three Decades Later and Still a Hazard for African Children?. Environ Health Perspect. 2007; 115(3): 321â€"2.	6 - Risk Factors
131756	Scientific literature	Lead poisoning of children in Africa, II. Kwazulu/Natal, South Africa	Nriagu J, Jinabhai CC, Naidoo R, Coutsoudis A. Lead poisoning of children in Africa, II. Kwazulu/Natal, South Africa. Sci Total Environ. 1997; 197(1-3): 1-11.	6 - Risk Factors
131749	Scientific literature	Lead reduction of petrol and blood lead concentrations of athletes	Grobler SR, Maresky LS, Kotze TJ. Lead reduction of petrol and blood lead concentrations of athletes. Arch Environ Health. 1992; 47(2): 139-42.	6 - Risk Factors
131750	Scientific literature	Maternal and fetal blood lead levels	Karimi PG, Moodley J, Jinabhai CC, Nriagu J. Maternal and fetal blood lead levels. S Afr Med J. 1999; 89(6): 676-9.	6 - Risk Factors
150204	Scientific literature	Mortality of women from intimate partner violence in South Africa: a national epidemiological study	Abrahams N, Jewkes R, Martin LJ, Matthews S, Vetten L, Lombard C. Mortality of women from intimate partner violence in South Africa: a national epidemiological study. Violence Vict. 2009; 24(4): 546-56.	6 - Risk Factors
144174	Scientific literature	Nutritional status and dietary intakes of children aged 2-5 years and their caregivers in a rural South African community	Faber M, Jogessar VB, BenadÃ© AJ. Nutritional status and dietary intakes of children aged 2-5 years and their caregivers in a rural South African community. Int J Food Sci Nutr. 2001; 52(5): 401-11.	6 - Risk Factors
142680	Scientific literature	Obesity and overweight in South African primary school children -- the Health of the Nation Study	Armstrong MEG, Lambert MI, Sharwood KA, Lambert EV. Obesity and overweight in South African primary school children -- the Health of the Nation Study. S Afr Med J. 2006; 96(5): 439-44.	6 - Risk Factors
144701	Scientific literature	Physical activity, change in blood pressure and predictors of mortality in older South Africans--a 2-year follow-up study	Charlton KE, Lambert EV, Kreft J. Physical activity, change in blood pressure and predictors of mortality in older South Africans--a 2-year follow-up study. S Afr Med J. 1997; 87(9): 1124-30.	6 - Risk Factors
436927	Scientific literature	Post-mortem toxicology in violent fatalities in Cape Town, South Africa: A preliminary investigation	Auckloo MBKM, Davies BB. Post-mortem toxicology in violent fatalities in Cape Town, South Africa: A preliminary investigation. J Forensic Leg Med. 2019; 63: 18-25.	6 - Risk Factors
278257	Scientific literature	Prenatal exposure to manganese in South African coastal communities	Rollin HB, Kootbodien T, Theodorou P, Odland JO. Prenatal exposure to manganese in South African coastal communities. Environ Sci Process Impacts. 2014; 16(8): 1903-12.	6 - Risk Factors
124311	Scientific literature	Prevalence of cardiovascular diseases and associated risk factors in a rural black population of South Africa	Alberts M, Urdal P, Steyn K, Stensvold I, Tverdal A, Nel JH, Steyn NP. Prevalence of cardiovascular diseases and associated risk factors in a rural black population of South Africa. Eur J Prev Cardiol. 2005; 12(4): 347-54.	6 - Risk Factors
150611	Scientific literature	Prevalence of emotional, physical and sexual abuse of women in three South African provinces	Jewkes R, Penn-Kekana L, Levin J, Ratsaka M, Schrieber M. Prevalence of emotional, physical and sexual abuse of women in three South African provinces. S Afr Med J. 2001; 91(5): 421-8.	6 - Risk Factors
131754	Scientific literature	Reductions in blood lead levels among school children following the introduction of unleaded petrol in South Africa	Mathee A, RÃ¶llin H, von Schirnding Y, Levin J, Naik I. Reductions in blood lead levels among school children following the introduction of unleaded petrol in South Africa. Environ Res. 2006; 100(3): 319-22.	6 - Risk Factors
333198	Scientific literature	Risk factors for admission and the role of respiratory syncytial virus-specific cytotoxic T-lymphocyte responses in children with acute bronchiolitis	Jeena PM, Ayannusi OE, Annamalai K, Naidoo P, Coovadia HM, Guldner P. Risk factors for admission and the role of respiratory syncytial virus-specific cytotoxic T-lymphocyte responses in children with acute bronchiolitis. S Afr Med J. 2003; 93(4): 291â€"4.	6 - Risk Factors
264304	Scientific literature	Sex differences in obesity incidence: 20-year prospective cohort in South Africa	Lundeen EA, Norris SA, Adair LS, Richter LM, Stein AD. Sex differences in obesity incidence: 20-year prospective cohort in South Africa. Pediatr Obes. 2016; 11(1): 75â€"80.	6 - Risk Factors
294625	Scientific literature	SODIUM INTAKES AROUND THE WORLD [Internet]. Paris: World	Brown I, Elliott P. SODIUM INTAKES AROUND THE WORLD [Internet]. Paris: World Health Organization;. 2006; 85.	6 - Risk Factors

		Health Organization;		
137171	Scientific literature	Substance abuse and behavioral correlates of sexual assault among South African adolescents	King G, Flisher AJ, Noubary F, Reece R, Marais A, Lombard C. Substance abuse and behavioral correlates of sexual assault among South African adolescents. Child Abuse Negl. 2004; 28(6): 683-96.	6 - Risk Factors
336701	Scientific literature	The association between body composition, 25(OH)D, and PTH and bone mineral density in black African and Asian Indian population groups	George JA, Micklesfield LK, Norris SA, Crowther NJ. The association between body composition, 25(OH)D, and PTH and bone mineral density in black African and Asian Indian population groups. J Clin Endocrinol Metab. 2014; 99(6): 2146â€"54.	6 - Risk Factors
142679	Scientific literature	The determinants of overweight and obesity among 10- to 15-year-old schoolchildren in the North West Province, South Africa - the THUSA BANA (Transition and Health during Urbanisation of South Africans; BANA, children) study	Kruger R, Kruger HS, Macintyre UE. The determinants of overweight and obesity among 10- to 15-year-old schoolchildren in the North West Province, South Africa - the THUSA BANA (Transition and Health during Urbanisation of South Africans; BANA, children) study. Public Health Nutr. 2006; 9(3): 351-8.	6 - Risk Factors
387845	Scientific literature	The effect of physical activity on mortality and cardiovascular disease in 130â€ˆ000 people from 17 high-income, middle-income, and low-income countries: the PURE study	Lear SA, Hu W, Rangarajan S, Gasevic D, Leong D, Iqbal R, Casanova A, Swaminathan S, Anjana RM, Kumar R, Rosengren A, Wei L, Yang W, Chuangshi W, Huaxing L, Nair S, Diaz R, Swidon H, Gupta R, Mohammadifard N, Lopez-Jaramillo P, Oguz A, Zatonska K, Seron P, Avezum A, Poirier P, Teo K, Yusuf S. The effect of physical activity on mortality and cardiovascular disease in 130â€ˆ000 people from 17 high-income, middle-income, and low-income countries: the PURE study. Lancet. 2017; 390(10113): 2643-2654.	6 - Risk Factors
144649	Scientific literature	The epidemic of obesity in South Africa: a study in a disadvantaged community	Temple NJ, Steyn K, Hoffman M, Levitt NS, Lombard CJ. The epidemic of obesity in South Africa: a study in a disadvantaged community. Ethn Dis. 2001; 11(3): 431-7.	6 - Risk Factors
144167	Scientific literature	The nutritional status of a rural community in KwaZulu-Natal, South Africa: the Ndunakazi project	Oelofse A, Faber M, BenadÃ© JG, BenadÃ© AJ, Kenoyer DG. The nutritional status of a rural community in KwaZulu-Natal, South Africa: the Ndunakazi project. Cent Afr J Med. 1999; 45(1): 14-9.	6 - Risk Factors
304092	Scientific literature	The nutritional status of asymptomatic HIV-infected Africans: directions for dietary intervention?	Vorster HH, Kruger A, Margetts BM, Venter CS, Kruger HS, Veldman FJ, Macintyre UE. The nutritional status of asymptomatic HIV-infected Africans: directions for dietary intervention?. Public Health Nutr. 2004; 7(8): 1055â€"64.	6 - Risk Factors
112860	Scientific literature	The prevalence and identification of risk factors for NIDDM in urban Africans in Cape Town, South Africa	Levitt NS, Katzenellenbogen JM, Bradshaw D, Hoffman MN, Bonnici F. The prevalence and identification of risk factors for NIDDM in urban Africans in Cape Town, South Africa. Diabetes Care. 1993; 16(4): 601-7.	6 - Risk Factors
264260	Scientific literature	The relationship between BMI and dietary intake of primary school children from a rural area of South Africa: The Ellisras longitudinal study	Van Den Ende C, Twisk JWR, Monyeki KD. The relationship between BMI and dietary intake of primary school children from a rural area of South Africa: The Ellisras longitudinal study. Am J Hum Biol. 2014; 26(5): 701â€"6.	6 - Risk Factors
120028	Scientific literature	Trauma exposure and post-traumatic stress symptoms in urban African schools Survey in CapeTown and Nairobi	Seedat S, Nyamai C, Njenga F, Vythilingum B, Stein DJ. Trauma exposure and post-traumatic stress symptoms in urban African schools Survey in CapeTown and Nairobi. Br J Psychiatry. 2004; 184(2): 169-75.	6 - Risk Factors
193750	Scientific literature	Use of ceramic water filtration in the prevention of diarrheal disease: a randomized controlled trial in rural South Africa and zimbabwe	Du Preez M, Conroy RM, Wright JA, Moyo S, Potgieter N, Gundry SW. Use of ceramic water filtration in the prevention of diarrheal disease: a randomized controlled trial in rural South Africa and zimbabwe. Am J Trop Med Hyg. 2008; 79(5): 696-701.	6 - Risk Factors
377874	Survey	South Africa - Agincourt Health and Aging in Africa Baseline Survey 2014-2015	Berkman, Lisa. Health and Aging in Africa: A Longitudinal Study of an INDEPTH Community in South Africa [HAALSI] Baseline Survey: Agincourt, South Africa, 2015. Ann Arbor, MI: Inter-university Consortium for Political and Social Research [distributor], 2017-10-30. https://doi.org/10.3886/ICPSR36633.v2	6 - Risk Factors
135119	Survey	South Africa - Agincourt INDEPTH Study on Global Ageing and Adult Health 2006-2007	MRC/Wits Rural Public Health and Health Transitions Research Unit (Agincourt), World Health Organization (WHO). South Africa - Agincourt INDEPTH Study on Global Ageing and Adult Health 2006-2007. Geneva, Switzerland: World Health Organization (WHO).	6 - Risk Factors
135825	Survey	South Africa - Agincourt Integrated Family Survey 2002	Case, A. 2003. Agincourt Integrated Family Survey 2002. [dataset] Version 1. Cape Town: DataFirst [distributor].	6 - Risk Factors
135826	Survey	South Africa - Agincourt Integrated Family Survey 2004	Case, A. 2005. Agincourt Integrated Family Survey 2004. [dataset] Version 1. Cape Town: DataFirst [distributor].	6 - Risk Factors
11928	Survey	South Africa - KwaZulu and Natal Income Dynamics Study 1993	International Food Policy Research Institute (IFPRI), University of Natal, University of Wisconsin, Southern Africa Labour Development Research Unit (SALDRU), School of Economics, University of Cape Town. South Africa KwaZulu-Natal Income	6 - Risk Factors

			Dynamics Study 1993. Durban, South Africa: University of Natal.	
261887	Survey	South Africa - Venda Malnutrition and Enteric Disease Study 2009-2014	Fogarty International Center, National Institutes of Health (NIH), Foundation for the National Institutes of Health (FNIH), University of Venda. South Africa - Venda Malnutrition and Enteric Disease Study 2009-2014.	6 - Risk Factors
139810	Survey	South Africa Anthropometric, Vitamin A, Iron and Immunisation Coverage Status in Children Aged 6 to 71 Months 1994	South African Vitamin A Consultative Group (SAVACG). South Africa Anthropometric, Vitamin A, Iron and Immunisation Coverage Status in Children Aged 6 to 71 Months 1994.	6 - Risk Factors
25100	Survey	South Africa Community Survey 2007	Statistics South Africa. South Africa Community Survey 2007. Pretoria, South Africa: Statistics South Africa.	6 - Risk Factors
280803	Survey	South Africa Community Survey 2016	Statistics South Africa. South Africa Community Survey 2016. Pretoria, South Africa: Statistics South Africa, 2016.	6 - Risk Factors
20796	Survey	South Africa Demographic and Health Survey 1998	Department of Health (South Africa), Macro International, Inc, South African Medical Research Council. South Africa Demographic and Health Survey 1998. Fairfax, United States of America: ICF International.	6 - Risk Factors
20798	Survey	South Africa Demographic and Health Survey 2003-2004	Department of Health (South Africa), Macro International, Inc, South African Medical Research Council. South Africa Demographic and Health Survey 2003-2004.	6 - Risk Factors
157064	Survey	South Africa Demographic and Health Survey 2016	Department of Health (South Africa), ICF International, South African Medical Research Council, Statistics South Africa. South Africa Demographic and Health Survey 2016. Fairfax, United States of America: ICF International, 2019.	6 - Risk Factors
115481	Survey	South Africa General Household Survey 2002	Statistics South Africa. South Africa General Household Survey 2002. Pretoria, South Africa: Statistics South Africa.	6 - Risk Factors
11787	Survey	South Africa General Household Survey 2003	Statistics South Africa. South Africa General Household Survey 2003. Pretoria, South Africa: Statistics South Africa.	6 - Risk Factors
11788	Survey	South Africa General Household Survey 2004	Statistics South Africa. South Africa General Household Survey 2004. Pretoria, South Africa: Statistics South Africa.	6 - Risk Factors
11789	Survey	South Africa General Household Survey 2005	Statistics South Africa. South Africa General Household Survey 2005. Pretoria, South Africa: Statistics South Africa.	6 - Risk Factors
115486	Survey	South Africa General Household Survey 2006	Statistics South Africa. South Africa General Household Survey 2006. Pretoria, South Africa: Statistics South Africa.	6 - Risk Factors
11790	Survey	South Africa General Household Survey 2007	Statistics South Africa. South Africa General Household Survey 2007. Cape Town, South Africa: DataFirst.	6 - Risk Factors
115488	Survey	South Africa General Household Survey 2008	Statistics South Africa. South Africa General Household Survey 2008. Pretoria, South Africa: Statistics South Africa.	6 - Risk Factors
115489	Survey	South Africa General Household Survey 2009	Statistics South Africa. South Africa General Household Survey 2009. Pretoria, South Africa: Statistics South Africa.	6 - Risk Factors
115490	Survey	South Africa General Household Survey 2010	Statistics South Africa. South Africa General Household Survey 2010. Pretoria, South Africa: Statistics South Africa.	6 - Risk Factors
115491	Survey	South Africa General Household Survey 2011	Statistics South Africa. South Africa General Household Survey 2011. Cape Town, South Africa: DataFirst.	6 - Risk Factors
135534	Survey	South Africa General Household Survey 2012	Statistics South Africa. South Africa General Household Survey 2012. Pretoria, South Africa: Statistics South Africa, 2013.	6 - Risk Factors
238483	Survey	South Africa General Household Survey 2013	Statistics South Africa. General Household Survey 2013 [dataset]. Version 1. Pretoria. Statistics South Africa [producer], 2014. Cape Town. DataFirst [distributor], 2014.10.25828/a704-wm31	6 - Risk Factors
238485	Survey	South Africa General Household Survey 2014	Statistics South Africa. South Africa General Household Survey 2014. Cape Town, South Africa: DataFirst, 2015.	6 - Risk Factors
265084	Survey	South Africa General Household Survey 2015	Statistics South Africa. South Africa General Household Survey 2015. Cape Town, South Africa: DataFirst, 2015.	6 - Risk Factors
317089	Survey	South Africa General Household Survey 2016	Statistics South Africa. South Africa General Household Survey 2016. Cape Town, South Africa: DataFirst, 2015.	6 - Risk Factors
11795	Survey	South Africa Global Youth Tobacco Survey 1999	Centers for Disease Control and Prevention (CDC) and World Health Organization (WHO). South Africa Global Youth Tobacco Survey 1999. Atlanta, United States: Centers for Disease Control and Prevention (CDC).	6 - Risk Factors
11800	Survey	South Africa Global Youth Tobacco Survey 2002	Centers for Disease Control and Prevention (CDC) and World Health Organization (WHO). South Africa Global Youth Tobacco Survey 2002. Atlanta, United States: Centers for Disease Control and Prevention (CDC).	6 - Risk Factors
110319	Survey	South Africa Global Youth Tobacco Survey 2008	Centers for Disease Control and Prevention (CDC), World Health Organization (WHO). South Africa Global Youth Tobacco Survey 2008. Atlanta, United States: Centers for Disease Control and Prevention (CDC).	6 - Risk Factors

110320	Survey	South Africa Global Youth Tobacco Survey 2011	Centers for Disease Control and Prevention (CDC), World Health Organization (WHO). South Africa Global Youth Tobacco Survey 2011. Atlanta, United States of America: Centers for Disease Control and Prevention (CDC), 2013.	6 - Risk Factors
12102	Survey	South Africa HIV/AIDS Behavioral Risks, Sero-Status, and Mass Media Impact Survey 2002	Center for AIDS Development, Research and Evaluation (CADRE) (South Africa), Department of Social Development (South Africa), Family Health International, Geospace International (South Africa), Human Sciences Research Council, Joint United Nations Program on HIV/AIDS (UNAIDS), Medical University of Southern Africa (MEDUNSA), National Agency for AIDS Research (ANRS) (France), National Health Laboratory Service (NHLS) (South Africa), South African Medical Research Council, University of Natal, Wits Health Consortium. South Africa HIV/AIDS Behavioral Risks, Sero-Status, and Mass Media Impact Survey 2002. Pretoria, South Africa: Human Sciences Research Council, 2011.	6 - Risk Factors
11826	Survey	South Africa Income and Expenditure Survey 2000	Statistics South Africa. South Africa Income and Expenditure Survey 2000. Pretoria, South Africa: Statistics South Africa.	6 - Risk Factors
11848	Survey	South Africa Income and Expenditure Survey 2005-2006	Statistics South Africa. South Africa Income and Expenditure Survey 2005-2006. Pretoria, South Africa: Statistics South Africa.	6 - Risk Factors
106158	Survey	South Africa Integrated Family Survey 1999	Southern Africa Labour Development Research Unit (SALDRU), University of Cape Town. South Africa Integrated Family Survey 1999.	6 - Risk Factors
322406	Survey	South Africa International Social Survey Programme: Citizenship II 2015	ISSP Research Group (2016): International Social Survey Programme: Citizenship II - ISSP 2014. GESIS Data Archive, Cologne. ZA6670 Data file Version 2.0.0, doi:10.4232/1.12590	6 - Risk Factors
313400	Survey	South Africa International Social Survey Programme: Environment III 2010	ISSP Research Group (2012): International Social Survey Programme: Environment III - ISSP 2010. GESIS Data Archive, Cologne. ZA5500 Data file Version 2.0.0, doi:10.4232/1.11418.	6 - Risk Factors
142804	Survey	South Africa International Social Survey Programme: Health and Health Care 2011	ISSP Research Group (2009): International Social Survey Programme: Health and Health Care - ISSP 2011. GESIS Data Archive, Cologne. ZA5800 Data file version 3.0.0, doi:10.4232/1.12252.	6 - Risk Factors
322368	Survey	South Africa International Social Survey Programme: National Identity III 2013-2014	ISSP Research Group (2015): International Social Survey Programme: National Identity III - ISSP 2013. GESIS Data Archive, Cologne. ZA5950 Data file Version 2.0.0, doi:10.4232/1.12312	6 - Risk Factors
322486	Survey	South Africa International Social Survey Programme: Work Orientations IV 2015-2016	The International Social Survey Programme (ISSP) represents a series of cross-national collaborations between organizations conducting similar social science research. ISSP researchers focus on developing survey questions that are relevant and comprehensible in all countries and languages, with survey results that are comparable across nations.Topics covered on this round of the ISSP include: working conditions; labor relations and conflict; and employment and unemployment.	6 - Risk Factors
12013	Survey	South Africa KwaZulu-Natal Income Dynamics Study 1998	International Food Policy Research Institute (IFPRI), University of Natal, University of Wisconsin, Data Research Africa (DRA), Policy and Praxis, Southern Africa Labour Development Research Unit (SALDRU), School of Economics, University of Cape Town. South Africa KwaZulu-Natal Income Dynamics Study 1998. Durban, South Africa: University of Natal.	6 - Risk Factors
31142	Survey	South Africa KwaZulu-Natal Income Dynamics Study 2004	University of Kwazulu-Natal, University of Wisconsin, London School of Hygiene and Tropical Medicine, International Food Policy Research Institute (IFPRI), Department of Social Development (South Africa), Norwegian Institute for Urban and Regional Research (NIBR). South Africa KwaZulu-Natal Income Dynamics Study 2004. Durban, South Africa: University of Kwazulu-Natal.	6 - Risk Factors
280043	Survey	South Africa Labor Market Dynamics 2008	Statistics South Africa. Labour Market Dynamics in South Africa 2008 [dataset]. Version 1.1. Pretoria: Statistics South Africa [producer], 2015. Cape Town: DataFirst [distributor], 2015.	6 - Risk Factors
280050	Survey	South Africa Labor Market Dynamics 2009	Statistics South Africa. Labour Market Dynamics in South Africa 2009 [dataset]. Version 1.1. Pretoria: Statistics South Africa [producer], 2015. Cape Town: DataFirst [distributor], 2015.	6 - Risk Factors
280051	Survey	South Africa Labor Market Dynamics 2010	Statistics South Africa. Labour Market Dynamics in South Africa 2010 [dataset]. Version 1.1. Pretoria: Statistics South Africa [producer], 2015. Cape Town: DataFirst [distributor], 2015.	6 - Risk Factors
280063	Survey	South Africa Labor Market Dynamics 2011	Statistics South Africa. Labour Market Dynamics in South Africa 2011 [dataset]. Version 1.2. Pretoria: Statistics South Africa [producer], 2015. Cape Town: DataFirst [distributor], 2015.	6 - Risk Factors

280069	Survey	South Africa Labor Market Dynamics 2012	Statistics South Africa. Labour Market Dynamics in South Africa 2012 [dataset]. Version 1.2. Pretoria: Statistics South Africa [producer], 2015. Cape Town: DataFirst [distributor], 2015.	6 - Risk Factors
280075	Survey	South Africa Labor Market Dynamics 2013	Statistics South Africa. Labour Market Dynamics in South Africa 2013 [dataset]. Version 1. Pretoria: Statistics South Africa [producer], 2014. Cape Town: DataFirst [distributor], 2015.	6 - Risk Factors
12101	Survey	South Africa Living Standards Measurement Study 1993	Southern Africa Labour Development Research Unit (SALDRU), University of Cape Town, World Bank. South Africa Living Standards Measurement Study 1993. Washington DC, United States of America: World Bank.	6 - Risk Factors
261367	Survey	South Africa National Food Consumption Survey - Fortification Baseline 2005	Department of Health (South Africa), North-West University, South African Medical Research Council, Stellenbosch University, University of Cape Town, University of Kwazulu-Natal, University of Limpopo, University of Pretoria, University of the Free State, University of the Western Cape. South Africa National Food Consumption Survey - Fortification Baseline 2005.	6 - Risk Factors
155628	Survey	South Africa National Health and Nutrition Examination Survey 2012	Human Sciences Research Council, South African Medical Research Council. South Africa National Health and Nutrition Examination Survey 2012.	6 - Risk Factors
313076	Survey	South Africa National HIV Prevalence, Incidence, and Behavior Survey 2011-2012	Centers for Disease Control and Prevention (CDC), Global Clinical and Viral Laboratory (South Africa), Human Sciences Research Council, National Institute for Communicable Diseases (South Africa), South African Medical Research Council, University of Cape Town. South Africa National HIV Prevalence, Incidence, and Behavior Survey 2011-2012. Pretoria, South Africa: Human Sciences Research Council, 2016.	6 - Risk Factors
313074	Survey	South Africa National HIV Prevalence, Incidence, Behavior and Communication Survey 2004-2005	Center for AIDS Development, Research and Evaluation (CADRE) (South Africa), Centers for Disease Control and Prevention (CDC), Global Clinical and Viral Laboratory (South Africa), Human Sciences Research Council, Maphume Research Services, National Institute for Communicable Diseases (South Africa). South Africa National HIV Prevalence, Incidence, Behavior and Communication Survey 2004-2005. Pretoria, South Africa: Human Sciences Research Council, 2011.	6 - Risk Factors
228102	Survey	South Africa National HIV Prevalence, Incidence, Behavior and Communication Survey 2008-2009	Center for AIDS Development, Research and Evaluation (CADRE) (South Africa), Geospace International (South Africa), Global Clinical and Viral Laboratory (South Africa), Human Sciences Research Council, Maphume Research Services, National Institute for Communicable Diseases (South Africa), South African Medical Research Council. South Africa National HIV Prevalence, Incidence, Behavior and Communication Survey 2008-2009. Pretoria, South Africa: Human Sciences Research Council, 2014.	6 - Risk Factors
27885	Survey	South Africa National Income Dynamics Study - Wave 1 2008	University of Cape Town, Southern Africa Labour and Development Research Unit. National Income Dynamics Study (NIDS) Wave 1 [computer files]. Cape Town: Southern Africa Labour and Development Research Unit [producer], 2009. Cape Town: DataFirst [distributor], 2009	6 - Risk Factors
133731	Survey	South Africa National Income Dynamics Study - Wave 2 2010-2011	Southern Africa Labour and Development Research Unit. National Income Dynamics Study 2010-2011, Wave 2. Version 1.0. Cape Town: Southern Africa Labour and Development Research Unit [producer], 2012. Cape Town: DataFirst [distributor], 2013.	6 - Risk Factors
133732	Survey	South Africa National Income Dynamics Study - Wave 3 2012	Southern Africa Labour and Development Research Unit. National Income Dynamics Study 2012, Wave 3 [dataset]. Version 1.2. Cape Town: Southern Africa Labour and Development Research Unit [producer], 2013. Cape Town: DataFirst [distributor], 2013	6 - Risk Factors
265153	Survey	South Africa National Income Dynamics Study - Wave 4 2014-2015	Southern Africa Labour and Development Research Unit. National Income Dynamics Study 2014 - 2015, Wave 4 [dataset]. Version 1.1. Cape Town: Southern Africa Labour and Development Research Unit [producer], 2016. Cape Town: DataFirst [distributor], 2016. Pretoria: Department of Planning Monitoring and Evaluation [commissioner], 2014	6 - Risk Factors
257511	Survey	South Africa National Youth Risk Behaviour Survey 2002	Centers for Disease Control and Prevention (CDC), Department of Health (South Africa), National Department of Education (South Africa), South African Medical Research Council. South Africa National Youth Risk Behaviour Survey 2002.	6 - Risk Factors
257515	Survey	South Africa National Youth Risk Behaviour Survey 2008	Department of Health (South Africa), National Department of Education (South Africa), South African Medical Research Council. South Africa National Youth Risk Behaviour Survey 2008.	6 - Risk Factors

105306	Survey	South Africa October Household Survey 1994	Central Statistical Service (South Africa). South Africa October Household Survey 1994.	6 - Risk Factors
106684	Survey	South Africa October Household Survey 1995	Central Statistical Service (South Africa). South Africa October Household Survey 1995.	6 - Risk Factors
106686	Survey	South Africa October Household Survey 1997	Central Statistical Service (South Africa). South Africa October Household Survey 1997.	6 - Risk Factors
12105	Survey	South Africa October Household Survey 1998	Statistics South Africa. South Africa October Household Survey 1998.	6 - Risk Factors
12106	Survey	South Africa October Household Survey 1999	Statistics South Africa. South Africa October Household Survey 1999. Cape Town, South Africa: DataFirst.	6 - Risk Factors
284975	Survey	South Africa Optimus Study 2014	Centre for Justice and Crime Prevention (South Africa), UBS Optimus Foundation, University of Cape Town. South Africa Optimus Study 2014. 2016.	6 - Risk Factors
286339	Survey	South Africa Programmatic Mapping and Size Estimation of Key Populations 2015	Human Sciences Research Council, Networking HIV, AIDS Community of South Africa (NACOSA). South Africa Programmatic Mapping and Size Estimation of Key Populations 2015.	6 - Risk Factors
280812	Survey	South Africa Quarterly Labor Force Survey 2008, Quarter 1	Statistics South Africa. Quarterly Labour Force Survey 2008: Q1 [dataset]. Version 2.0. Pretoria: Statistics South Africa [producer], 2008. Cape Town: DataFirst [distributor], 2012.	6 - Risk Factors
280819	Survey	South Africa Quarterly Labor Force Survey 2009, Quarter 1	Statistics South Africa. Quarterly Labour Force Survey 2009: Q1 [dataset]. Version 2.0. Pretoria: Statistics South Africa [producer], 2009. Cape Town: DataFirst [distributor], 2012.	6 - Risk Factors
280826	Survey	South Africa Quarterly Labor Force Survey 2010, Quarter 1	Statistics South Africa. Quarterly Labour Force Survey 2010: Q1 [dataset]. Version 3.0. Pretoria: Statistics South Africa [producer], 2010. Cape Town: DataFirst [distributor], 2012.	6 - Risk Factors
280854	Survey	South Africa Quarterly Labor Force Survey 2011, Quarter 1	Statistics South Africa. Quarterly Labour Force Survey 2011: Q1 [dataset]. Version 1.1. Pretoria: Statistics South Africa [producer], 2011. Cape Town: DataFirst [distributor], 2012.	6 - Risk Factors
280861	Survey	South Africa Quarterly Labor Force Survey 2012, Quarter 1	Statistics South Africa. Quarterly Labour Force Survey 2012: Q1 [dataset]. Version 1.1. Pretoria: Statistics South Africa [producer], 2012. Cape Town: DataFirst [distributor], 2012.	6 - Risk Factors
280869	Survey	South Africa Quarterly Labor Force Survey 2013, Quarter 1	Statistics South Africa. Quarterly Labour Force Survey 2013: Q1 [dataset]. Version 1.0. Pretoria: Statistics South Africa [producer], 2013. Cape Town: DataFirst [distributor], 2013.	6 - Risk Factors
280875	Survey	South Africa Quarterly Labor Force Survey 2014, Quarter 1	Statistics South Africa. Quarterly Labour Force Survey 2014: Q1 [dataset]. Version 1.0. Pretoria: Statistics South Africa [producer], 2014. Cape Town: DataFirst [distributor], 2014.	6 - Risk Factors
280881	Survey	South Africa Quarterly Labor Force Survey 2015, Quarter 1	Statistics South Africa. Quarterly Labour Force Survey 2015: Q1 [dataset]. Version 1.0. Pretoria: Statistics South Africa [producer], 2015. Cape Town: DataFirst [distributor], 2015.	6 - Risk Factors
280891	Survey	South Africa Quarterly Labor Force Survey 2016, Quarter 1	Statistics South Africa. Quarterly Labour Force Survey 2016: Q1 [dataset]. Version 1.0. Pretoria: Statistics South Africa [producer], 2016. Cape Town: DataFirst [distributor], 2016.	6 - Risk Factors
111488	Survey	South Africa WHO Study on Global AGEing and Adult Health 2007-2008	Department of Health (South Africa), Human Sciences Research Council, World Health Organization (WHO). South Africa WHO Study on Global AGEing and Adult Health 2007-2008. Geneva, Switzerland: World Health Organization (WHO).	6 - Risk Factors
21864	Survey	South Africa World Health Survey 2002-2003	World Health Organization (WHO). South Africa World Health Survey 2002-2003. Geneva, Switzerland: World Health Organization (WHO), 2005.	6 - Risk Factors
344630	Survey	South Africa World Poll 2005-2006	Gallup. South Africa World Poll 2005-2006.	6 - Risk Factors
344740	Survey	South Africa World Poll 2007	Gallup. South Africa World Poll 2007.	6 - Risk Factors
126610	Survey	United Kingdom - England Adult Psychiatric Morbidity Survey 2006-2007 - UK Data Service	National Centre for Social Research and University of Leicester, Adult Psychiatric Morbidity Survey, 2007 [computer file]. 3rd Edition. Colchester, Essex: UK Data Archive [distributor], January 2011. SN: 6379, http://dx.doi.org/10.5255/UKDA-SN-6379-1	6 - Risk Factors
22341	Survey	United Kingdom Health Survey for England 1997-1998 - UK Data Service	Joint Health Surveys Unit of Social and Community Planning Research and University College London, Health Survey for England, 1997 [computer file]. 2nd ed. Colchester, Essex: UK Data Archive [distributor], 4 December 2000. SN: 3979.	6 - Risk Factors
22352	Survey	United Kingdom Health Survey for England 1998-1999 - UK Data Service	National Centre for Social Research, University College London Department of Epidemiology and Public Health, Health Survey for England, 1998 [computer file]. 4th ed. Colchester, Essex: UK Data Archive [distributor], 30 November 2002. SN: 4150.	6 - Risk Factors
22364	Survey	United Kingdom Health Survey for England 1999-2000 - UK Data	National Centre for Social Research and University College London. Department of Epidemiology and Public Health, Health Survey for	6 - Risk Factors

		Service	England, 1999 [computer file]. 3rd Edition. Colchester, Essex: UK Data Archive [distributor], February 2002. SN: 4365.	
22374	Survey	United Kingdom Health Survey for England 2000-2001 - UK Data Service	National Centre for Social Research, University College London Department of Epidemiology and Public Health, Health Survey for England, 2000 [computer file]. Colchester, Essex: UK Data Archive [distributor], 23 April 2002. SN: 4487.	6 - Risk Factors
22388	Survey	United Kingdom Health Survey for England 2001-2002 - UK Data Service	National Centre for Social Research and University College London. Department of Epidemiology and Public Health, Health Survey for England, 2001 [computer file]. 2nd Edition. Colchester, Essex: UK Data Archive [distributor], June 2004. SN: 4628.	6 - Risk Factors
22433	Survey	United Kingdom Health Survey for England 2003-2004 - UK Data Service	National Centre for Social Research and University College London. Department of Epidemiology and Public Health, Health Survey for England, 2003 [computer file]. Colchester, Essex: UK Data Archive [distributor], March 2005. SN: 5098.	6 - Risk Factors
22449	Survey	United Kingdom Health Survey for England 2004-2005 - UK Data Service	National Centre for Social Research and University College London. Department of Epidemiology and Public Health, Health Survey for England, 2004 [computer file]. Colchester, Essex: UK Data Archive [distributor], July 2006. SN: 5439.	6 - Risk Factors
22463	Survey	United Kingdom Health Survey for England 2005-2006 - UK Data Service	National Centre for Social Research and University College London. Department of Epidemiology and Public Health, Health Survey for England, 2005 [computer file]. Colchester, Essex: UK Data Archive [distributor], July 2007. SN: 5675.	6 - Risk Factors
22476	Survey	United Kingdom Health Survey for England 2006-2007 - UK Data Service	National Centre for Social Research and University College London. Department of Epidemiology and Public Health, Health Survey for England, 2006 [computer file]. 4th Edition. Colchester, Essex: UK Data Archive [distributor], July 2011. SN: 5809, http://dx.doi.org/10.5255/UKDA-SN-5809-1	6 - Risk Factors
95628	Survey	United Kingdom Health Survey for England 2007-2008 - UK Data Service	National Centre for Social Research and University College London. Department of Epidemiology and Public Health, Health Survey for England, 2007 [computer file]. 2nd Edition. Colchester, Essex: UK Data Archive [distributor], April 2010. SN: 6112, http://dx.doi.org/10.5255/UKDA-SN-6112-1	6 - Risk Factors
95629	Survey	United Kingdom Health Survey for England 2008-2009 - UK Data Service	National Centre for Social Research and University College London. Department of Epidemiology and Public Health, Health Survey for England, 2008 [computer file]. 3rd Edition. Colchester, Essex: UK Data Archive [distributor], July 2011. SN: 6397, http://dx.doi.org/10.5255/UKDA-SN-6397-1	6 - Risk Factors
95630	Survey	United Kingdom Health Survey for England 2009-2010 - UK Data Service	National Centre for Social Research and University College London. Department of Epidemiology and Public Health, Health Survey for England, 2009 [computer file]. 2nd Edition. Colchester, Essex: UK Data Archive [distributor], July 2011. SN: 6732, http://dx.doi.org/10.5255/UKDA-SN-6732-1	6 - Risk Factors
130050	Survey	United Kingdom National Survey of Sexual Attitudes and Lifestyles 1999-2001 - UK Data Service	National Centre for Social Research et al. , National Survey of Sexual Attitudes and Lifestyles II, 2000-2001 [computer file]. Colchester, Essex: UK Data Archive [distributor], August 2005. SN: 5223, http://dx.doi.org/10.5255/UKDA-SN-5223-1	6 - Risk Factors
43713	Survey	WHO Global Database on Vitamin A Deficiency	World Health Organization (WHO). WHO Global Database on Vitamin A Deficiency. Geneva, Switzerland: World Health Organization (WHO).	6 - Risk Factors
162652	Survey	Zambia Access, Bottlenecks, Costs, and Equity Project 2011-2012	Central Statistical Office (Zambia), Churches Health Association of Zambia (CHAZ), Clinton Health Access Initiative (CHAI), Institute for Health Metrics and Evaluation (IHME), Ministry of Health (Zambia), University of Zambia. Access, Bottlenecks, Costs, and Equity (ABCE) project in Zambia, 2011-2012. Seattle, United States: Institute for Health Metrics and Evaluation (IHME), 2015.	6 - Risk Factors

Table S3: Mortality per 100,000 for all-cause and HIV/AIDS in South Africa, 1990 and 2019

Mortality per 100,000 for all-cause and HIV/AIDS, 1990 and 2019								
	Males				Females			
	All cause		HIV/AIDS		All cause		HIV/AIDS	
	1990	2019	1990	2019	1990	2019	1990	2019
Under 5	1,854.5 (1,595.8 to 2,151.5)	847.9 (656.9 to 1,101.1)	107.8 (50.3 to 201.1)	90.6 (52.7 to 147.9)	1,417.1 (1,221.5 to 1,627.6)	637.9 (509.8 to 809.2)	84 (40.1 to 149.1)	71.7 (42.7 to 116)
5 to 9	145.1 (136.9 to 153.9)	87.1 (77 to 99.8)	0.6 (0.2 to 1.1)	21.2 (14.9 to 28.8)	111.5 (106.1 to 116.9)	68.1 (61.4 to 76.2)	0.5 (0.2 to 1)	18.2 (13.6 to 23.8)
10 to 14	73 (69.4 to 76.7)	73.1 (67.6 to 79.4)	0 (0 to 0)	59 (51.2 to 66.5)	54.5 (52.3 to 57)	57.3 (53.9 to 61.1)	0.1 (0 to 0.1)	47.9 (42.2 to 53.1)
15 to 19	144.2 (136.7 to 152.1)	161.7 (145.4 to 179)	0.5 (0.2 to 1.1)	75.3 (56.9 to 96.1)	114.7 (109.8 to 119.9)	134.8 (119 to 164.3)	5.4 (3.4 to 9.3)	92 (64.7 to 137.6)
20 to 24	324.8 (307.6 to 342.1)	318.6 (299.7 to 341.9)	2 (1.2 to 3.6)	53.8 (37.1 to 79.6)	241.4 (225.7 to 257.6)	262.4 (228.6 to 323.7)	10.7 (6.7 to 18.4)	179.8 (116.8 to 273.4)
25 to 29	624.1 (597.6 to 650.8)	539.6 (505 to 600.4)	7.4 (3.1 to 14.6)	125.6 (56.1 to 235.3)	418.8 (388.5 to 450.7)	445.3 (382.1 to 554.4)	15.4 (6.8 to 28.3)	296.8 (154.6 to 483)
30 to 34	902.2 (863 to 942.6)	821 (751.7 to 953)	13.4 (6 to 24.9)	299.4 (155.8 to 511)	528 (492.7 to 564.5)	627 (541.4 to 761.1)	13.8 (6.6 to 24.1)	440.1 (278.6 to 635.8)
35 to 39	1,005.8 (951.6 to 1,059.5)	1,036.2 (922.3 to 1,233.4)	16.9 (7.4 to 33)	506.4 (291.8 to 821)	533.6 (499.7 to 570.2)	718.8 (621.6 to 866.6)	12.4 (5.5 to 24.3)	531 (370.7 to 734.7)
40 to 44	1,060.1 (991.9 to 1,127.7)	1,215.2 (1,068.5 to 1,434.6)	13.9 (7 to 25)	655.5 (457 to 891.3)	541.1 (507.6 to 575.7)	785.9 (686.7 to 922.7)	11.8 (5.5 to 21.5)	566.8 (449.1 to 706.2)
45 to 49	1,148.8 (1,064.4 to 1,232.3)	1,355.6 (1,191.8 to 1,574.8)	10.7 (5.6 to 18.6)	711.8 (527.3 to 969.8)	576.2 (540.7 to 613.3)	821.7 (731.1 to 935.4)	11.9 (5.9 to 21.7)	532.3 (424.7 to 658.2)
50 to 54	1,390.3 (1,287.5 to 1,493.7)	1,660.7 (1,495.1 to 1,858)	9.2 (5.1 to 15.5)	659 (505.7 to 869.8)	694.7 (650.2 to 738.9)	962.7 (877.3 to 1,060.4)	10.9 (5.8 to 19)	495.9 (402.3 to 601.5)
55 to 59	1,714.9 (1,589.9 to 1,843.8)	2,001.6 (1,839.4 to 2,178.3)	6.8 (3.9 to 11.4)	485.3 (376.7 to 646)	877.7 (822.4 to 933.4)	1,142.9 (1,065.2 to 1,232.3)	7.9 (4.2 to 13.5)	395.1 (324.1 to 474.8)
60 to 64	2,244.9 (2,095.3 to 2,400)	2,636.5 (2,463.7 to 2,818.3)	6.3 (3.6 to 10.4)	340.3 (261.3 to 456.5)	1,233.7 (1,163.2 to 1,303.4)	1,519.6 (1,437.6 to 1,611.2)	5.6 (3.1 to 9.7)	308.5 (250 to 374.3)
65 to 69	2,996.4 (2,809.4 to 3,188.1)	3,540.7 (3,340.9 to 3,741.8)	5.8 (3.3 to 9.8)	258.8 (196.2 to 344.4)	1,819.7 (1,726.5 to 1,911.4)	2,171.9 (2,073.6 to 2,278.9)	4.8 (2.7 to 8)	235.5 (189 to 289.8)
70 to 74	4,080.4 (3,838.8 to 4,323.1)	4,510.3 (4,274.3 to 4,758.9)	6.7 (3.9 to 10.7)	222.4 (166.3 to 299.1)	2,615.8 (2,486.9 to 2,739.8)	3,034.1 (2,903.5 to 3,171.4)	5.8 (3.4 to 9.6)	187.8 (150.7 to 232.6)

Achoki T, *et al. J Epidemiol Community Health* 2022; 76:471–481. doi: 10.1136/jech-2021-217480

75 to 79	5,684.8 (5,371.9 to 5,990.6)	6,352.5 (6,053.4 to 6,670.7)	5.6 (3.4 to 8.6)	196.5 (147.9 to 265.7)	3,783.3 (3,604 to 3,954.2)	4,429.8 (4,248.8 to 4,616.7)	3.5 (2.3 to 5.3)	147.8 (119.2 to 181.3)
80 to 84	9,822.1 (9,388.1 to 10,242.9)	10,701.9 (10,296.7 to 11,122.3)	2.9 (1.5 to 4.7)	81.7 (58.7 to 116.9)	7,819.8 (7,510.6 to 8,104)	8,625.1 (8,356.8 to 8,901.2)	2 (1.3 to 3.3)	49.5 (38.6 to 62.3)
85 to 89	15,979.9 (15,470 to 16,518.7)	17,191.7 (16,761.9 to 17,664.8)	6.1 (3 to 9.8)	74.7 (52.5 to 108.7)	12,814.9 (12,345.5 to 13,275.7)	14,311.3 (13,898.1 to 14,721.9)	3.6 (2.4 to 6.1)	44.5 (34.5 to 55.6)
90 to 94	24,023.5 (23,355.7 to 24,741.1)	25,813.5 (25,316.9 to 26,339.3)	4.6 (2.4 to 7.7)	74.6 (52.3 to 107.6)	20,324.1 (19,758.8 to 20,883.4)	22,718.5 (22,245.3 to 23,196.2)	5.6 (3.7 to 9.2)	44.2 (34.6 to 55.1)
95 plus	36,397.2 (35,483.2 to 37,349)	38,450.8 (37,937.5 to 38,964.4)	15.3 (7.1 to 27)	69.7 (49.3 to 99.1)	32,595.4 (31,957 to 33,207.4)	36,463.8 (36,022.7 to 36,879.3)	5.6 (3.5 to 9.6)	36.6 (28.4 to 45.7)

Achoki T, *et al. J Epidemiol Community Health* 2022; 76:471–481. doi: 10.1136/jech-2021-217480

Table S4: All-cause age-standardised DALY rates, 1990—2019 for South Africa and provinces

All-cause age-standardised DALY dates, 1990-2019, South Africa and provinces															
**Year**	**South Africa**			**Eastern Cape**			**Free State**			**Gauteng**			**KwaZulu-Natal**		
	Value	*Lower*	*Upper*	*Value*	*Lower*	*Upper*	*Value*	*Lower*	*Upper*	*Value*	*Lower*	*Upper*	*Value*	*Lower*	*Upper*
1990	53698.07	49887.61	57809.83	56852.11	52162.4	62075.22	61429.87	56248.65	67230.96	54072.77	49505.92	59167.49	58855.63	54122.83	63909.38
1991	53230.09	49543.03	57469.9	57411.83	52315.41	62728.44	60084	54853.14	65442.45	52929.85	48353.29	57677.71	57832.49	53037.14	63243.78
1992	54544.2	50879.37	58576.12	59411.94	54779.59	64561.8	61667.41	56899.14	67267.6	55146.98	50806.79	59920.34	58207.55	53678.38	63217.13
1993	52947.56	48947.33	56939.45	59065.46	54148.65	64190.85	60146.5	55273.94	64985.24	51773.74	47364.31	56170.81	58460.54	53625.51	63894.94
1994	54591.37	50545.6	58714.7	60879.16	55906.34	65786.62	59679.54	54505.46	64931.13	53097.52	48815.08	57888.3	60818.36	55855.69	66334.84
1995	55667.82	51531.74	60274.73	62091.04	57081.64	67378.72	59996.02	54897.35	65588.53	55497.62	50739.72	60703.1	63572.28	58071.36	69864.46
1996	58201.69	53873.39	63205.21	66854.03	61347.18	72531.68	63071.16	57651.1	69241.55	57935.11	53139.46	63219.89	65681.1	59863.38	73301.65
1997	64023.2	59176.92	69942.05	69815	64253.68	76193.87	69551.19	63516.44	76459.42	65634.42	59954.92	72540.21	74263.1	67758.05	83469.44
1998	68245.4	62875.9	75081.74	77603.22	71582.92	84894.96	75523.64	69142.95	83926.89	66154.03	60078.65	73595.14	81913.95	74010.52	92355.72
1999	70974.39	65022.74	78816.85	80243.33	73422.64	88013.71	80761.38	73489.1	90333.97	68193.07	61582.16	76583.29	84832.64	75718.41	96268.65
2000	75846.52	69039.11	84693.22	87719.21	80547.75	96880.13	88223.51	79957.44	98881.83	70615.47	63341.21	80132.27	90513.19	80566.39	103611.6
2001	78606.11	71153.67	87872.08	90829.19	82548.91	101180.4	93322.49	83923.16	104699.4	72649	64413.12	82202.3	95553.61	84688.74	108832.4
2002	82184.82	73730.49	91978.85	95265.59	86024.77	105928.8	100078.7	90224.93	112233.7	74682.76	65982.36	85058.56	101158.7	89426.56	115134.1
2003	85486.87	76621.15	95692.56	98715.23	88747.36	110090	109600.6	99417.1	121691.4	76198.62	67152.09	86785.84	106187.3	94008.07	120280.2
2004	87229.12	78424.25	97388.41	100825.6	90350.76	112131.1	116335.4	105902.6	128719.5	76879.84	68442.66	87109.37	109001.1	96802.18	123536
2005	87672.04	79167.04	97243.36	101243.2	90988.15	112558.9	119145.1	108698.2	131660.5	76581.47	67377.89	86833.9	109761.5	98254.64	123649.5
2006	87056.96	79022.18	96108.74	100805.3	90969.68	111828.5	118499	108904.6	130040.8	75052.53	66922.78	84343.01	108823.5	98189.4	121361
2007	83870.19	77044.69	92190.74	94868.88	85985.6	104734.3	115407.8	106502.2	126092.6	72275.12	65041.34	80500.75	103691.4	94537.2	115169.9
2008	80246.97	74124.41	87573.28	89838.35	82165.11	98214.26	111167.6	103129.5	120934.8	68812.07	62554.09	76517.01	98391.24	90829.81	108615.2
2009	75602.56	70220.6	82413.89	85150.36	78371.35	92777.7	105273.2	98119.19	114070.8	64592.07	58561	71426.44	91663.14	84891.77	101546.6
2010	71162.99	66110.64	77619.16	82287.28	76115.41	89546.5	96154.79	89557.02	104565.1	61165.91	55576.56	67578.84	85048.76	78559.06	94271.97
2011	66425.9	61795.57	72369.99	75195.6	69267.45	81809.08	86334.62	80083.49	94037.49	57778.41	52708.41	63851.89	79430.75	73277.43	87923.57
2012	62330.51	57948.43	67924.76	69342.39	63589.66	75773.56	76531.17	70381.31	83597.57	54763.93	49861	59961.01	73675.89	68161.48	81482.81
2013	59189.74	54748.36	64672.69	66667.03	61209.69	72809.01	71449.6	65627.13	78623.22	52262.3	47255.71	57520.25	68483.46	62993.25	75961.64
2014	57746.71	53333.09	63214.1	66888.39	61392.52	73529.52	68912.82	63159.85	75759.61	50813.49	45779.8	56383.29	65361.96	59812.74	72829.83
2015	56542.1	51950.48	62149.98	65255.58	59744.24	71894.47	67768.99	61815.16	75111.86	50255.5	45041.52	55868.87	63291.56	57757.94	71140.72
2016	55397.54	50682.6	61319.43	62275.88	56734.55	69368.1	67974.94	61752.01	75567.87	50186.97	44977.4	55971.9	61864.27	56482.55	69670.08
2017	54187.25	49673.57	59462.53	60364.89	54622.21	67660.7	66954.78	60031	74273.52	49300.29	44209.47	54313.78	60302	54748.81	67370.21
2018	51493.9	47103.55	56233.56	57251.82	51867.29	63270.53	63075.29	57161.48	70121.75	46591.17	41645.17	51565.09	57164.43	51805.69	63174.84
2019	49954.28	45909.49	54308.83	55592.99	50336.82	61730.79	61124.11	54800.99	67672.1	44946.18	40337.58	49868.19	55517.46	50048.55	61392.97

All-cause age-standardised DALY dates, 1990-2019, South Africa and provinces															
**Year**	**Limpopo**			**Mpumalanga**			**Northern Cape**			**North-West**			**Western Cape**		
	Value	*Lower*	*Upper*	*Value*	*Lower*	*Upper*	*Value*	*Lower*	*Upper*	*Value*	*Lower*	*Upper*	*Value*	*Lower*	*Upper*
1990	41411.58	37132.47	46192.74	48154.27	43719.91	52909.01	53097.94	48655.66	58050.06	48189.13	43588.67	53329.14	50566.62	46237.38	55115.47
1991	40641.62	36284.73	45408.75	48334.6	43896.41	53050.22	52903.61	48138.52	58029.74	48940.13	44299.41	54140.4	50885.71	46572.1	55526.92
1992	40387	36204.5	44652.49	50664.88	46094.01	55456.37	57566.86	52829.96	62680.79	51841.44	47250.6	56939.5	50780.43	46704.78	54945.16
1993	39662.45	35501.36	44217.84	46604.66	42283.31	51301.36	54261.59	49615.97	58706.14	51107.79	46283.73	56324.36	48081.46	43836.43	52193.71
1994	39987.47	35684.88	44211.05	49416.44	44801.13	54400.52	58736.91	54443.79	63063.04	54960.26	49783.58	60446.81	49136.59	44970.4	53192.19
1995	41880.26	37789.28	46431.21	48065.9	43483.01	53622.78	57298.96	53332.73	61999.54	53900.98	48554.98	59825.67	48311.87	44375.89	52377.57
1996	43012.39	38792.94	47784.34	52405.24	47360.99	58956.96	59899.5	55944.97	64405.89	58961.76	53224.09	65967.11	47417.44	43719.98	51135.8
1997	45787.09	41288.4	50672.64	57579.49	52075.37	64897.7	68753.67	64681.93	73735.59	63846.58	57332.07	71990.98	52389.23	48930.64	56129.73
1998	48981.68	43895.32	54658.84	63444.55	56649.95	72898.26	73829.92	69395.08	79343.11	69820.98	62642.38	79132.5	50507.44	47026.72	54344.06
1999	51916.78	46891.74	58541.83	69072.73	61717.71	79366.62	77227.51	72343.52	83184.7	74181.59	66288.95	84492.43	50393.47	46659.44	54109.94
2000	56311.96	50302.76	63175.96	76576.34	68090.92	88056.84	81179.34	75960.73	87642.09	82239.14	73017.19	93826.72	51534.2	47705.57	55456.13
2001	58621.66	52011.56	66175.18	79000.66	69439.91	91815.67	84348.94	78941.88	91139.15	84347.14	74744.85	96598.39	52059.62	48089.45	56226.5
2002	61948	54868.98	70007.52	83903.53	73843.66	97874.96	87278.98	81261.94	94324.09	87752.72	76902.44	100036.6	53255.26	49001.19	57706.78
2003	65420.78	58179.27	73527.69	90250.81	79623.41	104144.1	89976.01	83794.01	97321.06	90764.31	80029.85	104330.7	53464.93	49146.7	58273.81
2004	68283.32	60387.39	76661.34	93129.09	82125.8	106693.2	92091	85567.01	99570.41	92393.37	81446.57	105649.6	52278.56	47793.98	57212.24
2005	70521.56	62950.89	78710.25	96069.63	85225.38	109226.1	92032.59	85533.39	99100.61	92742.76	81580.52	105793.3	51104.84	46667.53	56146.18
2006	72924.18	65324.54	80829.44	98614.84	88375.42	111468.1	92142.66	85710.12	99224.24	91399.81	81134.07	103600.5	49941.48	45525.33	54926.63
2007	72545.74	65592.93	79708.38	95540.48	86336.05	107731.5	89806.96	83827.65	96648.05	90669.29	81041.63	101717.3	48759.97	44511.95	53532.32
2008	70981.48	64644.94	77789.92	91415.68	83067.68	102265.2	87199.82	81697.15	93602.74	88151.82	79079.05	99088.61	47775.48	43677.83	52526.85
2009	68491.45	62672.5	75231.29	85131.59	77667.79	95073.79	83377.48	78150.04	89078.58	83160.6	75219.63	92765.64	46046.72	42075.63	50577.43
2010	64540.97	59069.27	71215.92	77742.54	70699.9	87347.99	79289.69	74237.1	85191	78017.35	70370.46	87138.21	45383.81	41570.84	49752.51
2011	61019.04	55875.12	67243.97	70606.73	64229.57	79620.29	73908.94	68814.33	79654.94	72599.89	65278.63	81557.68	45052.61	41279.28	49553.76
2012	59639.85	54689.42	65387.52	65448.72	59377.13	73455.68	69066.2	64025.31	74736.84	69180.18	62386.48	76888.18	44096.44	40262.01	48580.24
2013	57688.44	52930.55	63448.86	61363.04	55458.14	69122.24	64845.61	59894.79	70136.45	66168.53	59963.25	73940.97	43235.55	39370.55	47880.39
2014	57253.5	52667.48	62821.12	59218.92	53263.09	67049.78	62753.68	58021.13	68253.18	64797.02	57668.44	72280.53	43060.78	39092.31	47834.4
2015	54216.23	49786.17	59480.21	58425.17	52066.57	66647.93	61062.57	55999.52	66683.44	64378.99	58105.15	71754.11	43431.1	39262.05	48633.6
2016	51726.05	47328.86	57206.87	58273.08	51881.42	67240.49	59435.79	54076.39	65312.67	64063.01	57422.77	72010.27	42216.69	37866.78	47770.11
2017	49935.18	45339.05	55174.62	57380.59	50827.89	65804.54	58446.73	52919.56	64654.24	62464.76	55929.41	69691.38	42129.74	37702.7	47631.48
2018	47695.92	43126.3	52628.81	54272.08	48256.25	62068.78	56143.43	50617	61960.19	59207.74	52586.4	66527.3	41167.26	36780.18	46767.34
2019	46544.79	41889.02	51613.66	52372.65	46176.88	59402.53	54509	49030.01	60399.82	57404.13	50320.4	64112.98	40386.96	36054.21	46142.5

Achoki T, *et al. J Epidemiol Community Health* 2022; 76:471–481. doi: 10.1136/jech-2021-217480

Table S5: Comparison of burden of disease estimates for South Africa

Indicator	SAMRC estimate for year 2017[58]	GBD 2019 estimate for the year 2017
Life expectancy at birth	64.2	63.2 (61.6-64.3)
Life expectancy at birth (Male)	61.2	60.0 (58.1-61.4)
Life expectancy at birth (Female)	67.4	66.3 (64.2-67.7)
Adult mortality (45q15) probability	32%	34.3% (32.0-38.2)
Adult mortality (45q15) probability (Male)	38%	40.0% (36.7-44.7)
Adult mortality (45q15) probability (Female)	26%	28.8% (25.7-33.3)
Average life expectancy at the age of 60 (Male)	15 years	16.2 years (15.9-16.5)
Average life expectancy at the age of 60 (Female)	19 years	19.6 years (19.3-19.8)
Under-5 mortality rate per 1000 livebirths	32	38.9 (33.5-45.3)
Infant mortality rate per 1000 livebirths	23	33.3 (28.8-38.8)
Neonatal mortality rate per 1000 livebirths	12	21.6 (18.6-25.3)
Maternal mortality ratio (MMR) per 100 000 live births *	134	91.1 (56.2-141.2)
* They provide estimate for 2016 not for 2017		

Achoki T, *et al. J Epidemiol Community Health* 2022; 76:471–481. doi: 10.1136/jech-2021-217480

Table S6: List of International Classification of Diseases (ICD) codes mapped to the Global Burden of Diseasecause list for causes of death

Appendix Table 6: List of International Classification of Diseases (ICD) codes mapped to the Global Burden of Disease cause list for causes of death

Cause	ICD10	ICD9
Communicable, maternal, neonatal, and nutritional diseases	A00-A00.9, A01.0-A14, A15-A28.9, A32-A39.9, A48.1-A48.2, A48.4-A48.5, A50- A58, A60-A60.9, A63-A63.8, A65-A65.0, A68-A70, A74, A74.8-A75.9, A77-A96.9,A98-A98.8, B00-B06.9, B10-B10.8, B15-B16.2, B17.0, B17.2, B19.1, B20-B27.9, B29.4, B33-B33.1, B33.3-B33.8, B47-B48.8, B50-B54.0, B55.0, B56-B57.5, B60- B60.8, B63, B65-B67.9, B69-B72.0, B74.3-B75, B77-B77.9, B83-B83.8, B90-B91,B94.1, B95-B95.5, B97.4-B97.6, C58-C58.0, D50.1-D50.8, D51-D52.0, D52.8-D53.9, D70.3, D89.3, E00-E02, E40-E46.9, E51-E61.9, E63-E64.0, E64.2-E64.9, F02.1, F02.4, F07.1, G00.0-G00.8, G03-G03.8, G04-G05.8, G14-G14.6, G21.3, H70-H70.9, I00, I02, I02.9, I98.0-I98.1, J00-J02.8, J03-J03.8, J04-J04.2, J05-J05.1, J06.0-J06.8, J09-J15.8, J16-J16.9, J20-J21.9, J36-J36.0, J91.0, K52.1, K67.0-K67.8, K75.3, K76.3, K77.0, K93.0-K93.1, M03.1, M12.1, M49.0-M49.1, M73.0-M73.1, M89.6, N74.1, N96, N98-N98.9, O00-O07.9, O09-O16.9, O20-O26.9, O28-O36.9, O40- O48.1, O60-O77.9, O80-O92.7, O96-O98.6, O98.8-P04.2, P04.5-P05.9, P07-P15.9, P19-P22.9, P23.0-P23.4, P24-P29.9, P35-P37.2, P37.5-P39.9, P50-P61.9, P70-P70.1, P70.3-P72.9, P74-P78.9, P80-P81.9, P83-P84, P90-P94.9, P96, P96.3-P96.4, P96.8,R19.7, U04-U04.9, U06-U06.9, U82-U89, Z16-Z16.3	001-001.9, 002.0-029, 032-034.9, 036-036.3, 036.5-037.9, 040, 040.1-041.0, 042-066.9, 070.0-070.2, 071-075.9, 078.3-078.7, 079-079.7, 080-083.9, 084.0-084.5, 084.7-084.9, 085.0, 086-088, 088.8-088.9, 090-101.6, 104-104.9, 120-124.9, 125.4-125.9, 127-127.1, 128-129.0, 136-136.2, 137-139.0, 181-181.9, 244.2, 260-263.9, 265 269.9, 281.0-281.9, 320.0-320.8, 321-323.9, 381-383.9, 390-390.9, 392, 392.9, 425.6, 460-464.4, 464.8-464.9, 465.0-465.8, 466-469, 470.0, 475-475.9, 476.9, 480-482.8, 483.0-483.9, 484.0-484.7, 487-489, 630-636.9, 638-638.9, 640-679.1, 716.0, 730.4-730.6, 760-760.6, 760.8-768, 768.2-770, 770.1-775.0, 775.4-779.3, 779.6-779.8, V09-V09.9
HIV/AIDS and sexually transmitted infections	A50-A58, A60-A60.9, A63-A63.8, B20-B24.9, B63, F02.4, I98.0, K67.0-K67.2,M03.1, M73.0-M73.1	042-044.9, 054.1, 090-099.9
HIV/AIDS	B20-B24.9, F02.4	042-044.9
HIV/AIDS - Drug-susceptible Tuberculosis	B20.0	
HIV/AIDS - Multidrug-resistant Tuberculosis without extensive drug resistance		
HIV/AIDS - Extensively drug-resistant Tuberculosis		
HIV/AIDS resulting in other diseases	B20, B20.1-B24.9, F02.4	042-044.9
Sexually transmitted infections excluding HIV	A50-A58, A60-A60.9, A63-A63.8, B63, I98.0, K67.0-K67.2, M03.1, M73.0-M73.1	054.1, 090-099.9
Syphilis	A50-A53.9, I98.0, K67.2, M03.1, M73.1	090-097.9
Chlamydial infection	A55-A56.8, K67.0	
Gonococcal infection	A54-A54.9, K67.1, M73.0	098-098.9
Other sexually transmitted infections	A57-A58, A63-A63.8, B63	099-099.9
Respiratory infections and tuberculosis	A10-A14, A15-A19.9, A48.1, A70, B90-B90.9, B97.4-B97.6, H70-H70.9, J00-J02.8,J03-J03.8, J04-J04.2, J05-J05.1, J06.0-J06.8, J09-J15.8, J16-J16.9, J20-J21.9, J36-J36.0, J91.0, K67.3, K93.0, M49.0, N74.1, P23.0-P23.4, P37.0, U04-U04.9, U84.3	010-019.9, 034.0, 079.6, 137-137.9, 138.0-138.9, 381-383.9, 460-464.4, 464.8-464.9, 465.0-465.8, 466-469, 470.0, 475-475.9, 476.9, 480-482.8, 483.0-483.9, 484.1-484.2, 484.6-484.7, 487-489, 730.4-730.6
Tuberculosis	A10-A14, A15-A19.9, B90-B90.9, K67.3, K93.0, M49.0, N74.1, P37.0, U84.3	010-019.9, 137-137.9, 138.0-138.9, 730.4-730.6
Drug-susceptible tuberculosis	A10-A14, A15-A19.9, B90-B90.9, K67.3, K93.0, M49.0, N74.1, P37.0	010-019.9, 137-137.9, 138.0-138.9, 730.4-730.6

Multidrug-resistant tuberculosis without extensive drug resistance	U84.3	
Extensively drug-resistant tuberculosis		
Lower respiratory infections	A48.1, A70, B97.4-B97.6, J09-J15.8, J16-J16.9, J20-J21.9, J91.0, P23.0-P23.4, U04-U04.9	079.6, 466-469, 470.0, 480-482.8, 483.0-483.9, 484.1-484.2, 484.6-484.7, 487-489
Upper respiratory infections	J00-J02.8, J03-J03.8, J04-J04.2, J05-J05.1, J06.0-J06.8, J36-J36.0	034.0, 460-464.4, 464.8-464.9, 465.0-465.8, 475-475.9, 476.9
Otitis media	H70-H70.9	381-383.9
Enteric infections	A00-A00.9, A01.0-A09.9, A80-A80.9, K52.1, R19.7	001-001.9, 002.0-009.9, 045-045.9, 138
Diarrheal diseases	A00-A00.9, A02-A02.0, A02.8-A07, A07.2-A07.4, A08-A09.9, K52.1, R19.7	001-001.9, 003.8-006.9, 007.4-007.8, 008.2-009.9
Typhoid and paratyphoid	A01.0-A01.4	002.0-002.9
Typhoid fever	A01.0	002.0
Paratyphoid fever	A01.1-A01.4	002.1-002.9
Invasive Non-typhoidal Salmonella (iNTS)	A02.1-A02.2	003-003.7
Other intestinal infectious diseases	A07.0-A07.1, A07.8-A07.9, A80-A80.9	007-007.3, 007.9-008.1, 045-045.9, 138
Neglected tropical diseases and malaria	A68-A68.9, A69.2-A69.9, A75-A75.9, A77-A79.9, A82-A82.9, A90-A96.9, A98- A98.8, B33.0-B33.1, B50-B54.0, B55.0, B56-B57.5, B60-B60.8, B65-B67.9, B69-B72.0, B74.3-B75, B77-B77.9, B83-B83.8, K93.1, P37.1, U06-U06.9	060-061.8, 065-066.9, 071-071.9, 080-083.9, 084.0-084.5, 084.7-084.9, 085.0, 086- 088, 088.8-088.9, 120-124.9, 125.4-125.9, 127-127.1, 128-129.0, 425.6
Malaria	B50-B54.0	084.0-084.5, 084.7-084.9
Leprosy	A30-A30.9	030-030.9
Chagas disease	B57-B57.5, K93.1	086-086.2, 086.9, 425.6
Leishmaniasis	B55.0	085.0
Visceral leishmaniasis	B55.0	085.0
African trypanosomiasis	B56-B56.9	086.3-086.5
Schistosomiasis	B65-B65.9	120-120.9
Cysticercosis	B69-B69.9	123.1
Cystic echinococcosis	B67-B67.4, B67.8-B67.9	122-122.4, 122.8-122.9
Dengue	A90-A91.9	061-061.8
Yellow fever	A95-A95.9	060-060.9
Rabies	A82-A82.9	071-071.9
Intestinal nematode infections	B77-B77.9	127.0
Ascariasis	B77-B77.9	127.0
Ebola	A98.4	
Zika virus	U06-U06.9	
Other neglected tropical diseases	A68-A68.9, A69.2-A69.9, A75-A75.9, A77-A79.9, A92-A94.0, A96-A96.9, A98-A98.3, A98.5-A98.8, B33.0-B33.1, B60-B60.8, B67.5-B67.7, B70-B71.9, B74.3-B75,B83-B83.8, P37.1	065-066.9, 080-083.9, 087-088, 088.8-088.9, 122.5-122.7, 123-123.0, 123.2-124.9, 125.4-125.6, 125.9, 127, 127.1, 128-129.0

Achoki T, *et al. J Epidemiol Community Health* 2022; 76:471–481. doi: 10.1136/jech-2021-217480

Appendix Table 6: List of International Classification of Diseases (ICD) codes mapped to the Global Burden of Disease cause list for causes of death

Cause	ICD10	ICD9
Other infectious diseases	A20-A28.9, A32-A39.9, A48.2, A48.4-A48.5, A65-A65.0, A69-A69.1, A74, A74.8-A74.9, A81-A81.9, A83-A89.9, B00-B06.9, B10-B10.8, B15-B16.2, B17.0, B17.2, B19.1, B25-B27.9, B29.4, B33, B33.3-B33.8, B47-B48.8, B91, B94.1, B95-B95.5, D70.3, D89.3, F02.1, F07.1, G00.0-G00.8, G03-G03.8, G04-G05.8, G14-G14.6, G21.3, I00, I02, I02.9, I98.1, K67.8, K75.3, K76.3, K77.0, M49.1, M89.6, P35-P35.9,P37, P37.2, P37.5-P37.9, U82-U84, U85-U89, Z16-Z16.3	020-029, 032-034, 034.1-034.9, 036-036.3, 036.5-037.9, 040, 040.1-041.0, 046-054.0, 054.2-059.9, 062-064.9, 070.0-070.2, 072-075.9, 078.3-078.7, 079-079.5, 079.7, 100-101.6, 104-104.9, 136-136.2, 139-139.0, 320.0-320.8, 321-323.9, 390-390.9, 392, 392.9, 484.0, 484.3-484.5, 771.0-771.3, V09-V09.9
Meningitis	A39-A39.9, A87-A87.9, G00.0-G00.8, G03-G03.8	036-036.3, 036.5-036.9, 047-049.9, 320.0-320.8, 321-322.9
Encephalitis	A83-A86.4, B94.1, F07.1, G04-G05.8, G21.3	062-064.9, 139.0, 323, 323.4-323.9
Diphtheria	A36-A36.9	032-032.9
Whooping cough	A37-A37.9	033-033.9, 484.3
Tetanus	A33-A35.0	037-037.9, 771.3
Measles	B05-B05.9	055-055.9, 484.0
Varicella and herpes zoster	B01-B02.9, P35.8	052-053.9
Acute hepatitis	B15-B16.2, B17.0, B17.2, B19.1, P35.3	070.0-070.2
Acute hepatitis A	B15-B15.9	070.0-070.1
Acute hepatitis B	B16-B16.2, B17.0, B19.1, P35.3	070.2
Acute hepatitis C		
Acute hepatitis E	B17.2	
Other unspecified infectious diseases	A20-A28.9, A32-A32.9, A38-A38.9, A48.2, A48.4-A48.5, A65-A65.0, A69-A69.1,A74, A74.8-A74.9, A81-A81.9, A88-A89.9, B00-B00.9, B03-B04, B06-B06.9, B10-B10.8, B25-B27.9, B29.4, B33, B33.3-B33.8, B47-B48.8, B91, B95-B95.5, D70.3, D89.3, F02.1, G14-G14.6, I00, I02, I02.9, I98.1, K67.8, K75.3, K76.3, K77.0, M49.1,M89.6, P35-P35.2, P35.9, P37, P37.2, P37.5-P37.9, U82-U84, U85-U89, Z16-Z16.3	020-029, 034, 034.1-034.9, 040, 040.1-041.0, 046-046.9, 050-051.9, 054-054.0, 054.2 054.9, 056-059.9, 072-075.9, 078.3-078.7, 079-079.5, 079.7, 100-101.6, 104-104.9, 136-136.2, 139, 323.0-323.3, 390-390.9, 392, 392.9, 484.4-484.5, 771.0-771.2, V09-V09.9
Maternal and neonatal disorders	C58-C58.0, N96, N98-N98.9, O00-O07.9, O09-O16.9, O20-O26.9, O28-O36.9, O40-O48.1, O60-O77.9, O80-O92.7, O96-O98.6, O98.8-P04.2, P04.5-P05.9, P07-P15.9, P19-P22.9, P24-P29.9, P36-P36.9, P38-P39.9, P50-P61.9, P70-P70.1, P70.3-P72.9,P74-P78.9, P80-P81.9, P83-P84, P90-P94.9, P96, P96.3-P96.4, P96.8	181-181.9, 630-636.9, 638-638.9, 640-679.1, 760-760.6, 760.8-768, 768.2-770, 770.1-771, 771.4-775.0, 775.4-779.3, 779.6-779.8
Maternal disorders	C58-C58.0, N96, N98-N98.9, O00-O07.9, O09-O16.9, O20-O26.9, O28-O36.9, O40-O48.1, O60-O77.9, O80-O92.7, O96-O98.6, O98.8-O99.9	181-181.9, 630-636.9, 638-638.9, 640-679.1
Maternal hemorrhage	O20-O20.9, O43.2, O44-O46.9, O62-O62.9, O67-O67.9, O70, O72-O72.3	640-641.9, 661-661.9, 665, 666-666.9
Maternal sepsis and other maternal infections	O23-O23.9, O85-O86.8, O91-O91.2	659.3, 670-670.9
Maternal hypertensive disorders	O10-O16.9	642-642.9
Maternal obstructed labor and uterine rupture	O32-O33.9, O64-O66.9, O71-O71.9	652-653.9, 660-660.9, 665.0-665.3
Maternal abortion and miscarriage	N96, O01-O07.9	630-632.9, 634-636.9, 638-638.9, 646.3
Ectopic pregnancy	O00-O00.9	633-633.9
Indirect maternal deaths	O24-O25.3, O98-O98.6, O98.8-O99.9	646-646.2, 646.4-649.9
Late maternal deaths	O96-O97.9	
Maternal deaths aggravated by HIV/AIDS		
Other maternal disorders	C58-C58.0, N98-N98.9, O09-O09.9, O21-O22.9, O26-O26.9, O28-O31.8, O34- O36.9, O40-O43.1, O43.8-O43.9, O47-O48.1, O60-O61.9, O63-O63.9, O68-O69.9,O70.0-O70.9, O73-O77.9, O80-O84.9, O87-O90.9, O92-O92.7	181-181.9, 643-645.2, 650-651.9, 654-659.2, 659.4-659.9, 662-664.9, 665.4-665.9, 667-669.9, 671-679.1
Neonatal disorders	P00-P04.2, P04.5-P05.9, P07-P15.9, P19-P22.9, P24-P29.9, P36-P36.9, P38-P39.9, P50-P61.9, P70-P70.1, P70.3-P72.9, P74-P78.9, P80-P81.9, P83-P84, P90-P94.9, P96,P96.3-P96.4, P96.8	760-760.6, 760.8-768, 768.2-770, 770.1-771, 771.4-775.0, 775.4-779.3, 779.6-779.8
Neonatal preterm birth	P01.0-P01.1, P07-P07.3, P22-P22.9, P25-P28.9, P61.2, P77-P77.9	761.0-761.1, 765-765.9, 769-769.9, 770.2-770.9, 776.6, 777.5-777.6
Neonatal encephalopathy due to birth asphyxia and trauma	P01.7, P02-P03.9, P10-P15.9, P20-P21.9, P24-P24.9, P90-P91.9	761.7-763.9, 767-768, 768.2-768.9, 770.1, 772.1-772.9, 779.0-779.2
Neonatal sepsis and other neonatal infections	P36-P36.9, P38-P39.9	771.4-771.9
Hemolytic disease and other neonatal jaundice	P55-P59.9	773-774.9
Other neonatal disorders	P00-P01, P01.2-P01.6, P01.8-P01.9, P04-P04.2, P04.5-P05.9, P08-P09, P19-P19.9, P29-P29.9, P50-P54.9, P60-P61.1, P61.3-P61.9, P70-P70.1, P70.3-P72.9, P74-P76.9,P78-P78.9, P80-P81.9, P83-P84, P92-P94.9, P96, P96.3-P96.4, P96.8	760-760.6, 760.8-761, 761.2-761.6, 764-764.9, 766-766.9, 770, 771, 772-772.0, 775-775.0, 775.4-776.5, 776.7-777.4, 777.7-779, 779.3, 779.6-779.8
Nutritional deficiencies	D50.1-D50.8, D51-D52.0, D52.8-D53.9, E00-E02, E40-E46.9, E51-E61.9, E63-E64.0,E64.2-E64.9, M12.1	244.2, 260-263.9, 265-269.9, 281.0-281.9, 716.0
Protein-energy malnutrition	E40-E46.9, E64.0	260-263.9
Other nutritional deficiencies	D51-D52.0, D52.8-D53.9, E00-E02, E51-E61.9, E63-E64, E64.2-E64.9, M12.1	244.2, 265-269.9, 281.0-281.9, 716.0

Appendix Table 6: List of International Classification of Diseases (ICD) codes mapped to the Global Burden of Disease cause list for causes of death

Cause	ICD10	ICD9

Non-communicable diseases	A46-A46.0, A66-A67.9, B18-B18.9, B33.2, B86, C00-C13.9, C15-C22.8, C23-C25.9,C30-C34.9, C37-C38.8, C40-C41.9, C43-C45.9, C47-C54.9, C56-C57.8, C60-C63.8, C64-C67.9, C68.0-C68.8, C69.0-C69.8, C70-C73.9, C75-C75.8, C81-C86.6, C88-C91.0, C91.2-C91.3, C91.6, C92-C92.6, C93-C93.1, C93.3, C93.8, C94-C96.9, D00.1 D00.2, D01.0-D01.3, D02.0-D02.3, D03-D06.9, D07.0-D07.2, D07.4-D07.5, D09.0, D09.2-D09.3, D09.8, D10.0-D10.7, D11-D12.9, D13.0-D13.7, D14.0-D14.3, D15-D16.9, D22-D27.9, D28.0-D28.7, D29.0-D29.8, D30.0-D30.8, D31-D36, D36.1-D36.7, D37.1-D37.5, D38.0-D38.5, D39.1-D39.2, D39.8, D40.0-D40.8, D41.0-D41.8, D42-D43.9, D44.0-D44.8, D45-D47.9, D48.0-D48.6, D49.2-D49.4, D49.6, D52.1, D55-D58.9, D59.0-D59.3, D59.5-D59.6, D60-D61.9, D63.1, D64.0, D66-D67, D68.0-D69.8, D70-D70.2, D70.4-D75.8, D76-D78.8, D86-D86.9, D89-D89.2, E03-E07.1, E09-E11.9, E15.0, E16.0-E16.9, E20-E34, E34.1-E34.8, E36-E36.8, E65-E68, E70- E85.2, E88-E89.9, F00-F02.0, F02.2-F02.3, F02.8-F03.9, F10-F16.9, F18-F18.9, F24, F50.0-F50.5, G10-G13.8, G20-G20.9, G21.0-G21.1, G23-G26.0, G30-G31.9, G35-G37.9, G40-G41.9, G45-G46.8, G47.3, G61-G61.9, G62.1, G70-G73.7, G90-G90.9, G93.7, G95-G95.9, G97-G97.9, H05.0-H05.1, I01-I01.9, I02.0, I05-I09.9, I11-I13.9, I20-I25.9, I27.0-I27.2, I28-I28.9, I30-I31.1, I31.8-I37.8, I38-I41.9, I42.1-I42.8, I43-I43.9, I47-I48.9, I51.0-I51.4, I60-I63.9, I65-I66.9, I67.0-I67.3, I67.5-I67.7, I68.0-I68.2, I69.0-I69.3, I70.2-I70.8, I71-I73.9, I77-I89.9, I95.2-I95.3, I97-I98, I98.2, I98.9,J30-J35.9, J37-J39.9, J41-J46.9, J60-J63.8, J65-J68.9, J70-J70.9, J82, J84-J84.9, J91, J91.8-J92.9, J95-J95.9, K20-K20.9, K22-K22.6, K22.8-K29.9, K31-K31.8, K35-K38.9, K40-K46.9, K50-K52.0, K52.2-K52.9, K55-K62.9, K63.5, K64-K64.9, K66.8, K67, K68, K70-K70.3, K71.7, K73-K75, K75.1-K75.2, K75.4-K76.2, K76.4-K77, K77.8, K80-K83.9, K85-K86.9, K90-K91.9, K92.8, K93.8-K95.8, L00-L05.9, L08-L08.9, L10-L14.0, L51-L51.9, L88-L89.9, L93-L93.2, L97-L98.4, M00-M03.0, M03.2-M03.6, M05-M09.8, M30-M36.8, M40-M43.1, M65-M65.0, M71.0-M71.1, M72.5-M72.6, M80-M82.8, M86.3-M86.4, M87-M87.1, M88-M89.0, M89.5, M89.7-M89.9, N00-N08.8, N10-N12.9, N13.6, N14-N16.8, N18-N18.9, N20-N23.0, N25-N28.1, N29-N30.3, N30.8-N32.0, N32.3-N32.4, N34-N34.3, N36-N36.9, N39-N39.2,N41-N41.9, N44-N44.0, N45-N45.9, N49-N49.9, N60-N60.9, N65-N65.1, N72- N72.0, N75-N77.8, N80-N81.9, N83-N83.9, N84.0-N84.1, N87-N87.9, N99-N99.9, P04.3-P04.4, P70.2, P96.0-P96.2, P96.5, Q00-Q07.9, Q10.4-Q18.9, Q20-Q28.9, Q30-Q36, Q37-Q45.9, Q50-Q87.8, Q89-Q89.8, Q90-Q93.9, Q95-Q99.8, R50.2, R78.0-R78.5, R95-R95.9, X45-X45.9, X65-X65.9, Y15-Y15.9	035-035.9, 036.4, 102-103.9, 133-133.6, 135-135.9, 140-148.9, 150-155.1, 155.3-158.9, 160-164.9, 170-175.9, 180-180.9, 182-183.8, 184.0-184.4, 184.8, 185-186.9, 187.1-187.8, 188-188.9, 189.0-189.8, 190-190.8, 191-193.9, 194.1-194.8, 200-204.0, 204.2, 205-205.3, 206-206.1, 207-208.9, 209.0-209.1, 209.4-209.5, 210.0-210.9, 211.0-211.8, 212.0-212.8, 213-213.9, 217-220.9, 221.0-221.8, 222.0-222.8, 223.0-223.8, 224-228.9, 229.0, 229.8, 230.1-230.8, 231.0-231.2, 232-232.9, 233.0-233.2, 233.4-233.5, 233.7, 234.0-234.8, 235.0, 235.4, 235.6-235.8, 236.0-236.2, 236.4-236.5, 236.7, 237-237.3, 237.5-237.9, 238.0-238.9, 239.2-239.4, 239.6, 240-243.9, 244.0-244.1, 244.3-244.8, 245-246.9, 251-259.1, 259.3-259.9, 270-273.9, 275-276, 277-277.2, 277.4-277.9, 278.0-278.8, 282-284.9, 286-286.5, 286.7-289.0, 289.4-289.7, 290-292.9, 294.1-294.9, 303-303.9, 304.0-304.8, 305.0, 305.2-305.8, 307.1, 327.2-327.8, 330-331.2, 331.5-332.0, 333-337.9, 340-341.9, 345-345.9, 349, 349.2-349.8, 353.8-353.9, 356-356.9, 357.0-357.1, 357.3-357.7, 358-359.9, 376.0-376.1, 391-391.9, 392.0, 393-398.9, 402-404.9, 410-414.9, 416.0-416.1, 417-417.9, 420-423, 423.1-423.9, 424.0-424.3, 424.8, 425.0-425.3, 425.5, 425.7-425.8, 427.0-427.3, 427.6-427.8, 429.0, 430-435.9, 437.0-437.2, 437.4-437.8, 440.2, 440.4, 441-443.9, 446-457, 457.1-457.9, 459, 459.1-459.3, 470, 470.9-474.9, 476-476.1, 477-479, 491-493.9, 495-504.9, 506-506.9, 508-509, 515, 516-517.8, 518.6-518.7, 518.9, 519.0-519.4, 530-530.0, 530.2-530.6, 531-536.1, 536.4, 537-537.6, 537.8, 538-543.9, 550-553.6, 555-558.9, 560-560.3, 560.8-560.9, 562-562.1, 564-564.7, 565-566.9, 569.0-569.7, 571-571.9, 572.2-573.0, 573.4-577.9, 579-583.9, 585-585.9, 588-590.9, 592-593.8, 594-599.6, 599.8, 601-602.9, 604-604.9, 608.2, 610-610.9, 617-618.9, 620-620.9, 621.4-621.9, 622.1-622.7, 629-629.8, 680-689, 694-695.5, 707-707.9, 710-711.9, 714-714.3, 714.8-714.9, 730.1, 732-732.9, 733.0-733.1, 740-749.0, 749.2-758.9, 759.0-759.8, 760.7, 775.1-775.3, 779.4-779.5, 788.0, 790.3, 798-798.0, E850,E860
Neoplasms	C00-C13.9, C15-C22.8, C23-C25.9, C30-C34.9, C37-C38.8, C40-C41.9, C43-C45.9,C47-C54.9, C56-C57.8, C60-C63.8, C64-C67.9, C68.0-C68.8, C69.0-C69.8, C70-C73.9, C75-C75.8, C81-C86.6, C88-C91.0, C91.2-C91.3, C91.6, C92-C92.6, C93-C93.1, C93.3, C93.8, C94-C96.9, D00.1-D00.2, D01.0-D01.3, D02.0-D02.3, D03-D06.9, D07.0-D07.2, D07.4-D07.5, D09.0, D09.2-D09.3, D09.8, D10.0-D10.7, D11-D12.9, D13.0-D13.7, D14.0-D14.3, D15-D16.9, D22-D24.9, D26.0-D27.9, D28.0-D28.1, D28.7, D29.0-D29.8, D30.0-D30.8, D31-D36, D36.1-D36.7, D37.1-D37.5, D38.0-D38.5, D39.1-D39.2, D39.8, D40.0-D40.8, D41.0-D41.8, D42-D43.9, D44.0-D44.8, D45-D47.9, D48.0-D48.6, D49.2-D49.4, D49.6, K62.0-K62.1, K63.5, N60-N60.9, N84.0-N84.1, N87-N87.9	140-148.9, 150-155.1, 155.3-158.9, 160-164.9, 170-175.9, 180-180.9, 182-183.8, 184.0-184.4, 184.8, 185-186.9, 187.1-187.8, 188-188.9, 189.0-189.8, 190-190.8, 191-193.9, 194.1-194.8, 200-204.0, 204.2, 205-205.3, 206-206.1, 207-208.9, 209.0-209.1, 209.4-209.5, 210.0-210.9, 211.0-211.8, 212.0-212.8, 213-213.9, 217-217.8, 219.0, 220-220.9, 221.0-221.8, 222.0-222.8, 223.0-223.8, 224-228.9, 229.0, 229.8, 230.1-230.8, 231.0-231.2, 232-232.9, 233.0-233.2, 233.4-233.5, 233.7, 234.0-234.8, 235.0, 235.4, 235.6-235.8, 236.1-236.2, 236.4-236.5, 236.7, 237-237.3, 237.5-237.9, 238.0-238.9, 239.2-239.4, 239.6, 569.0, 610-610.9, 622.1-622.2, 622.7
Lip and oral cavity cancer	C00-C08.9, D10.0-D10.5, D11-D11.9	140-145.9, 210.0-210.6, 235.0
Nasopharynx cancer	C11-C11.9, D10.6	147-147.9, 210.7-210.9
Other pharynx cancer	C09-C10.9, C12-C13.9, D10.7	146-146.9, 148-148.9
Esophageal cancer	C15-C15.9, D00.1, D13.0	150-150.9, 211.0, 230.1
Stomach cancer	C16-C16.9, D00.2, D13.1, D37.1	151-151.9, 211.1, 230.2
Colon and rectum cancer	C18-C21.9, D01.0-D01.3, D12-D12.9, D37.3-D37.5	153-154.9, 209.1, 209.5, 211.3-211.4, 230.3-230.6, 569.0
Liver cancer	C22-C22.8, D13.4	155-155.1, 155.3-155.9, 211.5
Liver cancer due to hepatitis B		
Liver cancer due to hepatitis C		
Liver cancer due to alcohol use		
Liver cancer due to NASH		
Hepatoblastoma	C22.2	
Liver cancer due to other causes (internal)		
Gallbladder and biliary tract cancer	C23-C24.9, D13.5	156-156.9
Pancreatic cancer	C25-C25.9, D13.6-D13.7	157-157.9, 211.6-211.7
Larynx cancer	C32-C32.9, D02.0, D14.1, D38.0	161-161.9, 212.1, 231.0, 235.6
Tracheal, bronchus, and lung cancer	C33-C34.9, D02.1-D02.3, D14.2-D14.3, D38.1	162-162.9, 212.2-212.3, 231.1-231.2, 235.7
Malignant skin melanoma	C43-C43.9, D03-D03.9, D22-D23.9, D48.5	172-172.9
Non-melanoma skin cancer	C44-C44.9, D04-D04.9, D49.2	173-173.9, 222.4, 232-232.9, 238.2

Non-melano6ma skin cancer (squamous-cell carcinoma)	C44-C44.9, D04-D04.9, D49.2	173-173.9, 222.4, 232-232.9, 238.2
Soft tissue and other extraosseous sarcomas	C49-C49.9	171-171.9

Malignant neoplasm of bone and articular cartilage	C40-C41.9	170-170.9
Breast cancer	C50-C50.9, D05-D05.9, D24-D24.9, D48.6, D49.3	174-175.9, 217-217.8, 233.0, 238.3, 239.3, 610-610.9
Cervical cancer	C53-C53.9, D06-D06.9, D26.0	180-180.9, 219.0, 233.1, 622.1-622.2, 622.7
Uterine cancer	C54-C54.9, D07.0-D07.2, D26.1-D26.9	182-182.9, 233.2
Ovarian cancer	C56-C56.9, D27-D27.9, D39.1	183-183.0, 220-220.9, 236.2
Prostate cancer	C61-C61.9, D07.5, D29.1, D40.0	185-185.9, 222.2, 236.5
Testicular cancer	C62-C62.9, D29.2-D29.8, D40.1-D40.8	186-186.9, 222.0, 222.3, 236.4
Kidney cancer	C64-C65.9, D30.0-D30.1, D41.0-D41.1	189.0-189.1, 189.5-189.6, 223.0-223.1
Bladder cancer	C67-C67.9, D09.0, D30.3, D41.4-D41.8, D49.4	188-188.9, 223.3, 233.7, 236.7, 239.4
Brain and central nervous system cancer	C70-C72.9	191-192.9
Eye cancer	C69.0-C69.8	190-190.8
Retinoblastoma	C69.2	190.5
Other eye cancers	C69.0-C69.1, C69.3-C69.8	190-190.4, 190.6-190.8

Appendix Table 6: List of International Classification of Diseases (ICD) codes mapped to the Global Burden of Disease cause list for causes of death

Cause	ICD10	ICD9
Neuroblastoma and other peripheral nervous cell tumors	C47-C47.9	
Thyroid cancer	C73-C73.9, D09.3, D09.8, D34-D34.9, D44.0	193-193.9, 226-226.9
Mesothelioma	C45-C45.9	
Hodgkin lymphoma	C81-C81.9	201-201.9
Non-Hodgkin lymphoma	C82-C86.6, C96-C96.9	200-200.9, 202-202.9
Burkitt lymphoma	C83.7-C83.8	200.2
Other non-Hodgkin lymphoma	C82-C83.6, C83.9-C86.6, C96-C96.9	200-200.1, 200.3-200.9, 202-202.9
Multiple myeloma	C88-C90.9	203-203.9
Leukemia	C91-C91.0, C91.2-C91.3, C91.6, C92-C92.6, C93-C93.1, C93.3, C93.8, C94-C95.9	204-204.0, 204.2, 205-205.3, 206-206.1, 207-208.9
Acute lymphoid leukemia	C91.0, C91.2-C91.3, C91.6	204.0, 204.2
Chronic lymphoid leukemia		
Acute myeloid leukemia	C92.0, C92.3-C92.6, C93.0, C94.0, C94.2, C94.4-C94.5	205.0, 205.2-205.3, 206.0, 207.0, 207.2-207.8
Chronic myeloid leukemia	C92.1-C92.2	205.1
Other leukemia	C93.1, C93.3, C93.8, C94.1, C94.3, C94.6-C95.9	206.1, 207.1, 207.9-208.9
Other malignant neoplasms (internal)	C17-C17.9, C30-C31.9, C37-C38.8, C48-C48.9, C4A, C51-C52.9, C57-C57.8, C60-C60.9, C63-C63.8, C66-C66.9, C68.0-C68.8, C75-C75.8, D07.4, D09.2, D13.2-D13.3, D14.0, D15-D16.9, D28.0-D28.1, D28.7, D29.0, D30.2, D30.4-D30.8, D31-D31.9, D35-D35.2, D35.5-D36, D36.1-D36.7, D37.2, D38.2-D38.5, D39.2, D39.8,D41.2-D41.3, D44.1-D44.8, D48.0-D48.4	152-152.9, 158-158.9, 160-160.9, 163-164.9, 183.2-183.8, 184.0-184.4, 184.8, 187.1-187.8, 189.2-189.4, 189.8, 194.1-194.8, 209.0, 209.4, 211.2, 211.8, 212.0, 212.4-212.8, 213-213.9, 221.0-221.8, 222.1, 222.8, 223.2, 223.8, 224-224.9, 227-228.9, 229.0, 229.8, 230.7-230.8, 233.4-233.5, 234.0-234.8, 235.4, 235.8, 236.1, 238.0-238.1, 239.2
Other neoplasms	D32-D33.9, D35.3-D35.4, D42-D43.9, D45-D47.9, D49.6, K62.0-K62.1, K63.5, N60-N60.9, N84.0-N84.1, N87-N87.9	225-225.9, 237-237.3, 237.5-237.9, 238.4-238.9, 239.6
Myelodysplastic, myeloproliferative, and other hematopoieticneoplasms	D45-D47.9	238.4-238.9
Cardiovascular diseases	B33.2, G45-G46.8, I01-I01.9, I02.0, I05-I09.9, I11-I11.9, I20-I25.9, I27.0, I27.2, I28-I28.9, I30-I31.1, I31.8-I37.8, I38-I41.9, I42.1-I42.8, I43-I43.9, I47-I48.9, I51.0-I51.4, I60-I63.9, I65-I66.9, I67.0-I67.3, I67.5-I67.6, I68.0-I68.2, I69.0-I69.3, I70.2-I70.8,I71-I73.9, I77-I83.9, I86-I89.0, I89.9, I98, K75.1	036.4, 391-391.9, 392.0, 393-398.9, 402-402.9, 410-414.9, 416.0, 417-417.9, 420-423, 423.1-423.9, 424.0-424.3, 424.8, 425.0-425.3, 425.5, 425.7-425.8, 427.0-427.3, 427.6-427.8, 429.0, 430-435.9, 437.0-437.2, 437.5-437.8, 440.2, 440.4, 441-443.9, 447-454.9, 456, 456.3-457, 457.1, 457.8-457.9, 459, 459.1-459.3
Rheumatic heart disease	I01-I01.9, I02.0, I05-I09.9	391-391.9, 392.0, 393-398.9
Ischemic heart disease	I20-I25.9	410-414.9
Stroke	G45-G46.8, I60-I63.9, I65-I66.9, I67.0-I67.3, I67.5-I67.6, I68.1-I68.2, I69.0-I69.3	430-435.9, 437.0-437.2, 437.5-437.8
Ischemic stroke	G45-G46.8, I63-I63.9, I65-I66.9, I67.2-I67.3, I67.5-I67.6, I69.3	433-435.9, 437.0-437.1, 437.5-437.8
Intracerebral hemorrhage	I61-I62, I62.1-I62.9, I68.1-I68.2, I69.1-I69.2	431-432.9, 437.2
Subarachnoid hemorrhage	I60-I60.9, I62.0, I67.0-I67.1, I69.0	430-430.9
Hypertensive heart disease	I11-I11.9	402-402.9
Non-rheumatic valvular heart disease	I34-I37.8	424.0-424.3, 424.8
Non-rheumatic calcific aortic valve disease	I35-I35.9	424.1
Non-rheumatic degenerative mitral valve disease	I34-I34.9	424.0
Other non-rheumatic valve diseases	I36-I37.8	424.2-424.3, 424.8
Cardiomyopathy and myocarditis	B33.2, I40-I41.9, I42.1-I42.8, I43-I43.9, I51.4	422-422.9, 425.0-425.3, 425.5, 425.7-425.8, 429.0
Myocarditis	B33.2, I40-I41.9, I51.4	422-422.9
Alcoholic cardiomyopathy	I42.6	425.5
Other cardiomyopathy	I42.1-I42.5, I42.7-I42.8, I43-I43.9	425.0-425.3, 425.7-425.8, 429.0
Pulmonary Arterial Hypertension	I27.0, I27.2	416.0
Atrial fibrillation and flutter	I48-I48.9	427.3
Aortic aneurysm	I71-I71.9	441-441.9
Peripheral artery disease	I70.2-I70.8, I73-I73.9	440.2, 440.4, 443.0-443.9
Endocarditis	I33-I33.9, I38-I39.9	421-421.9
Other cardiovascular and circulatory diseases (internal)	I28-I28.9, I30-I31.1, I31.8-I32.8, I47-I47.9, I51.0-I51.3, I68.0, I72-I72.9, I77-I83.9,I86-I89.0, I89.9, I98, K75.1	036.4, 417-417.9, 420-420.9, 423, 423.1-423.9, 427.0-427.2, 427.6-427.8, 442-443, 447-454.9, 456, 456.3-457, 457.1, 457.8-457.9, 459, 459.1-459.3
Chronic respiratory diseases	D86-D86.2, D86.9, G47.3, J30-J35.9, J37-J39.9, J41-J46.9, J60-J63.8, J65-J68.9, J70,J70.8-J70.9, J82, J84-J84.9, J91, J91.8-J92.9	135-135.9, 327.2-327.8, 470, 470.9-474.9, 476-476.1, 477-479, 491-493.9, 495-504.9, 506-506.9, 508-509, 515, 516-517.8, 518.6, 518.9, 519.1-519.4

Chronic obstructive pulmonary disease	J41-J44.9	491-492.9, 496-499
Pneumoconiosis	J60-J63.8, J65-J65.0, J92.0	500-504.9
Silicosis	J62-J62.9	502-502.9, 503.0, 503.9
Asbestosis	J61-J61.0, J92.0	501
Coal workers pneumoconiosis	J60-J60.0	500-500.9, 501.0-501.9
Other pneumoconiosis	J63-J63.8, J65-J65.0	503, 503.1, 504-504.9
Asthma	J45-J46.9	493-493.9
Interstitial lung disease and pulmonary sarcoidosis	D86-D86.2, D86.9, J84-J84.9	135-135.9, 515, 516-516.9
Other chronic respiratory diseases	G47.3, J30-J35.9, J37-J39.9, J66-J68.9, J70, J70.8-J70.9, J82, J91, J91.8-J92, J92.9	327.2-327.8, 470, 470.9-474.9, 476-476.1, 477-479, 495-495.9, 506-506.9, 508-509, 517-517.8, 518.6, 518.9, 519.1-519.4
Digestive diseases	B18-B18.9, I84-I85.9, I98.2, K20-K20.9, K22-K22.6, K22.8-K29.9, K31-K31.8, K35-K38.9, K40-K42.9, K44-K46.9, K50-K52, K52.2-K52.9, K55-K62, K62.2-K62.6, K62.8-K62.9, K64-K64.9, K66.8, K67, K68, K70-K70.3, K71.7, K73-K75, K75.2, K75.4-K76.2, K76.4-K77, K77.8, K80-K83.9, K85-K86.9, K90-K90.9, K92.8, K93.8,M09.1	455-455.9, 456.0-456.2, 530-530.0, 530.2-530.6, 531-536.1, 537-537.6, 537.8, 538, 540-543.9, 550-551.1, 551.3-552.1, 552.3-553.6, 555-558.9, 560-560.3, 560.8-560.9, 562-562.1, 564-564.1, 564.5-564.7, 565-566.9, 569.1-569.5, 569.7, 571-571.9, 572.2-573.0, 573.4-577.9, 579-579.2, 579.4-579.9
Cirrhosis and other chronic liver diseases	B18-B18.9, I85-I85.9, I98.2, K70-K70.3, K71.7, K73-K75, K75.2, K75.4-K76.2,K76.4-K76.9, K77.8	456.0-456.2, 571-571.9, 572.2-573.0, 573.4-573.9
Cirrhosis and other chronic liver diseases due to hepatitis B		
Cirrhosis and other chronic liver diseases due to hepatitis C		
Cirrhosis and other chronic liver diseases due to alcohol use		

Achoki T, *et al. J Epidemiol Community Health* 2022; 76:471–481. doi: 10.1136/jech-2021-217480

Appendix Table 6: List of International Classification of Diseases (ICD) codes mapped to the Global Burden of Disease cause list for causes of death

Cause	ICD10	ICD9
Cirrhosis and other chronic liver diseases due to NAFLD		
Cirrhosis and other chronic liver diseases due to other causes		
Upper digestive system diseases	K25-K29.9	531-535.9
Peptic ulcer disease	K25-K28.9	531-534.9
Gastritis and duodenitis	K29-K29.9	535-535.9
Appendicitis	K35-K37.9, K38.3-K38.9	540-542.9
Paralytic ileus and intestinal obstruction	K56-K56.9	560-560.3, 560.8-560.9
Inguinal, femoral, and abdominal hernia	K40-K42.9, K44-K46.9	550-551.1, 551.3-552.1, 552.3-553.0, 553.6
Inflammatory bowel disease	K50-K52, K52.8-K52.9, M09.1	555-556.9, 558-558.9, 569.5
Vascular intestinal disorders	K55-K55.9	557-557.9
Gallbladder and biliary diseases	K80-K83.9	574-576.9
Pancreatitis	K85-K86.9	577-577.9, 579.4
Other digestive diseases	I84-I84.9, K20-K20.9, K22-K22.6, K22.8-K24, K31-K31.8, K38-K38.2, K52.2- K52.3, K57-K62, K62.2-K62.6, K62.8-K62.9, K64-K64.9, K66.8, K67, K68, K77,K90-K90.9, K92.8, K93.8	455-455.9, 530-530.0, 530.2-530.6, 536-536.1, 537-537.6, 537.8, 538, 543-543.9, 553.1-553.3, 562-562.1, 564-564.1, 564.5-564.7, 565-566.9, 569.1-569.4, 569.7, 579- 579.2, 579.8-579.9
Neurological disorders	F00-F02.0, F02.2-F02.3, F02.8-F03.9, G10-G13.8, G20-G20.9, G23-G24, G24.1-G25.0, G25.2-G25.3, G25.5, G25.8-G26.0, G30-G31.1, G31.8-G31.9, G35-G37.9, G40-G41.9, G61-G61.9, G70-G71.1, G71.3-G72, G72.2-G73.7, G90-G90.9, G95-G95.9, M33-M33.9	290-290.9, 294.1-294.9, 330-331.2, 331.5-332.0, 333-337.9, 340-341.9, 345-345.9, 349, 349.2-349.8, 353.8-353.9, 356-356.9, 357.0-357.1, 357.3-357.4, 357.7, 358-359.9, 775.2
Alzheimer's disease and other dementias	F00-F02.0, F02.8-F03.9, G30-G31.1, G31.8-G31.9	290-290.9, 294.1-294.9, 331-331.2
Parkinson's disease	F02.3, G20-G20.9	332-332.0
Idiopathic epilepsy	G40-G41.9	345-345.9
Multiple sclerosis	G35-G35.9	340-340.9
Motor neuron disease	G12.2-G12.9	335-335.2, 335.8-335.9
Other neurological disorders	F02.2, G10-G12.1, G13-G13.8, G23-G24, G24.1-G25.0, G25.2-G25.3, G25.5, G25.8- G26.0, G36-G37.9, G61-G61.9, G70-G71.1, G71.3-G72, G72.2-G73.7, G90-G90.9,G95-G95.9, M33-M33.9	330-330.9, 331.5-331.9, 333-334.9, 335.3, 336-337.9, 341-341.9, 349, 349.2-349.8, 353.8-353.9, 356-356.9, 357.0-357.1, 357.3-357.4, 357.7, 358-359.9, 775.2
Mental disorders	F24, F50.0-F50.5	307.1
Eating disorders	F50.0-F50.5	307.1
Anorexia nervosa	F50.0-F50.1	307.1
Bulimia nervosa	F50.2-F50.5	
Substance use disorders	E24.4, F10-F16.9, F18-F18.9, G31.2, G62.1, G72.1, P04.3-P04.4, P96.1, Q86.0,R78.0-R78.5, X45-X45.9, X65-X65.9, Y15-Y15.9	291-292.9, 303-303.9, 304.0-304.8, 305.0, 305.2-305.8, 357.5, 760.7, 790.3, E850,E860
Alcohol use disorders	E24.4, F10-F10.9, G31.2, G62.1, G72.1, P04.3, Q86.0, R78.0, X45-X45.9, X65-X65.9, Y15-Y15.9	291-291.9, 303-303.9, 305.0, 357.5, 790.3, E860
Drug use disorders	F11-F16.9, F18-F18.9, P04.4, P96.1, R78.1-R78.5	292-292.9, 304.0-304.8, 305.2-305.8, 760.7, E850
Opioid use disorders	F11-F11.9, P96.1, R78.1	304.0, 305.5
Cocaine use disorders	F14-F14.9, R78.2	304.2, 305.6
Amphetamine use disorders	F15-F15.9	304.4, 305.7
Other drug use disorders	F13-F13.9, F16-F16.9, F18-F18.9, P04.4, R78.3-R78.5	292-292.9, 304.1, 304.5-304.8, 305.3-305.4, 305.8, 760.7
Diabetes and kidney diseases	D63.1, E10-E11.9, I12-I13.9, N00-N08.8, N15.0, N18-N18.9, P70.2, Q61-Q62.8	403-404.9, 580-583.9, 585-585.9, 589-589.9, 753-753.3, 775.1
Diabetes mellitus	E10-E10.1, E10.3-E11.1, E11.3-E11.9, P70.2	775.1
Diabetes mellitus type 1	E10-E10.1, E10.3-E10.9, P70.2	775.1
Diabetes mellitus type 2	E11-E11.1, E11.3-E11.9	
Chronic kidney disease	D63.1, E10.2, E11.2, I12-I13.9, N02-N08.8, N15.0, N18-N18.9, Q61-Q62.8	403-404.9, 581-583.9, 585-585.9, 589-589.9, 753-753.3
Chronic kidney disease due to diabetes mellitus type 1	E10.2	
Chronic kidney disease due to diabetes mellitus type 2	E11.2	
Chronic kidney disease due to hypertension	I12-I13.9	403-404.9
Chronic kidney disease due to glomerulonephritis	N03-N06.9	581-583.9
Chronic kidney disease due to other and unspecified causes	N02-N02.9, N07-N08.8, N15.0, Q61-Q62.8	589-589.9, 753-753.3

Acute glomerulonephritis	N00-N01.9	580-580.9
Skin and subcutaneous diseases	A46-A46.0, A66-A67.9, B86, D86.3, I89.1-I89.8, L00-L05.9, L08-L08.9, L10-L14.0,L51-L51.9, L88-L89.9, L97-L98.4, M72.5-M72.6	035-035.9, 102-103.9, 133-133.6, 457.2-457.3, 680-689, 694-695.3, 707-707.9
Bacterial skin diseases	A46-A46.0, A66-A67.9, I89.1-I89.8, L00-L05.9, L08-L08.9, L88, L97-L98.4, M72.5-M72.6	035-035.9, 102-103.9, 457.2-457.3, 680-689
Cellulitis	L03-L03.9, M72.5-M72.6	681-682.9
Pyoderma	A46-A46.0, A66-A67.9, I89.1-I89.8, L00-L02.9, L04-L05.9, L08-L08.9, L88, L97-L98.4	035-035.9, 102-103.9, 457.2-457.3, 680-680.9, 683-689
Decubitus ulcer	L89-L89.9	707-707.9
Other skin and subcutaneous diseases	D86.3, L10-L14.0, L51-L51.9	694-695.3
Musculoskeletal disorders	I27.1, I67.7, L93-L93.2, M00-M03.0, M03.2-M03.6, M05-M09.0, M09.2-M09.8, M30 M32.9, M34-M36.8, M40-M43.1, M65-M65.0, M71.0-M71.1, M80-M82.8, M86.3-M86.4, M87-M87.0, M88-M89.0, M89.5, M89.7-M89.9	416.1, 437.4, 446-446.9, 695.4-695.5, 710-711.9, 714-714.3, 714.8-714.9, 730.1, 732- 732.9, 733.0-733.1
Rheumatoid arthritis	M05-M06.9, M08.0-M08.8	714-714.3, 714.8-714.9
Other musculoskeletal disorders	I27.1, I67.7, L93-L93.2, M00-M03.0, M03.2-M03.6, M07-M08, M08.9-M09.0, M09.2 M09.8, M30-M32.9, M34-M36.8, M40-M43.1, M65-M65.0, M71.0-M71.1, M80-M82.8, M86.3-M86.4, M87-M87.0, M88-M89.0, M89.5, M89.7-M89.9	416.1, 437.4, 446-446.9, 695.4-695.5, 710-711.9, 730.1, 732-732.9, 733.0-733.1

Appendix Table 6: List of International Classification of Diseases (ICD) codes mapped to the Global Burden of Disease cause list for causes of death

Cause	ICD10	ICD9
Other non-communicable diseases	D25-D26, D28.2, D52.1, D55-D58.9, D59.0-D59.3, D59.5-D59.6, D60-D61.9, D64.0, D66-D67, D68.0-D69.8, D70-D70.2, D70.4-D75.8, D76-D78.8, D86.8, D89-D89.2, E03-E07.1, E09-E09.9, E15.0, E16.0-E16.9, E20-E24.3, E24.8-E34, E34.1-E34.8, E36 E36.8, E65-E68, E70-E85.2, E88-E89.9, G21.0-G21.1, G24.0, G25.1, G25.4, G25.6- G25.7, G71.2, G72.0, G93.7, G97-G97.9, I95.2-I95.3, I97-I97.9, I98.9, J70.0-J70.5, J95-J95.9, K43-K43.9, K52.0, K62.7, K91-K91.9, K94-K95.8, M87.1, N10-N12.9, N13.6, N14-N15, N15.1-N16.8, N20-N23.0, N25-N28.1, N29-N30.3, N30.8-N32.0, N32.3-N32.4, N34-N34.3, N36-N36.9, N39-N39.2, N41-N41.9, N44-N44.0, N45-N45.9, N49-N49.9, N65-N65.1, N72-N72.0, N75-N77.8, N80-N81.9, N83-N83.9, N99-N99.9, P96.0, P96.2, P96.5, Q00-Q07.9, Q10.4-Q18.9, Q20-Q28.9, Q30-Q36,Q37-Q45.9, Q50-Q60.6, Q63-Q86, Q86.1-Q87.8, Q89-Q89.8, Q90-Q93.9, Q95- Q99.8, R50.2, R95-R95.9	218-219, 219.1-219.9, 236.0, 240-243.9, 244.0-244.1, 244.3-244.8, 245-246.9, 251- 259.1, 259.3-259.9, 270-273.9, 275-276, 277-277.2, 277.4-277.9, 278.0-278.8, 282- 284.9, 286-286.5, 286.7-289.0, 289.4-289.7, 357.6, 518.7, 519.0, 536.4, 539-539.9, 551.2, 552.2, 564.2-564.4, 569.6, 579.3, 588-588.9, 590-590.9, 592-593.8, 594-599.6, 599.8, 601-602.9, 604-604.9, 608.2, 617-618.9, 620-620.9, 621.4-621.9, 622.3-622.6, 629-629.8, 740-749.0, 749.2-752.9, 753.4-758.9, 759.0-759.8, 775.3, 779.4-779.5, 788.0, 798-798.0
Congenital birth defects	G71.2, P96.0, Q00-Q07.9, Q10.4-Q18.9, Q20-Q28.9, Q30-Q36, Q37-Q45.9, Q50-Q60.6, Q63-Q86, Q86.1-Q87.8, Q89-Q89.8, Q90-Q93.9, Q95-Q99.8	740-749.0, 749.2-752.9, 753.4-758.9, 759.0-759.8
Neural tube defects	Q00-Q01.9, Q05-Q05.9	740-741.9, 742.0
Congenital heart anomalies	Q20-Q28.9	745-747.9
Orofacial clefts	Q35-Q36, Q37-Q37.9	749-749.0, 749.2-749.9
Down syndrome	Q90-Q90.9	758.0
Other chromosomal abnormalities	Q87-Q87.8, Q91-Q93.9, Q95-Q95.9, Q97-Q97.9, Q99-Q99.8	758, 758.1-758.6, 758.8-758.9
Congenital musculoskeletal and limb anomalies	Q65-Q79, Q79.6-Q79.9	742.5, 754-756.5, 756.8-756.9
Urogenital congenital anomalies	P96.0, Q50-Q60.6, Q63-Q64.9	752-752.9, 753.4-753.9
Digestive congenital anomalies	Q38-Q45.9, Q79.0-Q79.5	750-751.9, 756.6-756.7
Other congenital birth defects	G71.2, Q02-Q04.9, Q06-Q07.9, Q10.4-Q18.9, Q30-Q34.9, Q80-Q86, Q86.1-Q86.8,Q89-Q89.8	742, 742.1-742.4, 742.8-744.9, 748-748.9, 757-757.9, 759.0-759.8
Urinary diseases and male infertility	N10-N12.9, N13.6, N15, N15.1-N16.8, N20-N23.0, N25-N28.1, N29-N30.3, N30.8- N32.0, N32.3-N32.4, N34-N34.3, N36-N36.9, N39-N39.2, N41-N41.9, N44-N44.0,N45-N45.9, N49-N49.9	588-588.9, 590-590.9, 592-593.8, 594-598.1, 598.8-599.6, 599.8, 601-602.9, 604- 604.9, 608.2, 788.0
Urinary tract infections	N10-N12.9, N13.6, N15, N15.1-N16.8, N30-N30.3, N30.8-N30.9, N34-N34.3, N39.0-N39.2	590-590.9, 595-595.9, 597-597.9, 599.0
Urolithiasis	N20-N23.0	592-592.9, 594-594.9, 788.0
Other urinary diseases	N25-N28.1, N29-N29.8, N31-N32.0, N32.3-N32.4, N36-N36.9, N39, N41-N41.9,N44-N44.0, N45-N45.9, N49-N49.9	588-588.9, 593-593.8, 596-596.9, 598-598.1, 598.8-599, 599.1-599.6, 599.8, 601- 602.9, 604-604.9, 608.2
Gynecological diseases	D25-D26, D28.2, E28.2, N72-N72.0, N75-N77.8, N80-N81.9, N83-N83.9	218-219, 219.1-219.9, 236.0, 256.4, 617-618.9, 620-620.9, 621.4-621.9, 622.3-622.6, 629-629.8
Uterine fibroids	D25-D26, D28.2	218-219, 219.1-219.9, 236.0
Endometriosis	N80-N80.9	617-617.9
Genital prolapse	N81-N81.9	618-618.9
Other gynecological diseases	N72-N72.0, N75-N77.8, N83-N83.9	620-620.9, 621.4-621.9, 622.3-622.6, 629-629.8
Hemoglobinopathies and hemolytic anemias	D55-D58.9, D59.1, D59.3, D59.5, D60-D61.9, D64.0	282-284.9
Thalassemias	D56-D56.9	282.4-282.5
Sickle cell disorders	D57-D57.8	282.6
G6PD deficiency	D55-D55.2	282.2-282.3
Other hemoglobinopathies and hemolytic anemias	D55.3-D55.9, D58-D58.9, D59.1, D59.3, D59.5, D60-D61.9, D64.0	282-282.1, 282.7-284.9

Achoki T, *et al. J Epidemiol Community Health* 2022; 76:471–481. doi: 10.1136/jech-2021-217480

Endocrine, metabolic, blood, and immune disorders	D52.1, D59.0, D59.2, D59.6, D66-D67, D68.0-D69.8, D70-D70.2, D70.4-D75.8, D76- D78.8, D86.8, D89-D89.2, E03-E07.1, E09-E09.9, E15.0, E16.0-E16.9, E20-E24.3, E24.8-E28.1, E28.3-E34, E34.1-E34.8, E36-E36.8, E65-E68, E70-E85.2, E88-E89.9, G21.0-G21.1, G24.0, G25.1, G25.4, G25.6-G25.7, G72.0, G93.7, G97-G97.9, I95.2- I95.3, I97-I97.9, I98.9, J70.0-J70.5, J95-J95.9, K43-K43.9, K52.0, K62.7, K91-K91.9, K94-K95.8, M87.1, N14-N14.4, N65-N65.1, N99-N99.9, P96.2, P96.5, R50.2	240-243.9, 244.0-244.1, 244.3-244.8, 245-246.9, 251-256.3, 256.8-259.1, 259.3-259.9, 270-273.9, 275-276, 277-277.2, 277.4-277.9, 278.0-278.8, 286-286.5, 286.7- 289.0, 289.4-289.7, 357.6, 518.7, 519.0, 536.4, 539-539.9, 551.2, 552.2, 564.2-564.4, 569.6, 579.3, 598.2, 775.3, 779.4-779.5
Sudden infant death syndrome	R95-R95.9	798-798.0
Injuries	L55-L55.9, L56.3, L56.8-L56.9, L58-L58.9, N30.4, U00-U03, V00-V86.9, V87.2-V87.3, V88.2-V88.3, V90-V98.8, W00-W46.2, W49-W62.9, W64-W70.9, W73-W75.9, W77-W81.9, W83-W94.9, W97.9, W99-X06.9, X08-X39.9, X47-X48.9, X50- X54.9, X57-X58.9, X60-X64.9, X66-X83.9, X85-Y08.9, Y35-Y84.9, Y87.0-Y87.1,Y88-Y88.3, Y89.0-Y89.1	349.0-349.1, 457.0, E800-E807, E830-E838, E840-E849, E856-E857, E861-E865,E867-E869, E870-E876, E878-E879, E880-E886, E888-E928, E930- E979, E990- E999
Transport injuries	V00-V86.9, V87.2-V87.3, V88.2-V88.3, V90-V98.8	E800-E807, E830-E838, E840-E849
Road injuries	V01-V04.9, V06-V80.9, V82-V82.9, V87.2-V87.3	
Pedestrian road injuries	V01-V04.9, V06-V09.9	
Cyclist road injuries	V10-V19.9	
Motorcyclist road injuries	V20-V29.9	
Motor vehicle road injuries	V30-V79.9, V87.2-V87.3	
Other road injuries	V80-V80.9, V82-V82.9	
Other transport injuries	V00-V00.8, V05-V05.9, V81-V81.9, V83-V86.9, V88.2-V88.3, V90-V98.8	E800-E807, E830-E838, E840-E849
Unintentional injuries	L55-L55.9, L56.3, L56.8-L56.9, L58-L58.9, N30.4, W00-W46.2, W49-W62.9, W64- W70.9, W73-W75.9, W77-W81.9, W83-W94.9, W97.9, W99-X06.9, X08-X39.9,X47-X48.9, X50-X54.9, X57-X58.9, Y40-Y84.9, Y88-Y88.3	349.0-349.1, 457.0, E856-E857, E861-E865, E867-E869, E870-E876, E878-E879,E880-E886, E888-E928, E930-E949
Falls	W00-W19.9	E880-E886, E888
Drowning	W65-W70.9, W73-W74.9	E910
Fire, heat, and hot substances	X00-X06.9, X08-X19.9	E890-E899, E924
Poisonings	X47-X48.9	E856-E857, E861-E865, E867-E869
Poisoning by carbon monoxide	X47-X47.9	E862, E868-E869
Poisoning by other means	X48-X48.9	E856-E857, E861, E863-E865, E867
Exposure to mechanical forces	W20-W38.9, W40-W43.9, W45.0-W45.2, W46-W46.2, W49-W52	E916-E922
Unintentional firearm injuries	W32-W34.9	E922
Other exposure to mechanical forces	W20-W31.9, W35-W38.9, W40-W43.9, W45.0-W45.2, W46-W46.2, W49-W52	E916-E921

Achoki T, *et al. J Epidemiol Community Health* 2022; 76:471–481. doi: 10.1136/jech-2021-217480

Appendix Table 6: List of International Classification of Diseases (ICD) codes mapped to the Global Burden of Disease cause list for causes of death

Cause	ICD10	ICD9
Adverse effects of medical treatment	N30.4, Y40-Y84.9, Y88-Y88.3	349.0-349.1, 457.0, E870-E876, E878-E879, E930-E949
Animal contact	W52.0-W62.9, W64-W64.9, X20-X29.9	E905-E906
Venomous animal contact	X20-X29.9	E905
Non-venomous animal contact	W52.0-W62.9, W64-W64.9	E906
Foreign body	W44-W45, W45.3-W45.9, W75-W75.9, W78-W80.9, W83-W84.9	E911-E915
Pulmonary aspiration and foreign body in airway	W75-W75.9, W78-W80.9, W83-W84.9	E911-E913
Foreign body in other body part	W44-W45, W45.3-W45.9	E914-E915
Environmental heat and cold exposure	L55-L55.9, L56.3, L56.8-L56.9, L58-L58.9, W88-W94.9, W97.9, W99-W99.9, X30-X32.9, X39-X39.9	E900-E902, E926
Exposure to forces of nature	X33-X38.9	E907-E909
Still Born	P95-P95.9	768.0-768.1
Other unintentional injuries	W39-W39.9, W77-W77.9, W81-W81.9, W85-W87.9, X50-X54.9, X57-X58.9	E903-E904, E923, E925, E927-E928
Self-harm and interpersonal violence	U00-U03, X60-X64.9, X66-X83.9, X85-Y08.9, Y35-Y38.9, Y87.0-Y87.1, Y89.0-Y89.1	E950-E979, E990-E999
Self-harm	X60-X64.9, X66-X83.9, Y87.0	E950-E959
Self-harm by firearm	X72-X74.9	E955
Self-harm by other specified means	X60-X64.9, X66-X71.9, X75-X83.9, Y87.0	E950-E954, E956-E959
Interpersonal violence	X85-Y08.9, Y87.1	E960-E969
Physical violence by firearm	X93-X95.9	E965
Physical violence by sharp object	X99-X99.9	E966
Physical violence by other means	X85-X92.9, X96-X98.9, Y00-Y04.9, Y06-Y08.9, Y87.1	E961-E964, E967-E969
Conflict and terrorism	U00-U03, Y36-Y38.9, Y89.1	E979, E990-E999
Executions and police conflict	Y35-Y35.9, Y89.0	E970-E978
Garbage Code (GBD Level 1)	A40-A41.9, A48.0, A48.3, A49.0-A49.1, A59-A59.9, A71-A71.9, A74.0, B07- B07.9, B30-B30.9, B35-B36.9, B85-B85.4, B87-B88.9, B94.0, D50-D50.0, D50.9, D62- D63.0, D63.8-D64, D64.1-D65.9, D68, D69.9, E15, E16, E50-E50.9, E64.1, E85.3- E87.6, E87.8-E87.9, F06.2-F06.4, F07.2, F09-F09.9, F19-F23.9, F25-F49, F51- F99.0, G06-G08.0, G32-G32.8, G43-G44.2, G44.4-G44.8, G47-G47.2, G47.4-G47.9, G50- G60.9, G62-G62.0, G62.2-G65.2, G80-G83.9, G89-G89.4, G91-G91.2, G91.4- G93, G93.1-G93.2, G93.4-G93.6, G94.0-G94.8, G99-H05, H05.2-H69.9, H71-H99, I26- I26.9, I31.2-I31.4, I46-I46.9, I50.0-I50.4, I76, I95-I95.1, I95.8-I95.9, J69-J69.9, J80- J80.9, J81.0, J85-J85.3, J86-J86.9, J93-J93.1, J93.8-J93.9, J94.2, J96-J96.9, J98.1- J98.3, K00-K19, K30, K65-K66.1, K66.9, K68.1-K68.9, K71-K71.6, K71.8- K72.9, K75.0, L20-L30.9, L40-L50.9, L52-L54.8, L56-L56.2, L56.4-L56.5, L57-L57.9, L59- L68.9, L70-L76.8, L80-L87.9, L90-L92.9, L94-L96, L98.5-L99.8, M04, M10- M12.0, M12.2-M29, M37-M39, M43.2-M49, M49.2-M64, M65.1-M71, M71.2- M72.4, M72.8 M73, M73.8-M79.9, M83-M86.2, M86.5-M86.9, M87.2- M87.9, M89.1-M89.4, M90- M99.9, N17-N17.9, N19-N19.9, N32.1-N32.2, N32.8-N33.8, N35-N35.9, N37- N37.8, N39.3-N39.8, N42-N43.4, N44.1-N44.8, N46-N48.9, N50-N53.9, N61-N64.9, N82- N82.9, N91-N91.5, N95, N95.1-N95.9, N97-N97.9, R02-R02.9, R03.1, R07.0, R08- R09, R09.3, R11-R12.0, R14-R19.6, R19.8-R23, R23.1-R30.9, R32-R50.1, R50.8- R57.9, R58.0-R72.9, R74-R78, R78.6-R94.8, R96-R99.9, U05, U07-U81, U89.9-U99,X40-X44.9, X46-X46.9, X49-X49.9, Y10-Y14.9, Y16-Y19.9, Z00- Z15.8, Z17-unsp.	038-038.9, 040.0, 041.1, 076-078.2, 110-111.9, 125-125.3, 126-126.9, 127.2- 127.9, 131-132.9, 133.8-134.9, 136.6, 139.1, 139.9, 247-248, 264-264.9, 274-274.9, 276.0- 276.5, 276.7-276.9, 277.3, 280-281, 285-285.9, 286.6, 289.1-289.3, 293, 294- 294.0, 295-302.9, 305, 305.9-307.0, 307.2-307.4, 307.6-319.9, 324-327.1, 328-329, 338- 339.1, 339.3-339.8, 342-344.9, 346-346.9, 350-353.6, 354-355.9, 360-362, 362.1- 376, 376.2-380.9, 384-389.9, 415-415.9, 423.0, 424, 424.4-424.5, 424.9, 427.5, 427.9 428.9, 437.3, 458-458.9, 459.0, 507-507.9, 510-510.9, 512-513.9, 518.1-518.2, 520- 529.9, 536.3, 536.8-536.9, 537.7, 537.9, 564.8-564.9, 567-568.9, 570-570.9, 572- 572.1, 573.1-573.3, 584-584.9, 586-587.9, 603-603.9, 605-608.1, 608.3-609, 611- 612.1, 615-616.9, 619-619.9, 621-621.3, 622-622.0, 622.8-623.6, 623.8-624.5, 624.8- 628.9, 629.9, 690-693.9, 695.8-706.9, 708-709.9, 712-713.8, 715-716, 716.2- 721.6, 721.8-730.0, 730.2-730.3, 730.7-731.9, 733, 733.2-734.2, 737-738, 738.2-739.9, 780- 782.4, 782.6-784.6, 784.9, 785.4-786, 786.6, 786.8, 787, 787.3-788, 788.3-789, 789.1 789.2, 789.5, 790-790.1, 790.4-796.1, 796.3-797.9, 798.1-799, 799.2-799.9, 999.0- 999.9, E851-E855, E858, E866, E980-E982, V01-V08, V10-uns
	A72-A73, A76, A97, B08-B09, B11-B14, B28-B29, B31-B32.4, B34-B34.9,	000-000.9, 030-030.9, 041.2-041.9, 067-069, 078.8-078.9, 079.8-079.9, 089- 089.9, 105-109.9, 119, 136.8-136.9, 139.8, 304, 304.9, 305.1, 339.2, 401-401.9, 405- 405.9, 416, 416.2-416.9, 440-440.1, 440.3, 440.8-440.9, 444-445.8, 490-490.9, 494- 494.9, 511-511.9, 514-514.9, 515.0-515.9, 518-518.0, 518.3-518.5, 518.8, 536.2, 578- 578.9, 599.7, 613-614.9, 714.4, 716.1, 721.7, 735-736.9, 738.0-738.1, 784.7-784.8, 786.3, 787.0-787.2, 789.0, 789.3-789.4, 789.6-789.9, 796.2, 799.0-799.1, 800-999, E000-E80, E83, E839, E85, E859, E87, E877, E88, E887, E929, E983-E985, E988-E989
	B61-B62, B68-B68.9, B73-B74.2, B76-B76.9, B78-B81.8, B84, B92-B94,	
	B94.8-B94.9, B95.6-B97.3, B97.7-B99.9, D59, D59.4, D59.8-D59.9, F17-F17.9,	
Garbage Code (GBD Level 2)	G44.3, G91.3, G93.0, G93.3, I10-I10.9, I15-I15.9, I27, I27.8-I27.9, I50, I50.8-I50.9, I67.4, I70-I70.1,	
	I70.9,	
	I74-I75.8, J81, J81.1, J90-J90.0, J94-J94.1, J94.8-J94.9, K92.0-K92.2, N70-	
	N71.9,	
	N73-N74.0, N74.2-N74.8, R03-R03.0, R04-R06.9, R09.0-R09.2, R09.8-R10.9,	
	R13- R13.9, R23.0, R58, S00-T98.3, W47-W48, W63, W71-W72, W76-W76.9,	
	W82, W95-W97, W98, X07, X55-X56, X59-X59.9, Y20-Y34.9, Y86-Y87, Y87.2, Y89, Y89.9- Y99.9	

Garbage Code (GBD Level 3)	A01, A31-A31.9, A42-A44.9, A49.2, A64-A64.0, A99-A99.0, B17, B17.1, B17.8- B17.9, B19-B19.0, B19.2-B19.9, B37-B46.9, B49-B49.9, B55, B55.1-B55.9, B58-B59.9, B89, B94.2, C14-C14.9, C22.9, C26-C29, C35-C36, C39-C39.9, C42, C46- C46.9, C55-C55.9, C57.9, C59, C63.9, C68, C68.9, C74-C74.9, C75.9-C80.9, C87, C97-D00.0, D01, D01.4-D02, D02.4-D02.9, D07, D07.3, D07.6-D09, D09.1, D09.7, D09.9-D10, D10.9, D13, D13.9-D14, D14.4, D17-D21.9, D28, D28.9-D29, D29.9- D30, D30.9, D36.0, D36.9-D37.0, D37.6-D38, D38.6-D39.0, D39.7, D39.9- D40, D40.9-D41, D41.9, D44, D44.9, D48, D48.7-D49.1, D49.5, D49.7-D49.9, D54, D75.9, D79-D85, D87-D88, D89.8-D99, E07.8-E08.9, E17-E19, E34.0, E34.9- E35.8,E37-E39, E47-E49., E62, E69, E87.7, E90-E998, F04-F06.1, F06.5-F07.0, F07.8-F08,F50, F50.8-F50.9, G09-G09.9, G15-G19, G21, G21.2, G21.4-G22.0, G27-G29, G33- G34, G38-G39., G42, G48-G49, G66-G69, G74-G79, G84-G88, G93.8-G94, G96- G96.9, G98-G98.9, I00.0, I03-I04., I14-I14., I16-I19, I29- I29.9, I44-I45.9, I49-I49.9, I51, I51.6-I59, I90-I94, I96-I96.9, I98.4-I98.8, I99-ID5.9, J02.9, J03.9, J04.3, J06, J06.9, J40-J40.9, J47-J59, J71-J79, J81.9, J83, J85.9, J87-J89, J90.9, J93.6, J97-J98.0,J98.4-J99.8, K21-K21.9, K22.7, K31.9-K34, K39, K47-K49, K53- K54, K63-K63.4, K63.8-K63.9, K69, K70.4-K70.9, K78-K79, K84, K87-K89, K92, K92.9-K93, K96- K99, L06-L07, L09, L15-L19, L31-L39, L69, L77-L79, N09, N13-N13.5, N13.7- N13.9, N24, N28.8-N28.9, N38, N39.9-N40.9, N54- N59, N66-N69, N78-N79, N84, N84.2-N86, N88-N90.9, N92-N94.9, N95.0, O08-O08.9, O17-O19, O27, O37-O39, O49-O59, O78-O79, O93-O95.9, P06, P16-P18, P30-P34.2, P40-P49, P62-P69, P73, P79, P82, P85-P89, P96.9-P99.9, Q08-Q10.3, Q19, Q29-Q29., Q36.0-Q36.9, Q46- Q49, Q88, Q89.9, Q94, Q99.9- R01.2, R07, R07.1-R07.9, R31-R31.9	002, 031-031.9, 039-039.9, 070, 070.4-070.9, 085, 085.1-085.9, 088.0-088.7, 112- 118.9, 130-130.9, 136.3-136.5, 149-149.9, 155.2, 159-159.9, 165-169, 176-179.9, 183.9-184, 184.5, 184.9, 187, 187.9, 189, 189.9, 190.9, 195-199.9, 209, 209.2- 209.3, 209.6-210, 211, 211.9-212, 212.9, 214-216.9, 221, 221.9-222, 222.9-223, 223.9, 229, 229.1, 229.9-230.0, 230.9-231, 231.8-231.9, 233, 233.3, 233.6, 233.9-234, 234.9- 235, 235.1-235.3, 235.5, 235.9-236, 236.3, 236.6, 236.9, 237.4, 239-239.1, 239.5, 239.7- 239.9, 249-249.9, 259.2, 276.6, 278, 279-279.9, 293.0-293.9, 331.3-331.4, 332.1- 332.9, 347-348.9, 349.9, 357, 357.8-357.9, 399-400.0, 406-409.4, 418-419.9, 426- 427, 427.4, 429, 429.2-429.9, 459.5-459.9, 464.5, 465, 465.9, 505-505.9, 519, 519.8- 519.9, 530.1, 530.7-530.9, 544-549, 553.8-553.9, 559-559.0, 560.4-560.7, 561, 562.2- 563, 569, 569.8-569.9, 591-591.9, 593.9, 599.9-600.9, 623.7, 624.6, 637-637.9, 639- 639.9, 749.1, 759, 759.9, 779.9, 782.5, 785-785.3, 786.0-786.2, 786.4-786.5, 786.7, 786.9, 788.1-788.2, E986-E987

Appendix Table 6: List of International Classification of Diseases (ICD) codes mapped to the Global Burden of Disease cause list for causes of death

Cause	ICD10	ICD9
	B16.9, B64, B82-B82.9, B83.9, C69, C69.9, C91.1, C91.4-C91.5, C91.7-C91.9,	
	C92.7-C92.9, C93.2, C93.5-C93.7, C93.9, E12-E14.9, G00, G00.9-G02.8, G03.9,	070.3, 084, 084.6, 194-194.0, 194.9, 204.1, 204.5-204.9, 205.8-205.9, 206.2- 206.9,
Garbage Code (GBD Level 4)	I37.9, I42-I42.0, I42.9, I51.5, I64-I64.9, I67, I67.8-I68, I68.8-I69, I69.4-I69.9, J07- J08, J15.9, J17-J19.6, J22-J29, J64-J64.9, P23, P23.5-P23.9, P37.3-P37.4, R73- R73.9,	238, 244, 244.9, 250-250.9, 289.8-289.9, 307.5, 320, 320.9, 357.2, 362.0, 425, 425.4, 425.9, 429.1, 436-437, 437.9-439.6, 482.9-483, 484, 484.8-486.9, 770.0, 790.2, E808-
	V87-V87.1, V87.4-V88.1, V88.4-V89.9, V99-V99.0, X84-X84.9, Y09-Y09.9, Y85-	E829
	Y85.9	
